# Late-Stage Functionalization of a Hexa-*peri*-Benzocoronene–Fluoranthene Hybrid Influenced by Dynamic Inversion of Helicity

**DOI:** 10.1021/acsphyschemau.6c00115

**Published:** 2026-08-13

**Authors:** Lina M. Filla, Alexander R. Krappe, Jacob C. Mayer, Jan M. Sühlo, Larissa S. Eitelhuber, Anja Wiesner, Lukasz Polewski, Ute Resch-Genger, Beate Paulus, Emil J. W. List-Kratochvil, Siegfried Eigler

**Affiliations:** † Institut für Chemie und Biochemie (SupraFAB), 54203 Freie Universität Berlin, Altensteinstraße 23a, Berlin 14195, Germany; ‡ 9151 Helmholtz-Zentrum Berlin für Materialien und Energie GmbH, Hahn-Meitner-Platz 1, Berlin 14109, Germany; § Institut für Physik, Institut für Chemie, Humboldt-Universität zu Berlin, Zum Großen Windkanal 2, Berlin 12489, Germany; ∥ Institut für Chemie und Biochemie, Freie Universität Berlin, Arnimallee 22, Berlin 14195, Germany; ⊥ Institut für Chemie und Biochemie, 9166 Freie Universität Berlin, Fabeckstraße 34/36, Berlin 14195, Germany; # Bundesanstalt für Materialforschung und -prüfung (BAM), Department 1, Division Biophotonics, Richard-Willstätter-Straße 11, Berlin 12489, Germany; ¶ Institut für Physik, Institut für Chemie, Center for the Science of Materials Berlin, Humboldt-Universität zu Berlin, Zum Großen Windkanal 2, Berlin 12489, Germany

**Keywords:** hydrocarbons, dyes, halogenation, helicity, OLED

## Abstract

The functionalization of a hexa-*peri*-benzocoronene–fluoranthene hybrid with a K-type bay region is investigated. Bromination proceeds regioselectively at two peripheral positions, which contradicts the electronic and structural predictions suggesting that substitution at the K-type bay region should be favored. Computational studies reveal that the transition state and intermediate energies for all substitution positions are comparable, though no substitution in the bay region is observed. To rationalize this unexpected regioselectivity, a model is proposed based on dynamic helical inversion, which prevents the corresponding Wheland intermediates from being stabilized. Subsequent conversion of the brominated compounds to nitrile derivatives affords compounds with photoluminescence quantum yields of up to 76% in solution. Organic light-emitting diodes with luminance values of up to 6,500 cd·m^–2^ are realized due to the effective energy level alignment by a hole-transport and electron-blocking layer, which was not possible with the related nonfunctionalized derivative.

## Graphical abstract

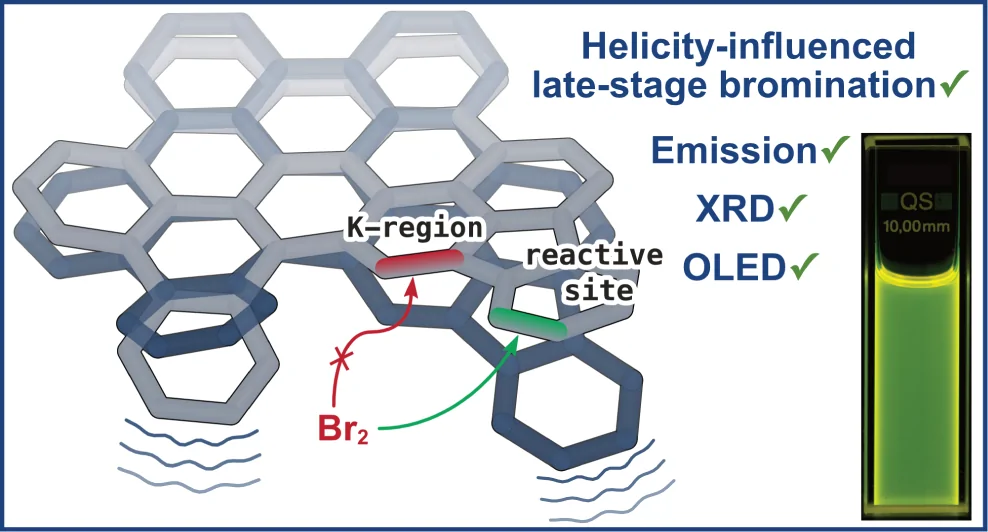

## Introduction

Research on polycyclic aromatic hydrocarbons (PAHs) dates back more than 100 years. Clar and coworkers’ systematic research starting in the 1930s provided the first coherent framework for understanding PAH aromaticity,^1–3^ ultimately giving rise to Clar’s rule.^4^ To date, PAH chemistry has evolved from Müllen and his research group’s pioneering syntheses of large-area, planar nanographenes in the early 2000s^5–7^ to topological structures^8,9^ and curved and asymmetric systems.^10–12^ The potential of nanographenes for a wide range of advanced applications is currently being investigated. These applications include catalysis,^13,14^ chemical sensing,^15–17^ and high-performance optoelectronic technologies such as organic field-effect transistors (OFETs),^18^ photovoltaic devices,^19^ memory phototransistors,^20^ and organic light-emitting diodes (OLEDs).^21,22^ While disc-shaped and symmetric structures such as hexa-*peri-*benzocoronene (HBC)^23^ are usually barely soluble and exhibit weak photoluminescence with a quantum yield of around 3%, introducing asymmetry, curvature, or helicity can have a significant impact on their solubility and optoelectronic properties.^22,24–29^

Within the variety of PAHs, helicenes are an interesting subclass bearing a helical topology caused by the steric repulsion of *o*-fused benzene rings. One remarkable property of these molecules is their high optical rotation values and, coupled to chromophores, the ability to emit circularly polarized luminescence (CPL).^30^ Recently, it was shown that helicenes and helical nanographenes exhibit chiral-induced spin selectivity (CISS), which can be used in molecular spintronics, making them interesting candidates for future data storage or quantum applications.^31,32^

When it comes to functionalization of these systems, the use of harsh reaction conditions like strong oxidants in combination with strong acids in the Scholl oxidation^33^ or oxidative photochemical cyclizations^34^ can limit functional group tolerance. However, modifying functional groups can significantly impact their electronic properties, which is essential for their use in optoelectronic applications. Therefore, late-stage functionalization is a desirable strategy for the tuning of PAHs and especially helicenes. Among the available late-stage synthetic methods, halogenation is one of the most common methods of functionalizing PAHs. This allows a variety of building blocks to be connected, for instance, via palladium-catalyzed cross-coupling or by lithium-halogen exchange. While iodine is usually too unreactive and controlled chlorination is typically carried out as perchlorination in order to prevent the formation of inseparable product mixtures and to reduce reactivity,^35^ bromination of PAH systems is easier to handle. The backbone of carbohelicenes can relatively easily be functionalized at its K-region,^36–38^ similar to the bromination of phenanthrene in the K-region, which essentially behaves like a double bond^39^ and has become a classic example used to explain Clar’s aromatic sextet rule (Figure 1).^4^ However, the K-regions of known helicenes only enable functionalization at the outer backbone, while reactions at the inner helix, namely, in the 1-position, are rarely observed.^36,40^

**Figure 1:**
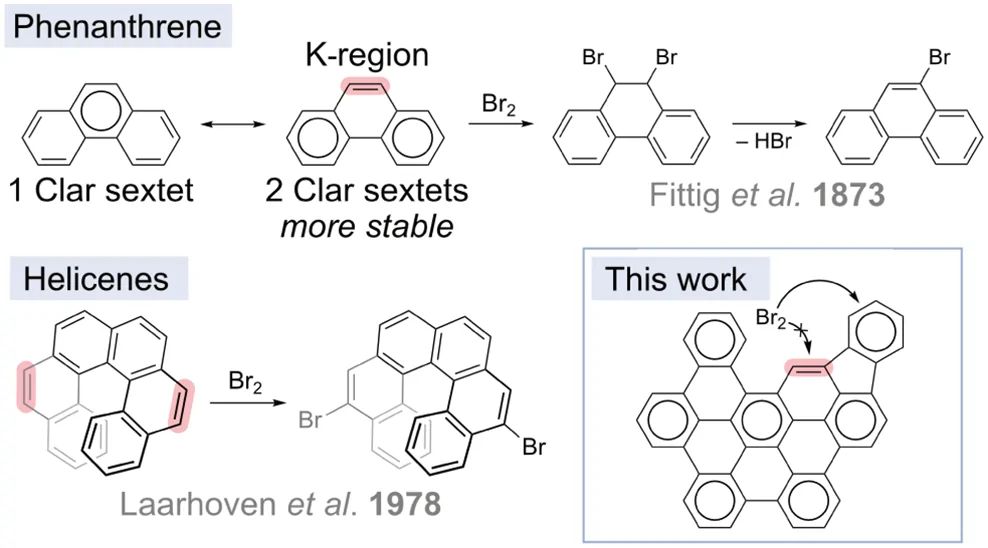
Schematic illustration explaining the reactivity of phenanthrene and [6]­helicene in the K-region upon bromination in contrast to the unusual bromination presented in this work. The circles in the structures indicate benzenoid rings according to Clar.

Recently, we demonstrated that introducing asymmetry by starting with a bisalkyne rather than a tolane derivative leads to a “symmetry-broken” nanographene **2** with a fluorescence quantum yield of up to 67% in toluene.^22,25^ We not only demonstrated the fluorescent properties of compound **2** but also showed that it can be used as a green emitter in OLEDs. In contrast to other nanographenes, our system is one of the few systems exhibiting a K-region in the inner helix.^41,42^ Apart from that, expanded helicenes also bear nonquaternary carbon atoms at the inner helix,^43^ but substitution at these positions has only been explored using prefunctionalized precursors.^44^

In this study, we investigated the bromination of a [4]­helicene nanographene with a K-type region in its inner-helicene topology. Contrary to our expectations, the intended bromination did not occur in the K-region, defying conventional trends, but selectively at two positions on the outer edge of the bay area. On the basis of computational studies, we propose a mechanistic model based on the dynamic inversion of the helix, which inhibits the formation of a stable Wheland intermediate, thus preventing bromination in the bay area. We exploit this distinctive behavior of our system to introduce nitrile groups at a late stage, which allows for an improved OLED performance compared to the devices built from the nonfunctionalized emitter material.

## Methods and Experimental Details

### General Information

All reagents were purchased from commercial sources and were used without further purification unless otherwise stated. All air- and moisture-sensitive reactions were performed under the standard Schlenk technique using nitrogen as an inert gas. Dry DCM was obtained from MBraun Solvent Purification System MB-SPS 800 filled with Al_2_O_3_. Dry DMF over molecular sieves and bromine were purchased from Thermo Fisher Scientific. Copper cyanide was purchased from Acros Organics. Thin-layer chromatography was performed on ALUGRAM Xtra SIL G/UV254 plates from Macherey-Nagel. Column chromatography was performed with Silica 60 M (0.04–0.063 mm) from Macherey-Nagel.

The compounds were characterized by NMR spectroscopy (Bruker AVANCE 500, JEOL ECZ 600, Bruker AVANCE 700) at 25 °C. IR spectra were recorded on a FT-IR Spectrum Two with a LiTaO_3_ detector from PerkinElmer. HR-EI mass spectra were measured on a Waters Autospec Premier mass spectrometer. UV/vis spectra were recorded on an Agilent Technologies Cary 8454 spectrometer. Photoluminescence spectra of solutions were recorded on a FL 6500 Fluorescence Spectrometer from PerkinElmer. The photoluminescence spectra of thin films were obtained on a FLS980 spectrometer from Edinburgh Instruments with excitation at 470 nm. Photoelectron yield spectroscopy (PYS) measurements were performed on a RIKEN KEIKI instrument, model AC/2E DC1 in air. Photoluminescence quantum yields were determined absolutely with an integrating sphere setup from Hamamatsu (Quantaurus-QY C11347–11). The fluorescence lifetime was recorded on a fluorometer FLS 920 (Edinburgh Instruments) equipped with a Hamamatsu R3809U-50 (range 200–850 nm, response width <25 ps), a multichannel plate (MCP) detector, Czerny–Turner double monochromators, and an Edinburgh Instruments EPL-375 (picosecond-pulsed light-emitting diode) for excitation at 375 nm. Single-crystal X-ray diffraction data were collected on a Bruker D8 VENTURE diffractometer with a Bruker PHOTON II detector using Cu*K*_α_ radiation (λ = 1.54178 Å). The supplementary crystallographic data for compounds **3a** and **3b** can be found in the CCDC under the deposit number 2512288. These data can be obtained free of charge from www.ccdc.cam.ac.uk/data_request/cif.

Spectroscopic analysis and measurement details are provided in the Supporting Information.

### Synthesis

#### Compounds **3a** and **3b**

Compound **2** (0.107 g, 121 μmol, 1.0 equiv) was dissolved in DCM (10 mL) and cooled to 0 °C. A solution of bromine (0.141 g, 882 μmol, 7.3 equiv) in DCM (2.5 mL) was added dropwise, and the mixture was stirred for 4.5 h at 0 °C. The reaction was quenched with aq. NaHSO_3_ (10 mL). The phases were separated, and the organic phase was washed with NaHSO_3_ (10 mL) and water (10 mL). The aqueous phase was extracted with DCM (2× 10 mL). The combined organic phases were dried over MgSO_4_, and the solvent was removed under reduced pressure. The products **3a** and **3b** were yielded as a mixture (0.114 g, 118 μmol, 94%). The crude product was used without further purification.

#### Compounds **4a** and **4b**

Under a nitrogen atmosphere, the mixture of compounds **3a** and **3b** (53.7 mg, 55.7 μmol, 1.0 equiv) and CuCN (20 mg, 223 μmol, 4.0 equiv) were dissolved in DMF (7.0 mL). The mixture was stirred for 3 d at 153 °C and subsequently quenched with brine (10 mL). The aqueous phase was extracted with EtOAc (2× 20 mL) and DCM (2× 20 mL). The combined organic layers were washed with brine (20 mL) and water (2× 20 mL) and dried over MgSO_4_. The solvent was removed under reduced pressure and the crude product was purified by column chromatography (cyclohexane/DCM 3:1; R_f_ = 0.46 (**4a**); R_f_ = 0.36 (**4b**)), yielding **4a** (16.8 mg, 18.5 μmol, 36%) and **4b** (18.8 mg, 20.7 μmol, 45%) as orange solids in a total yield of 81%.

### Computational Details

Quantum chemical calculations were performed using ORCA (versions 6.0.1 and 6.1.0) to investigate the reaction mechanism of the bromination.^45^ Structure optimizations were performed at the ωB97X-D3/def2-SVP level of density functional theory (DFT) using default convergence criteria.^46–48^ Additional single-point calculations on the optimized structures were performed at the ωB97X-D3BJ/def2-TZVP level of theory. The optimized structures were validated with frequency analysis. Solvent effects were considered by using the Conductor-like Polarizable Continuum Model (CPCM) with dichloromethane (*ε* = 8.93) as solvent.^49^ Time-dependent DFT (TD-DFT) calculations were performed without TDA approximation, using several functionals for benchmarking, based on the optimized geometries.^50,51^ The electron densities of delocalized and localized π-electrons and Wiberg bond indices were calculated using the programs Gaussian 16 ^52^ and NBO 7.0 ^53^ according to the provided workflow.^54–56^ Fukui functions^57^ and dual descriptor were calculated using the programs Multiwfn^58,59^ and Gaussian 16.^52^ The generated electron density surfaces were plotted with UCSF ChimeraX.^60^

### Device Fabrication

OLEDs were produced on prepatterned indium tin oxide (ITO) substrates (Psiotec Ltd.) which were cleaned by sonicating them first in acetone and then in isopropanol. As a last cleaning step and to activate the surface, the substrates were treated with an oxygen plasma for 5 min. PEDOT:PSS (Al 4083, Ossila) as a hole injection material was spin-coated on the substrates (1,000 rpm, 12 s; 2,000 rpm, 30 s) and annealed at 220 °C for 15 min to form a 50–60 nm thick layer. As a hole-transport and electron-blocking layer, poly­(9,9-dioctylfluorene-*alt*-*N*-(4-*sec*-butylphenyl)-diphenylamine) (TFB) was spin-coated on top of the PEDOT:PSS from a 3 mg/mL toluene solution at 6,000 rpm for 45 s. The film was dried in a vacuum oven at 200 °C for 1 h. The emitter layer was spin-coated from 3 mg/mL solutions of either molecule **4a** or **4b** in chloroform, sometimes with the addition of a host material, as indicated in Table S12. Pinhole-free films of 50–60 nm thickness were achieved with a rotation speed of 4,000 rpm for 30 s. The top contact consisting of a 5 nm calcium and 200 nm aluminum layer was fabricated *via* thermal evaporation. A design with eight pixels with an active area of 0.04 cm^2^ each was created using a shadow mask. The devices were encapsulated with UV-curable resin (Ossila) and glass coverslips.

## Results and Discussion

The unsubstituted pentaphene **2** was synthesized by Scholl oxidation of pentaarylalkynylbenzene **1** as reported previously.^22^ Bromination of compound **2** using elemental bromine yielded **3a** and **3b** in a combined yield of 92% (Scheme 1). Unexpectedly, the bromination did not occur in the K-type region in the bay area, the most reactive site of the molecule according to Mulliken population analysis (Table S1) with the shortest outer bond as depicted by the bond length alternation and Wiberg bond indices (Figure S1) as well as a large coefficient of the highest occupied molecular orbital (HOMO) (Figure S2). Instead, it reacted in the peripheral area, specifically at positions 15 and 16 (see Scheme 1). The bromination proceeds without the addition of iron powder as a catalyst, which indicates that these positions are activated aromatic units. Rapid conversion was also observed using glass pipettes instead of metal cannulas to exclude traces of iron. A theoretically possible dibromination could be ruled out by mass spectrometry of the product mixture, as both the fragmentation and the isotope patterns confirmed monobrominated isomers. We were able to control the ratio of compounds by varying the reaction temperature (Table S8). While at room temperature and slightly elevated temperatures, compound **3b** is dominant, at −40 °C and below, a 1:1 ratio is obtained. The resulting ^1^H NMR spectrum from the bromination shows two sets of signals in a ratio of 1:2.5, indicating the yield ratio of compounds **3a** and **3b** (Figure 2A). A detailed discussion of the NMR spectra can be found in the Supporting Information (Chapter 4, Figures S8–S9). However, the compounds could not be separated at this stage due to their similar polarity.

**Scheme 1:**
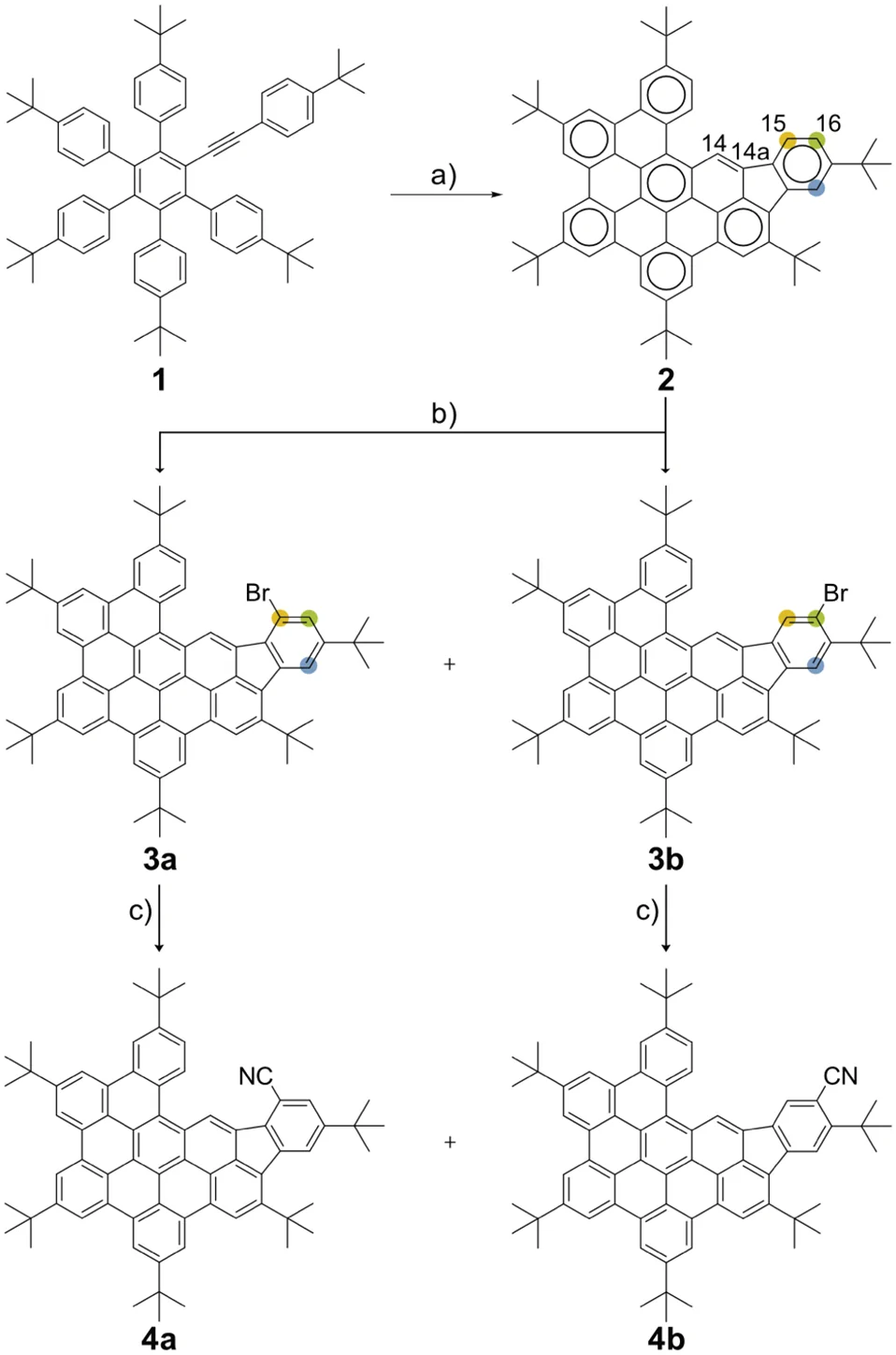
Synthetic Route to Nitrile Compounds **4a** and **4b** *via* Bromination. a) FeCl_3_, DCM, MeNO_2_, 0 °C, 32 h; b) Br_2_, DCM, 0 °C, 4.5 h; c) CuCN, DMF, 153 °C, 3 d. Atom Numbering According to IUPAC-Numbering of the Scaffold^a^

a Circles in structure **2** indicate benzenoid rings according to Clar.

**Figure 2:**
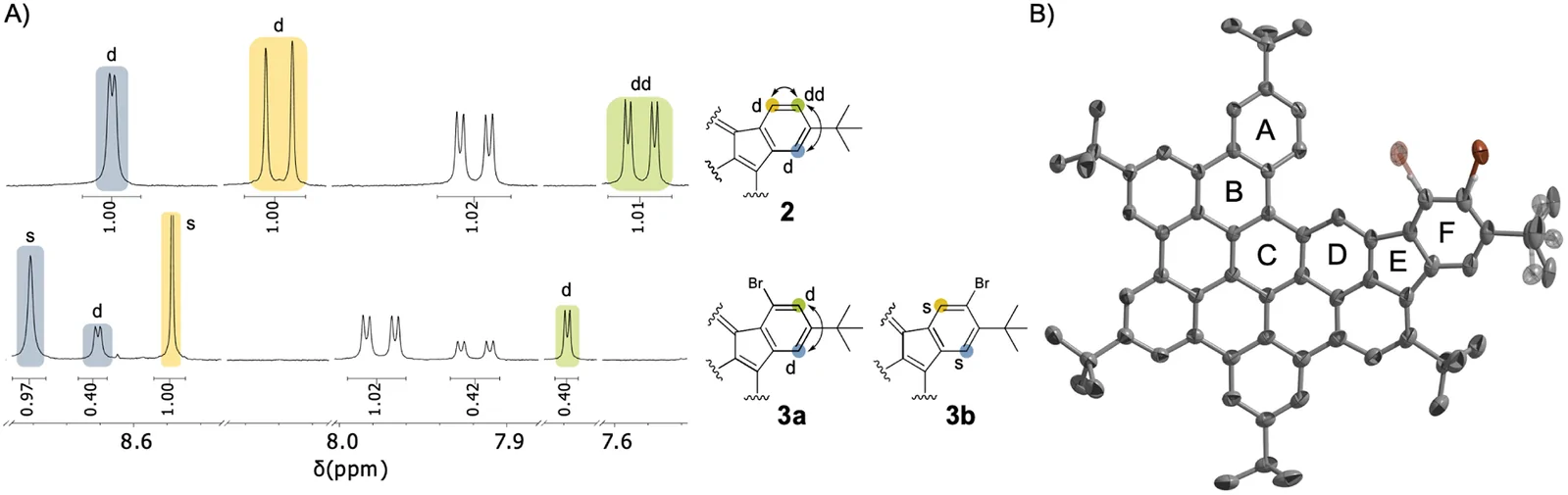
A) ^1^H NMR spectra of compounds **2** (top) and **3a** and **3b** (bottom); B) Molecular structure of single-crystal X-ray of cocrystallized compounds **3a** and **3b** in the solid state; solvent molecules omitted for clarity (C: gray, H: white, Br: red). Thermal ellipsoids are drawn at a 50% probability level; minor part of positional disorder displayed transparent.

The separation of the regioisomers by column chromatography is not possible as both compounds have the same retention factor in a variety of investigated eluents (Table S9). Attempts to separate the isomers by partial recrystallization were not successful, since no difference in solubility was observed. Further, the reaction with other reagents like iodine, ICl, or BrCN to yield a single product was not successful. Subsequently, the bromides underwent substitution *via* a Rosenmund–von Braun reaction, yielding the nitriles **4a** (36%) and **4b** (45%) in a total yield of 81%. We note that compounds **4a** and **4b** could be separated by column chromatography due to their different dipole moments, resulting from nitrile formation.

Single crystals suitable for single-crystal X-ray diffraction analysis were obtained by vapor diffusion of MeOH into a solution of the compound mixture **3a/b** in DCM (Figures 2B and S10–S12). Both regioisomers cocrystallized at the same lattice position in a sandwich-herringbone type^61^ in the space group *P*2_1_/*n*, with two molecules per asymmetric unit. While isomer **3b** occupies the major part of lattice positions, in line with the product ratio in solution, isomer **3a** is observed in the form of positional disorder in one molecule in the asymmetric unit. Further, both (*P*)- and (*M*)-enantiomers are visible in the solid state with an interplanar distance of 3.61 Å (Figure S13) and a helical torsion angle of approximately 34°. As calculated, the bromination at the outer bay region has no effect on the rotational barrier, which is about 11 kcal/mol for **3a** and **3b** and identical to the nonfunctionalized compound **2** (Figure S5). Thus, a separation of enantiomers from solution is not possible.

The unexpected substitution pattern raises questions about the reaction mechanism. As bromination at ring F occurs at positions 15 or 16, a 1,2-dibromination followed by HBr elimination can be ruled out as the mechanism. This is also supported by the respective bond lengths of 1.397(3) Å and 1.384(4) Å for the (*P*)- and (*M*)-enantiomer in the single-crystal X-ray diffraction structure of precursor **2**, which are significantly longer than classical double bonds and also than the bond of the K-region of phenanthrene (1.372 Å).^62^ As mentioned earlier, the Mulliken population analysis and the comparably short bond length of 1.348(3) Å between atoms 14 and 14a of compound **2** and a Wiberg bond index of 1.596, almost resembling a classical C=C double bond, led us to expect the most reactive site of the molecule to be at the K-type region (ring D) (Figure S1). This 14–14a bond length is even shorter than that reported for unsubstituted fluoranthene (1.367 Å).^63^ While magnetic quantification methods for aromaticity like nucleus-independent chemical shift (NICS) and the anisotropy of the induced current density (AICD) show a reduced but still diatropic ring current,^22^ the electron densities of delocalized and localized π-electrons (EDDB_π_ and EDLB_π_)^54–56^ reveal a strongly local nature of the π-electrons in contrast to the delocalized π-electrons of the benzenoid rings (Figure S3). Additionally, both the Fukui function *f* ^–^(*r*),^57^ which characterizes reactive sites toward electrophiles, and the negative dual descriptor Δ*f*(*r*) show a pronounced contribution on the bond under discussion (Figure S4). Despite this double bond character, a classical 1,2-dibromination on the atoms 14 and 14a is unlikely because it would generate two adjacent *sp* ^3^-hybridized carbon centers in the bay region, resulting in substantial strain and loss of aromatic stabilization.

To solve this puzzle, we opted for bromination *via* an electrophilic aromatic substitution mechanism and studied the formation of the Wheland intermediate, which is the reversible and rate-determining step in this process, *via* density functional theory (DFT) at the ωB97X-D3BJ/def2-TZVP level of theory with DCM as the solvent. Figure 3 depicts the energy profile for the formation of the Wheland intermediate at positions 15 and 16, respectively, which lead to the observed bromination products.

**Figure 3:**
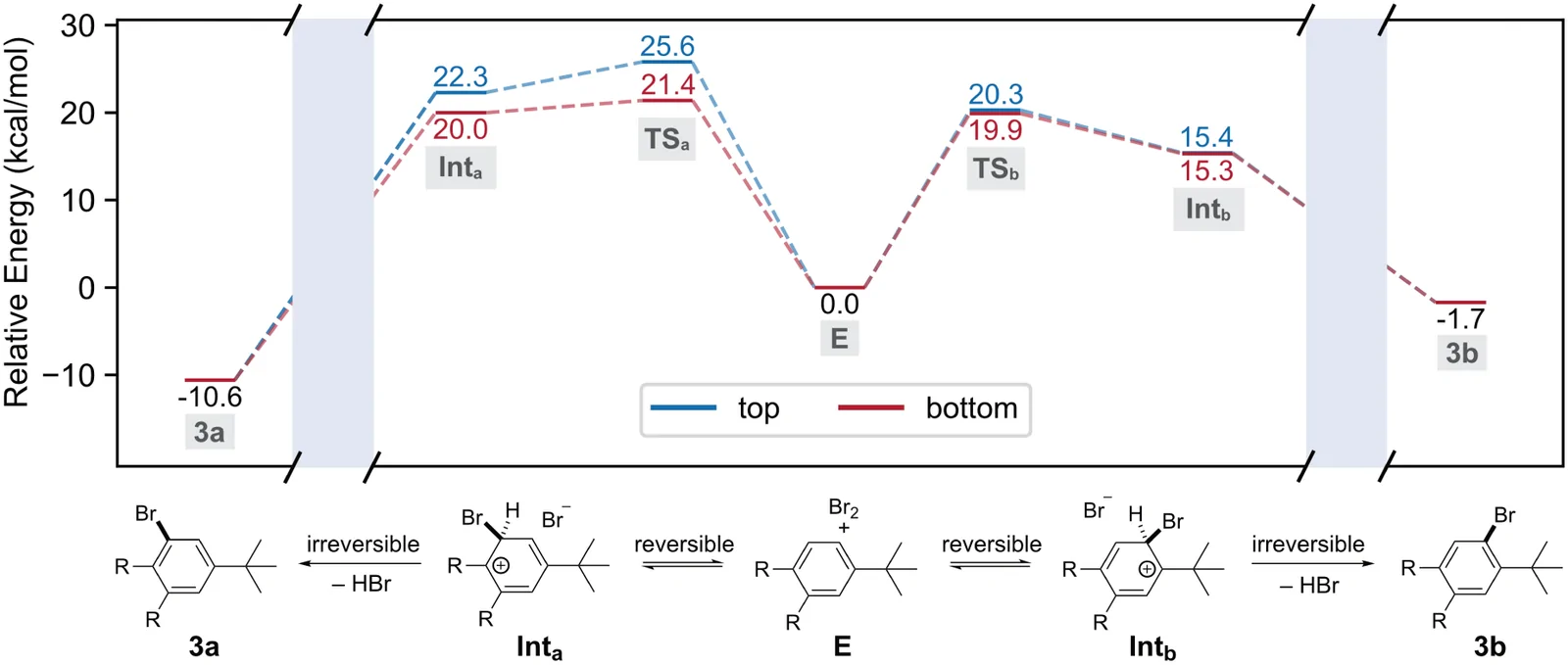
Top: Energy diagram for the bromination of compound **2** to form the respective Wheland intermediates in positions 15 (right) and 16 (left) with the approach of bromine from the top (blue) or bottom (red), as well as energies of the products **3a** and **3b**. Bottom: Schematic illustration of the respective bromination sides and indication of reversible and irreversible reaction steps.

The activation barriers for the formation of all Wheland intermediates range from 19.9 to 25.6 kcal/mol, suggesting that the reaction could occur at room temperature without a Lewis acid catalyst. Formation of a Wheland intermediate at the 16-position is favored, with energies of 20.3 and 19.9 kcal/mol for **TS**_**a**_^**1**^ and **TS**_**a**_^**2**^, respectively. Here, **TS**_**a**_^**1**^ and **TS**_**a**_^**2**^ indicate the attack of bromine from the top or bottom, respectively. This is consistent with the experimental results, in which product **3b** forms in a higher ratio than **3a**, as the corresponding transition states, **TS**_**b**_^**1**^ and **TS**_**b**_^**2**^, have slightly higher energies. Since the elimination of HBr to yield the products is irreversible, the selectivity is determined only by the relative energies of the transition states. Even though the transition-state energies of 21.7 and 22.5 kcal/mol for the bay-substituted product (position 14) fall within the same range as the others (Figure S7), no product formation was observed.

The formation of the respective intermediates appears to be energetically possible, but it is known that the inversion of the (*P*)- and (*M*)-enantiomers occurs dynamically. Assuming the validity of transition-state theory, the relative rates of the two unimolecular processes are proportional to an exponential function of the difference in the activation free energies divided by *RT* (Supporting Information, Chapter 3.6, eq 2). Consequently, even a moderate difference of ∼5–6 kcal/mol in activation free energies corresponds to a rate ratio of around 10^4^. In the present case, the helical inversion barrier of 11 kcal/mol is therefore expected to be several orders of magnitude lower than the barrier for the formation of the reaction intermediate. This means that the interconversion of enantiomers occurs thousands of times before the intermediate can form. We therefore propose that the higher-energy transition state is effectively not reached when a significantly lower-energy competing transition state is available. The interconversion of enantiomers implies that a bromine molecule cannot form a stable intermediate, since the interconversion of helicity would cause the intermediate to break down. To support this hypothesis, we calculated the inversion barrier of a theoretically stable intermediate in the bay region to be ∼7 kcal/mol (Figure S6) showing that in a theoretically formed intermediate **Int**_c_, fast helical inversion would occur. Optimization of the Wheland intermediate structure at the highest energy point of helical inversion necessarily leads to disintegration of the intermediate.

The photophysical properties of compounds **4a** and **4b** were analyzed in three solvents with different polarity and proticity (EtOH (polar/protic), DCM (medium polar/aprotic), and toluene (apolar/aprotic)). The absolutely determined photoluminescence quantum yields Φ_*fl*_ and the fluorescence lifetimes *τ_fl_* of the nitriles **4a** and **4b** are displayed in Table 1. The functionalized HBC derivatives **4a** and **4b** reach about 10% higher Φ_*fl*_ than those of the unsubstituted HBC **2** with a maximum Φ_*fl*_ of 73% and 76% in toluene for **4a** and **4b**, respectively. No changes of the photoluminescence lifetimes *τ_fl_* were observed for compounds **4a** and **4b** compared to **2**.

**Table 1:** Absolutely Determined Photoluminescence Quantum Yields Φ_*fl*_ and Fluorescence Lifetimes *τ_fl_* of Compounds **2**,^22^ **4a**, and **4b** in Solvents of Different Polarity^a^

	**2**		**4a**		**4b**	
Solvent	Φ_*fl*_ (%)	*τ_fl_* (ns)	Φ_*fl*_ (%)	*τ_fl_* (ns)	Φ_*fl*_ (%)	*τ_fl_* (ns)
EtOH	61	9.2	70	8.0	76	7.6
DCM	51	5.6	61	6.5	64	6.1
Toluene	67	7.3	73	6.7	76	6.3

a All measurements were performed in argon-saturated solutions.

The photoluminescence and absorption spectra are shown in Figure 4 exemplarily for toluene. Complete absorption, photoluminescence, and photoluminescence lifetime spectra are available in Figures S14–S17. Compounds **4a** and **4b** exhibit multiple electronic transitions in the absorption spectrum in the area between 300 and 550 nm, which have been assigned using time-dependent density functional theory (TD-DFT) (Tables S3–S7). We note that the ωB97X and CAM-B3LYP functionals overestimate the transition energies, while the use of B3LYP in TD-DFT leads to a cancellation of the errors and close match with the experimental absorption spectra. The molar absorption coefficients reach maximum values of about 50,000 and 59,000 L·mol^–1^·cm^–1^ in DCM and toluene for **4a** and **4b**, respectively. Both compounds show three emission maxima in the region of 500 to 650 nm. At an excitation wavelength of 379 nm, the emission maxima are at 520 nm, 560 nm, and 602 nm for **4a** and at 517 nm, 555 nm, and 598 nm for **4b**.

**Figure 4:**
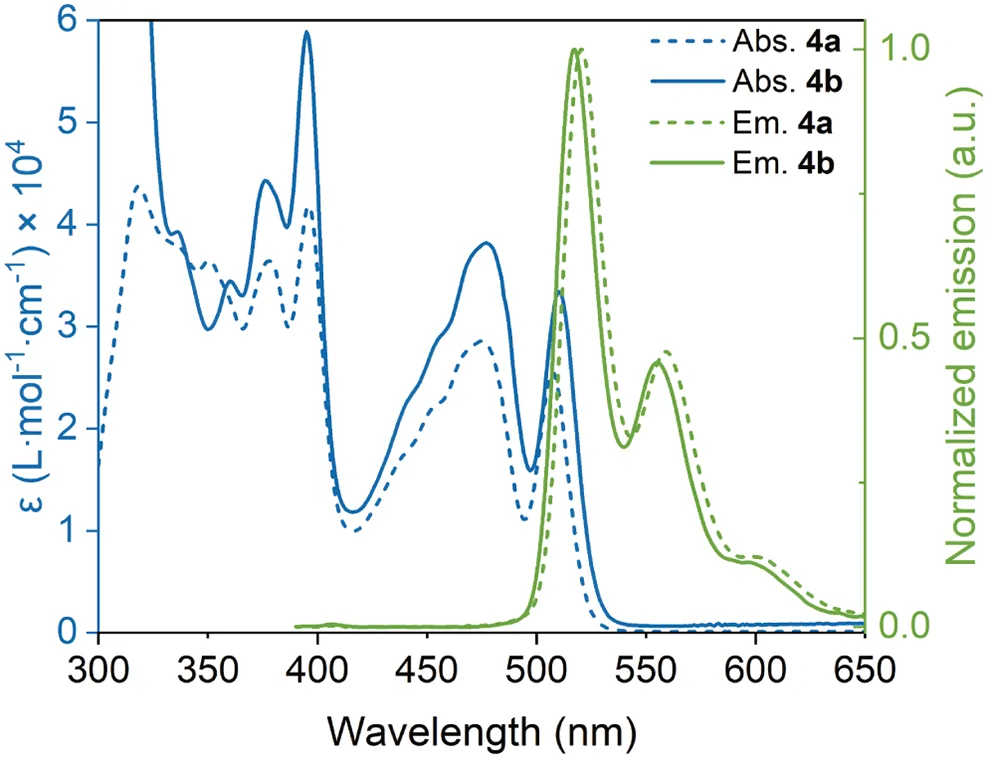
Absorption spectra (blue) and fluorescence spectra (green, normalized) of compounds **4a** (solid) and **4b** (dashed) in toluene. Absorption was measured at a concentration of 1·10^–5^ mol·L^–1^; emission at a concentration of 1·10^–6^ mol·L^–1^ with excitation at λ_exc_ = 379 nm.

In comparison to the unsubstituted HBC **2**, emission and absorption are shifted bathochromically by approximately 20 nm. Tables S10 and S11 provide an overview of the absorption and emission maxima. The Stokes shifts for both compounds **4a** and **4b** range from 1,622 to 2,399 cm^–1^.

We have shown earlier that compound **2** is well-suited for the fabrication of OLEDs without tedious optimization processes.^22^ To compare the performances, we hence fabricated first-generation OLEDs from compounds **4a** and **4b** to investigate the effect of nitrile substitution. Optimal thin films of 50–60 nm thickness were obtained by spin-coating dye solutions in chloroform. The HOMO (highest occupied molecular orbital) and LUMO (lowest unoccupied molecular orbital) energies were determined by photoelectron yield spectroscopy and UV/vis spectroscopy of the spin-coated films to be 5.6/3.4 eV and 5.5/–3.2 eV for **4a** and **4b**, respectively. As observed by the similar color, the nitrile substitution leads to an absolute shift in the frontier orbital energy, while the HOMO–LUMO gap for **4a** and **4b** decreases marginally compared to the unsubstituted compound **2** (2.2/2.3 eV vs 2.4 eV, Figure S44). An overview of selected fabricated devices is provided in Table S12 and Figures S18–S41; a complete list of experiments is provided within the raw data. Similar to **2**, devices with pure **4a** or **4b** as an emitter are dominated by yellow excimer emission with an average luminance of 1,390 and 2,280 cd·m^–2^ over five devices and CIE coordinates of 0.47/0.52 and 0.48/0.51, respectively (Figure 5A,C,D). To suppress excimer emission, **4a** or **4b** was spin-coated together with the host material tris­(4-carbazoyl-9-ylphenyl)­amine (TCTA) to fabricate OLEDs of the structure ITO/PEDOT:PSS/(**4a** or **4b**) in TCTA/Ca/Al (Figure 5B,C,D; Figures S42 and S43), shifting to a greener color with CIE coordinates of 0.32/0.62 and 0.35/0.60, respectively. The highest average luminances of 3,060 and 3,390 cd·m^–2^, respectively, were measured at a concentration of 1 wt % emitter in TCTA, accompanied by a 6-fold increase of the current efficacy. Both types of OLEDs show a maximum current efficacy at 6 V but roll off at higher voltages (Figures S39 and S40). We further optimized the device structure by the introduction of a hole-transport layer and electron-blocking layer using poly­(9,9-dioctylfluorene-*alt*-*N*-(4-*sec*-butylphenyl)-diphenylamine) (TFB) in devices of the type ITO/PEDOT:PSS/TFB/0.5 wt % (**4a** or **4b**) in TCTA/Ca/Al, which leads to a 200% increase of the average luminance to 6,520 and 6,310 cd·m^–2^, respectively. This contrasts with compound **2**, where the additional hole-transport and electron-blocking layer had no significant effect on the device performance. The former device exhibits a turn-on voltage of 5 V with only a small roll-off above 10 V (Figure S41) while having similar current efficacies of 1.31 and 1.10 cd·A^–1^ compared to devices with emitter **2**.

**Figure 5:**
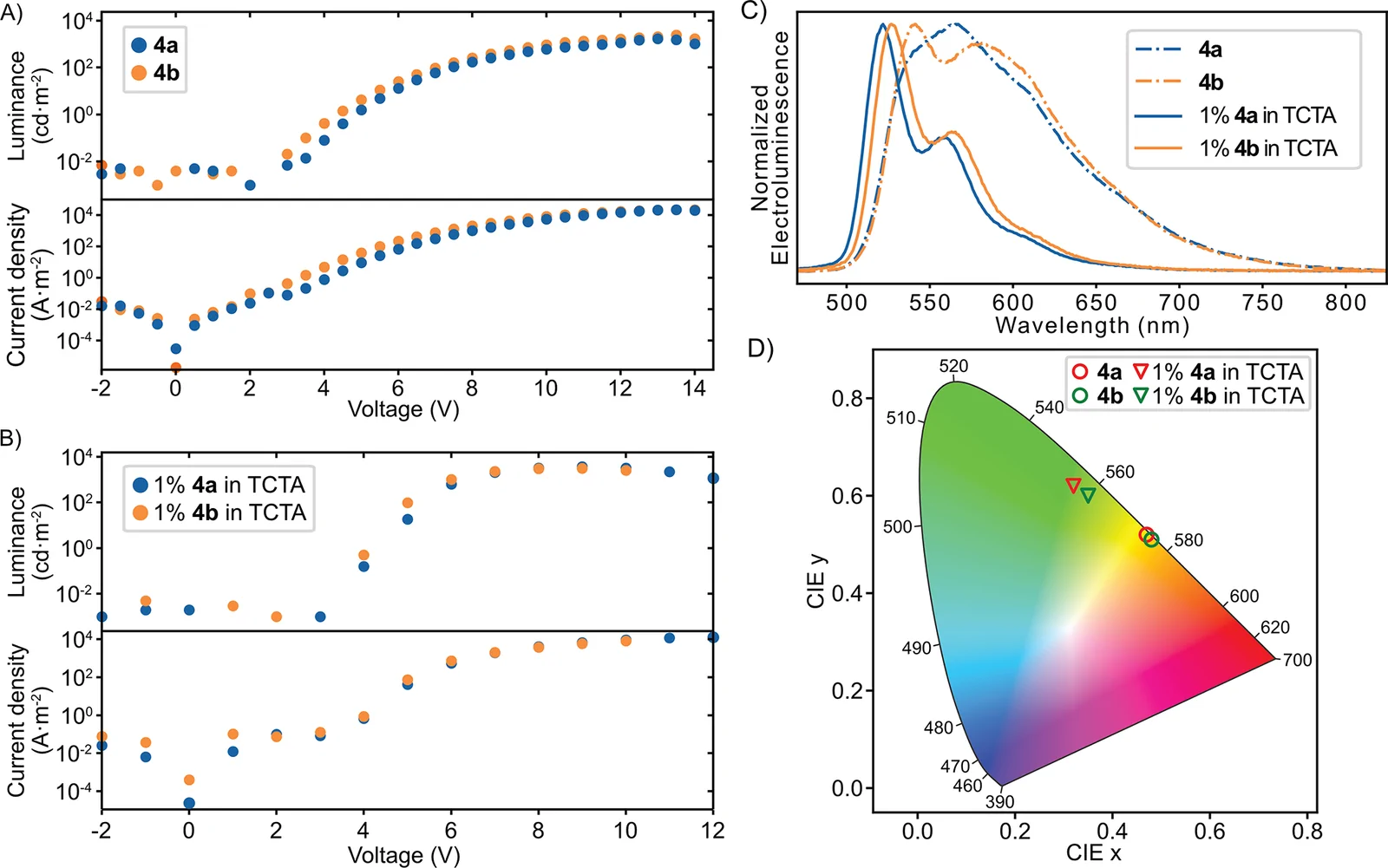
A) JVL characteristics for OLED devices with pure **4a** or **4b** as the emitter. B) JVL characteristics for OLED devices with 1% **4a** or **4b** in a TCTA host. C) Electroluminescence spectra in OLED devices for compounds **4a** and **4b** in pure form (dashed-dotted) or 1% in a TCTA host (solid). D) CIE 1931 chromaticity diagram with colors of OLED devices with pure **4a** and **4b** as the emitter (circle) or 1% in a TCTA host (triangle).

## Conclusions

In conclusion, we report the regioselective bromination of a symmetry-broken hexa-*peri*-benzocoronene–fluoranthene hybrid, which exclusively occurs at two vicinal peripheral positions rather than at the K-type site predicted by electronic calculations and structural characteristics. Computational studies revealed similar energy barriers for the formation of possible intermediates. We propose that based on the fast helical inversion, the formation of Wheland intermediates in the K-type region is prevented. Instead, the reaction occurs on accessible sites, which were assumed to be less favorable, e.g., in terms of bond lengths or electron density. The resulting brominated products were efficiently converted into the corresponding nitriles, **4a** and **4b**, which exhibit photoluminescence quantum yields of up to 73% and 76%, respectively, in toluene. First-generation OLED emitters were prepared and outperform the parent compound **2** in direct comparison, as an effective hole-transport and electron-blocking layer (TFB) can be introduced, leading to an increase in luminance up to 6,500 cd·m^–2^. This study demonstrates that structural asymmetry and helicity in polycyclic aromatic hydrocarbons can lead to unexpected reactivity and proposes that dynamic processes should be taken into account for rationalization.

## Supplementary Material



## Notes

The Supporting Information is available free of charge at https://pubs.acs.org/doi/10.1021/acsphyschemau.6c00115. Raw data are provided in the Refubium repository under https://dx.doi.org/10.17169/refubium-50619.

General information on experimental procedures, computational details, X-ray crystallographic data, photophysical characterization, OLED performance measures, and spectroscopic data (PDF)

## Abbreviations

AICD: anisotropy of the induced current density
CISS: chirality-induced spin selectivity
CPL: circularly polarized luminescence
EDDB_π_: electron density of delocalized π-bonds
EDLB_π_: electron density of localized π-bonds
HBC: hexa-*peri*-benzocoronene
HOMO: highest occupied molecular orbital
ITO: indium tin oxide
LUMO: lowest unoccupied molecular orbital
NICS: nucleus-independent chemical shift
OFET: organic field-effect transistor
OLED: organic light-emitting diode
PAH: polycyclic aromatic hydrocarbon
PYS: photoelectron yield spectroscopy
TCTA: tris­(4-carbazoyl-9-ylphenyl)­amine
(TD)-DFT: (time-dependent) density functional theory
TFB: poly­(9,9-dioctylfluorene-*alt*-*N*-(4-*sec*-butylphenyl)-diphenylamine)
