# Supplementary material for: Late-Stage Functionalization of a Hexa-peri-Benzocoronene–Fluoranthene Hybrid Influenced by Dynamic Inversion of Helicity
Source: ACS Phys Chem Au. 2026 Aug 13;6(5):1363–71. doi: 10.1021/acsphyschemau.6c00115 (PMC13614034; doi:10.1021/acsphyschemau.6c00115)
Supplement: Supplementary file 1 [file pg6c00115_si_001.pdf]

# Supporting Information

## Late-Stage Functionalization of Hexa-*peri*-Benzocoronene-Fluoranthene Hybrid Influenced by Dynamic Inversion of Helicity

Lina M. Filla,[a] Alexander R. Krappe,[a] Jacob C. Mayer,[b] Jan M. Sühlo,[c] Larissa S. Eitelhuber,[d] Anja Wiesner,[e] Lukasz Polewski,[a] Ute Resch-Genger,[f] Beate Paulus,[d] Emil J. W. List-Kratochvil,[b,g] and Siegfried Eigler*[a]

[a] Institut für Chemie und Biochemie (SupraFAB), Freie Universität Berlin, Altensteinstraße 23a, 14195 Berlin, Germany; E-mail: siegfried.eigler@fu-berlin.de
[b] Helmholtz-Zentrum Berlin für Materialien und Energie GmbH, Hahn-Meitner-Platz 1, 14109 Berlin, Germany
[c] Institut für Physik, Institut für Chemie, Humboldt-Universität zu Berlin, Zum Großen Windkanal 2, 12489 Berlin, Germany
[d] Institut für Chemie und Biochemie, Freie Universität Berlin, Arnimallee 22, 14195 Berlin, Germany
[e] Institut für Chemie und Biochemie, Freie Universität Berlin, Fabeckstraße 34/36, 14195 Berlin, Germany
[f] Bundesanstalt für Materialforschung und -prüfung (BAM), Department 1, Division Biophotonics, Richard-Willstätter-Straße 11, 12489 Berlin, Germany
[g] Institut für Physik, Institut für Chemie, Center for the Science of Materials Berlin, Humboldt-Universität zu Berlin, Zum Großen Windkanal 2, 12489 Berlin, Germany

## Table of Contents


1 General Information ....... 3

2 Synthetic Procedures ....... 5

3 Computational Details ....... 8

3.1 Population Analysis ....... 8

3.2 Electronically Excited States (TD-DFT) ....... 9

3.3 Bond Length Analysis ....... 15

3.4 Orbital Analysis ....... 16

3.5 Inversion Barrier ....... 17

3.6 Transition States and Intermediates ....... 18

4 NMR Discussion ....... 19

5 Reaction Conditions ....... 20

6 Crystallographic Data ....... 21

7 UV/vis, Fluorescence and Lifetime Measurements ....... 25

8 Organic Light-Emitting Diode (OLED) ....... 28

8.1 Device Fabrication ....... 28

8.2 Device Performance ....... 29

9 References ....... 44

10 Spectra Appendix ....... 46

11 Optimized Geometries ....... 57

# 1 General Information

All reagents were purchased from commercial sources and were used without further purification unless otherwise stated. All air and moisture sensitive reactions were performed under standard Schlenk technique using nitrogen as inert gas. Dry DCM was obtained from MBraun Solvent Purification System MB-SPS 800 filled with $Al_2O_3$. Dry DMF over molecular sieves and bromine were purchased from Thermo Fisher Scientific. Copper cyanide was purchased from Acros Organics.

**Thin-layer chromatography** was performed on ALUGRAM® Xtra SIL G/$UV_{254}$ plates from Macherey-Nagel. Indication was performed by irradiation with UV light of wavelengths $\lambda$ = 254 and 365 nm. Column chromatography was performed with silica gel Silica 60 M (0.04-0.063 mm) from Macherey-Nagel.

**NMR spectra** were recorded on a Bruker AVANCE 500 ($^1$H 500 MHz, $^{13}$C 126 MHz), a JEOL ECZ 600 ($^1$H 600 MHz, $^{13}$C 151 MHz), and a Bruker AVANCE 700 ($^1$H 700 MHz, $^{13}$C 176 MHz) spectrometer at 25 °C. The chemical shifts *δ* are calibrated on the respective residual solvent signal as internal standard. All shifts are reported in ppm and NMR multiplicities are abbreviated as s (singlet), d (doublet), t (triplet), q (quartet), m (multiplet). Coupling constants *J* are reported in Hz. NMR spectra were processed using the software MestReNova (version 14.3.0) from Mestrelab Research.

**UV/vis spectra of solutions** were recorded using an Agilent Technologies Cary 8454 UV/vis spectrometer. **UV/vis spectra of films** were recorded on a Lambda 950 UV/vis spectrometer from PerkinElmer. Thin films were spin coated onto cleaned quartz glass substrates according to the device fabrication procedure. The absorption was calculated from the transmission. **Photoluminescence spectra of solutions** were recorded on a FL 6500 Fluorescence Spectrometer from PerkinElmer. UV/vis and photoluminescence spectra were measured in quartz glass cuvettes from Hellma GmbH with 1 cm path length. The **photoluminescence spectra of thin films** were obtained on a FLS980 spectrometer from Edinburgh Instruments. The excitation wavelength was set to 470 nm.

**Photoelectron yield spectroscopy** (PYS) measurements were prepared by spin-coating according to the device fabrication procedure (Chapter 9.1) onto cleaned, ITO covered glass substrate. The measurement was performed on a RIKEN KEIKI instrument, model AC/2E DC1 in air.

**IR spectra** were recorded on a FT-IR Spectrum Two with $LiTaO_3$ detector from Perkin Elmer.

**HR-EI mass spectra** were measured on a Waters Autospec Premier mass spectrometer by the analytical department.

**Photoluminescence quantum yields** (Φ) were determined absolutely with an integrating sphere setup from Hamamatsu (Quantaurus-QY C11347-11). All Φ measurements were performed at 25 °C using special 10 mm x 10 mm long neck quartz cuvettes from Hamamatsu. The **fluorescence lifetime** ($\tau$) was recorded on a fluorometer FLS 920 (Edinburgh Instruments) equipped with a Hamamatsu R3809U-50 (range 200–850 nm, response width <25 ps), multichannel plate (MCP) detector, Czerny-Turner double monochromators and an Edinburgh Instruments EPL-375 (picosecond-pulsed light-emitting diode) for excitation at 375 nm. All the measurements were performed at 25 °C using 10 mm x 10 mm quartz cuvettes from Hellma GmbH. Before each measurement, the instrument response function (IRF) was measured. The lifetime measurements were analyzed with Edinburgh Instruments FAST

Software and fitted with a reconvolution fit. All the lifetimes could be evaluated mono-, bi- or tri-exponentially with a reduced $X^2$ between 1.06 and 1.14.

**Single crystal X-ray diffraction** data were collected from a shock-cooled single crystal at 101(2) K on a Bruker D8 VENTURE dual wavelength Mo/Cu three-circle diffractometer with a microfocus sealed X-ray tube using a mirror optics as monochromator and a Bruker PHOTON II detector. A brown, plate-shaped crystal was mounted on a MiTeGen micromount with perfluoroether oil. The diffractometer was equipped with an Oxford Cryostream 700 low temperature device and used Cu$K_\alpha$ radiation (λ = 1.54178 Å). All data were integrated with SAINT V8.40B and a multi-scan absorption correction using SADABS 2016/2 was applied.[1, 2] The structure was solved by direct methods with SHELXT and refined by full-matrix least-squares methods against $F^2$ using SHELXL-2018/3.[3, 4] All non-hydrogen atoms were refined with anisotropic displacement parameters. All hydrogen atoms were refined isotropic on calculated positions using a riding model with their $U_{iso}$ values constrained to 1.5 times the $U_{eq}$ of their pivot atoms for terminal sp$^3$ carbon atoms and 1.2 times for all other carbon atoms. Disordered moieties were refined using bond lengths restraints and displacement parameter restraints. ORTEP illustrations were generated using PLATON.[5] Publication figures were generated with the program Diamond (Version 4.1) by Crystal Impact.

## 2 Synthetic Procedures

15-Bromo-2,5,8,11,17,19-hexa-*tert*-butyldibenzo[*fg*,*ij*]indeno[1',2',3':8,9]pyreno-[5,4,3,2,1-*pqrst*]pentaphene (**3a**) and 16-bromo-2,5,8,11,17,19-hexa-*tert*-butyl-dibenzo[*fg*,*ij*]indeno[1',2',3':8,9]pyreno[5,4,3,2,1-*pqrst*]pentaphene (**3b**)

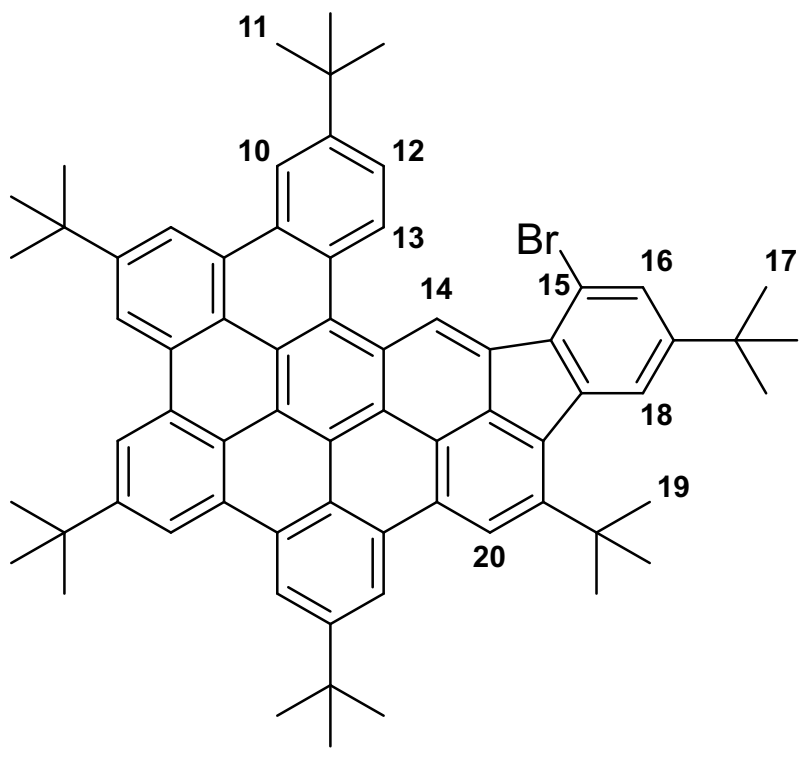

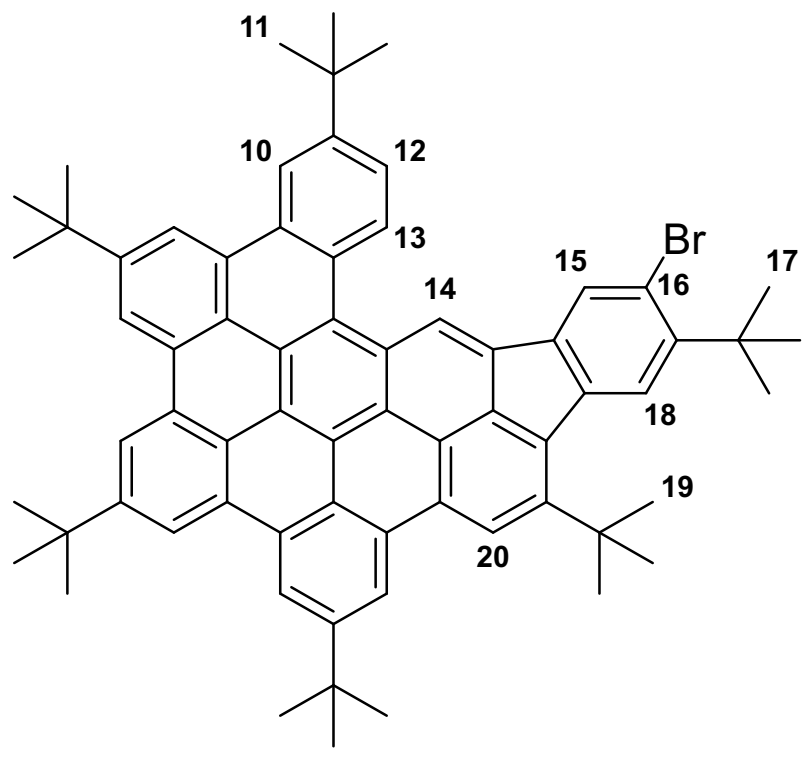

**3a** **3b**

Compound **2** (0.107 g, 121 µmol, 1.0 equiv) was dissolved in DCM (10 mL) and cooled to 0 °C. A solution of bromine (0.141 g, 882 µmol, 7.3 equiv) in DCM (2.5 mL) was added dropwise, and the mixture was stirred for 4.5 h at 0 °C. The reaction was quenched with $NaHSO_3$ (10 mL). The phases were separated, and the organic phase was washed with $NaHSO_3$ (10 mL) and water (1x 10 mL). The aqueous phase was extracted with DCM (2x 10 mL). The combined organic phases were dried over $MgSO_4$, and the solvent was removed under reduced pressure. The products **3a** and **3b** were yielded as a mixture (0.114 g, 118 µmol, 94%). The crude product was used without further purification.

**3a:**

**$^1$H NMR** (500 MHz, $CDCl_3$, rt): *δ* (ppm) = 10.84 (s, 1H, **14**), 9.32 (s, 1H, **20**), 9.30–9.29 (m, 2H), 9.19 (d, $^4J$ = 1.7 Hz, 1H), 9.18–9.17 (m, 4H), 9.14 (d, $^4J$ = 1.5 Hz, 1H, **10**), 8.62 (d, $^4J$ = 1.5 Hz, 1H, **18**), 7.92 (dd, $^3J$ = 8.6 Hz, $^4J$ = 2.0 Hz, 1H, **12**), 7.72 (d, $^4J$ = 1.4 Hz, 1H, **16**), 2.05 (s, 9H, **19**), 1.83 (s, 9H, ***t*-Bu**), 1.82 (s, 9H, ***t*-Bu**), 1.79 (s, 9H, ***t*-Bu**), 1.68 (s, 9H, **11**), 1.56 (s, 9H, **1**).

**3b**:

**$^1$H NMR** (500 MHz, $CDCl_3$, rt): *δ* (ppm) = 9.90 (s, 1H, **14**), 9.32 (s, 1H, **20**), 9.31–9.29 (m, 4H), 9.17 (d, $^4J$ = 1.7 Hz, 1H), 9.13 (d, $^4J$ = 1.7 Hz, 1H, **10**), 9.07 (d, $^3J$ = 8.5 Hz, 1H), 8.99 (d, $^4J$ = 2.0 Hz, 1H), 8.66 (s, 1H, **18**), 8.58 (s, 1H, 1**5**), 7.98 (dd, $^3J$ = 8.6 Hz, $^4J$ = 2.0 Hz, 1H, **12**), 2.04 (s, 9H, **19**), 1.83 (s, 9H, ***t*-Bu**), 1.81 (s, 9H, ***t*-Bu**), 1.79 (s, 9H, ***t*-Bu**),1.76 (s, 9H, **11**), 1.70 (s, 9H, **1**).

**3a** and **3b**:

**$^{13}$C NMR** (176 MHz, $CDCl_3$, rt): *δ* (ppm) = 151.7, 151.4, 151.2, 150.3, 150.1, 149.5, 149.5, 149.4, 149.4, 149.4, 149.3, 149.3, 147.6, 147.6, 146.3, 146.1, 143.1, 141.6, 141.4, 139.9, 139.5, 137.9, 135.4, 135.4, 134.9, 133.2, 132.9, 132.9, 132.8, 132.3, 132.1, 131.9, 131.8, 131.7, 131.7, 131.6, 131.1, 131.0, 130.9, 130.9, 130.8, 130.8, 130.6, 130.5, 130.4, 130.2, 130.2, 130.1, 130.0, 128.8, 128.4, 128.2, 127.9, 127.7, 127.3, 126.9, 126.8, 126.4, 126.1, 125.9, 125.9, 125.9, 125.4, 125.0, 124.8, 124.7, 124.7, 124.5, 124.3, 124.3, 124.0, 123.9, 123.9, 123.8, 123.7, 123.6, 123.6, 123.5, 122.6, 122.4, 122.3, 122.1, 122.0, 121.9, 121.8, 121.7, 121.3, 121.3, 121.2, 120.7, 120.6, 120.1, 120.0, 119.6, 119.5, 119.5, 119.4, 119.3, 119.3,

119.1, 119.1, 118.9, 118.9, 118.6, 118.5, 37.7, 36.6, 36.5, 35.9, 35.9, 35.9, 35.8, 35.8, 35.7, 35.5, 35.4, 32.3, 32.2, 32.2, 32.2, 32.1, 31.9, 31.8, 31.8, 31.4, 31.3, 30.4.

**FT-IR (ATR):** $\tilde{\nu}$ (cm$^{-1}$) = 2954 (s), 2868 (w), 1607 (m), 1479 (m), 1464 (m), 1444 (m), 1394 (m), 1364 (s), 1311 (w), 1258 (m), 1203 (w), 1117 (w), 1008 (w), 970 (w), 938 (w), 899 (w), 870 (vs), 846 (w), 831 (w), 775 (w), 745 (w), 713 (w), 674 (w), 634 (w), 624 (m), 604 (w), 462 (vw).

**MS** (EI): m/z = calc.: 962.4421 [M-$^{79}$Br]$^{\cdot+}$, meas.: 962.4510 [M-$^{79}$Br]$^{\cdot+}$.

## 2,5,8,11,17,19-Hexa-*tert*-butyldibenzo[*fg*,*ij*]indeno[1',2',3':8,9]pyreno[5,4,3,2,1-*pqrst*]pentaphene-15-carbonitrile (**4a**) and 2,5,8,11,17,19-Hexa-*tert*-butyldibenzo[*fg*,*ij*]-indeno[1',2',3':8,9]pyreno[5,4,3,2,1-*pqrst*]pentaphene-16-carbonitrile (**4b**)

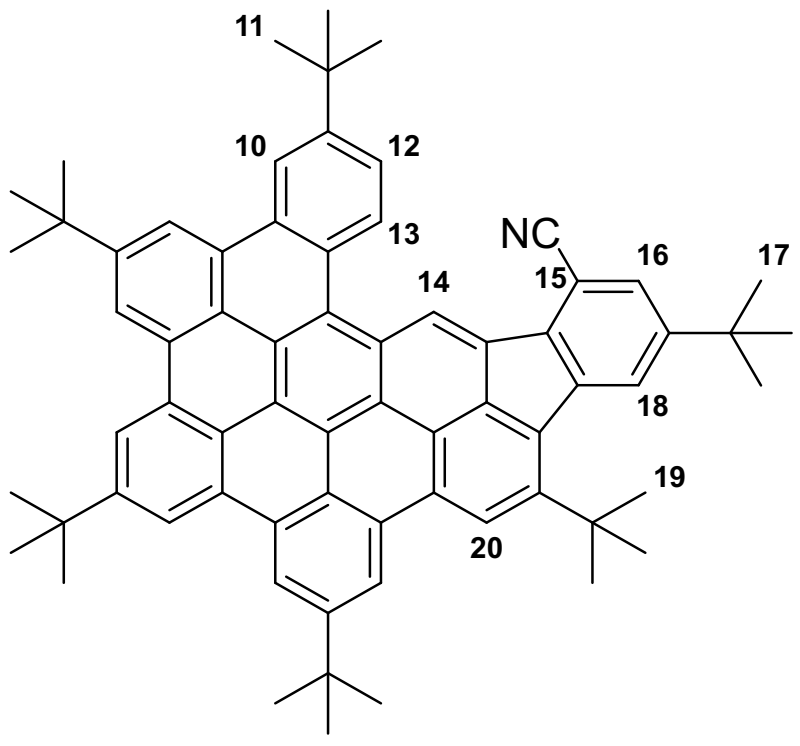


**4a**

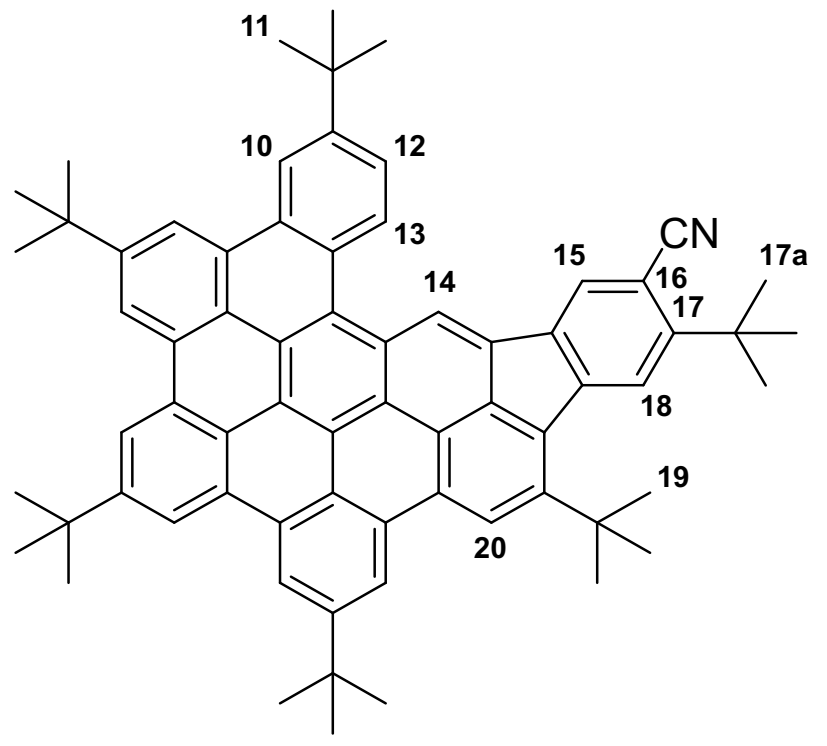


**4b**

Under nitrogen atmosphere, the mixture of compounds **3a** and **3b** (53.7 mg, 55.7 µmol, 1.0 equiv) and CuCN (20 mg, 223 µmol, 4.0 equiv) were dissolved in DMF (7.0 mL). The mixture was stirred for 3 d at 153 °C and subsequently quenched with brine (10 mL). The aqueous phase was extracted with EtOAc (2x 20 mL) and DCM (2x 20 mL). The combined organic layers were washed with brine (20 mL) and water (2x 20 mL), and dried over $MgSO_4$. The solvent was removed under reduced pressure and the crude product was purified by column chromatography (cyclohexane/DCM 3:1; $R_f$ = 0.46 (**4a**); $R_f$ = 0.36 (**4b**)), yielding **4a** (16.8 mg, 18.5 µmol, 36%) and **4b** (18.8 mg, 20.7 µmol, 45%) as orange solids in a total yield of 81%.

**4a**:

**$^{1}$H NMR** (700 MHz, $CDCl_3$, rt): $\delta$ (ppm) = 10.70 (s, 1H, **14**), 9.34–9.33 (m, 1H), 9.32 (m, 2H, **20**), 9.30 (m, 1H), 9.27 (d, $^{3}J$ = 8.4 Hz, 1H, **13**), 9.20 (m, 1H), 9.18 (m, 1H), 9.14 (d, $^{4}J$ = 1.3 Hz, 1H), 8.99 (d, $^{4}J$ = 2.0 Hz, 1H, **13**), 8.85 (d, $^{4}J$ = 1.6 Hz, 1H, **18**), 8.02 (dd, $J$ = 8.4, 2.0 Hz, **12**), 7.79 (d, $^{4}J$ = 1.5 Hz, 1H, **16**), 2.04 (s, 9H, **19**), 1.83 (s, 9H, ***t*-Bu**), 1.82 (s, 9H, ***t*-Bu**), 1.79 (s, 9H, ***t*-Bu**), 1.68 (s, 9H, **11**), 1.57 (s, 9H, **1**).

**$^{13}$C NMR** (176 MHz, $CDCl_3$, rt): $\delta$ (ppm) = 150.7 (1C, **q11**), 150.6 (1C, **q1**), 149.9 (1C, **q**), 149.6 (1C, **q**), 149.5 (1C, **q**), 148.1 (1C, **q19**), 141.6 (1C, **q**), 139.0 (1C, **q**), 132.8, (1C, **q**) 132.3 (1C, **13**), 131.6 (1C, **q**), 131.2 (1C, **q**), 131.1 (1C, **q**), 131.0 (1C, **q**), 130.9 (1C, **q**), 130.4 (1C, **q**), 130.2 (1C, **q**), 129.8 (1C, **q**), 129.5 (1C, **q**), 129.3 (1C, **18**), 128.1 (1C, **q**), 127.4 (1C, **q**), 126.7 (1C, **16**), 125.7 (1C, **14**), 125.2 (1C, **12**), 124.7 (1C, **q**), 124.0 (1C, **q**), 123.7 (1C, **q**), 123.5 (1C, **q**), 122.7 (1C, **q**), 122.6 (1C, **q**), 122.2 (1C, **q**), 121.4 (1C, **q**), 120.6 (1C, **q**), 119.9 (1C, **10**), 119.7 (1C), 119.7 (1C), 119.6 (1C), 119.6 (1C), 119.5 (1C), 119.2 (1C), 119.0 (1C), 118.8 (1C), 105.6 (1C, **NC**), 36.6 (1C, **q*t*-Bu19**), 36.0 (1C, **q**), 36.0 (1C, **q**), 35.9 (1C, **q**), 35.8 (1C, **q*t*-Bu1**), 35.5 (1C, **q*t*-Bu11**), 32.1 (6C, ***t*-Bu**), 32.1 (3C, ***t*-Bu**), 31.7 (3C, **11**), 31.6 (3C, **1**), 31.5 (3C, **19**).

**FT-IR (ATR):** $\tilde{\nu}$ (cm$^{-1}$) = 2955 (b), 2906 (s), 2868 (m), 2222 (w), 1750 (w), 1636 (w), 1607 (s), 1570 (w), 1479 (s), 1464 (s), 1426 (s), 1394 (s), 1364 (vs), 1311 (m), 1246 (vs), 1220 (m), 1203 (m), 1174 (m), 1157 (w), 1137 (w), 1086 (w), 1026 (w).

**UV/vis** (EtOH) $\lambda_{max}$ (nm): 335, 359, 373, 392, 473, 507.

**UV/vis** (DCM) $\lambda_{max}$ (nm), (ε (L·mol$^{-1}$·cm$^{-1}$)): 336 (45,000), 349 (43,000), 375 (45,000), 394 (50,000), 471 (32,000), 504 (24,000).

**UV/vis** (Toluene) $\lambda_{max}$ (nm), (ε (L·mol$^{-1}$·cm$^{-1}$)): 337 (38,000), 351 (36,500), 378 (36,500), 396 (42,000), 475 (28,500), 507 (25,500).

**MS** (EI): m/z = calc.: 909.5268 [M]$^{\cdot+}$, meas.: 909.5222 [M]$^{\cdot+}$.

**4b**:

**$^1$H NMR** (700 MHz, $CDCl_3$, rt): $\delta$ (ppm) = 10.02 (s, 1H, **14**), 9.35 (m, 1H), 9.34 (m, 2H, **20**), 9.30 (m, 1H), 9.20 (m, 1H), 9.18 (m, 1H), 9.14 (d, $^4J$ = 1.8 Hz), 9.07 (d, $^4J$ = 8.4 Hz, 1H, **13**), 9.01 (d, $^4J$ = 2.0 Hz, 1H, **10**), 8.71 (s, 1H, **18**), 8.69 (s, 1H, **15**), 8.00 (dd, J = 8.5, 2.0 Hz, 1H, **12**), 2.05 (s, 9H, **19**), 1.83 (s, 9H, ***t*-Bu**), 1.81 (s, 9H, ***t*-Bu**), 1.79 (s, 9H, ***t*-Bu**), 1.77 (s, 9H, **11**), 1.71 (s, 9H, **1**).

**$^{13}$C NMR** (176 MHz, $CDCl_3$, rt): $\delta$ (ppm) = 152.6 (1C, **q11**), 150.7 (1C, **q14**), 150.0 (1C, **q**), 149.8 (1C, **q**), 149.7 (1C, **q**), 149.1 (1C, **q19**), 144.3 (1C, **q**), 137.5 (1C, **q**), 133.5 (1C, **q**), 132.1 (1C, **q**), 131.9 (1C, **13**), 131.9 (1C, **q**), 131.7 (1C, **q**), 131.1 (1C, **q**), 131.0 (1C, **q**), 131.0 (1C, **q**), 130.3 (1C, **q**), 130.2 (1C, **q**), 129.8 (1C, **q**), 128.6 (1C, **15**), 128.2 (1C, **q**), 126.9 (1C, **q**), 125.6 (1C, **18**), 124.9 (1C, **12**), 124.7 (1C, **q**), 124.1 (1C, **q**), 123.7 (1C, **q**), 123.5 (1C, **q**), 122.9 (1C, **14**), 122.6 (1C, **q**), 122.4 (1C, **q**), 122.0 (1C, **q**), 121.7 (1C, **q**), 121.4 (1C, **quart.**), 120.7 (1C, **q**), 120.1 (1C, **10**), 119.8 (1C), 119.7 (1C), 119.5 (1C), 119.2 (1C), 119.2 (1C), 118.7 (1C), 107.2 (1C, **NC**), 36.7 (1C), 36.7 (1C), 36.0 (1C), 35.9 (1C), 35.9 (1C), 35.5 (1C, **q*t*-Bu1**), 32.1 (6C, ***t*-Bu**), 32.1 (3C, ***t*-Bu**), 31.8 (3C, **1**), 31.4 (3C, **q*t*-Bu19**), 30.9 (3C, **q*t*-Bu11**).

**FT-IR (ATR):** $\tilde{\nu}$ (cm$^{-1}$) = 2956 (b), 2925 (s), 2869 (m), 2218 (w), 1608 (s), 1569 (w), 1465 (s), 1394 (m), 1366 (s), 1310 (m), 1247 (s), 1204 (m), 1052 (w), 938 (w), 906 (w), 871 (vs), 846 (w), 831 (m), 815 (w), 775 (w), 746 (w), 705 (w), 689 (w), 677 (w), 627 (m), 604 (w), 545 (w).

**UV/vis** (EtOH) $\lambda_{max}$ (nm): 336, 348, 373, 392, 473, 507.

**UV/vis** (DCM) $\lambda_{max}$ (nm), (ε (L·mol$^{-1}$·cm$^{-1}$)): 336 (29,500), 360 (26,500), 375 (35,000), 394 (44,500), 475 (28,000), 508 (20,500).

**UV/vis** (Toluene) $\lambda_{max}$ (nm), (ε (L·mol$^{-1}$·cm$^{-1}$)): 336 (39,000), 360 (34,500), 376 (44,500), 395 (59,000), 477 (38,000), 510 (33,500).

**MS** (EI): m/z = calc.: 909.5268 [M]$^{\cdot+}$, meas.: 909.5242 [M]$^{\cdot+}$.

# 3 Computational Details

Quantum chemical calculations were performed using ORCA (versions 6.0.1 and 6.1.0) to investigate the reaction mechanism of the bromination.[6]

Structure optimizations were performed at the ωB97X-D3/def2-SVP level of density functional theory (DFT) using default convergence criteria.[7-9] Additional single point calculations on the optimized structures were performed at the ωB97X-D3BJ/def2-TZVP level of theory. The optimized structures were validated with frequency analysis. Solvent effects were considered by using the Conductor-like Polarizable Continuum Model (CPCM) with dichloromethane ($\varepsilon$ = 8.93) as solvent.[10]

Time-dependent DFT (TD-DFT) calculations were performed without TDA approximation, using several functionals for benchmarking, based on the optimized geometries.[11, 12]

The electron densities of delocalized and localized π-electrons, and Wiberg bond indices were calculated using the programs Gaussian 16[13] and NBO 7.0[14] according to the provided workflow.[15-17] Fukui functions[18] and dual descriptor were calculated using the programs Multiwfn[19, 20] and Gaussian 16.[13] The generated electron density surfaces were plotted with UCSF ChimeraX.[21]

## 3.1 Population Analysis

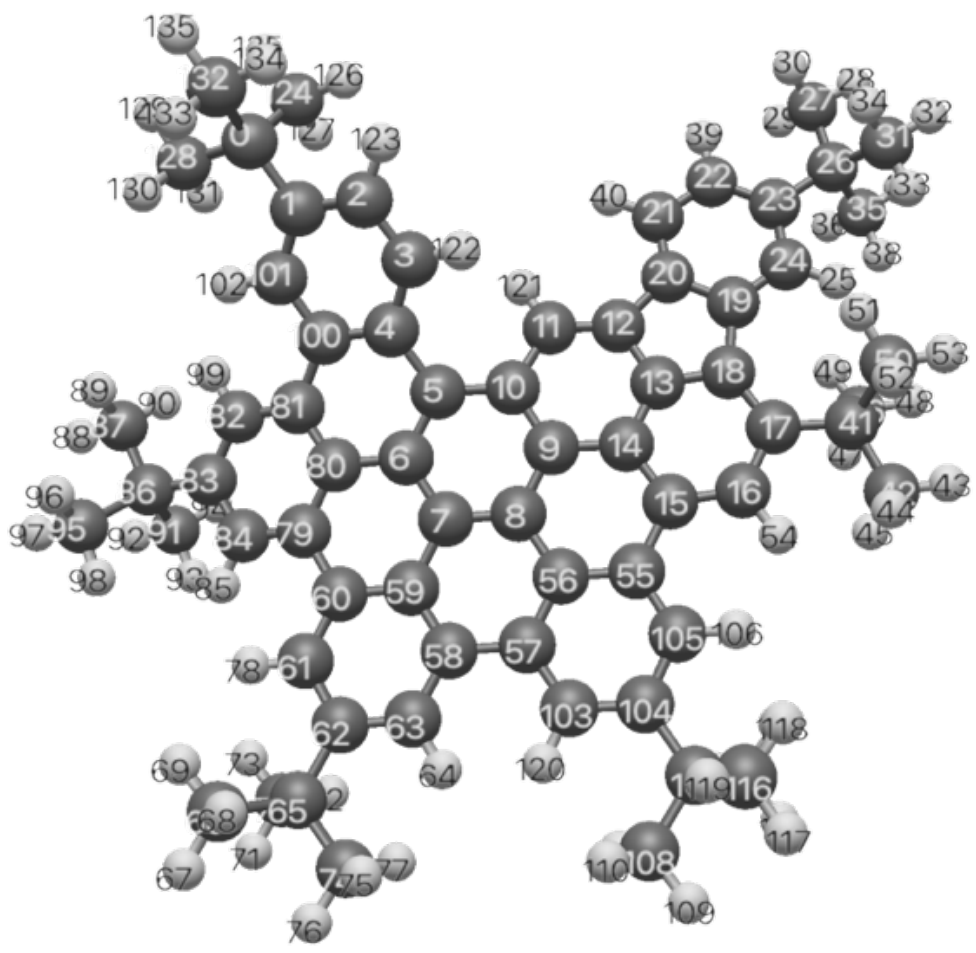

**Table S1.** Different population analyses of compound **2** calculated at the ωB97X-D3/def2-TZVP level of theory. Color scale individually for every column to visualize highest (green) and lowest (red) value. Lower values indicate more negative charge at the respective atom.

| No | Mulliken | Loewdin | NPA | HOMO Mulliken | HOMO Loewdin | Hirshfeld | MBIS | CHELPG |
|---|---|---|---|---|---|---|---|---|
| 2 | -0.242369 | -0.108627 | 0.01555 | 0.010675 | 0.011404 | -0.041943 | -0.198986 | -0.275724 |
| 3 | -0.386841 | -0.103255 | -0.21889 | 0.010141 | 0.011156 | -0.037564 | -0.191204 | -0.188297 |
| 4 | 0.069826 | -0.060623 | -0.18887 | 0.030109 | 0.031113 | -0.011923 | 0.016378 | 0.141888 |
| 5 | 0.232593 | -0.014802 | -0.05366 | 0.066764 | 0.059732 | -0.003488 | -0.015898 | -0.208084 |
| 10 | 0.199546 | -0.045146 | -0.02268 | 0.051136 | 0.048222 | -0.006342 | 0.008467 | 0.125216 |
| **11** | **-0.543624** | **-0.047128** | **-0.03923** | **0.042089** | **0.043703** | **-0.026203** | **-0.201625** | **-0.228586** |
| 12 | 0.044329 | -0.063484 | -0.15394 | 0.06005 | 0.053007 | -0.016798 | -0.001205 | -0.121441 |
| 20 | 0.012459 | -0.06705 | -0.0249 | 0.001889 | 0.006263 | -0.020235 | 0.005379 | 0.143329 |
| 21 | -0.299256 | -0.101736 | -0.05438 | 0.019634 | 0.016926 | -0.042607 | -0.168098 | -0.25209 |
| 22 | -0.169669 | -0.114122 | -0.18602 | 0.005371 | 0.007044 | -0.049888 | -0.225381 | -0.23372 |

## 3.2 Electronically Excited States (TD-DFT)

**Table S2.** Electronically excited singlet states of compound **4a** with major orbital contributions (>5%), energies and oscillator strength calculated at the B3LYP/def2-TZVP level of theory using TD-DFT. Highest occupied molecular orbital (HOMO) no. 243, lowest unoccupied molecular orbital (LUMO) no. 244.

| State | Orbital Contribution | Energy (eV) | Wavelength (nm) | $f_{osc}$ |
|---|---|---|---|---|
| 1 | 243 → 244 (0.929) | 2.56 | 483.7 | 0.46 |
| 2 | 242 → 244 (0.868)<br>243 → 245 (0.061) | 2.71 | 457.3 | 0.31 |
| 3 | 240 → 244 (0.115)<br>241 → 244 (0.812) | 3.04 | 407.5 | 0.02 |
| 4 | 242 → 244 (0.054)<br>243 → 245 (0.869) | 3.14 | 394.3 | 0.31 |
| 5 | 240 → 244 (0.746)<br>241 → 244 (0.083)<br>242 → 245 (0.056)<br>243 → 247 (0.081) | 3.27 | 378.6 | 0.02 |
| 6 | 242 → 245 (0.453)<br>243 → 246 (0.443) | 3.30 | 375.9 | 0.15 |
| 7 | 242 → 245 (0.371)<br>242 → 246 (0.050)<br>243 → 246 (0.456) | 3.37 | 367.5 | 0.46 |
| 8 | 241 → 245 (0.057)<br>242 → 245 (0.059)<br>242 → 246 (0.388)<br>243 → 246 (0.051)<br>243 → 247 (0.335) | 3.49 | 355.4 | 0.01 |
| 9 | 241 → 245 (0.127)<br>242 → 246 (0.480)<br>243 → 247 (0.260) | 3.60 | 344.8 | 0.16 |
| 10 | 238 → 244 (0.128)<br>241 → 245 (0.206)<br>242 → 247 (0.417)<br>243 → 247 (0.159) | 3.65 | 339.4 | 0.14 |
| 11 | 239 → 244 (0.744)<br>242 → 247 (0.087) | 3.75 | 330.5 | 0.12 |
| 12 | 238 → 244 (0.068)<br>239 → 244 (0.135)<br>240 → 245 (0.096)<br>241 → 245 (0.198)<br>241 → 246 (0.067)<br>242 → 247 (0.308) | 3.78 | 328.2 | 0.48 |
| 13 | 238 → 244 (0.486)<br>241 → 245 (0.222)<br>243 → 247 (0.071) | 3.84 | 323.2 | 0.18 |
| 14 | 237 → 244 (0.341)<br>239 → 244 (0.067)<br>240 → 245 (0.186)<br>242 → 247 (0.052)<br>243 → 249 (0.083) | 3.87 | 320.4 | 0.17 |
| 15 | 237 → 244 (0.278)<br>238 → 244 (0.068)<br>240 → 245 (0.102)<br>241 → 246 (0.070)<br>243 → 248 (0.376) | 3.93 | 315.2 | 0.17 |

**Table S3.** Electronically excited singlet states of compound **4a** with major orbital contributions (>5%), energies and oscillator strength calculated at the ωB97X/def2-TZVP level of theory. HOMO no. 243, LUMO no. 244.

| State | Orbital Contribution | Energy (eV) | Wavelength (nm) | $f_{osc}$ |
|---|---|---|---|---|
| 1 | 243 → 244 (0.826) | 3.23 | 383.3 | 0.79 |
| 2 | 242 → 244 (0.636)<br>243 → 245 (0.135) | 3.41 | 363.7 | 0.19 |
| 3 | 240 → 244 (0.397)<br>241 → 244 (0.278)<br>242 → 244 (0.073) | 3.83 | 324 | 0.23 |
| 4 | 240 → 244 (0.059)<br>242 → 244 (0.082)<br>242 → 245 (0.105)<br>243 → 245 (0.482)<br>243 → 246 (0.099) | 4.01 | 309.2 | 1.14 |
| 5 | 242 → 244 (0.056)<br>242 → 245 (0.463)<br>243 → 245 (0.054)<br>243 → 247 (0.103) | 4.16 | 297.7 | 0.76 |
| 6 | 237 → 244 (0.059)<br>240 → 244 (0.083)<br>241 → 244 (0.138)<br>243 → 247 (0.200) | 4.3 | 288.2 | 0.13 |
| 7 | 236 → 244 (0.077)<br>238 → 244 (0.086)<br>240 → 244 (0.052)<br>241 → 245 (0.107)<br>242 → 246 (0.053)<br>242 → 247 (0.181)<br>243 → 246 (0.072) | 4.38 | 282.9 | 0.17 |
| 8 | 240 → 245 (0.094)<br>240 → 246 (0.225)<br>241 → 245 (0.051)<br>241 → 246 (0.126)<br>242 → 247 (0.127) | 4.5 | 275.6 | 0.73 |
| 9 | 240 → 244 (0.084)<br>241 → 244 (0.173)<br>242 → 245 (0.086)<br>243 → 246 (0.121)<br>243 → 247 (0.190) | 4.57 | 271.2 | 0.64 |
| 10 | 240 → 246 (0.085)<br>241 → 245 (0.071)<br>243 → 246 (0.208)<br>243 → 247 (0.119)<br>243 → 249 (0.062) | 4.63 | 267.7 | 0.31 |
| 11 | 240 → 245 (0.096)<br>241 → 245 (0.094)<br>241 → 247 (0.054)<br>242 → 247 (0.190)<br>242 → 248 (0.053) | 4.65 | 266.6 | 0.22 |
| 12 | 237 → 244 (0.075)<br>242 → 246 (0.087)<br>242 → 249 (0.104)<br>242 → 250 (0.117) | 4.74 | 261.5 | 0.06 |
| 13 | 238 → 244 (0.132)<br>242 → 249 (0.084)<br>243 → 248 (0.093)<br>243 → 249 (0.175) | 4.83 | 256.7 | 0.21 |
| 14 | 237 → 244 (0.093)<br>239 → 244 (0.167)<br>239 → 245 (0.061)<br>241 → 245 (0.078)<br>242 → 249 (0.053)<br>242 → 250 (0.059) | 4.91 | 252.6 | 0.06 |
| 15 | 239 → 247 (0.075)<br>242 → 246 (0.063)<br>243 → 249 (0.104) | 4.96 | 249.9 | 0.07 |

**Table S4.** Electronically excited singlet states of compound **4a** with major orbital contributions (>5%), energies and oscillator strength calculated at the CAM-B3LYP/def2-TZVP level of theory. HOMO no. 243, LUMO no. 244.

| State | Orbital Contribution | Energy (eV) | Wavelength (nm) | $f_{osc}$ |
|---|---|---|---|---|
| 1 | 243 → 244 (0.892) | 3.01 | 411.8 | 0.73 |
| 2 | 242 → 244 (0.753)<br>243 → 245 (0.103) | 3.18 | 389.4 | 0.24 |
| 3 | 240 → 244 (0.373)<br>241 → 244 (0.375)<br>242 → 244 (0.063)<br>243 → 245 (0.069) | 3.57 | 347.2 | 0.19 |
| 4 | 240 → 244 (0.076)<br>242 → 244 (0.078)<br>243 → 245 (0.617)<br>243 → 246 (0.092) | 3.74 | 331.3 | 0.89 |
| 5 | 242 → 245 (0.606)<br>243 → 247 (0.092) | 3.87 | 320.0 | 0.46 |
| 6 | 240 → 244 (0.158)<br>241 → 244 (0.259)<br>243 → 245 (0.055)<br>243 → 247 (0.199) | 3.98 | 311.1 | 0.07 |
| 7 | 238 → 244 (0.073)<br>240 → 244 (0.086)<br>241 → 245 (0.079)<br>242 → 246 (0.136)<br>243 → 246 (0.323) | 4.07 | 304.4 | 0.15 |
| 8 | 240 → 244 (0.080)<br>241 → 244 (0.102)<br>242 → 245 (0.097)<br>242 → 247 (0.198)<br>243 → 246 (0.199)<br>243 → 247 (0.165) | 4.19 | 295.7 | 0.70 |
| 9 | 240 → 244 (0.056)<br>240 → 245 (0.067)<br>241 → 246 (0.106)<br>242 → 247 (0.209)<br>243 → 247 (0.214) | 4.28 | 290.0 | 0.22 |
| 10 | 238 → 244 (0.083)<br>240 → 245 (0.085)<br>240 → 246 (0.221)<br>241 → 245 (0.198)<br>243 → 246 (0.057)<br>243 → 247 (0.087) | 4.31 | 287.8 | 0.51 |
| 11 | 239 → 244 (0.102)<br>240 → 245 (0.112)<br>241 → 245 (0.078)<br>241 → 247 (0.055)<br>242 → 247 (0.201) | 4.40 | 282.0 | 0.15 |
| 12 | 237 → 244 (0.059)<br>241 → 245 (0.069)<br>242 → 246 (0.280)<br>242 → 249 (0.072)<br>242 → 250 (0.070)<br>243 → 246 (0.053) | 4.44 | 279.5 | 0.14 |
| 13 | 238 → 244 (0.164)<br>241 → 245 (0.079)<br>242 → 246 (0.068)<br>243 → 248 (0.137)<br>243 → 249 (0.272) | 4.52 | 274.4 | 0.14 |
| 14 | 237 → 244 (0.075)<br>239 → 244 (0.329)<br>239 → 245 (0.053)<br>241 → 245 (0.072)<br>242 → 249 (0.076)<br>243 → 249 (0.058) | 4.60 | 269.7 | 0.03 |
| 15 | 237 → 244 (0.112)<br>238 → 244 (0.062) | 4.65 | 266.6 | 0.03 |

| | | | | |
|---|---|---|---|---|
| | 240 → 245 (0.092)<br>241 → 247 (0.055)<br>242 → 246 (0.069)<br>242 → 248 (0.068)<br>242 → 249 (0.077)<br>243 → 249 (0.086) | | | |

**Table S5.** Electronically excited singlet states of compound **4b** with major orbital contributions (>5%), energies and oscillator strength calculated at the B3LYP/def2-TZVP level of theory using TD-DFT. Highest occupied molecular orbital (HOMO) 243, lowest unoccupied molecular orbital (LUMO) 244.

| State | Orbital Contribution | Energy (eV) | Wavelength (nm) | $f_{osc}$ |
|---|---|---|---|---|
| 1 | 243 → 244 (0.932) | 2.55 | 486.6 | 0.54 |
| 2 | 242 → 244 (0.877)<br>243 → 245 (0.054) | 2.70 | 458.4 | 0.36 |
| 3 | 240 → 244 (0.106)<br>241 → 244 (0.843) | 3.07 | 404.1 | 0.05 |
| 4 | 242 → 244 (0.054)<br>243 → 245 (0.885) | 3.17 | 391.3 | 0.45 |
| 5 | 240 → 244 (0.638)<br>241 → 244 (0.065)<br>242 → 245 (0.130)<br>243 → 246 (0.084) | 3.28 | 377.6 | 0.02 |
| 6 | 240 → 244 (0.169)<br>242 → 245 (0.701) | 3.33 | 372.2 | 0.41 |
| 7 | 243 → 246 (0.795) | 3.50 | 353.8 | 0.05 |
| 8 | 241 → 245 (0.174)<br>242 → 246 (0.233)<br>243 → 247 (0.425) | 3.56 | 348.6 | 0.07 |
| 9 | 242 → 246 (0.431)<br>242 → 247 (0.068)<br>243 → 247 (0.332) | 3.66 | 338.5 | 0.14 |
| 10 | 238 → 244 (0.100)<br>239 → 244 (0.053)<br>241 → 245 (0.228)<br>242 → 246 (0.227)<br>242 → 247 (0.268) | 3.75 | 330.4 | 0.05 |
| 11 | 237 → 244 (0.057)<br>239 → 244 (0.745)<br>242 → 247 (0.054) | 3.76 | 329.5 | 0.03 |
| 12 | 237 → 244 (0.087)<br>238 → 244 (0.471)<br>242 → 247 (0.113)<br>243 → 248 (0.196) | 3.82 | 324.3 | 0.01 |
| 13 | 238 → 244 (0.079)<br>241 → 245 (0.357)<br>242 → 247 (0.247)<br>243 → 248 (0.118) | 3.84 | 322.7 | 0.34 |
| 14 | 237 → 244 (0.300)<br>239 → 244 (0.095)<br>241 → 245 (0.070)<br>243 → 248 (0.287)<br>243 → 249 (0.062) | 3.87 | 320.4 | 0.25 |
| 15 | 237 → 244 (0.200)<br>238 → 244 (0.105)<br>240 → 245 (0.081)<br>242 → 247 (0.137)<br>243 → 247 (0.041)<br>243 → 248 (0.233) | 3.88 | 319.2 | 0.25 |

**Table S6.** Electronically excited singlet states of compound **4b** with major orbital contributions (>5%), energies and oscillator strength calculated at the ωB97X/def2-TZVP level of theory. HOMO no. 243, LUMO no. 244.

| State | Orbital Contribution | Energy (eV) | Wavelength (nm) | $f_{osc}$ |
|---|---|---|---|---|
| 1 | 243 → 244 (0.809) | 3.23 | 383.8 | 0.92 |
| 2 | 242 → 244 (0.613)<br>243 → 245 (0.132) | 3.41 | 363.2 | 0.18 |
| 3 | 240 → 244 (0.379)<br>241 → 244 (0.282)<br>242 → 244 (0.075)<br>243 → 245 (0.076) | 3.84 | 323.0 | 0.44 |
| 4 | 240 → 244 (0.107)<br>242 → 244 (0.066)<br>242 → 245 (0.103)<br>243 → 245 (0.512) | 4.00 | 310.3 | 0.95 |
| 5 | 242 → 244 (0.069)<br>242 → 245 (0.478)<br>243 → 246 (0.089) | 4.15 | 298.5 | 0.86 |
| 6 | 237 → 244 (0.078)<br>240 → 244 (0.092)<br>241 → 244 (0.100)<br>243 → 246 (0.247) | 4.30 | 288.4 | 0.13 |
| 7 | 236 → 244 (0.088)<br>241 → 245 (0.083)<br>242 → 245 (0.051)<br>242 → 246 (0.331) | 4.40 | 281.6 | 0.47 |
| 8 | 238 → 244 (0.128)<br>240 → 247 (0.068)<br>241 → 244 (0.051)<br>241 → 245 (0.146)<br>242 → 246 (0.063)<br>243 → 246 (0.096) | 4.57 | 271.2 | 0.72 |
| 9 | 240 → 244 (0.079)<br>241 → 244 (0.082)<br>241 → 246 (0.099)<br>242 → 246 (0.054)<br>243 → 246 (0.222) | 4.60 | 269.4 | 0.27 |
| 10 | 240 → 245 (0.114)<br>241 → 245 (0.134)<br>242 → 246 (0.141) | 4.68 | 264.7 | 0.24 |
| 11 | 241 → 244 (0.054)<br>242 → 247 (0.077)<br>243 → 247 (0.192)<br>243 → 248 (0.274)<br>243 → 249 (0.060) | 4.71 | 263.5 | 0.11 |
| 12 | 237 → 244 (0.064)<br>241 → 245 (0.062)<br>242 → 248 (0.075)<br>242 → 250 (0.071)<br>243 → 247 (0.062)<br>243 → 249 (0.052) | 4.75 | 261.1 | 0.06 |
| 13 | 240 → 245 (0.076)<br>242 → 247 (0.065)<br>242 → 249 (0.112)<br>243 → 249 (0.113) | 4.84 | 256.1 | 0.08 |
| 14 | 239 → 244 (0.081)<br>239 → 245 (0.115)<br>239 → 246 (0.068)<br>242 → 249 (0.070) | 4.95 | 250.6 | 0.20 |
| 15 | 236 → 244 (0.090)<br>240 → 246 (0.051)<br>241 → 246 (0.058)<br>243 → 249 (0.073)<br>243 → 250 (0.125) | 4.98 | 248.9 | 0.03 |

**Table S7.** Electronically excited singlet states of compound **4b** with major orbital contributions (>5%), energies and oscillator strength calculated at the CAM-B3LYP/def2-TZVP level of theory. HOMO no. 243, LUMO no. 244.

| State | Orbital Contribution | Energy (eV) | Wavelength (nm) | $f_{osc}$ |
|---|---|---|---|---|
| 1 | 243 → 244 (0.883) | 3.00 | 412.8 | 0.86 |
| 2 | 242 → 244 (0.738)<br>243 → 245 (0.100) | 3.19 | 389.2 | 0.24 |
| 3 | 240 → 244 (0.364)<br>241 → 244 (0.371)<br>242 → 244 (0.064)<br>243 → 245 (0.092) | 3.59 | 345.0 | 0.36 |
| 4 | 240 → 244 (0.114)<br>242 → 244 (0.066)<br>243 → 245 (0.656) | 3.72 | 332.9 | 0.72 |
| 5 | 242 → 245 (0.629)<br>243 → 246 (0.110) | 3.87 | 320.6 | 0.60 |
| 6 | 240 → 244 (0.192)<br>241 → 244 (0.190)<br>242 → 245 (0.060)<br>243 → 246 (0.222) | 4.00 | 310.1 | 0.21 |
| 7 | 236 → 244 (0.070)<br>238 → 244 (0.053)<br>241 → 245 (0.107)<br>242 → 246 (0.338)<br>243 → 247 (0.128) | 4.14 | 299.5 | 0.23 |
| 8 | 240 → 244 (0.117)<br>241 → 244 (0.202)<br>243 → 246 (0.433) | 4.24 | 292.6 | 0.41 |
| 9 | 238 → 244 (0.129)<br>242 → 247 (0.162)<br>243 → 247 (0.385) | 4.33 | 286.0 | 0.27 |
| 10 | 238 → 244 (0.103)<br>241 → 245 (0.068)<br>241 → 246 (0.084)<br>242 → 246 (0.296)<br>243 → 248 (0.077) | 4.35 | 285.2 | 0.17 |
| 11 | 238 → 244 (0.074)<br>240 → 245 (0.101)<br>241 → 245 (0.123)<br>243 → 248 (0.196) | 4.43 | 280.1 | 0.03 |
| 12 | 237 → 244 (0.101)<br>239 → 244 (0.066)<br>241 → 245 (0.140)<br>242 → 248 (0.080)<br>243 → 249 (0.119) | 4.46 | 277.7 | 0.03 |
| 13 | 240 → 245 (0.098)<br>242 → 247 (0.078)<br>242 → 249 (0.107)<br>243 → 249 (0.190) | 4.56 | 271.7 | 0.13 |
| 14 | 239 → 244 (0.151)<br>239 → 245 (0.104)<br>240 → 247 (0.055)<br>241 → 245 (0.080)<br>242 → 247 (0.142)<br>242 → 249 (0.059)<br>243 → 247 (0.054) | 4.65 | 266.9 | 0.35 |
| 15 | 239 → 244 (0.064)<br>241 → 246 (0.063)<br>243 → 248 (0.234)<br>243 → 249 (0.114) | 4.70 | 264.0 | 0.06 |

## 3.3 Bond Length Analysis

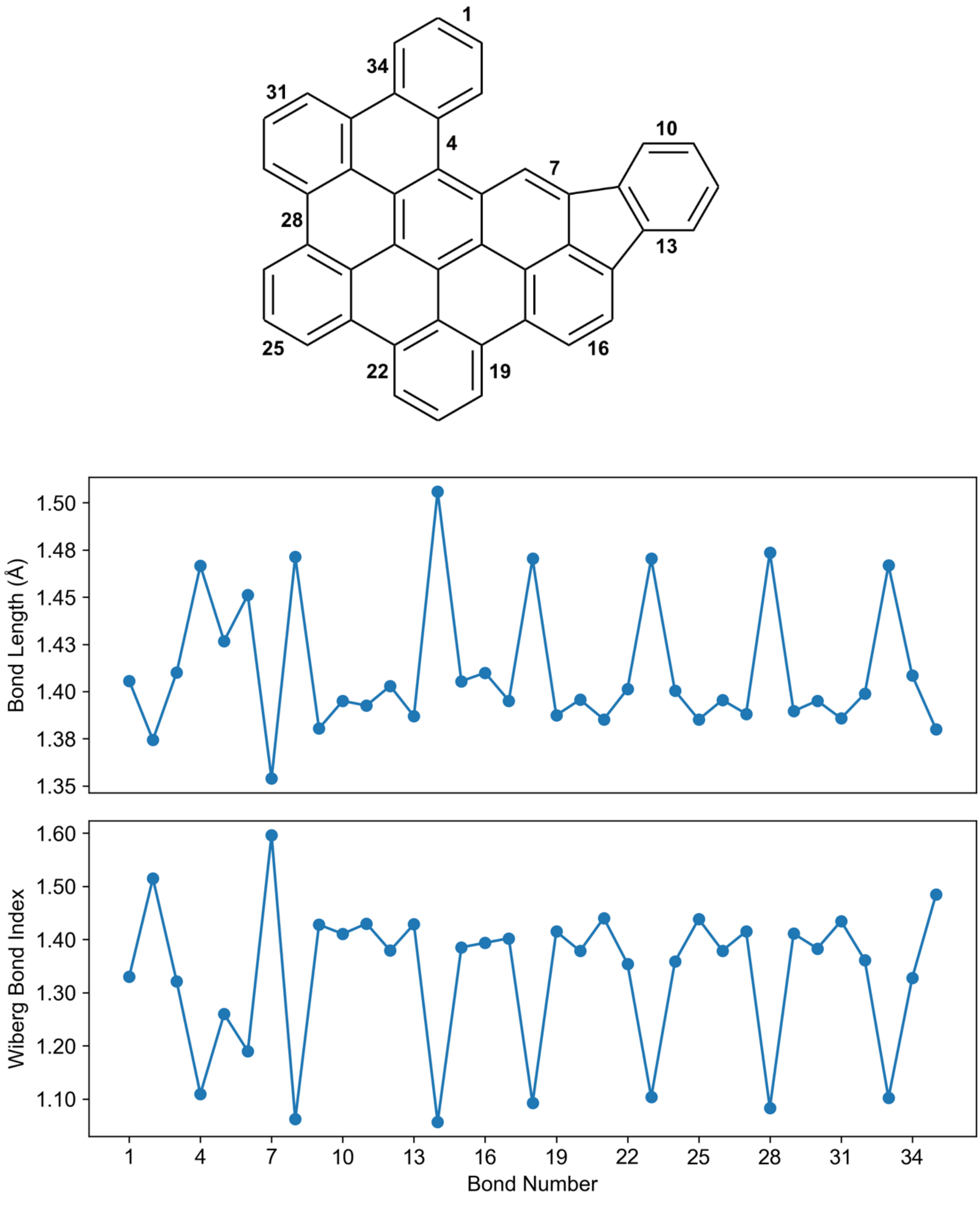


**Figure S1.** Bond length alternation (BLA) (top) and Wiberg bond indices (bottom) of outer bonds of the PAH scaffold of compound **2** calculated at the ωB97X-D3BJ/def-TZVP level of theory.

## 3.4 Orbital Analysis

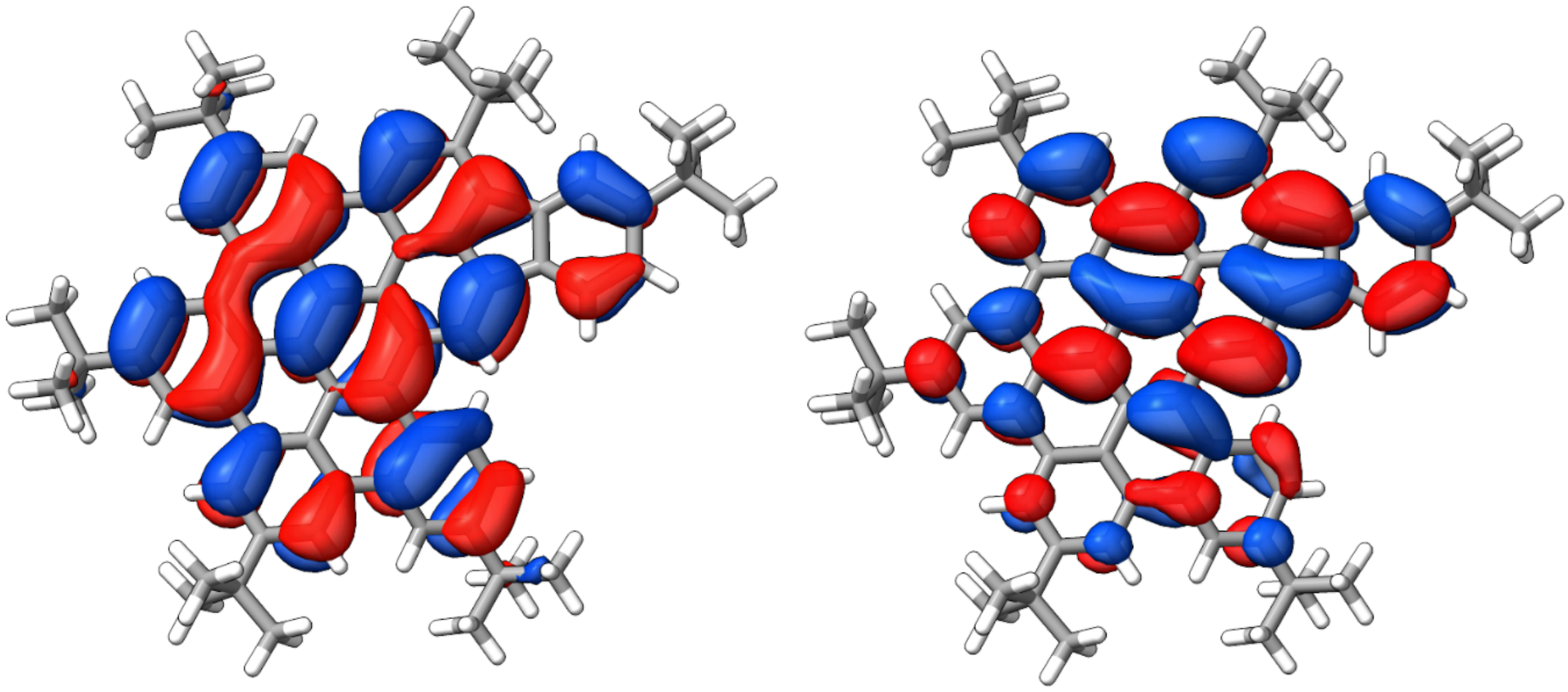

**Figure S2.** Highest occupied molecular orbital (HOMO), isovalue 0.015 (left) and lowest unoccupied molecular orbital (LUMO), isovalue 0.015 (right) of compound **2**.

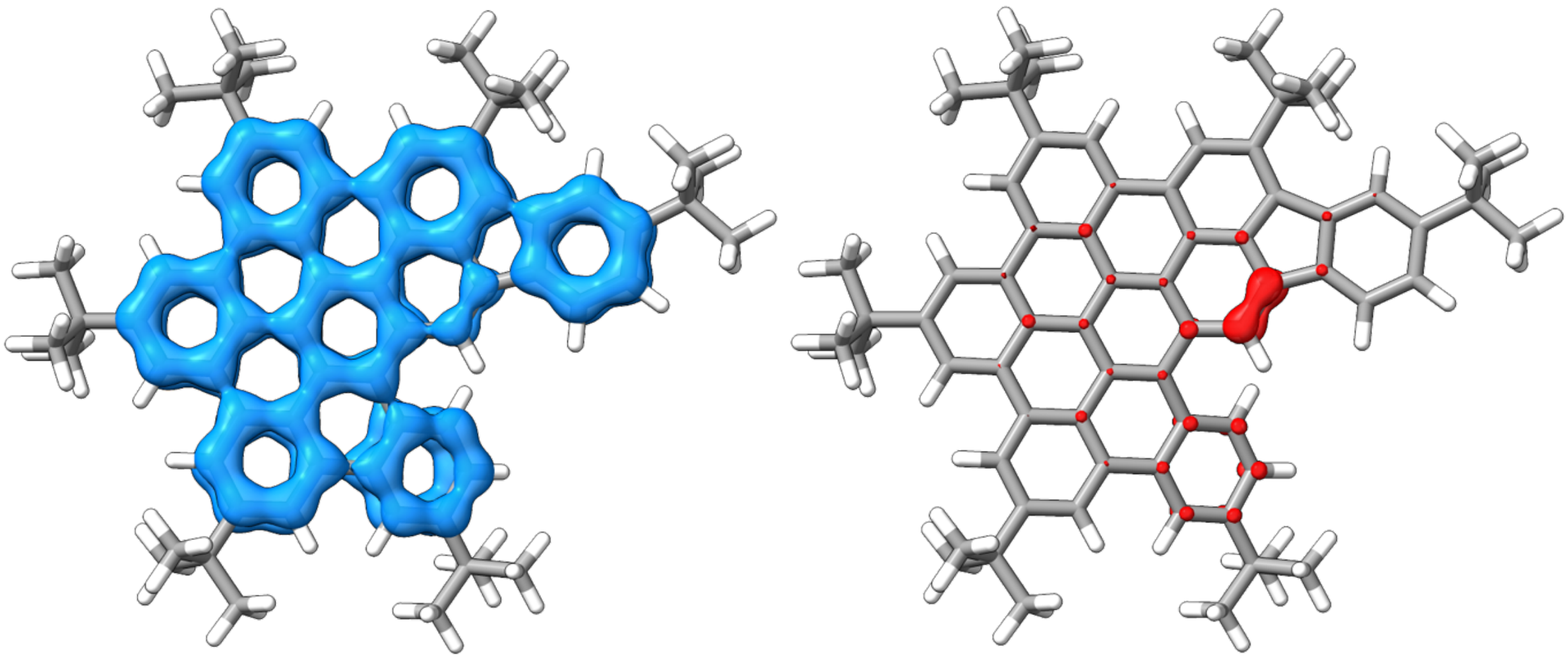

**Figure S3.** Isosurface of electron density of delocalized π-bonds ($EDDB_{\pi}$), isovalue 0.02 (left). Isosurface of electron density of localized π-bonds ($EDLB_{\pi}$), isovalue 0.02 (right).

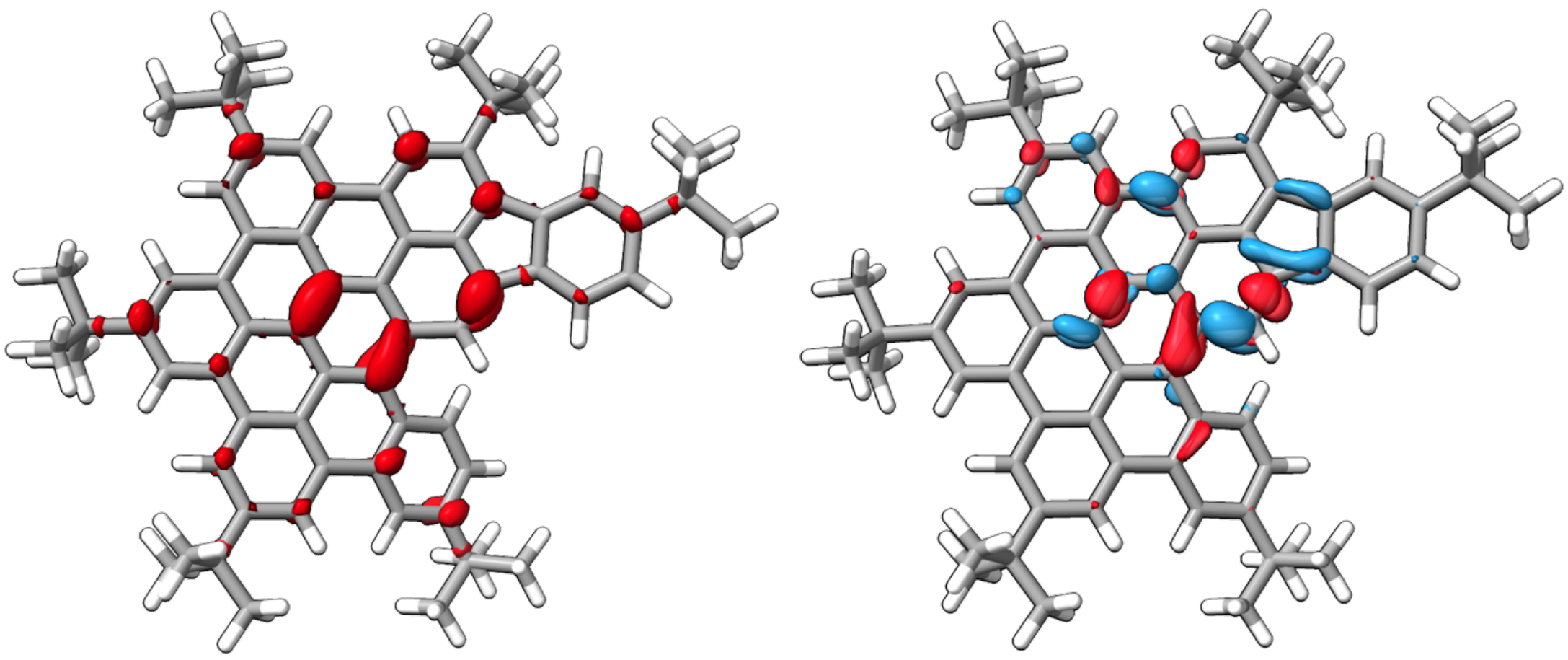

**Figure S4.** Left: Isosurface of the Fukui function $f^{-}(r) = \rho_N(r) - \rho_{N-1}(r)$, with $N$ being the number of electrons in the system, depicting reactive sites of the molecule towards electrophilic attacks, isovalue 0.002. Right: Dual descriptor $\Delta f(r) = f^{+}(r) - f^{-}(r)$, $\Delta f < 0$ for electrophilic reaction sites (red) and $\Delta f > 0$ for nucleophilic reaction sites (blue), isovalues -0.002 and 0.002.

## 3.5 Inversion Barrier

Helical inversion barrier calculations were performed at the ωB97X-D3BJ/def2-SVP level of theory to reduce the run time of the calculations. To investigate the inversion barrier of compounds **2**, **3a**, **3b**, **4a**, and **4b**, we performed a relaxed redundant coordinate scan by varying the dihedral angle of the [4]helicene unit starting from the calculated ground state geometry. The discontinuities in the curve are caused by a flip of the aromatic backbone resulting in a strain release and energy gain. The applicability of this method for small helicene systems was shown before.[22]

To evaluate the inversion barrier during the bromination reaction, a relaxed redundant coordinate scan was performed on the geometries of the respective Wheland intermediates. To prevent the Wheland intermediate from disintegrating during the calculation, the coordinates of the bromine, hydrogen, and carbon atom involved in its formation were fixed by constraints, while all other atoms were allowed to move freely.

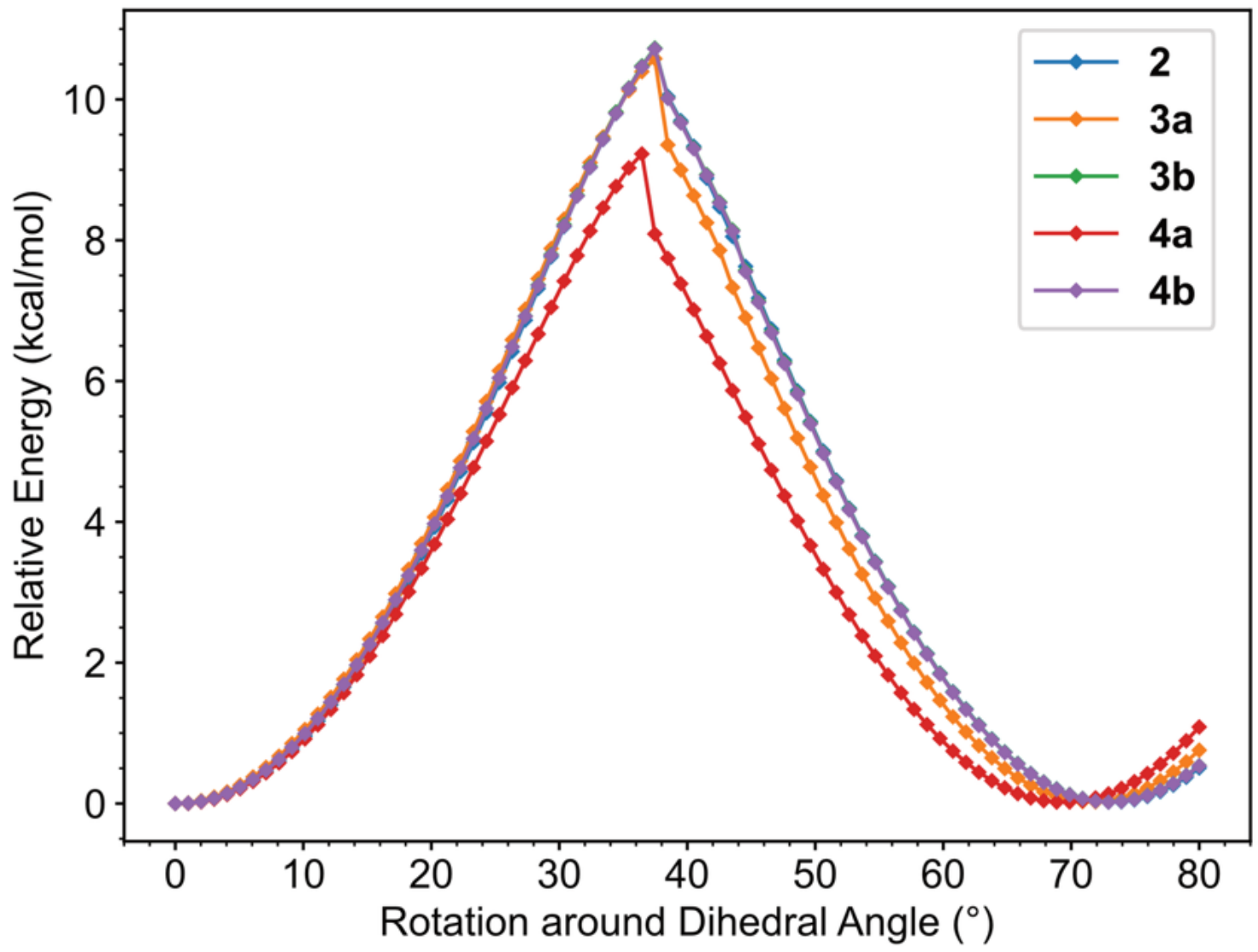


**Figure S5.** Inversion barrier of compounds **2, 3a, 3b, 4a**, and **4b** calculated at the ωB97X-D3BJ/def2-SVP level of theory in dependence of the dihedral angle of the [4]helicene unit.

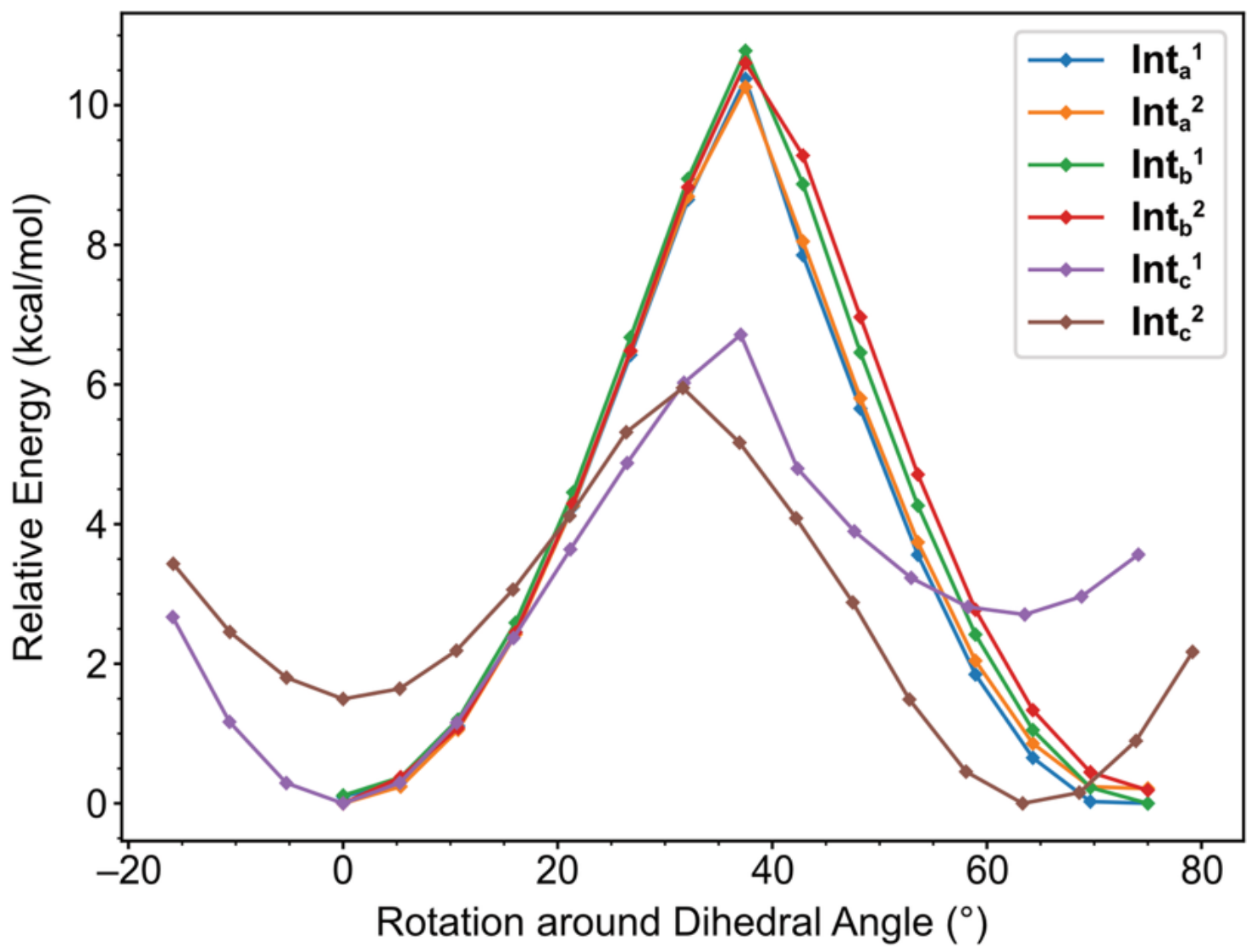


**Figure S6.** Inversion barrier of Wheland intermediates **Int$_a$$^1$**, **Int$_a$$^2$**, **Int$_b$$^1$**, **Int$_b$$^2$**, **Int$_c$$^1$**, and **Int$_c$$^2$** calculated at the ωB97X-D3BJ/def2-SVP level of theory in dependence of the dihedral angle of the [4]helicene unit.

## 3.6 Transition States and Intermediates

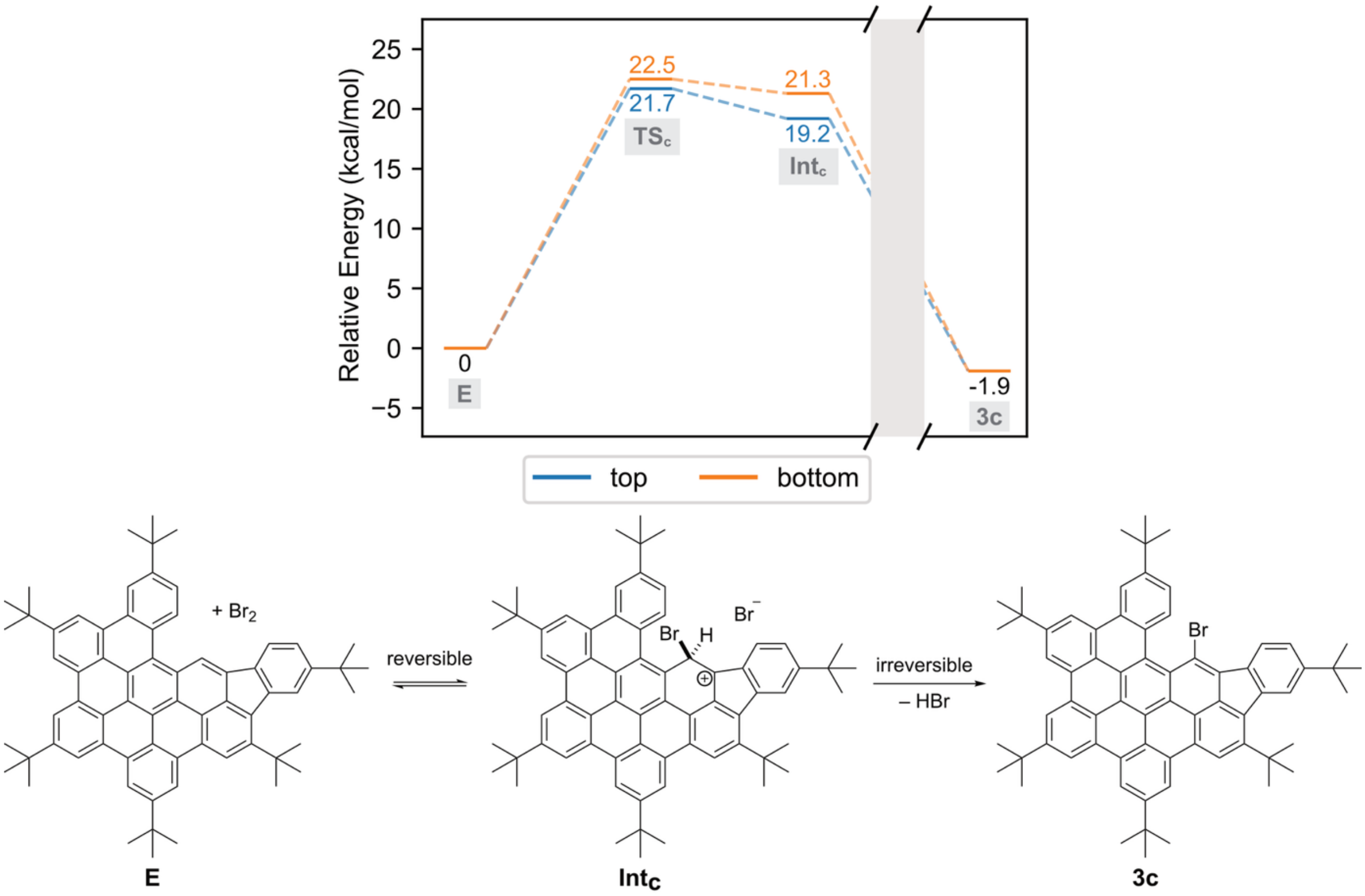


**Figure S7.** Top: Energy diagram for the formation of the brominated intermediates **Int$_c$$^1$** (blue) and **Int$_c$$^2$** (orange) *via* the corresponding transition states, **TS$_c$$^1$** (blue) and **TS$_c$$^2$** (orange), to the product **3c** after HBr elimination. Bottom: Chemical structures of the investigated reaction path.

According to the Eyring equation (1), which connects the transition state theory with the concept of the chemical equilibrium,[23] the rate constant $k$ is related to the reaction enthalpy by

$$k = \kappa \cdot \frac{k_b \cdot T}{h} \cdot e^{-\frac{\Delta G^{\ddagger}}{RT}} \tag{1}$$

where $\kappa$ is the transmission coefficient, $k_b$ is the Boltzmann constant and $h$ is the Planck constant. For two concurring reactions, the equation is as follows:

$$\frac{k_1}{k_2} = \exp\left(-\frac{\Delta G_1^{\ddagger} - \Delta G_2^{\ddagger}}{RT}\right) = \exp\left(-\frac{\Delta\Delta G^{\ddagger}}{RT}\right) \tag{2}$$

# 4 NMR Discussion

In the $^{1}$H NMR spectrum of the product mixture **3a**/**3b** (bottom), the resonance pattern of the aromatic protons shows characteristic deviations from the spectrum of precursor **2** (top), which allow the substituted aromatic positions to be pinpointed on the basis of the altered coupling patterns and thereby provide direct spectroscopic evidence for the structure of **3a** and **3b** (**Figure S8**).

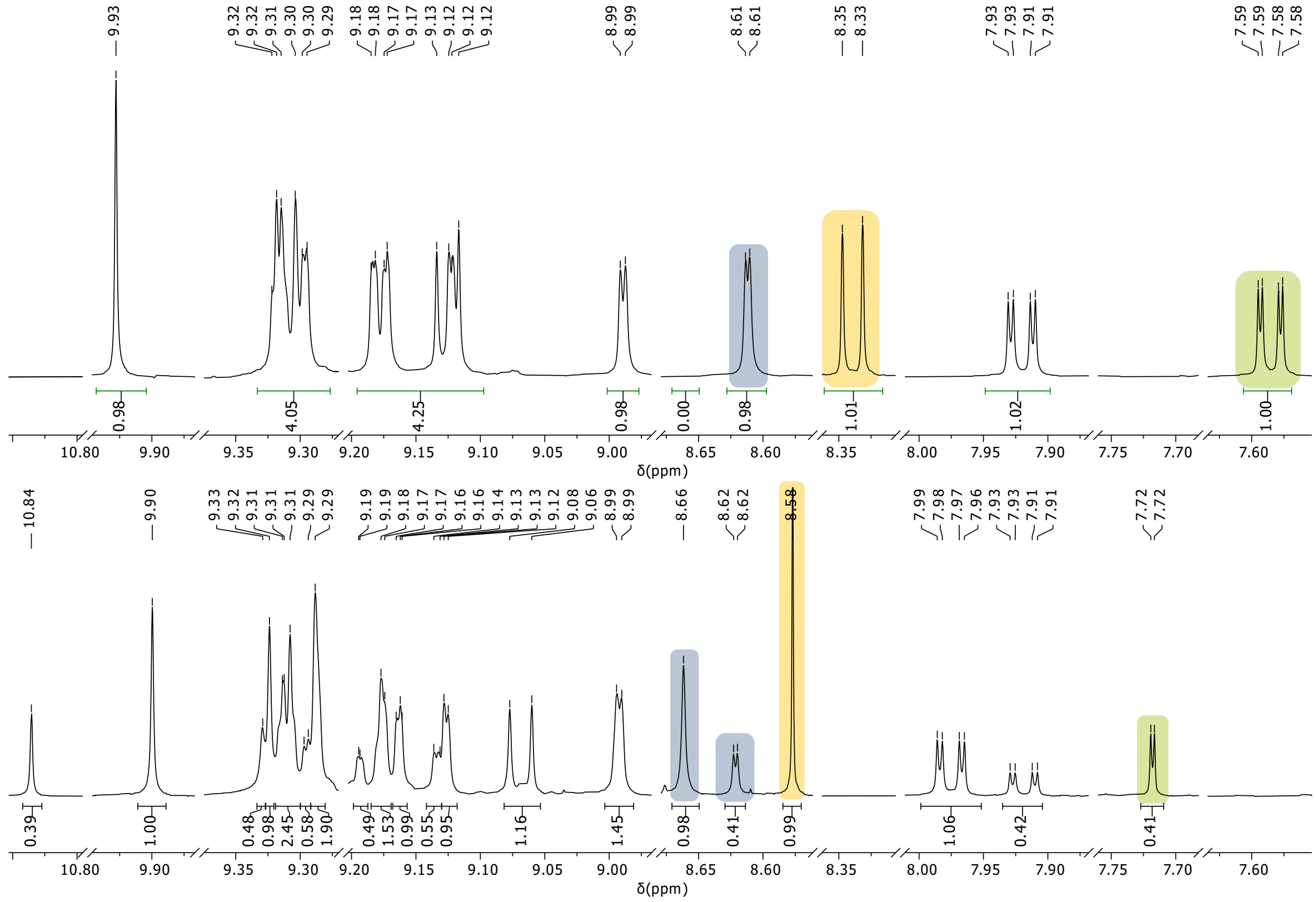


**Figure S8.** $^{1}$H NMR Spectra of compound **2** (top) and the mixture of compounds **3a** and **3b** (bottom).

The missing doublet (yellow) of the $^{3}J$ coupling at 8.33 ppm in the spectrum of **3a**/**3b** (bottom) compared to the $^{1}$H NMR spectrum of **2** (top) indicates a substitution of the H atoms 15 and 16 (yellow and green) in the peripheral area. Instead of a doublet of doublets (green) at 7.58 ppm, the proton in position 16 (green) shows only a doublet at 7.72 ppm, which only integrates over one proton in one out of the two products. This also suggests substitution of 15 and 16. The doublet (blue) at 8.61 ppm, which integrates over the aromatic proton 18 (blue) in **2** also corresponds to only one proton in one of the two products, which also indicates the substitution of proton 16 (green).

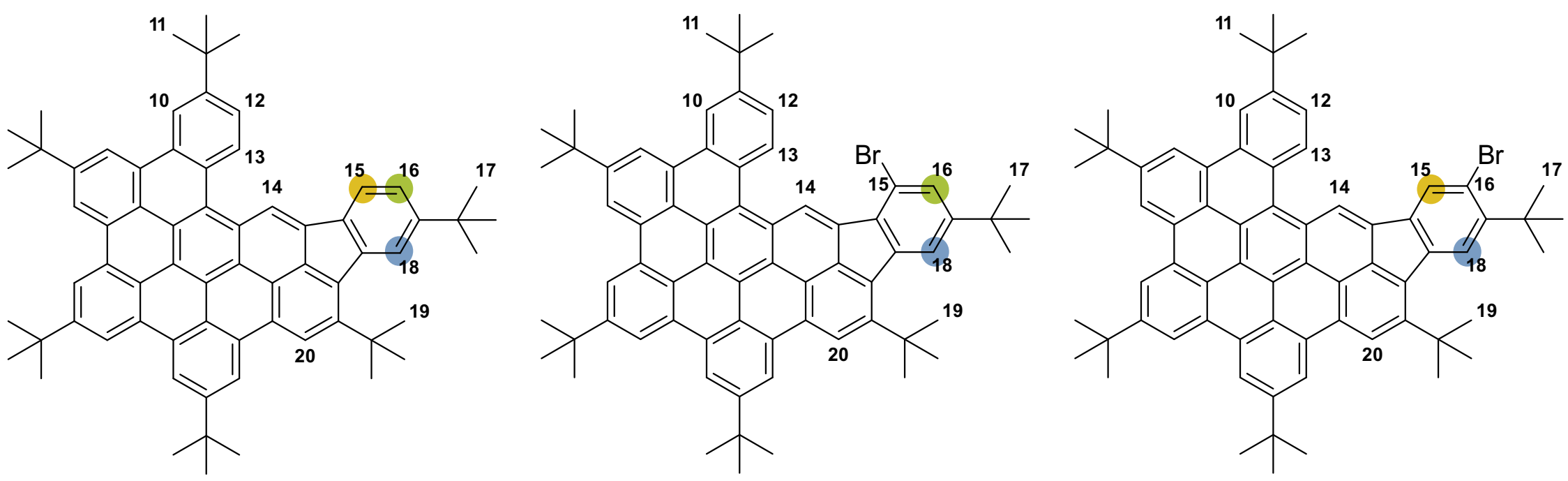


**2** **3a** **3b**

**Figure S9.** Structures of compound **2**, **3a** and **3b** with numbering of the assignable $^{1}$H NMR signals.

# 5 Reaction Conditions

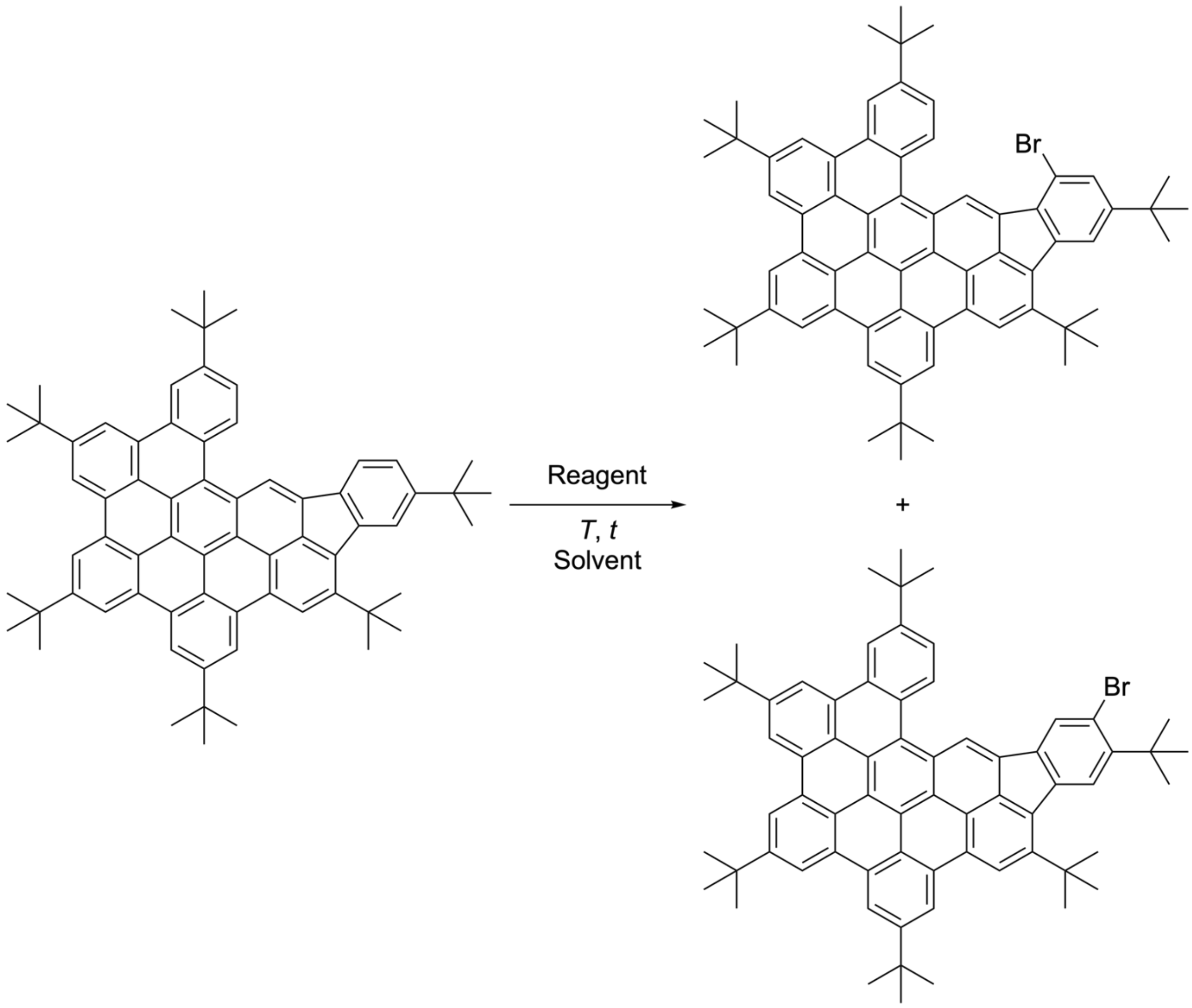


**Table S8.** Investigated reaction conditions for the post-functionalization of compound **2**.

| No. | Reagent | Equivalents | *T* (°C) | *t* (h) | Solvent | Conversion | Product | Ratio |
|---|---|---|---|---|---|---|---|---|
| **1** | $Br_2$ | 30 | -78 | 96 | DCM | Incomplete[a] | **3a**, **3b** | 1:1 |
| **2** | $Br_2$ | 30 | -40 | 22 | DCM | 100 | **3a**, **3b** | 1:1 |
| **3** | $Br_2$ | 7 | 0 | 4.5 | DCM | 100 | **3a**, **3b** | 1:2.5 |
| **4** | $Br_2$ | 10 | 40 | 2.5 | DCM | 100 | **3a**, **3b** | 1:3 |
| **5** | $Br_2$ | 9 | 50 | 2 | $CHCl_3$ | 100 | **3a**, **3b** | 1:3 |
| **6** | $Br_2$ | 5 | 60 | 2 | $CCl_4$ | 0[b] | **–** | – |
| **7** | $Br_2$ | 10 | 76 | 1 | $CCl_4$ | 0[b] | **–** | – |
| **8** | $I_2$ | 4 | 40 | 3 | DCM | 0 | – | – |
| **9** | ICl | 4 | 20 | 2 | DCM | 0[b] | – | – |
| **10** | ICl | 2 | 0 | 1.5 | DCM | 0[b] | – | – |
| **11** | BrCN | 2 | 20 | 2 | THF | 0 | – | – |

[a]Reaction stopped prematurely. [b]Decomposition.

**Table S9.** Tested solvent mixtures and retention factors on thin-layer chromatography for the separation of **3a**/**3b**.

| No. | Solvent 1 | Solvent 2 | Ratio | $R_f$ | Separation |
|---|---|---|---|---|---|
| **1** | *c*-Hexane | – | pure | 0.18 | No |
| **2** | *c*-Hexane | DCM | 10:1 | 0.48 | No |
| **3** | *c*-Hexane | EtOAc | 10:1 | 0.72 | No |
| **4** | $CS_2$ | – | pure | 0.70 | No |
| **5** | $CS_2$ | *c*-Hexane | 1:1 | 0.38 | No |
| **6** | Toluene | – | pure | 1.00 | No |

# 6 Crystallographic Data

The supplementary crystallographic data for compounds **3a** and **3b** can be found in the CCDC under the deposit number 2512288. These data can be obtained free of charge from www.ccdc.cam.ac.uk/data_request/cif.

The disorder at the *tert*-butyl groups (C117-C119 and C144-C146) is resolved by using PART 1 and 2 with a ratio of 85.3% to 14.7%. The disorder at the *tert*-butyl groups (C66-C71) is resolved by using PART 1 and 2 with a ratio of 65.6% to 34.4%. Furthermore, one molecule of the asymmetric unit shows a disorder of the bromine atom (Br1 and Br2) which was resolved by using PART 1 and 2 with a ratio of 82.9% to 17.1%. A cocrystallized dichloromethane with an occupation of 29.3% was also found in the asymmetric unit. The unit cell contains two dichloromethane molecules which have been treated as a diffuse contribution to the overall scattering without specific atom positions by SQUEEZE/PLATON.[5]

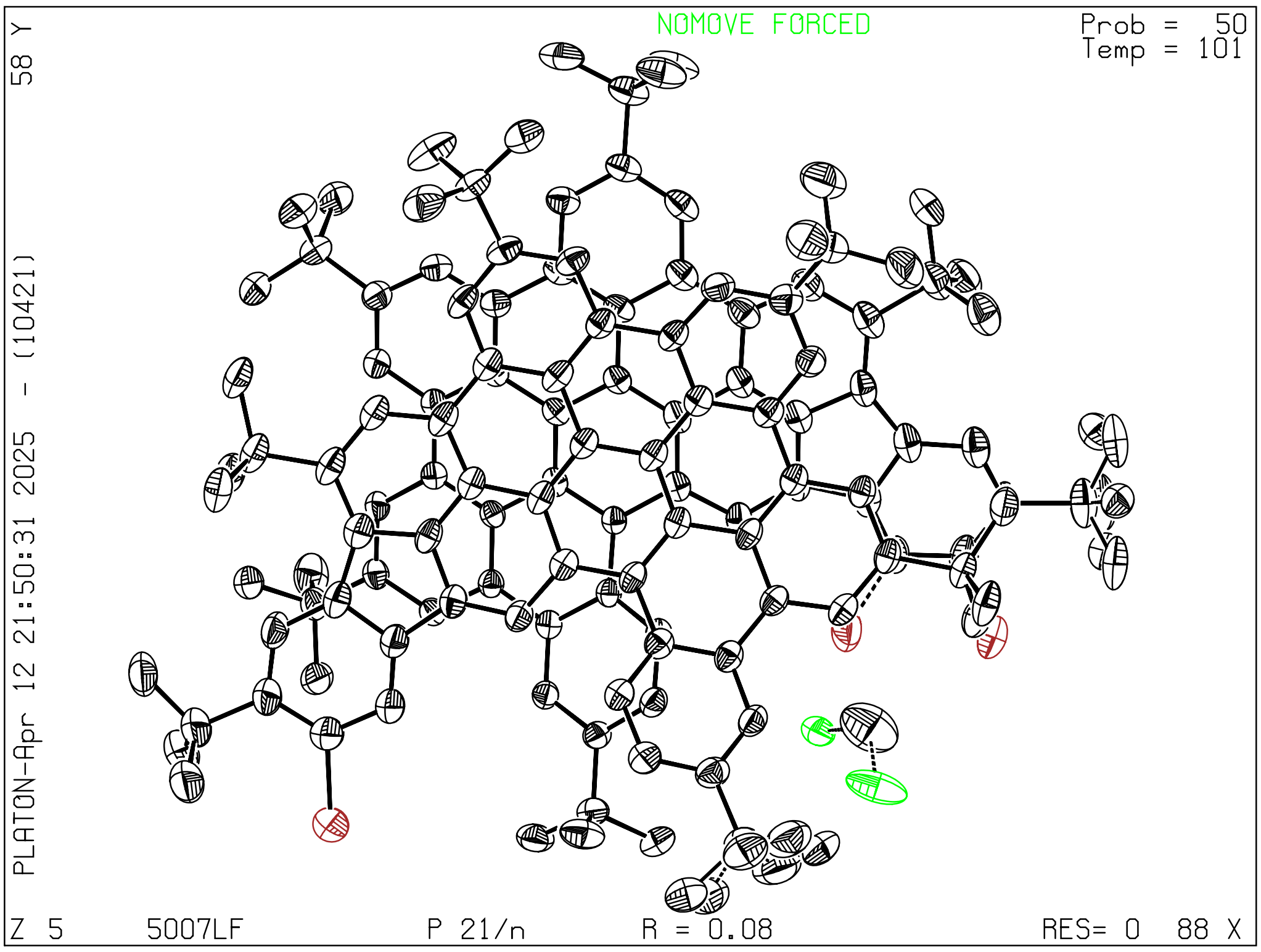


**Figure S10.** Asymmetric unit of compounds **3a** and **3b** in the solid state, carbon (black), bromine (brown), chlorine (green), hydrogen atoms omitted for clarity. Thermal ellipsoids shown at the 50% probability level. Disorder depicted with dashed bonds.

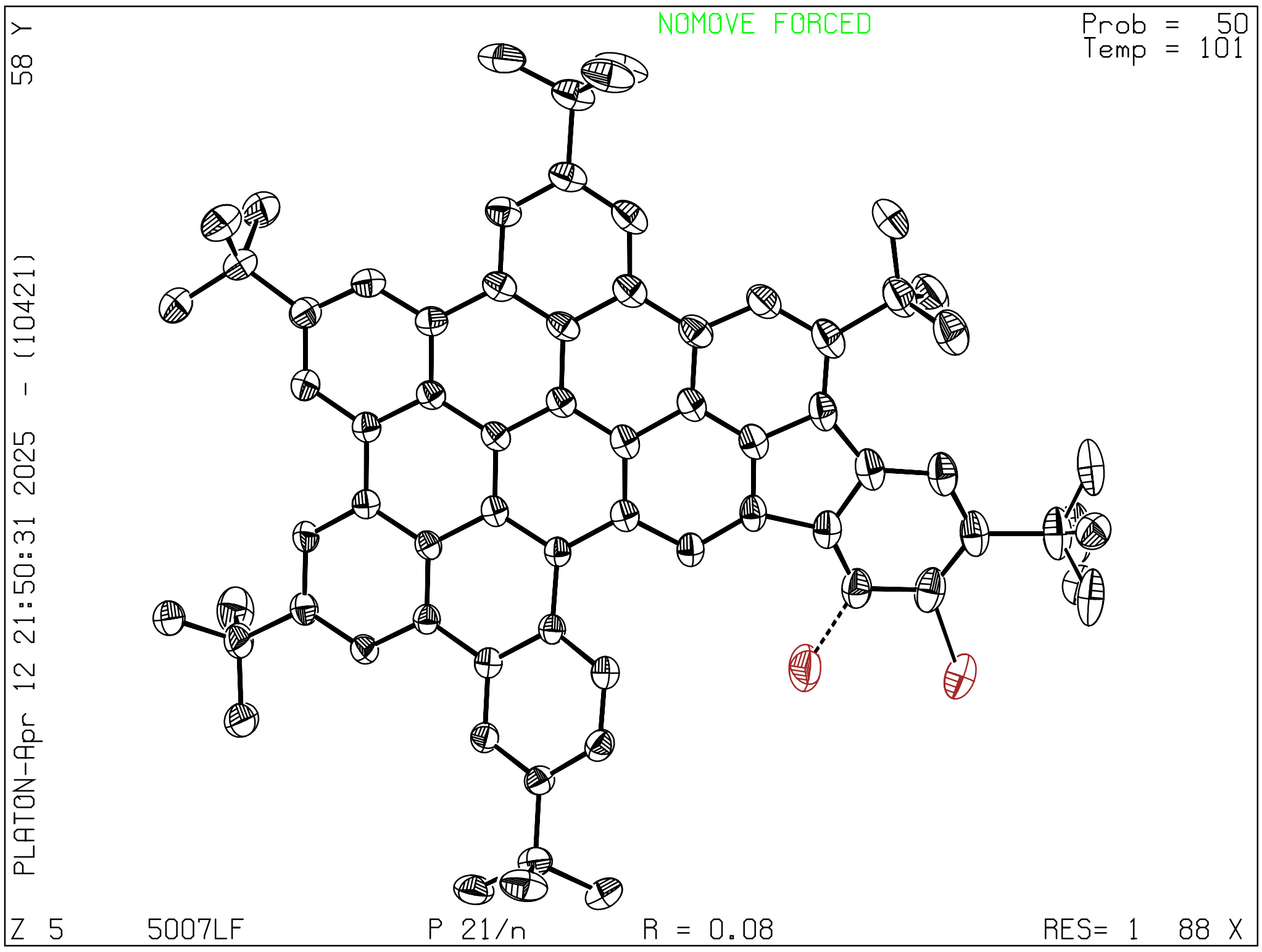


**Figure S11.** Structure of (*P*)-**3a** and (*P*)-**3b** in the solid state, carbon (black), bromine (brown), hydrogen atoms omitted for clarity. Thermal ellipsoids shown at the 50% probability level. Disorder depicted with dashed bonds.

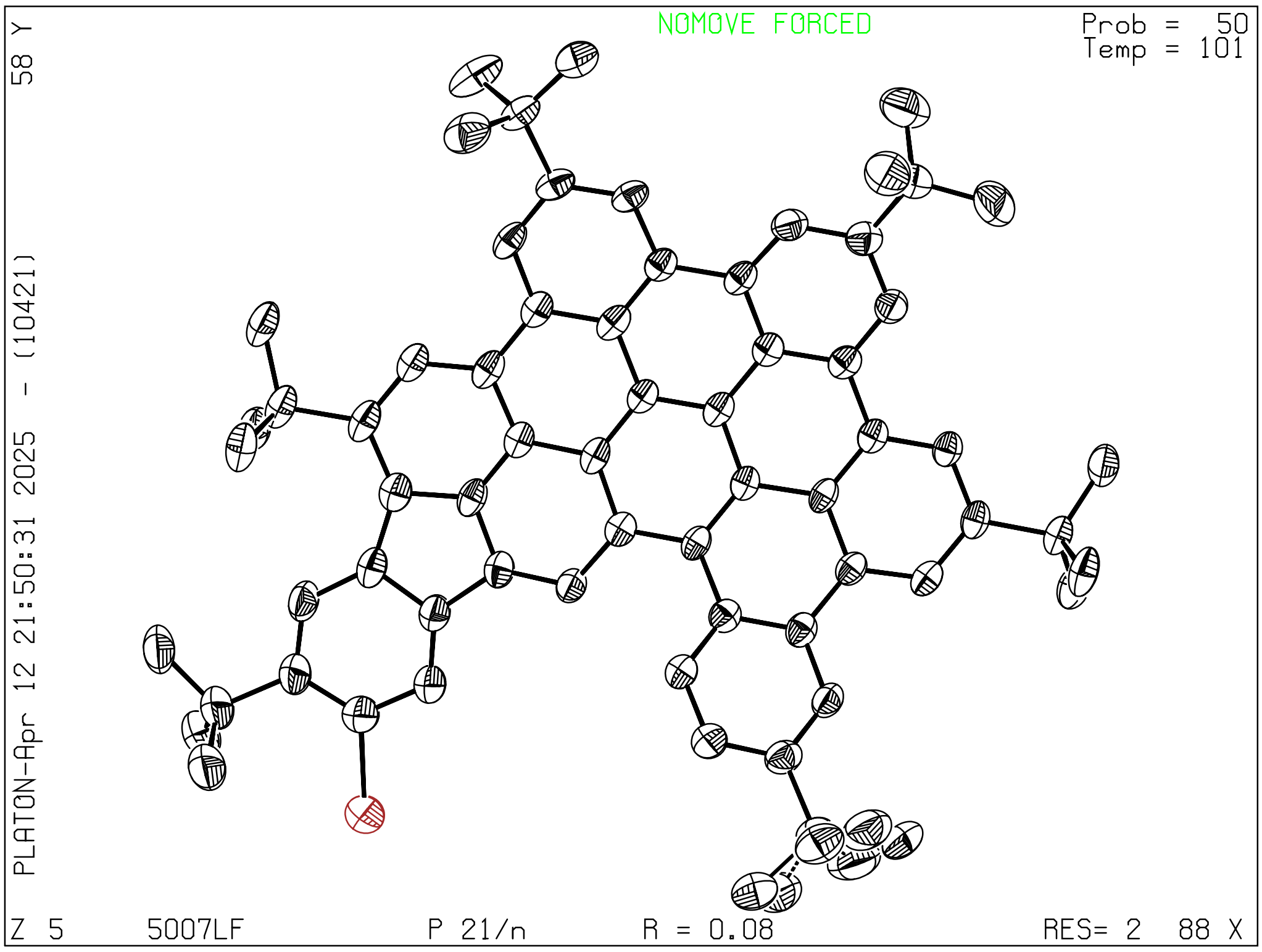


**Figure S12.** Structure of (*M*)-**3a** in the solid state, carbon (black), bromine (brown), hydrogen atoms omitted for clarity. Thermal ellipsoids shown at the 50% probability level. Disorder depicted with dashed bonds.

| | |
|---|---|
| CCDC number | 2512288 |
| Empirical formula | $C_{136.29}H_{134.59}Br_2Cl_{0.59}$ |
| Formula weight | 1953.13 |
| Temperature [K] | 101(2) |
| Crystal system | monoclinic |
| Space group (number) | $P2_1/n$ (14) |
| *a* [Å] | 20.1607(15) |
| *b* [Å] | 17.0006(13) |
| *c* [Å] | 33.042(2) |
| α [°] | 90 |
| β [°] | 102.018(4) |
| γ [°] | 90 |
| Volume [$Å^3$] | 11076.7(14) |
| *Z* | 4 |
| $\rho_{calc}$ [$gcm^{-3}$] | 1.171 |
| $\mu$ [$mm^{-1}$] | 1.430 |
| *F*(000) | 4129 |
| Crystal size [$mm^3$] | 0.082×0.231×0.266 |
| Crystal color | brown |
| Crystal shape | plate |
| Radiation | Cu$K_\alpha$ (λ=1.54178 Å) |
| 2θ range [°] | 4.74 to 130.16 (0.85 Å) |
| Index ranges | −23 ≤ h ≤ 23<br>−20 ≤ k ≤ 19<br>−38 ≤ l ≤ 38 |
| Reflections collected | 201010 |
| Independent reflections | 18819<br>$R_{int}$ = 0.0947<br>$R_{sigma}$ = 0.0370 |
| Completeness to θ = 65.080° | 99.7% |
| Data / Restraints / Parameters | 18819 / 78 / 1375 |
| Absorption correction $T_{min}/T_{max}$ (method) | 0.6135 / 0.7538<br>(multi-scan) |
| Goodness-of-fit on $F^2$ | 1.063 |
| Final *R* indexes<br>[$I \geq 2\sigma(I)$] | $R_1$ = 0.0821<br>w$R_2$ = 0.2304 |
| Final *R* indexes<br>[all data] | $R_1$ = 0.1053<br>w$R_2$ = 0.2510 |
| Largest peak/hole [$eÅ^{-3}$] | 1.63/−1.47 |

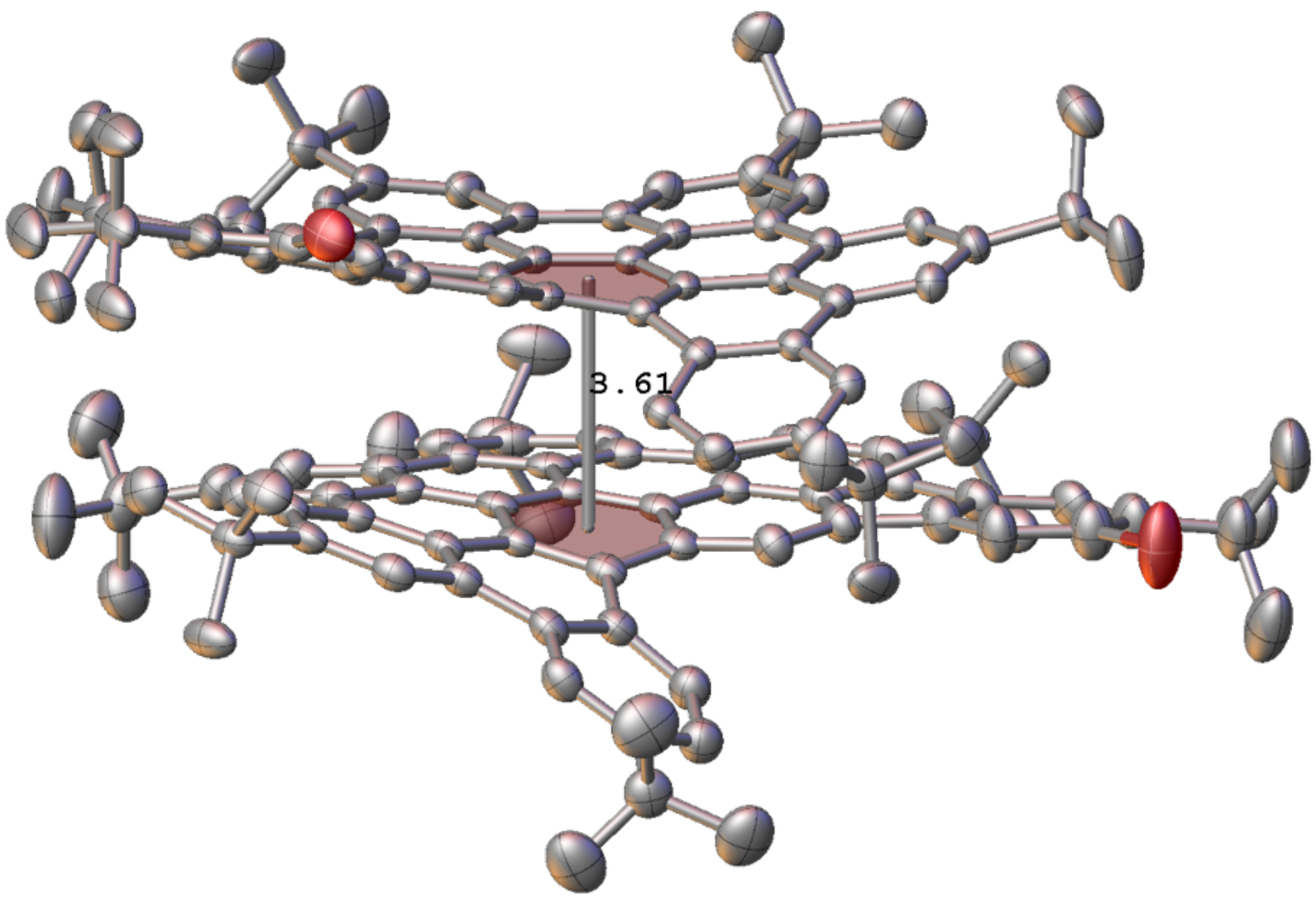


**Figure S13.** Interlayer distance in the solid state. Thermal ellipsoids shown at the 50% probability level, hydrogen atoms and disorder omitted for clarity.

# 7 UV/vis, Fluorescence and Lifetime Measurements

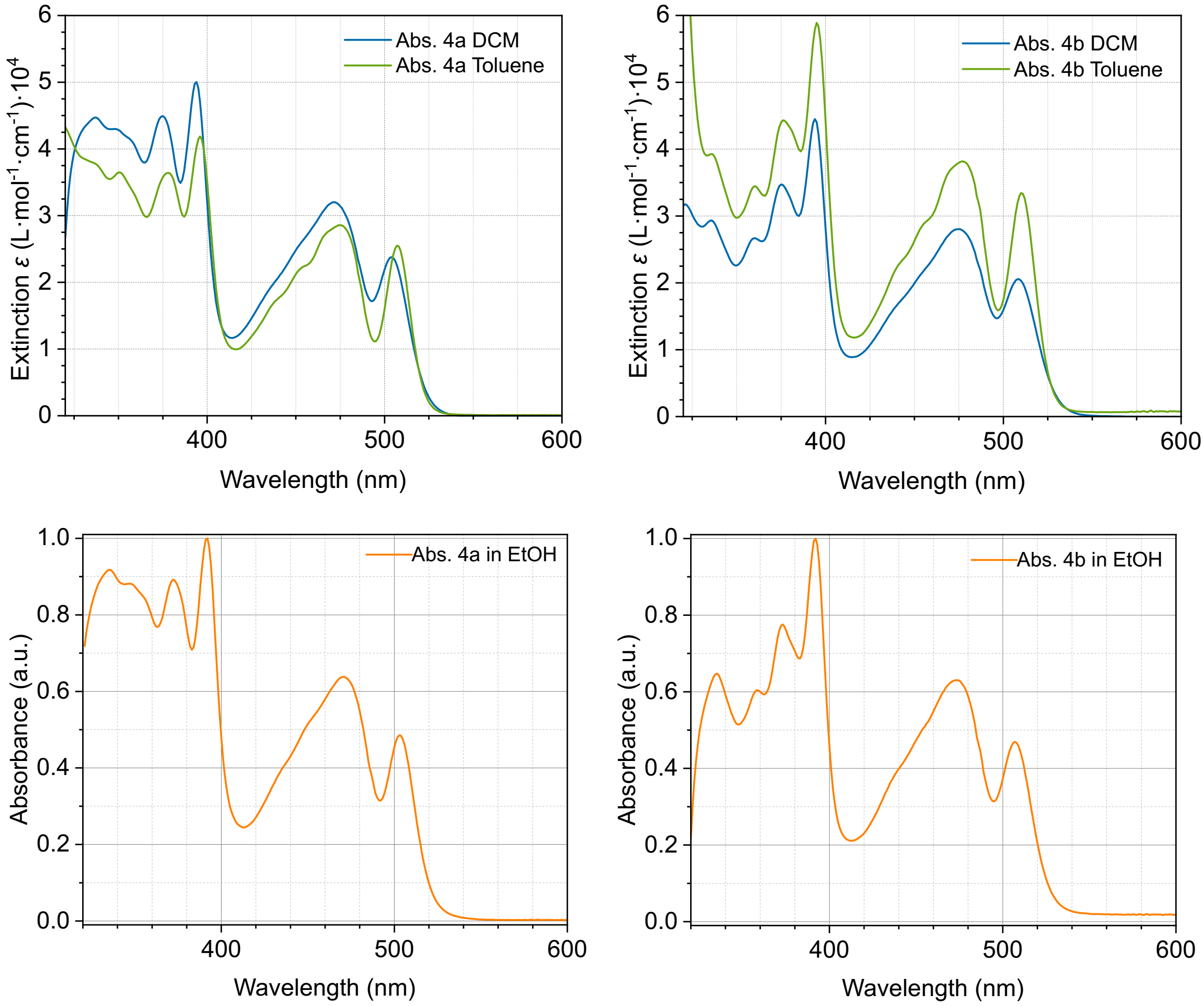


**Figure S14.** Absorption spectra of compound **4a** (left) and **4b** (right) in DCM (blue), EtOH (orange), and toluene (green).

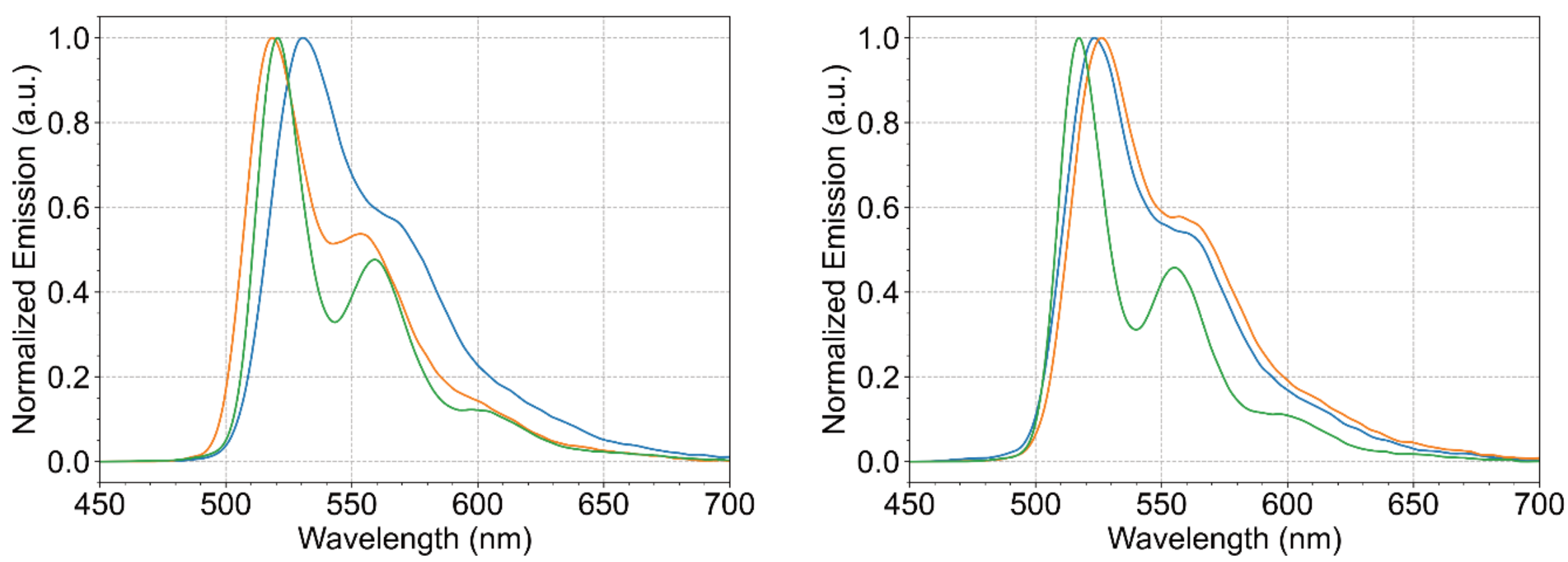


**Figure S15.** Normalized photoluminescence spectra of compounds **4a** (left) and **4b** (right) in DCM (blue), EtOH (orange), and toluene (green). All compounds were excited at 379 nm. The corresponding excitation and emission wavelengths and Stokes shifts are listed in **Table S10** and **S11**.

**Table S10.** Absorption and emission maxima of compound **4a** with Stokes shift calculated from respective absorption and emission maxima marked with asterisk (lowest energy transition, as determined by TD-DFT).

| $E_T^N$ | Solvent | Absorption Maxima (nm) | Emission Maxima (nm) | Stokes Shift (cm$^{-1}$) |
|---|---|---|---|---|
| 0.654 | EtOH | 473*<br>392<br>373<br>359<br>335 | 518*<br>554<br>600 | 1,837 |
| 0.309 | DCM | 471*<br>394<br>375<br>349<br>336 | 531*<br>577 | 2,399 |
| 0.099 | Toluene | 475*<br>396<br>378<br>351<br>337 | 520*<br>560<br>602 | 1,822 |

**Table S11.** Absorption and emission maxima of compound **4b** with Stokes shift calculated from respective absorption and emission maxima marked with asterisk (lowest energy transition, as determined by TD-DFT).

| $E_T^N$ | Solvent | Absorption Maxima (nm) | Emission Maxima (nm) | Stokes Shift (cm$^{-1}$) |
|---|---|---|---|---|
| 0.654 | EtOH | 473*<br>392<br>373<br>348<br>336 | 526*<br>559 | 2,130 |
| 0.309 | DCM | 475*<br>394<br>375<br>360<br>336 | 523*<br>559 | 1,932 |
| 0.099 | Toluene | 477*<br>395<br>376<br>360<br>336 | 517*<br>555<br>599 | 1,622 |

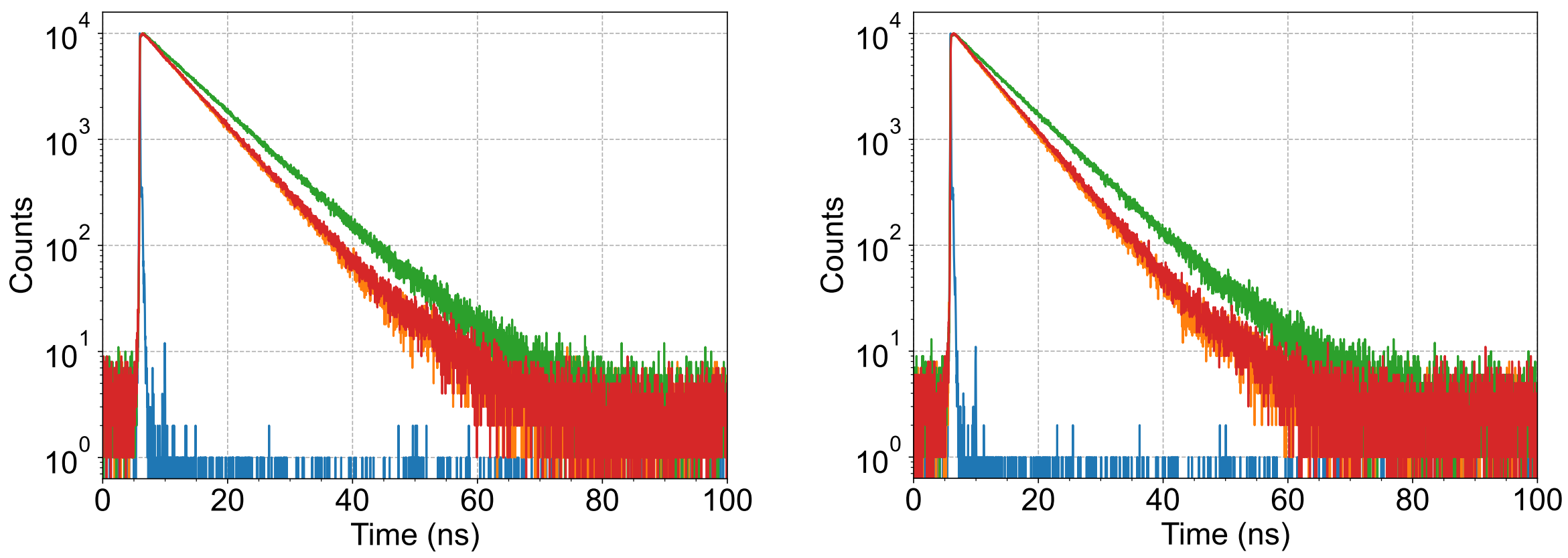


**Figure S16.** Luminescence lifetimes of compound **4a** (left) and **4b** (right) in DCM (orange), EtOH (green), and toluene (red), instrument response function (IRF) in blue. All measurements performed in solutions saturated with argon. Compounds were excited at 375 nm, the emission was measured at the maximum emission of the first fluorescence peak, respectively (s. **Tables S10** and **S11**).

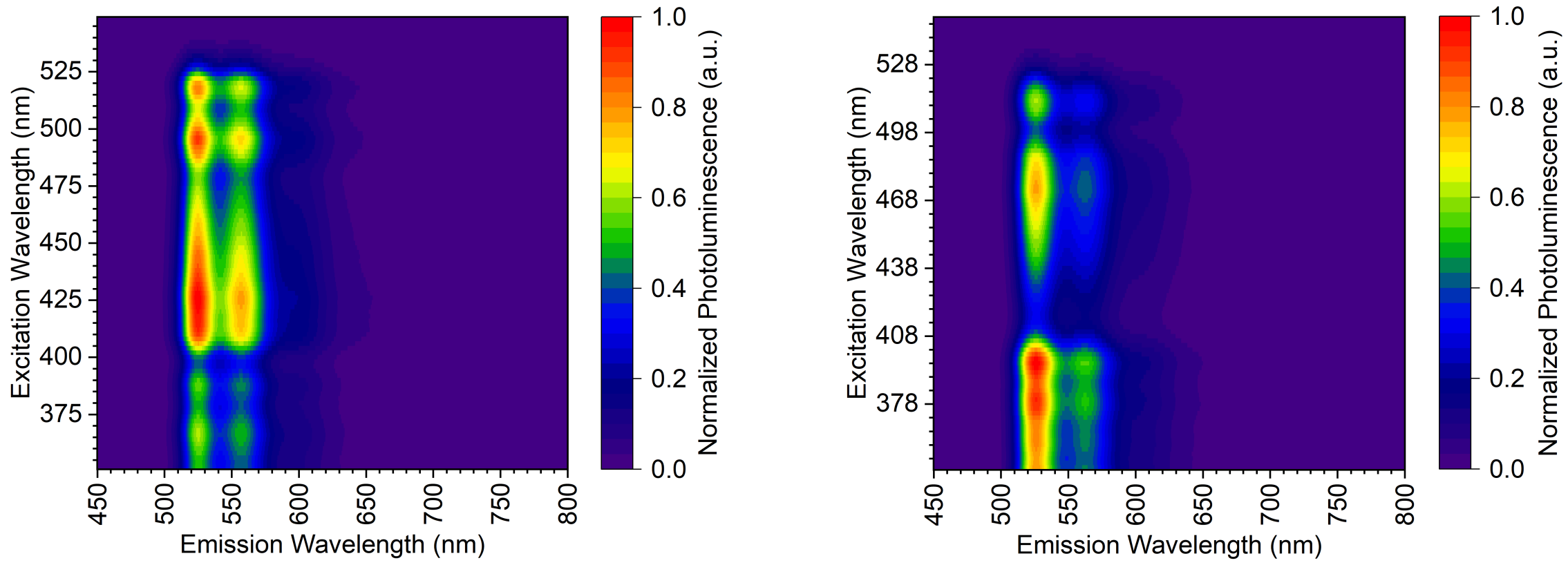


**Figure S17.** Excitation spectra of compounds **4a** (left) and **4b** (right) measured at a concentration of $1{\cdot}10^{-5}$ mol $L^{-1}$ in toluene.

# 8 Organic Light-Emitting Diode (OLED)

## 8.1 Device Fabrication

The OLEDs were produced on prepatterned indium tin oxide (ITO) substrates (Psiotec Ltd.) which were cleaned by sonicating them first in acetone and then in isopropanol. As a last cleaning step and to activate the surface, the substrates were treated with an oxygen plasma for 5 min.

PEDOT:PSS (Al 4083, Ossila) as a hole injection material was spin-coated on the substrates (1,000 rpm, 12 s; 2,000 rpm, 30 s) and annealed at 220 °C for 15 min to form a 50-60 nm thick layer.

As a hole-transport and electron-blocking layer, poly(9,9-dioctylfluorene-*alt*-*N*-(4-*sec*-butylphenyl)-diphenylamine) (TFB) was spin-coated on top of the PEDOT:PSS from a 3 mg/mL toluene solution at 6,000 rpm for 45 s. The film was dried in a vacuum oven at 200 °C for 1 h.

The emitter layer was spin coated from 3 mg/mL solutions of either molecule **4a** or **4b** in chloroform, sometimes with the addition of a host material, as indicated in **Table S12**. Pinhole-free films of 50-60 nm thickness were achieved with a rotation speed of 4,000 rpm for 30 s.

The top contact consisting of a 5 nm calcium and 200 nm aluminum layer was fabricated via thermal evaporation. A design with eight pixels with an active area of 0.04 $cm^2$ each was created using a shadow mask.

The devices were encapsulated with UV-curable resin (Ossila) and glass coverslips.

## 8.2 Device Performance

**Table S12.** Device data for selected fabricated OLEDs. A list of all fabricated OLEDs is provided within the raw data. The luminous power efficiency is calculated by considering the OLED as a Lambertian source, that emits into the half plane.[24, 25]

| No. | Device stack | luminance maximum | Average luminance | turn-on | current efficacy | luminous power efficiency | CIE x | CIE y |
|---|---|---|---|---|---|---|---|---|
| 1 | ITO/PEDOT:PSS/**4a**/Ca/Al | 1,620 cd·m$^{-2}$ | 1,390 cd·m$^{-2}$ | 3 V | | 0.10 lm/W | | |
| 2 | ITO/PEDOT:PSS/**4b**/Ca/Al | 2,280 cd·m$^{-2}$ | 2,280 cd·m$^{-2}$ | 3 V | | 0.07 lm/W | | |
| 3 | ITO/PEDOT:PSS/**4a**/Ca/Al | 1,690 cd·m$^{-2}$ | 1,580 cd·m$^{-2}$ | 3.5 V | 0.03 cd·A$^{-1}$ | 0.11 lm/W | 0.47 | 0.52 |
| 4 | ITO/PEDOT:PSS/**4b**/Ca/Al | 2,390 cd·m$^{-2}$ | 2,170 cd·m$^{-2}$ | 3 V | 0.11 cd·A$^{-1}$ | 0.07 lm/W | 0.48 | 0.51 |
| 5 | ITO/PEDOT:PSS/0.5 wt% **4a** in TCTA/Ca/Al | 2,110 cd·m$^{-2}$ | 2,040 cd·m$^{-2}$ | 4 V | 0.44 cd·A$^{-1}$ | 0.65 lm/W | | |
| 6 | ITO/PEDOT:PSS/1 wt% **4a** in TCTA/Ca/Al | 3,120 cd·m$^{-2}$ | 3,060 cd·m$^{-2}$ | 4 V | 0.53 cd·A$^{-1}$ | 0.82 lm/W | 0.32 | 0.62 |
| 7 | ITO/PEDOT:PSS/2.5 wt% **4a** in TCTA/Ca/Al | 3,150 cd·m$^{-2}$ | 3,040 cd·m$^{-2}$ | 4 V | 0.51 cd·A$^{-1}$ | 0.41 lm/W | | |
| 8 | ITO/PEDOT:PSS/5 wt% **4a** in TCTA/Ca/Al | 1,530 cd·m$^{-2}$ | 1,400 cd·m$^{-2}$ | 4 V | 0.30 cd·A$^{-1}$ | 0.23 lm/W | 0.35 | 0.60 |
| 9 | ITO/PEDOT:PSS/10 wt% **4a** in TCTA/Ca/Al | 552 cd·m$^{-2}$ | 520 cd·m$^{-2}$ | 5 V | 0.11 cd·A$^{-1}$ | 0.15 lm/W | | |
| 10 | ITO/PEDOT:PSS/20 wt% **4a** in TCTA/Ca/Al | 1,590 cd·m$^{-2}$ | 1,550 cd·m$^{-2}$ | 4 V | 0.14 cd·A$^{-1}$ | 0.05 lm/W | | |
| 11 | ITO/PEDOT:PSS/0.5 wt% **4b** in TCTA/Ca/Al | 3,050 cd·m$^{-2}$ | 2,880 cd·m$^{-2}$ | 4 V | 0.60 cd·A$^{-1}$ | 0.79 lm/W | | |
| 12 | ITO/PEDOT:PSS/1 wt% **4b** in TCTA/Ca/Al | 3,710 cd·m$^{-2}$ | 3,390 cd·m$^{-2}$ | 4 V | 0.56 cd·A$^{-1}$ | 0.60 lm/W | 0.35 | 0.60 |
| 13 | ITO/PEDOT:PSS/5 wt% **4b** in TCTA/Ca/Al | 2,270 cd·m$^{-2}$ | 1,990 cd·m$^{-2}$ | 4 V | 0.21 cd·A$^{-1}$ | 0.16 lm/W | 0.36 | 0.61 |
| 14 | ITO/PEDOT:PSS/10 wt% **4b** in TCTA/Ca/Al | 670 cd·m$^{-2}$ | 560 cd·m$^{-2}$ | 4 V | 0.06 cd·A$^{-1}$ | 0.01 lm/W | 0.34 | 0.57 |
| 15 | ITO/PEDOT:PSS/20 wt% **4b** in TCTA/Ca/Al | 1,780 cd·m$^{-2}$ | 1,640 cd·m$^{-2}$ | 4 V | 0.17 cd·A$^{-1}$ | 0.17 lm/W | 0.41 | 0.57 |
| 16 | ITO/PEDOT:PSS/TFB/0.5 wt% **4a** in TCTA/Ca/Al | 6,880 cd·m$^{-2}$ | 6,520 cd·m$^{-2}$ | 5 V | 1.31 cd·A$^{-1}$ | 0.91 lm/W | 0.33 | 0.61 |
| 17 | ITO/PEDOT:PSS/TFB/1 wt% **4a** in TCTA/Ca/Al | 5,880 cd·m$^{-2}$ | 5,710 cd·m$^{-2}$ | 5 V | 0.95 cd·A$^{-1}$ | 0.44 lm/W | | |
| 18 | ITO/PEDOT:PSS/TFB/2.5 wt% **4a** in TCTA/Ca/Al | 3,250 cd·m$^{-2}$ | 2,990 cd·m$^{-2}$ | 5 V | 0.38 cd·A$^{-1}$ | 0.29 lm/W | | |
| 19 | ITO/PEDOT:PSS/TFB/0.5 wt% **4b** in TCTA/Ca/Al | 6,740 cd·m$^{-2}$ | 6,310 cd·m$^{-2}$ | 5 V | 1.10 cd·A$^{-1}$ | 0.76 lm/W | 0.34 | 0.58 |
| 20 | ITO/PEDOT:PSS/TFB/1 wt% **4b** in TCTA/Ca/Al | 5,770 cd·m$^{-2}$ | 5,230 cd·m$^{-2}$ | 5 V | 0.83 cd·A$^{-1}$ | 0.72 lm/W | | |
| 21 | ITO/PEDOT:PSS/TFB/2.5 wt% **4b** in TCTA/Ca/Al | 2,470 cd·m$^{-2}$ | 2,230 cd·m$^{-2}$ | 5 V | 0.28 cd·A$^{-1}$ | 0.14 lm/W | | |

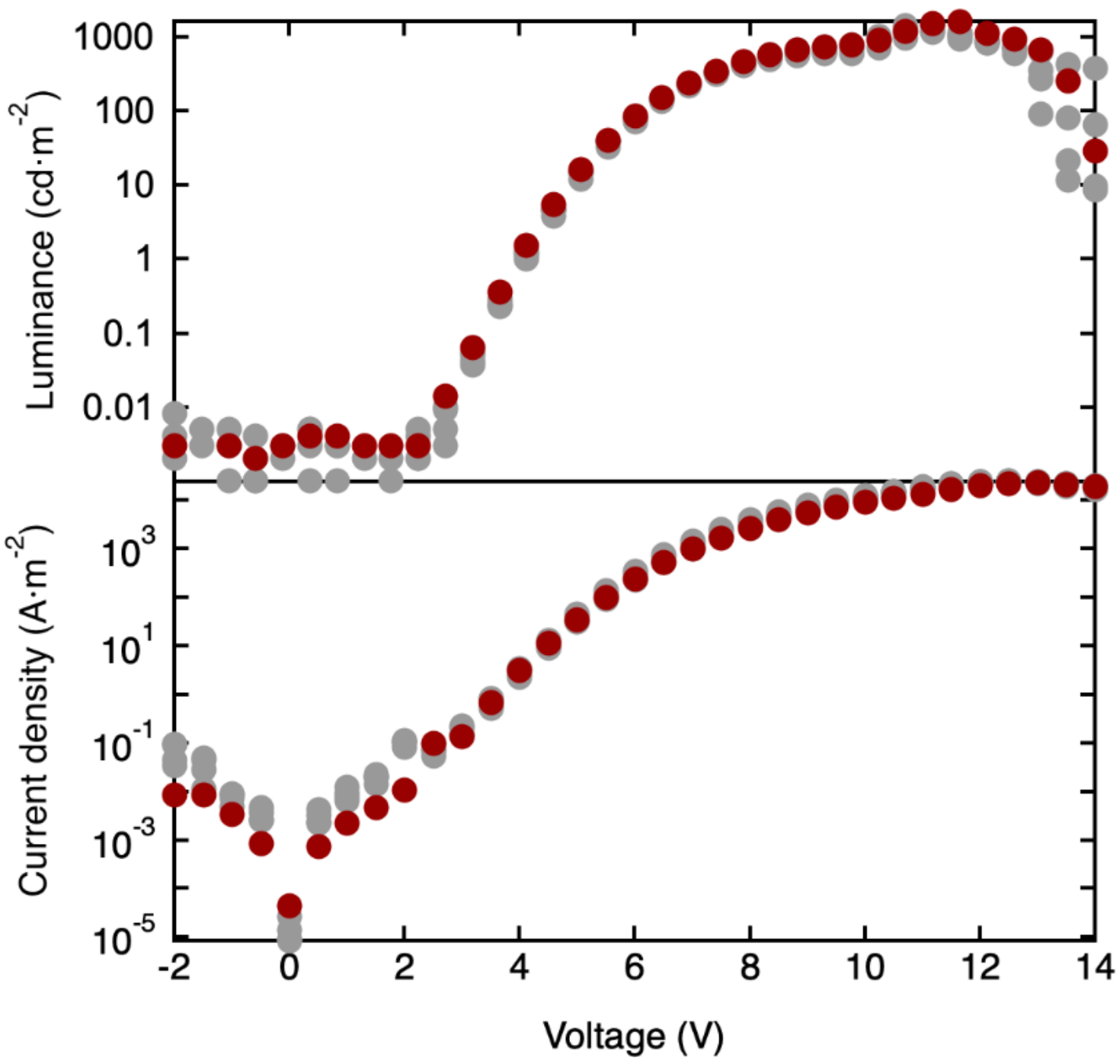


**Figure S18.** JVL characteristics of OLED 1, luminance (top) and current density (bottom). The brightest pixel is displayed in red.

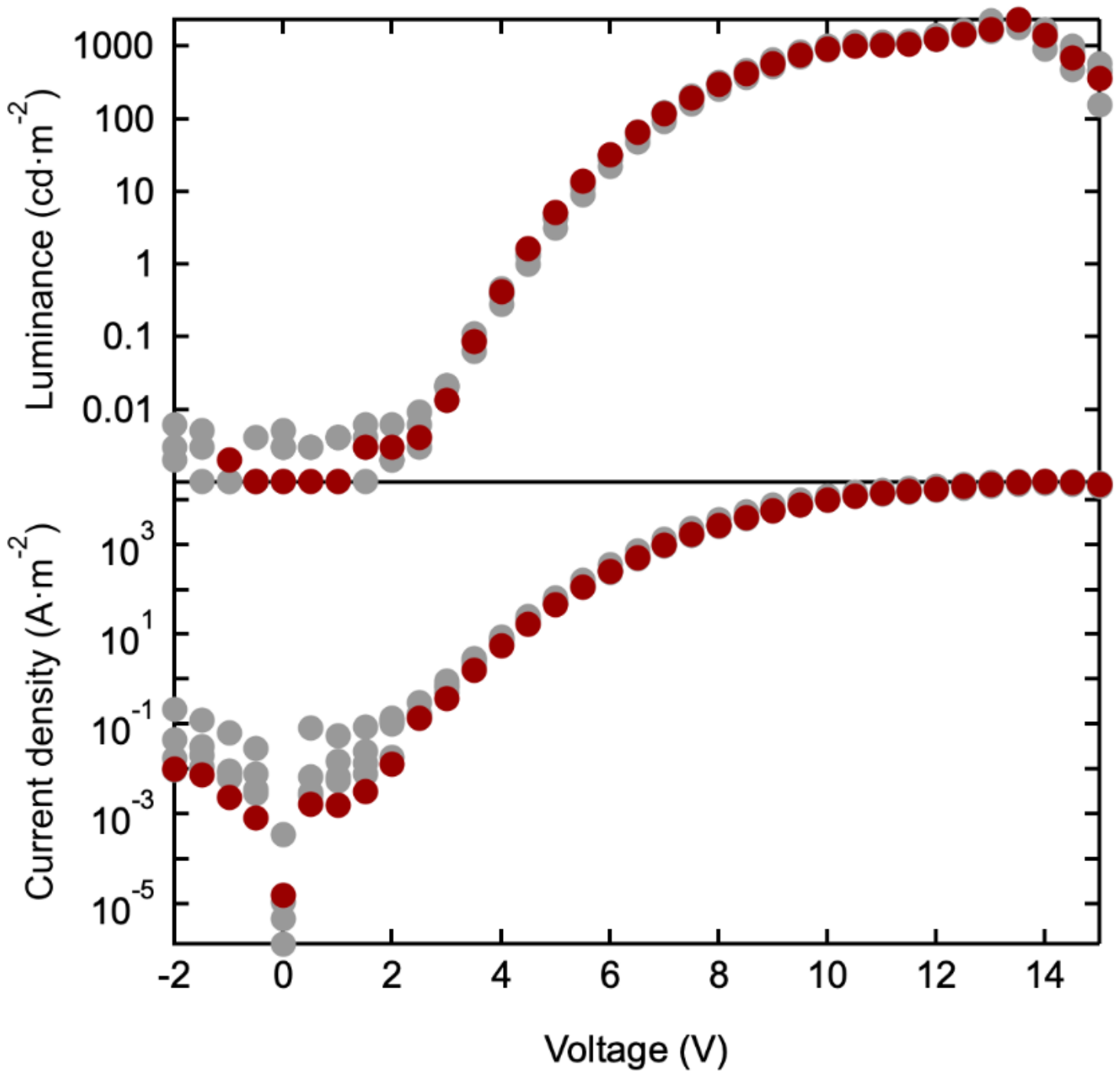


**Figure S19.** JVL characteristics of OLED 2, luminance (top) and current density (bottom). The brightest pixel is displayed in red.

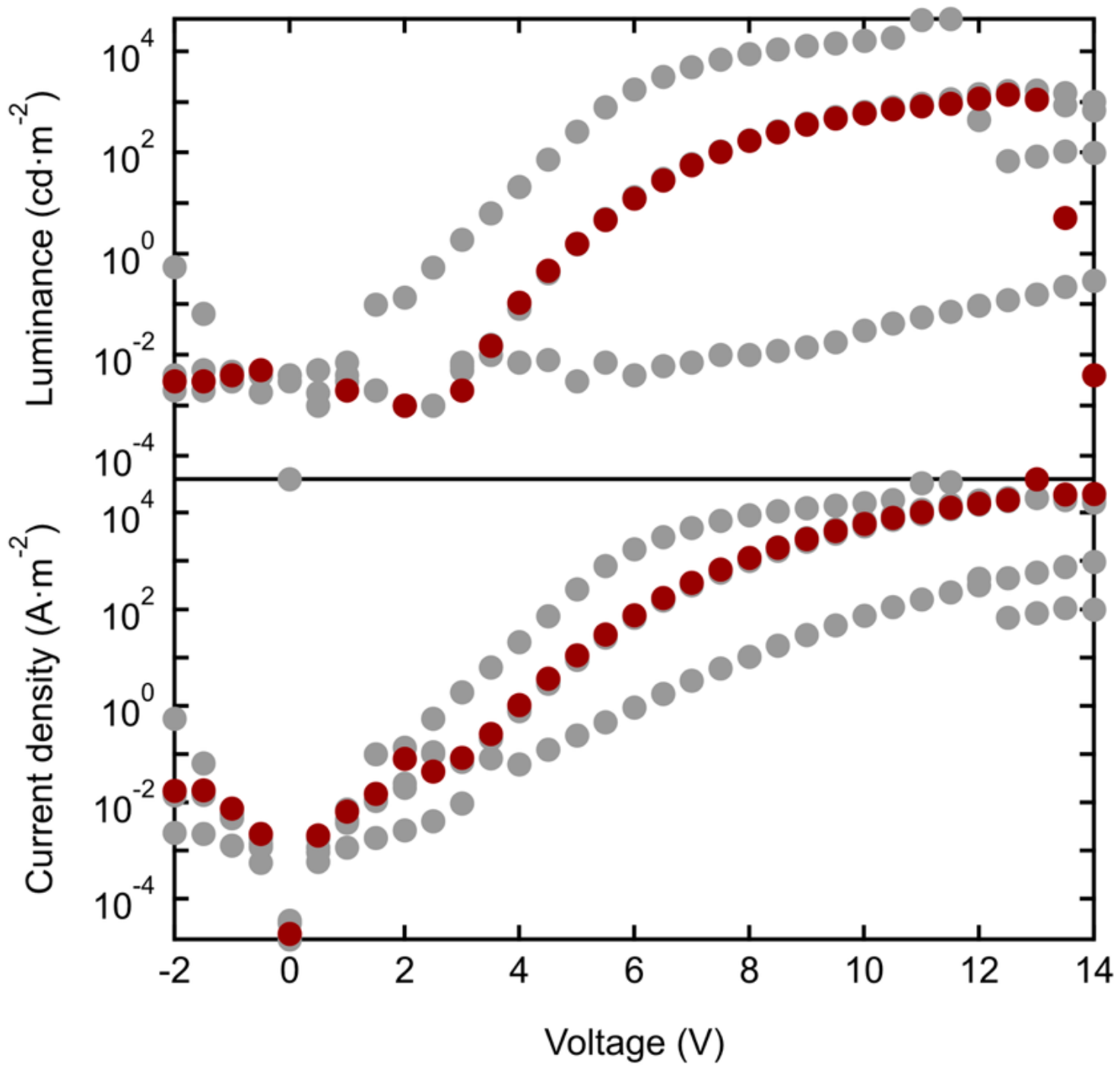


**Figure S20.** JVL characteristics of OLED 3, luminance (top) and current density (bottom). The brightest pixel is displayed in red.

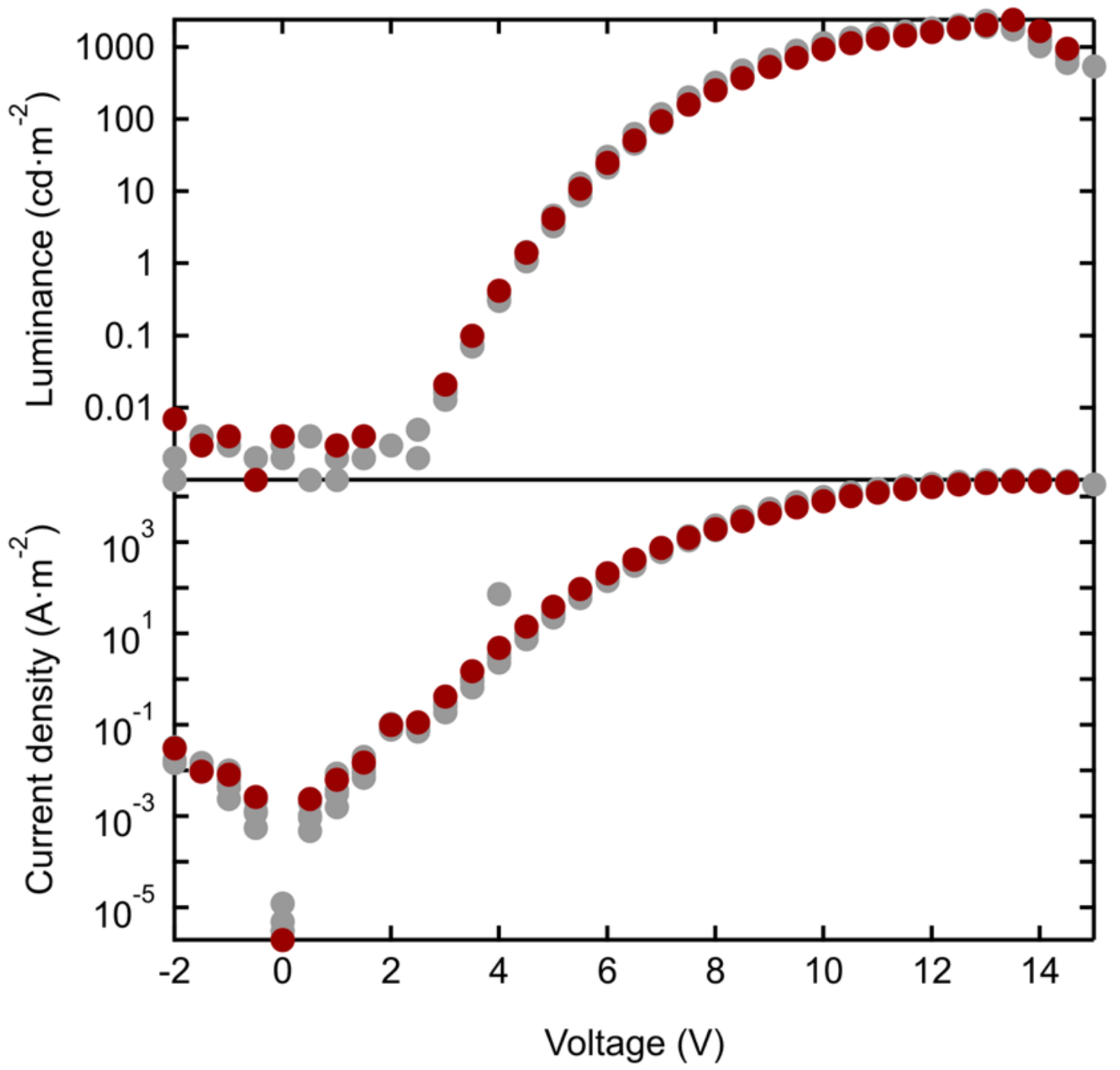


**Figure S21.** JVL characteristics of OLED 4, luminance (top) and current density (bottom). The brightest pixel is displayed in red.

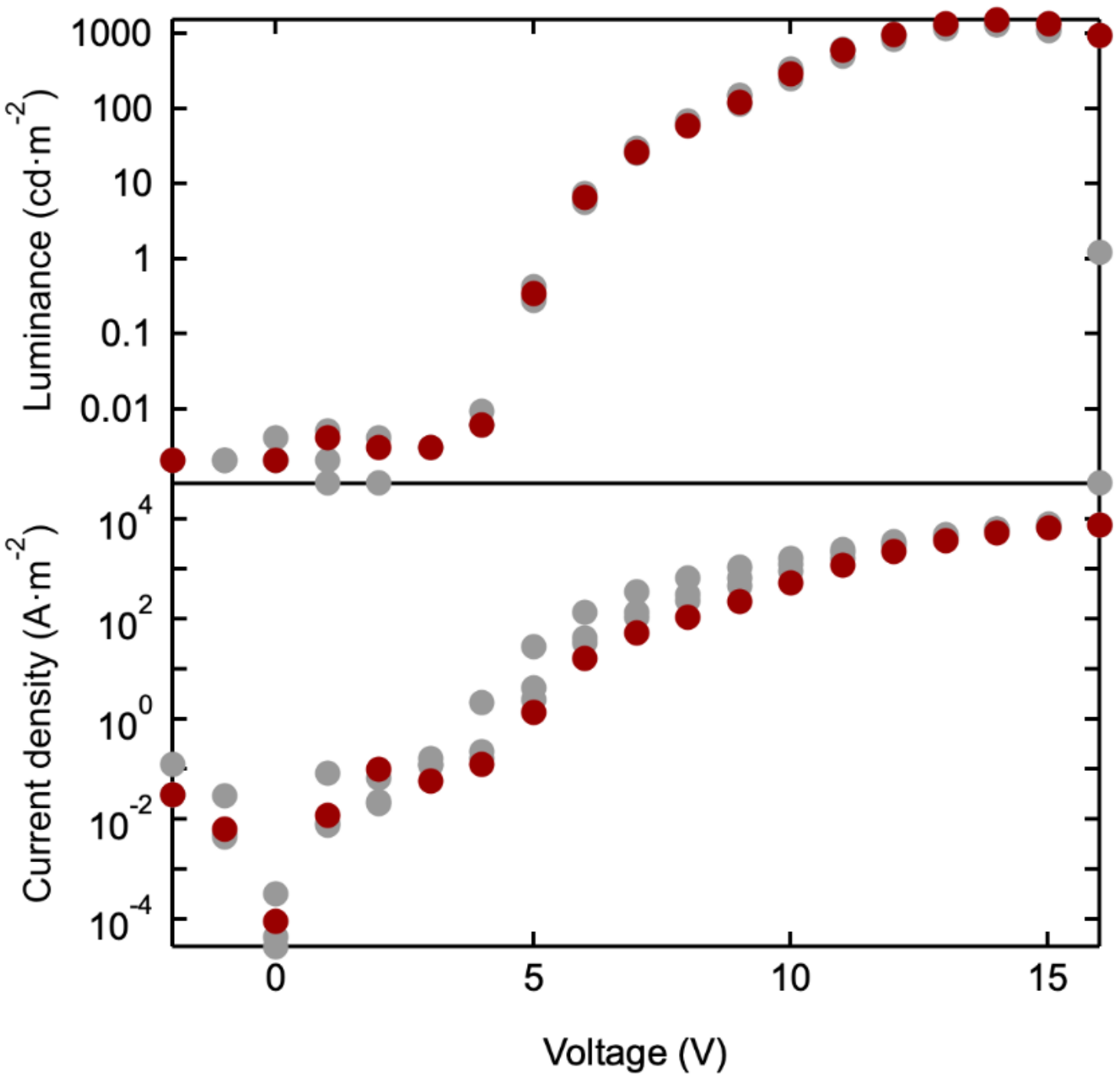


**Figure S22.** JVL characteristics of OLED 8, luminance (top) and current density (bottom). The brightest pixel is displayed in red.

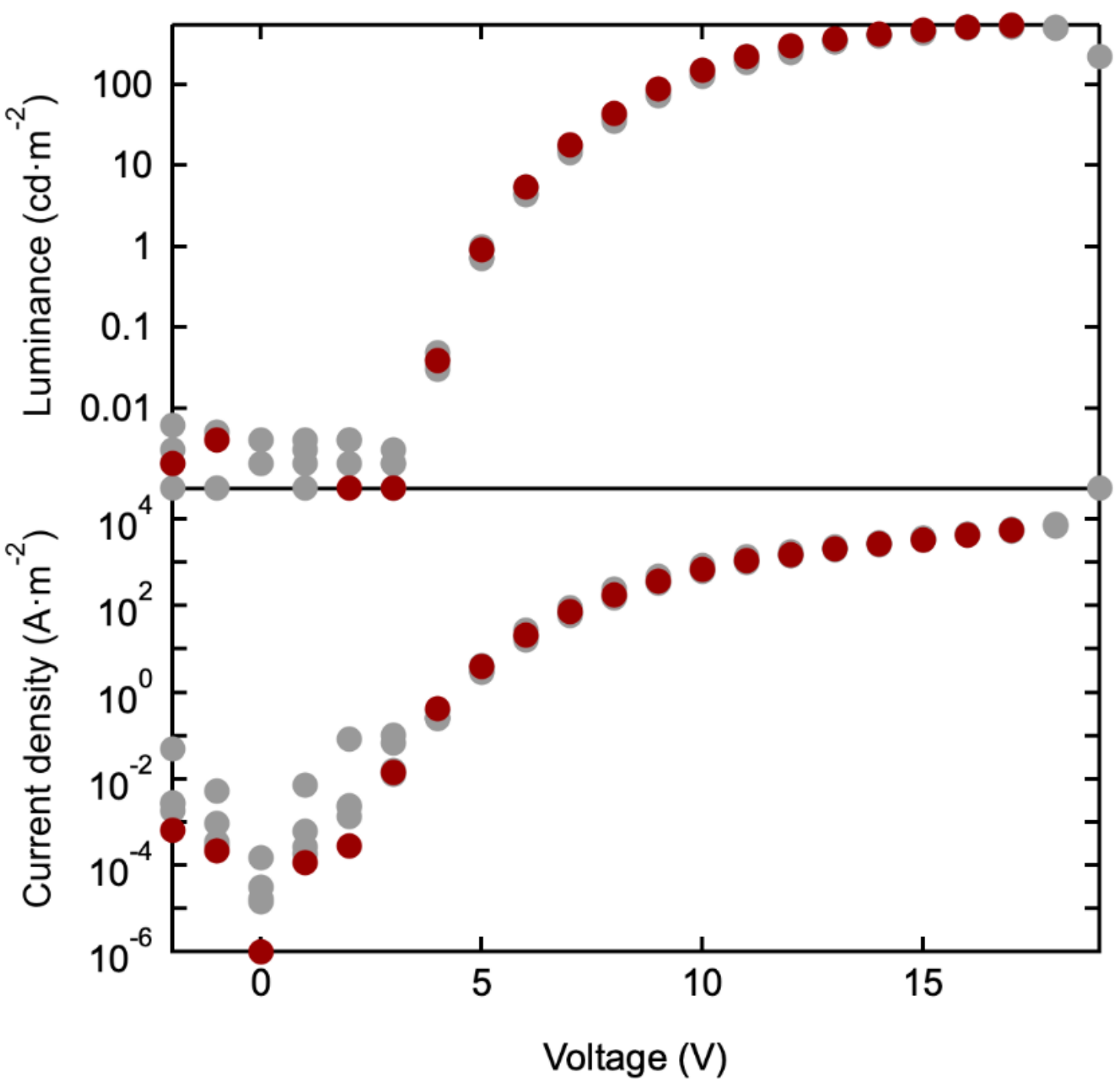


**Figure S23.** JVL characteristics of OLED 9, luminance (top) and current density (bottom). The brightest pixel is displayed in red.

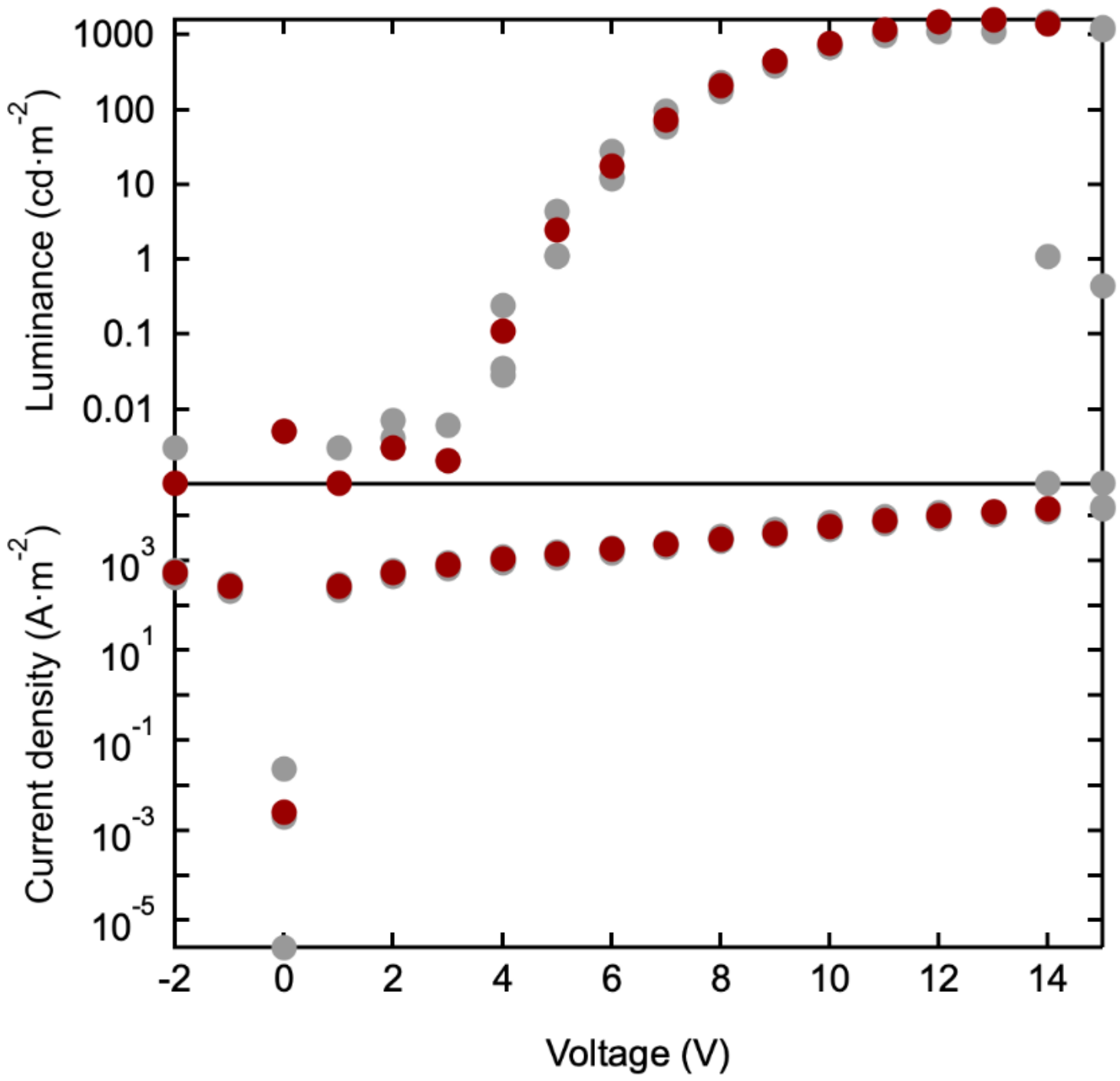


**Figure S24.** JVL characteristics of OLED 10, luminance (top) and current density (bottom). The brightest pixel is displayed in red.

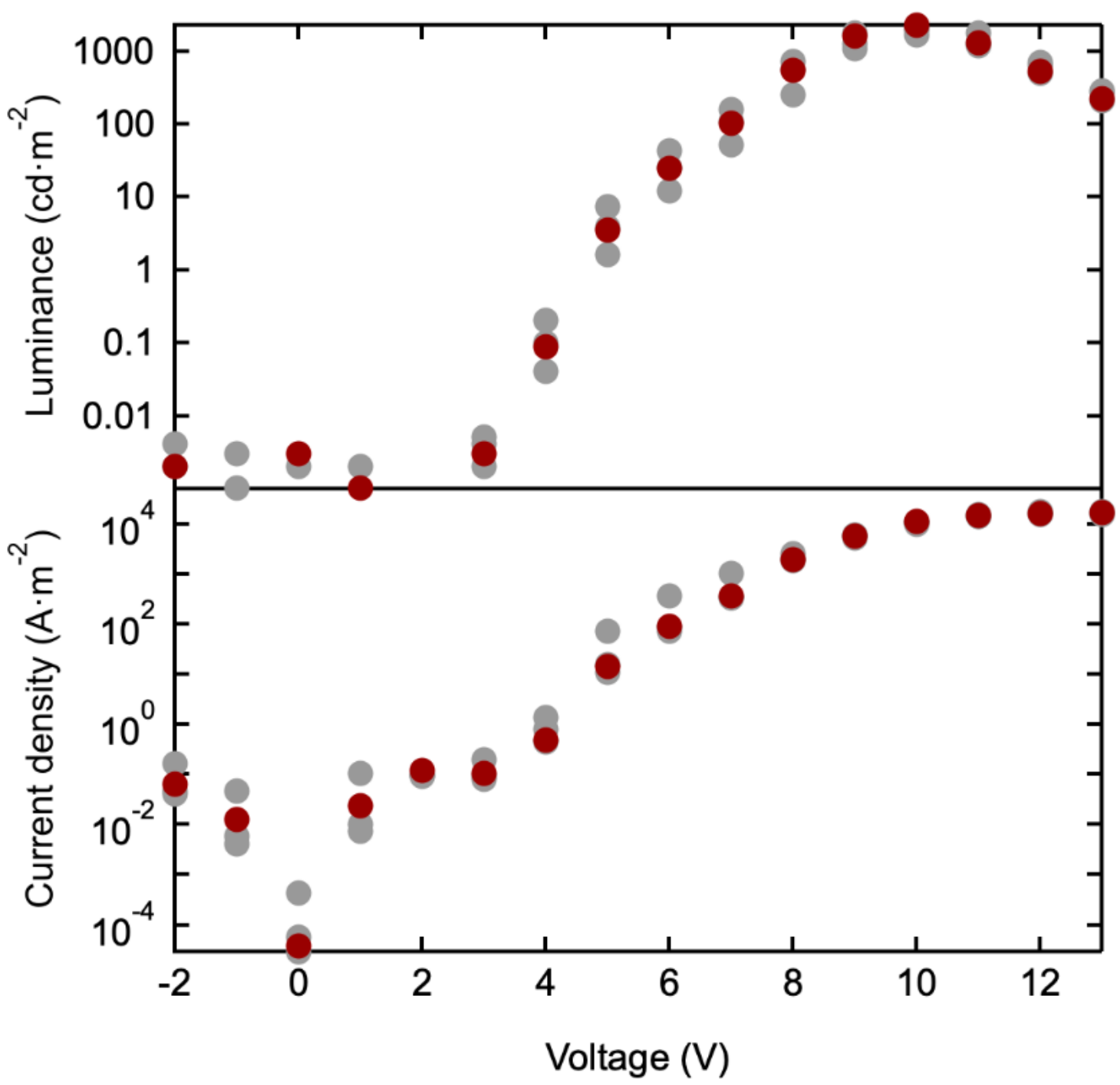


**Figure S25.** JVL characteristics of OLED 13, luminance (top) and current density (bottom). The brightest pixel is displayed in red.

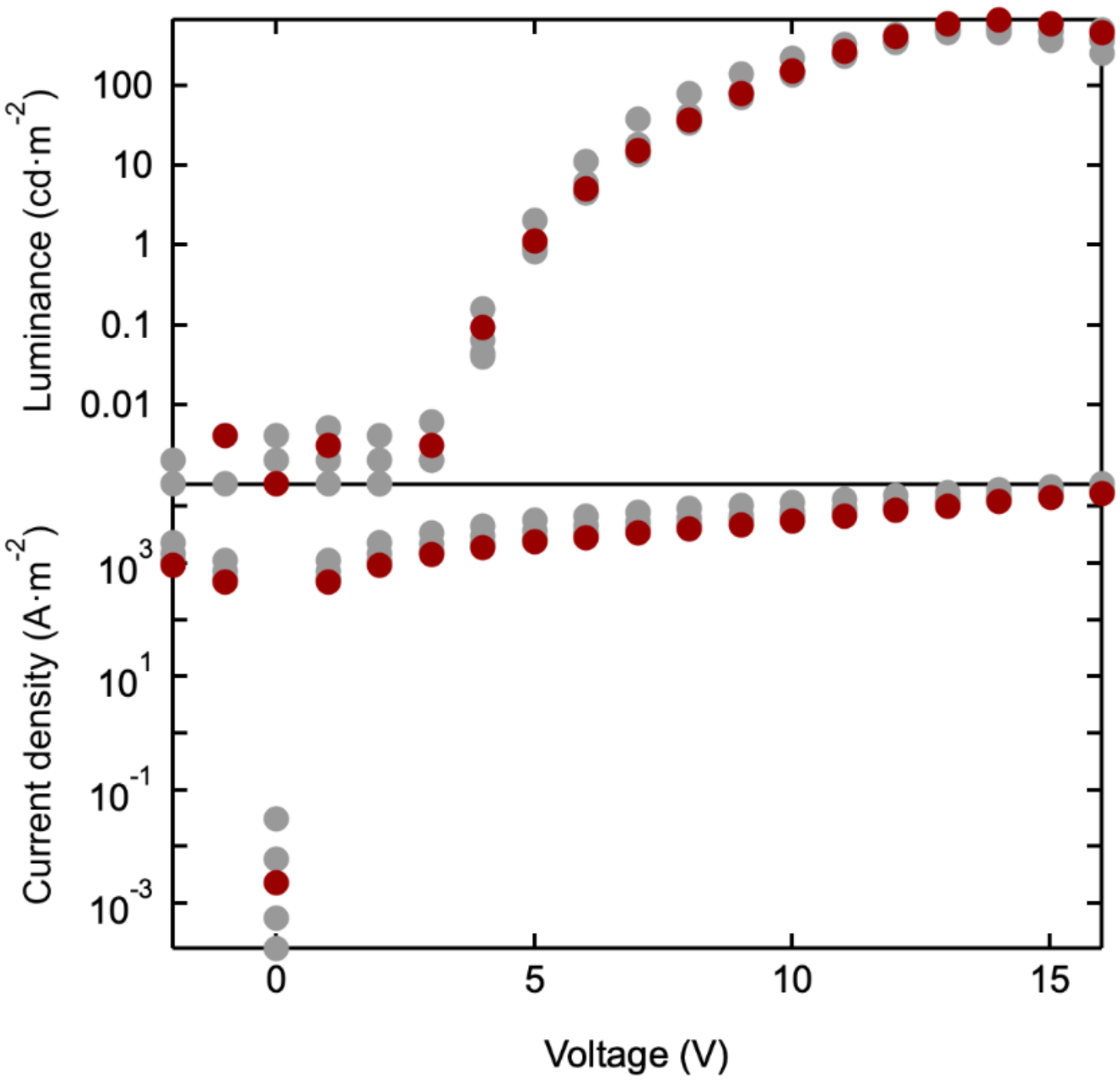


**Figure S26.** JVL characteristics of OLED 14, luminance (top) and current density (bottom). The brightest pixel is displayed in red.

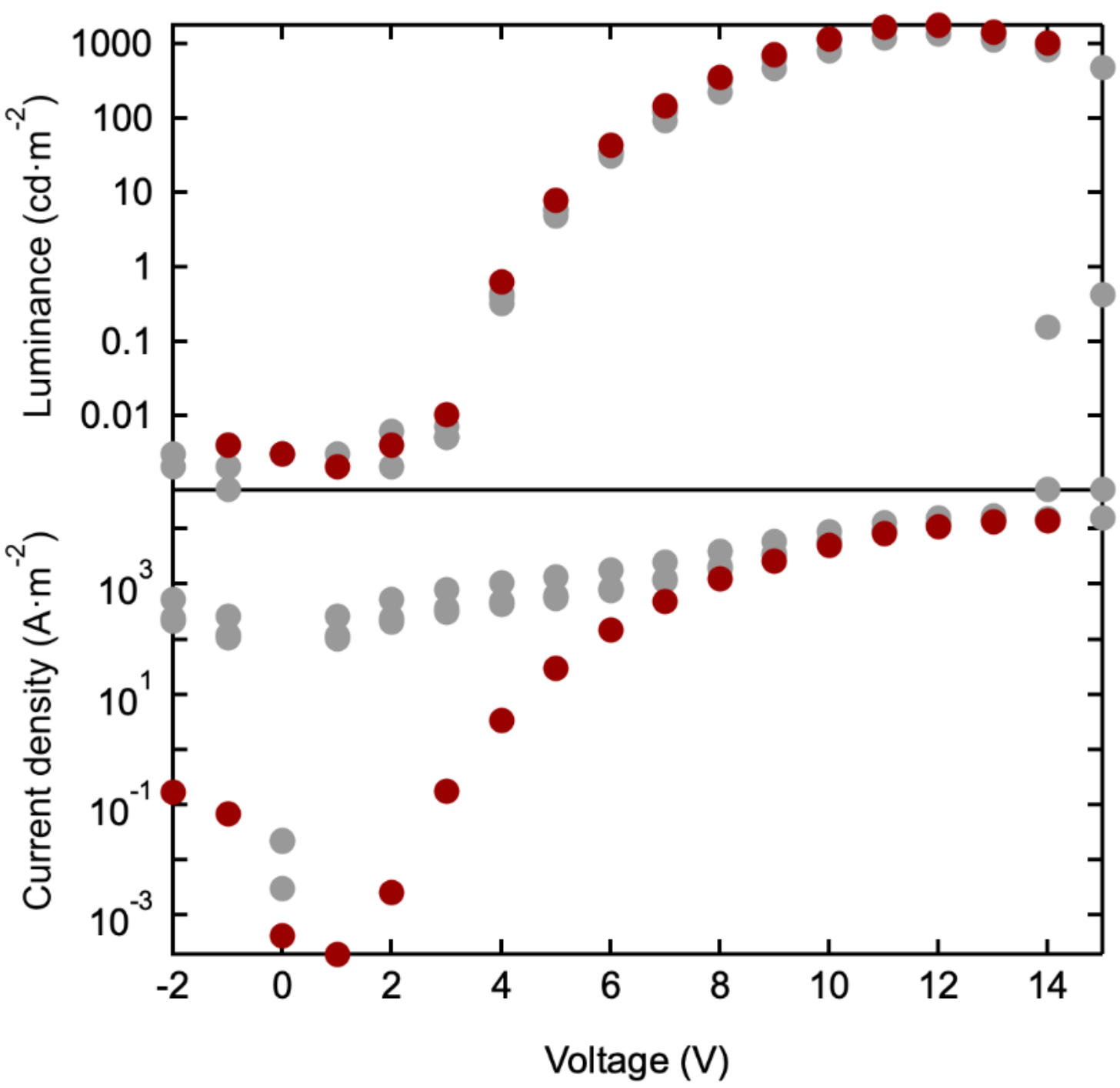


**Figure S27.** JVL characteristics of OLED 15, luminance (top) and current density (bottom). The brightest pixel is displayed in red.

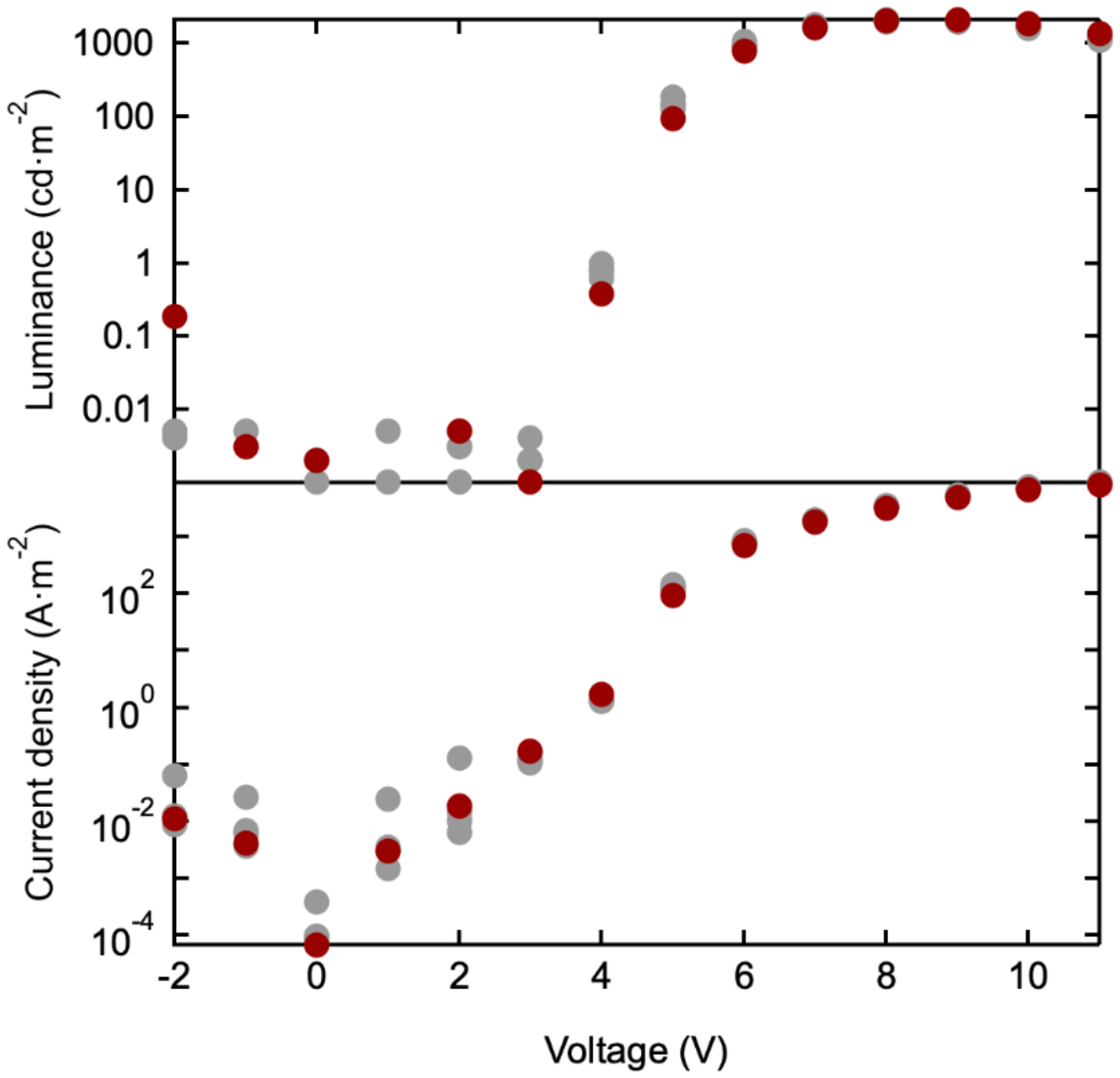


**Figure S28.** JVL characteristics of OLED 5, luminance (top) and current density (bottom). The brightest pixel is displayed in red.

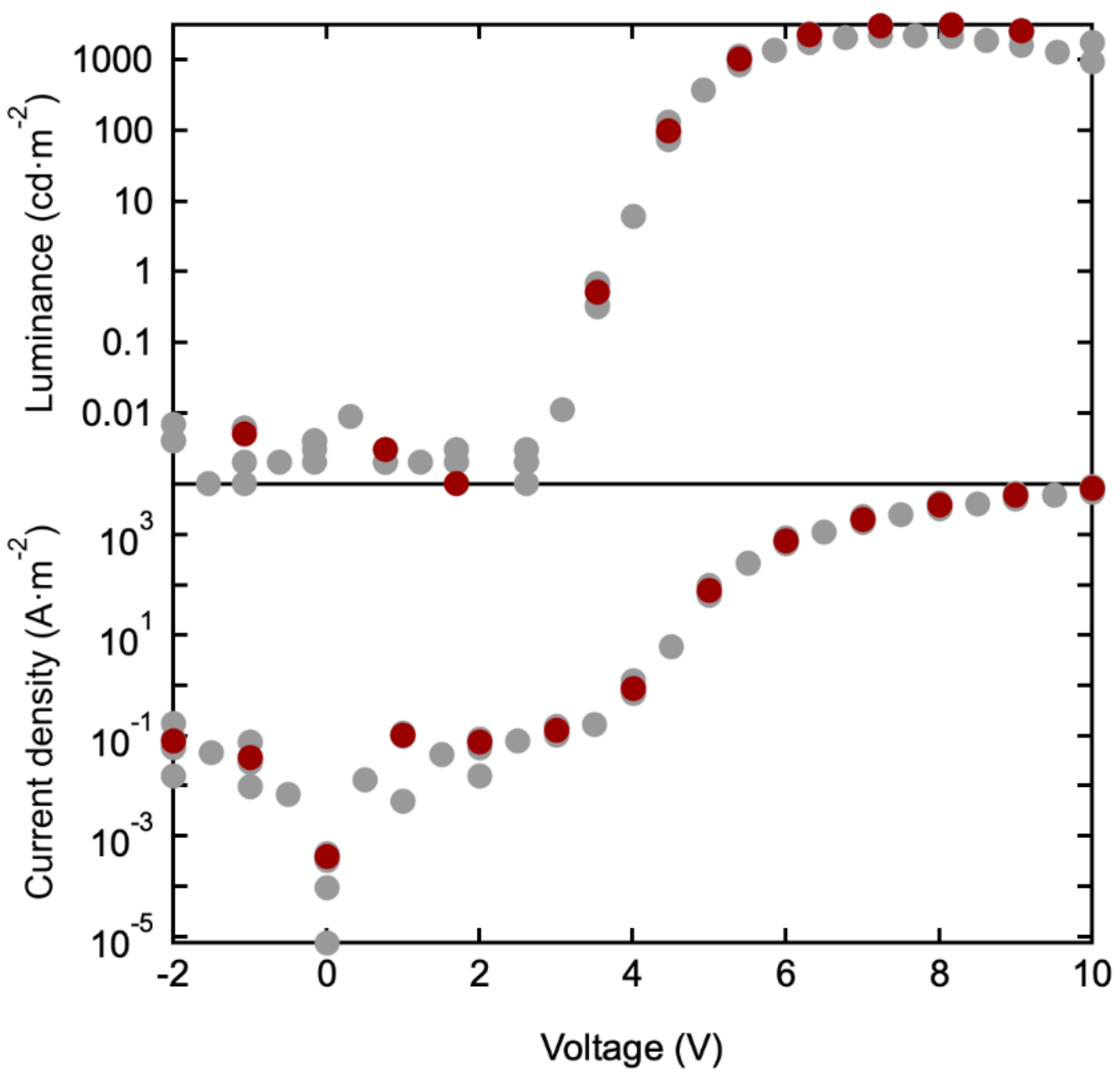


**Figure S29.** JVL characteristics of OLED 6, luminance (top) and current density (bottom). The brightest pixel is displayed in red.

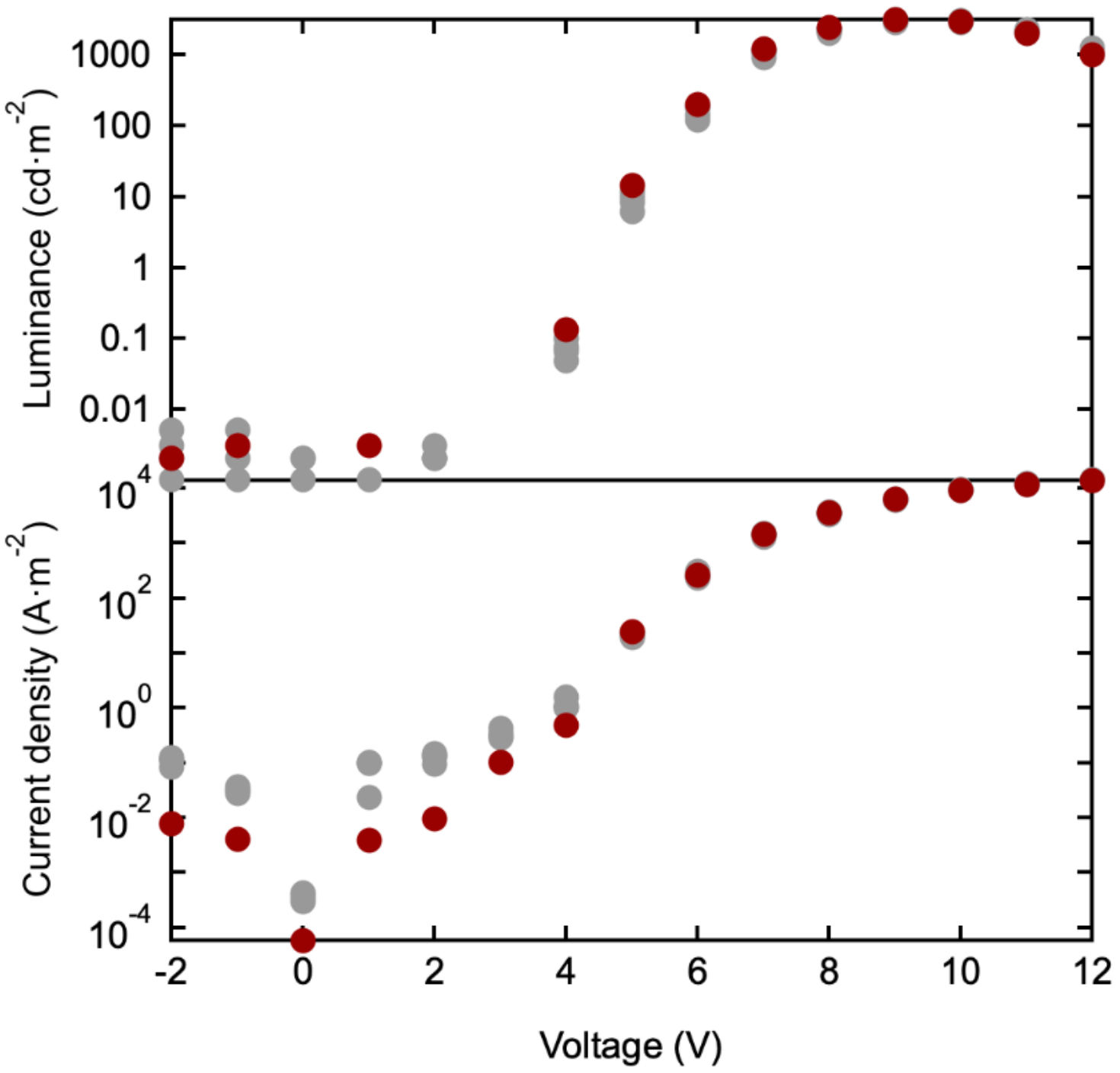


**Figure S30.** JVL characteristics of OLED 7, luminance (top) and current density (bottom). The brightest pixel is displayed in red.

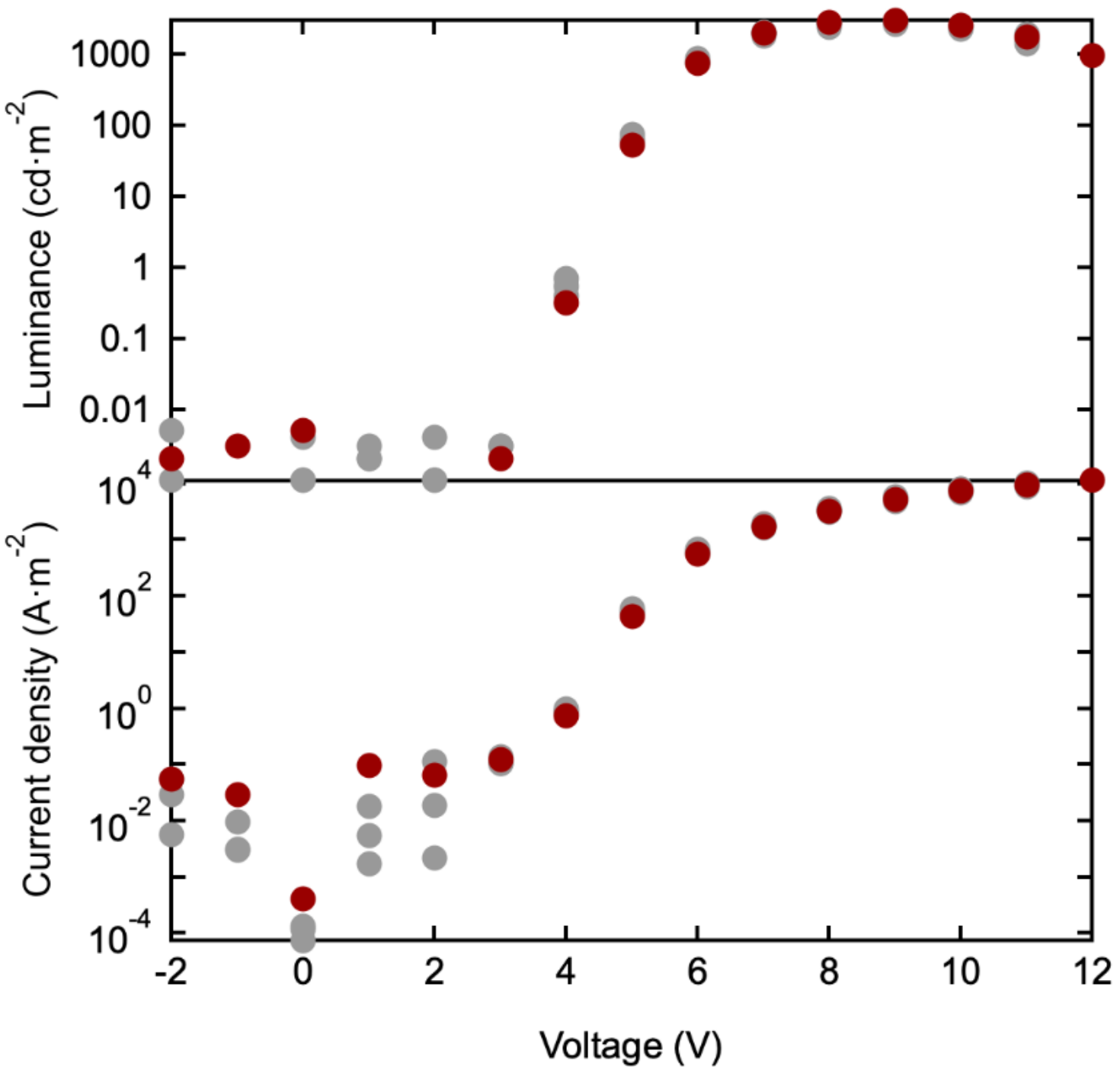


**Figure S31.** JVL characteristics of OLED 11, luminance (top) and current density (bottom). The brightest pixel is displayed in red.

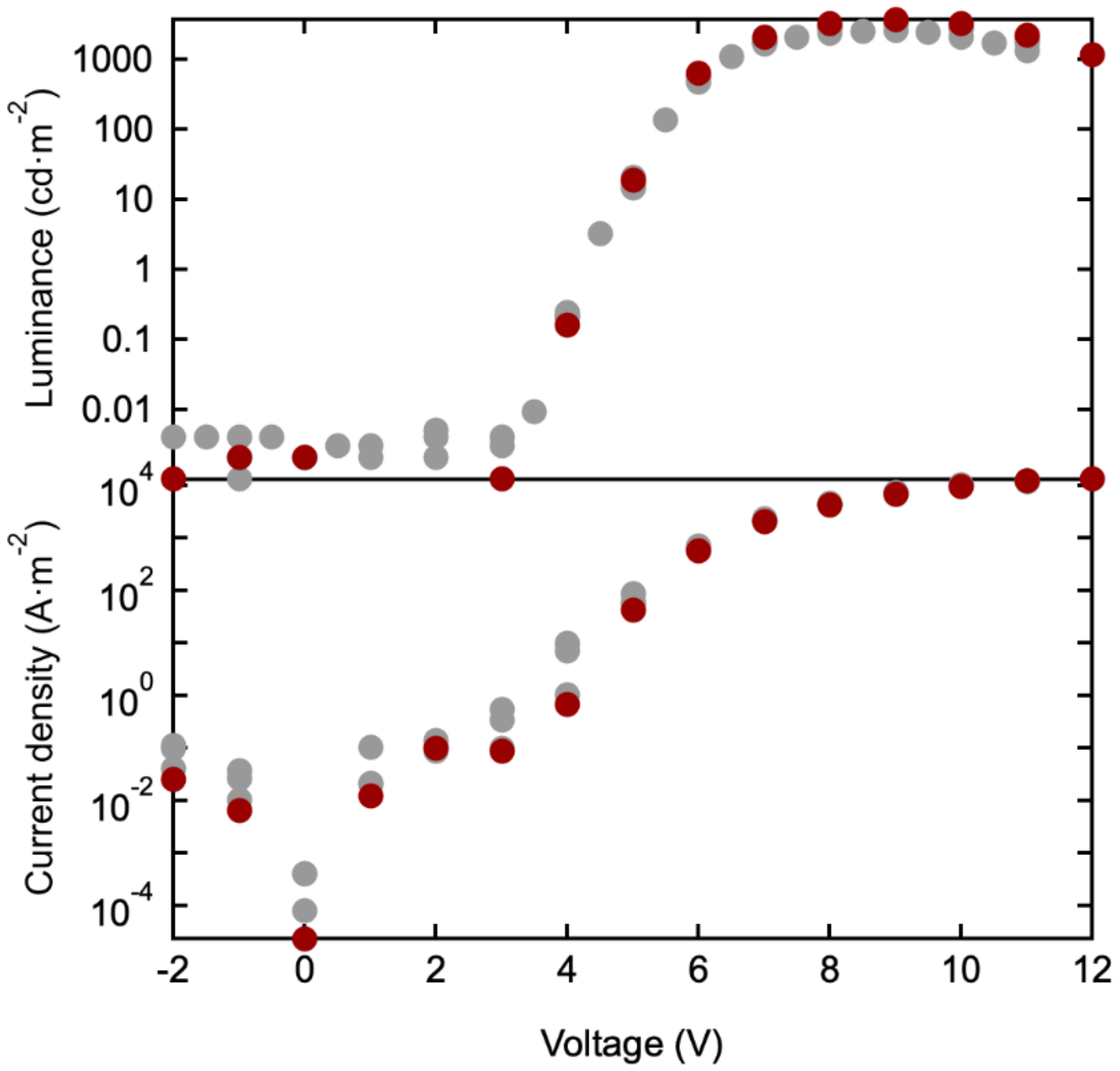


**Figure S32.** JVL characteristics of OLED 12, luminance (top) and current density (bottom). The brightest pixel is displayed in red.

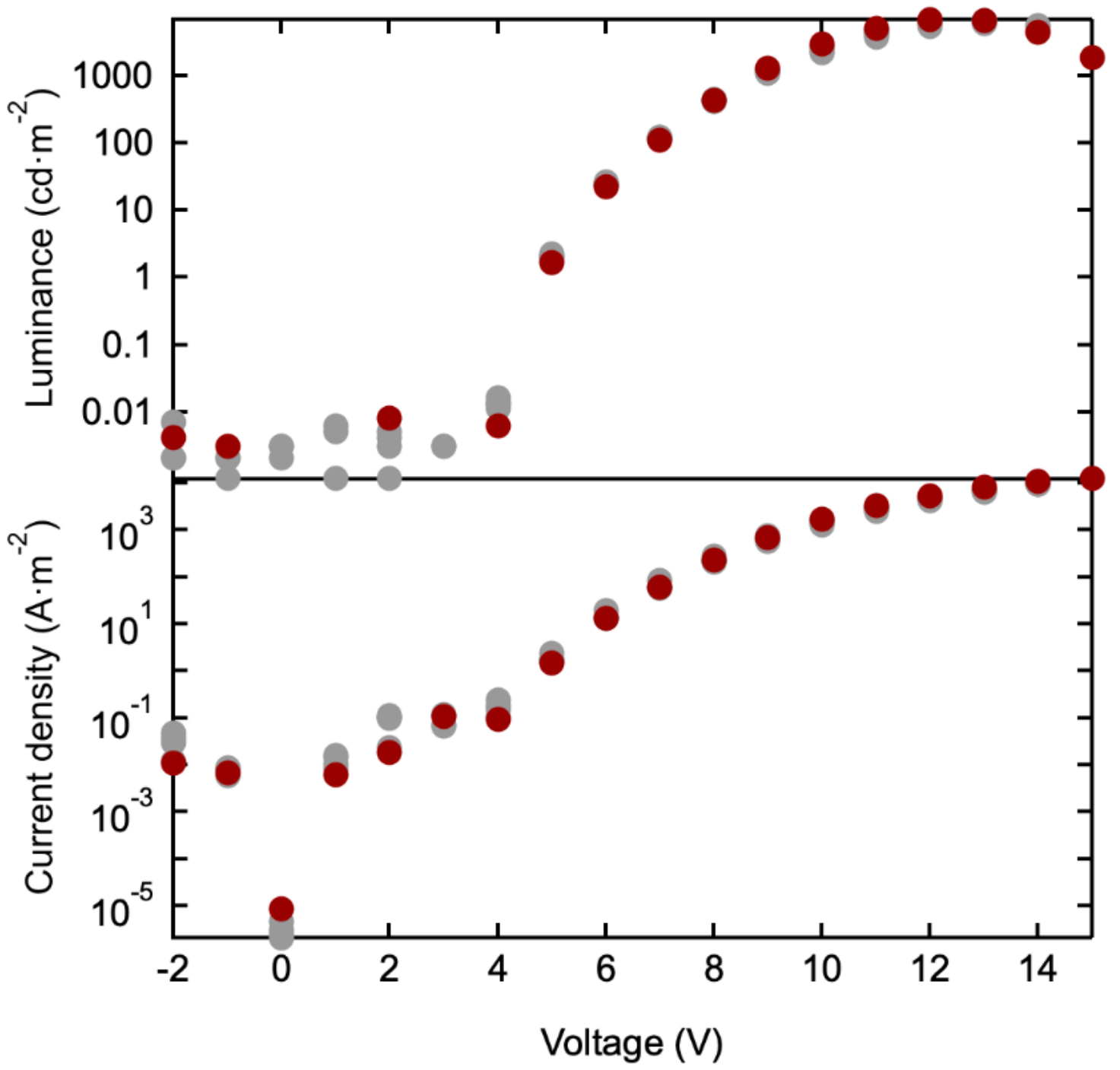


**Figure S33.** JVL characteristics of OLED 16, luminance (top) and current density (bottom). The brightest pixel is displayed in red.

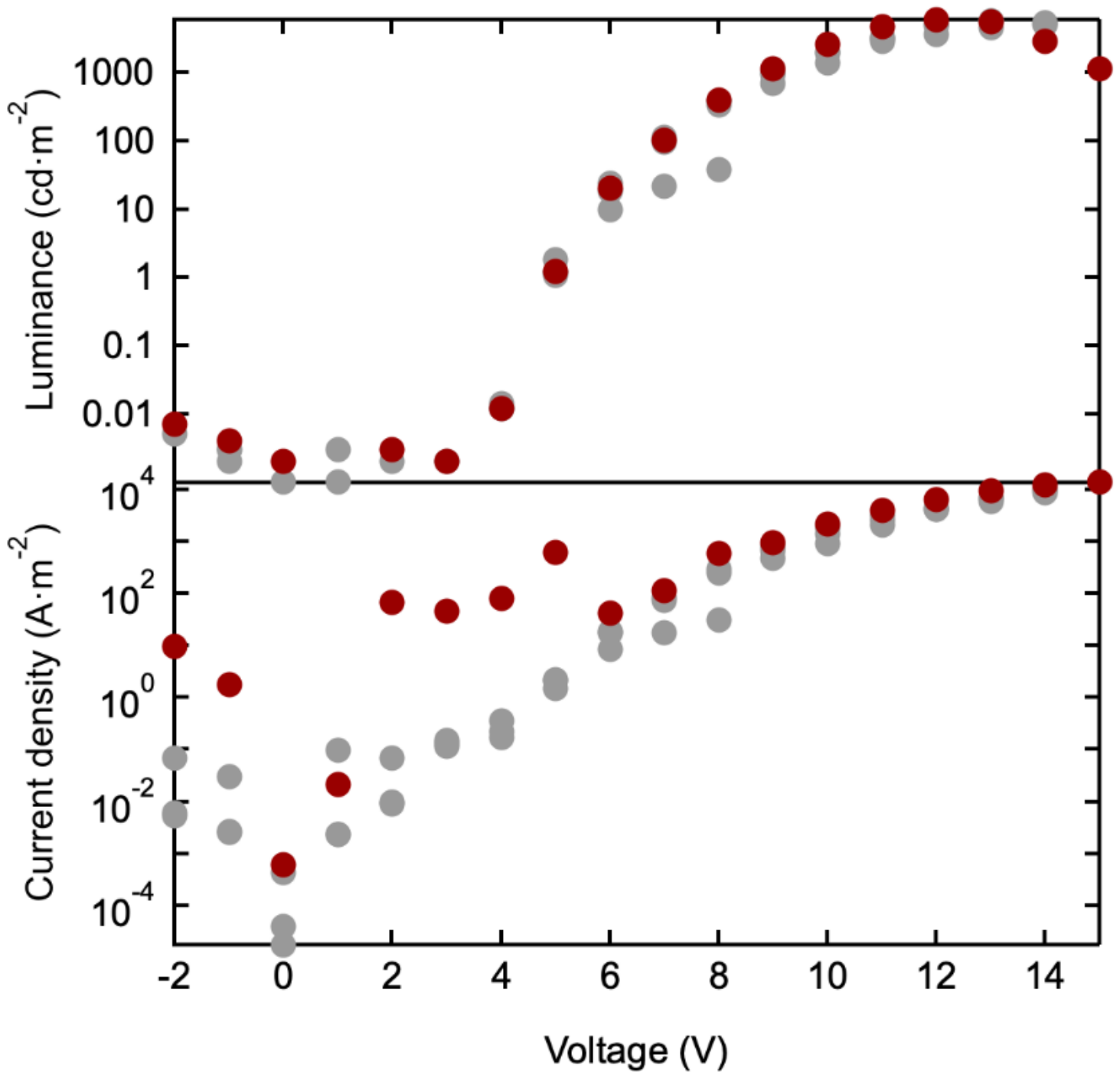


**Figure S34.** JVL characteristics of OLED 17, luminance (top) and current density (bottom). The brightest pixel is displayed in red.

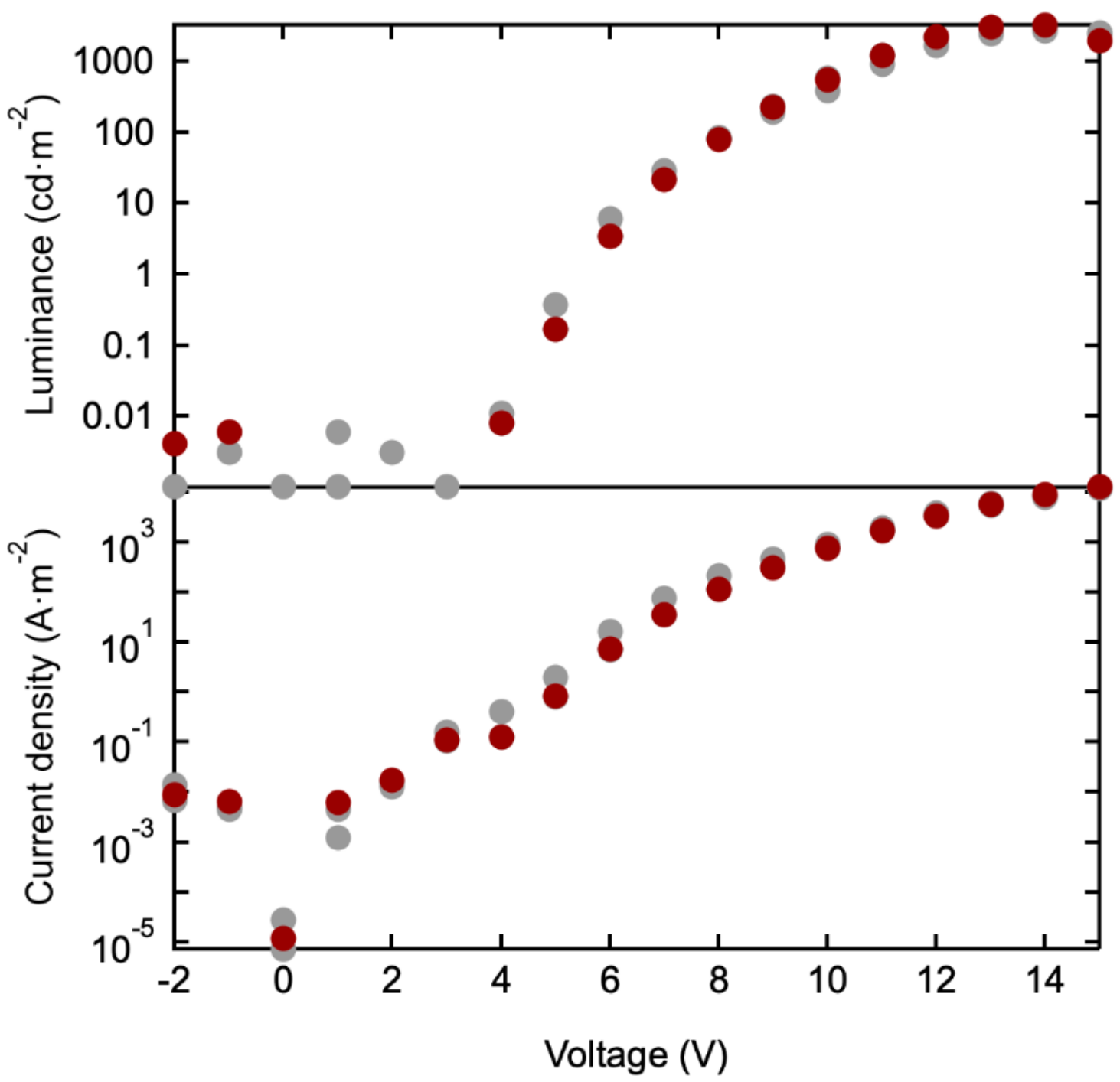


**Figure S35.** JVL characteristics of OLED 18, luminance (top) and current density (bottom). The brightest pixel is displayed in red.

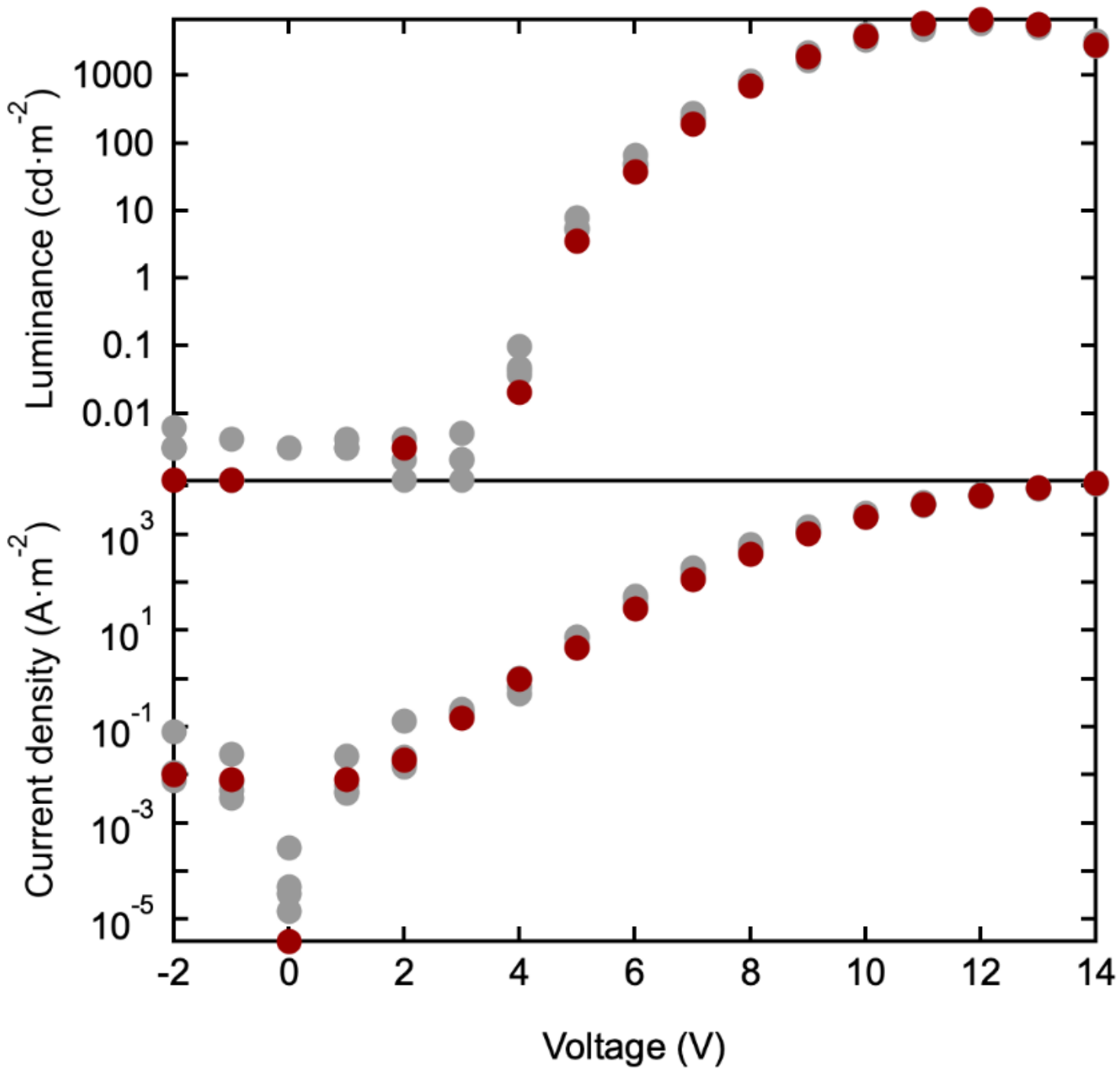


**Figure S36.** JVL characteristics of OLED 19, luminance (top) and current density (bottom). The brightest pixel is displayed in red.

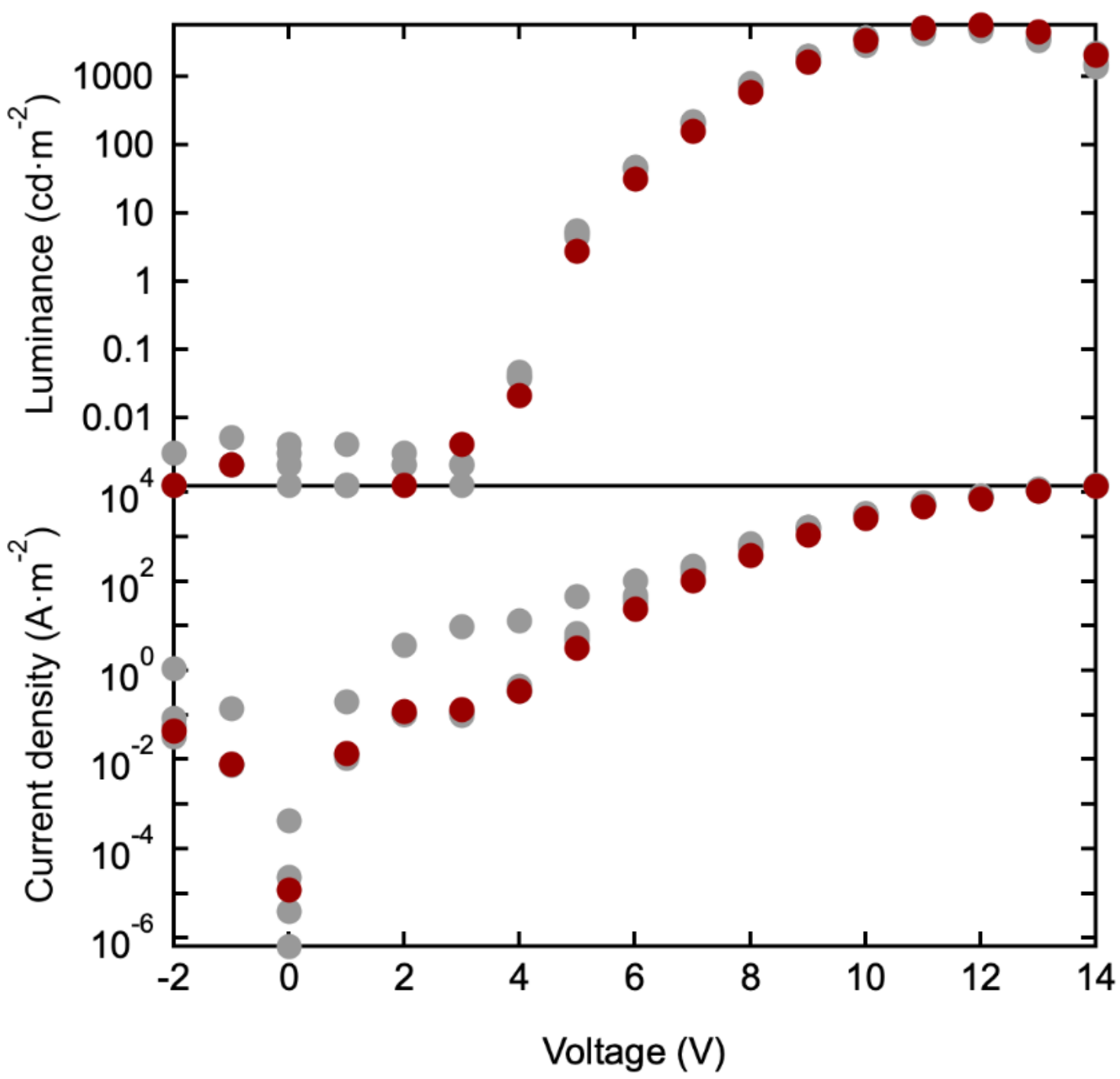


**Figure S37.** JVL characteristics of OLED 20, luminance (top) and current density (bottom). The brightest pixel is displayed in red.

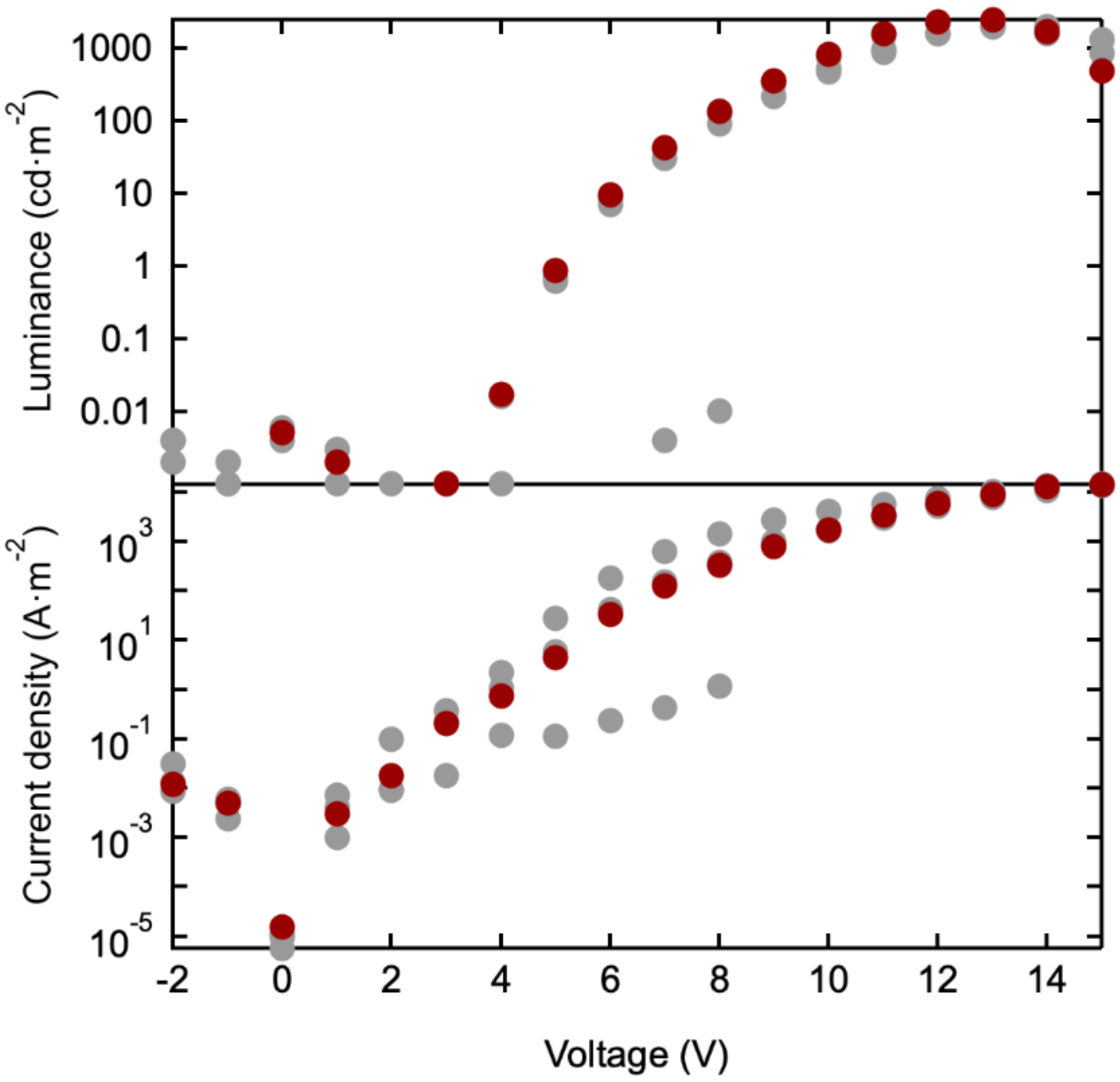


**Figure S38.** JVL characteristics of OLED 21, luminance (top) and current density (bottom). The brightest pixel is displayed in red.

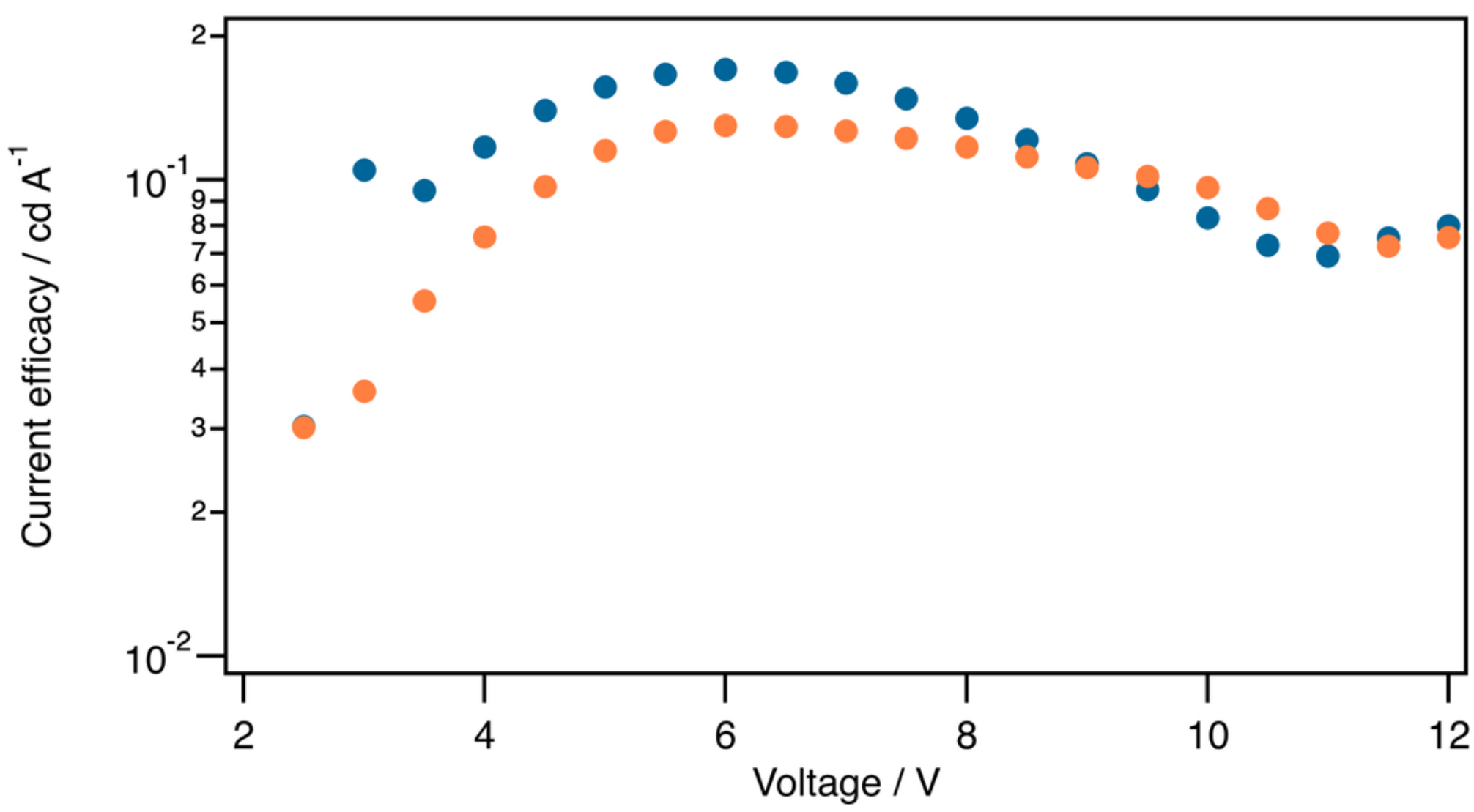


**Figure S39.** Current efficacy of OLED 1 (blue) and OLED 2 (orange) without host material. The highest efficacy is at 6 V and roll-off at higher voltages is observed.

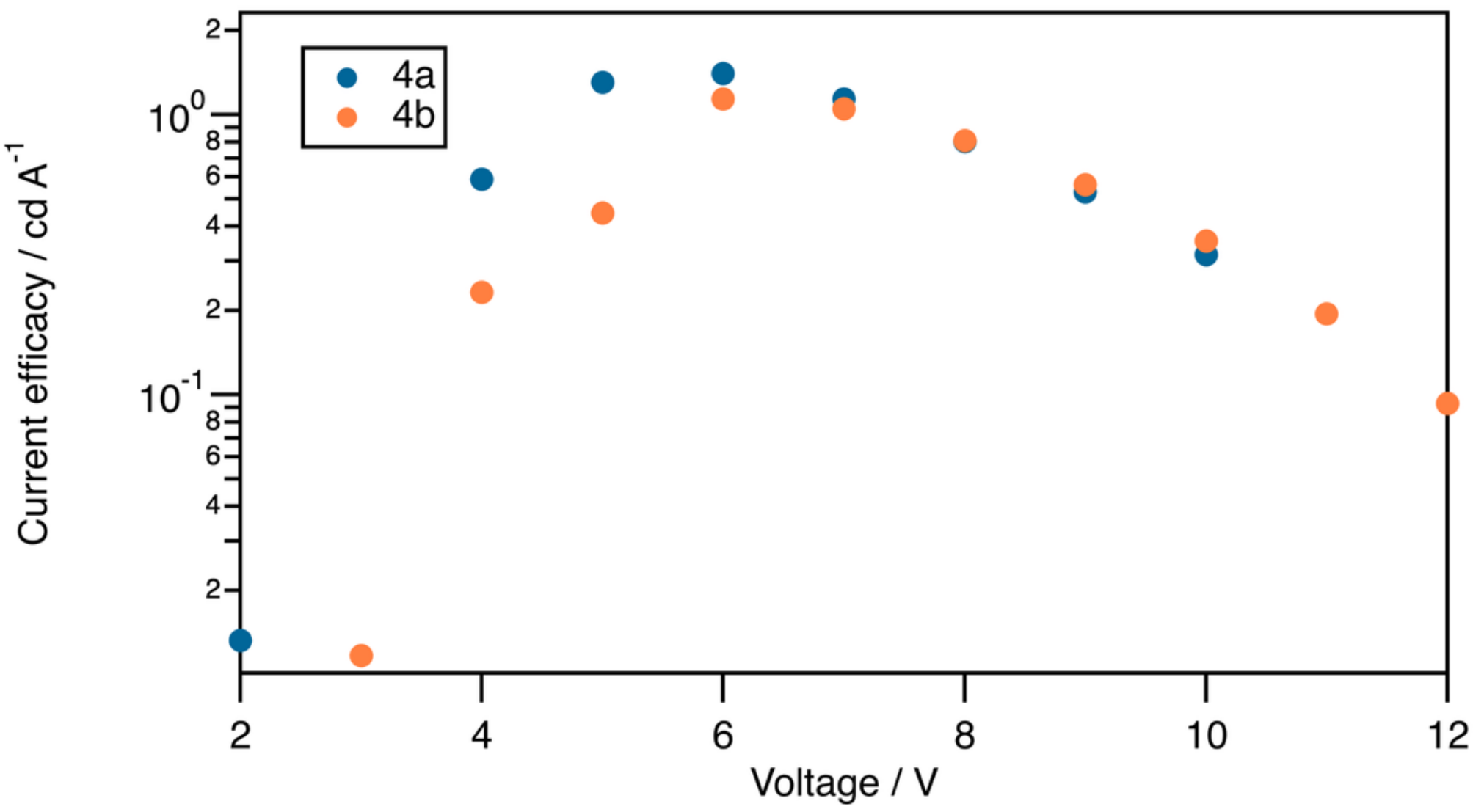


**Figure S40.** Current efficacy of OLED 6 (blue) and OLED 12 (orange) (1 wt% in TCTA). The highest efficacy is at 6 V and roll-off at higher voltages is observed.

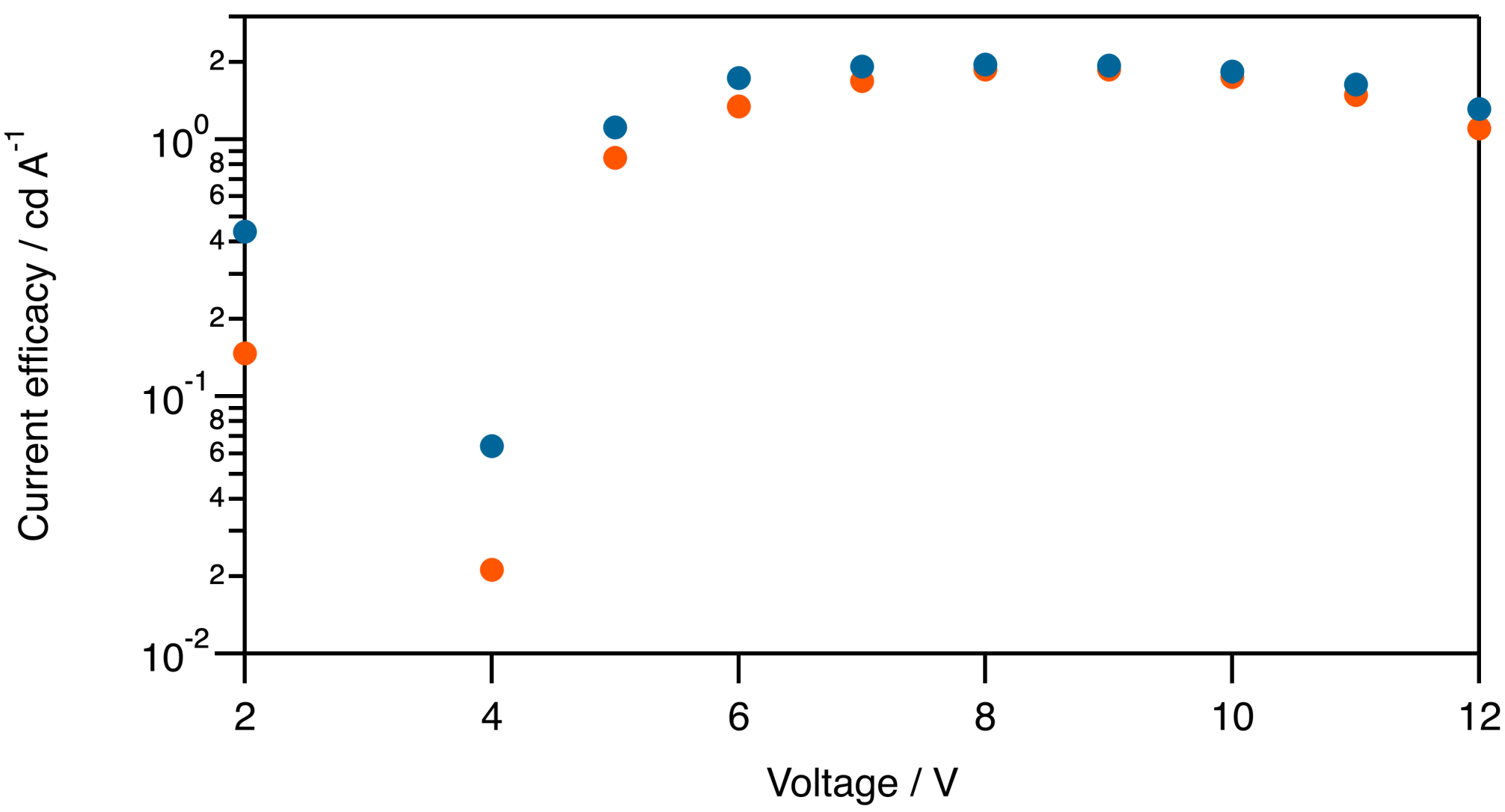


**Figure S41.** Current efficacy of OLED 16 (blue) and OLED 19 (orange) (0.5 wt% in TCTA and TFB layer). The highest efficacy is at 8 V. Only minor roll-off above 10 V is observed.

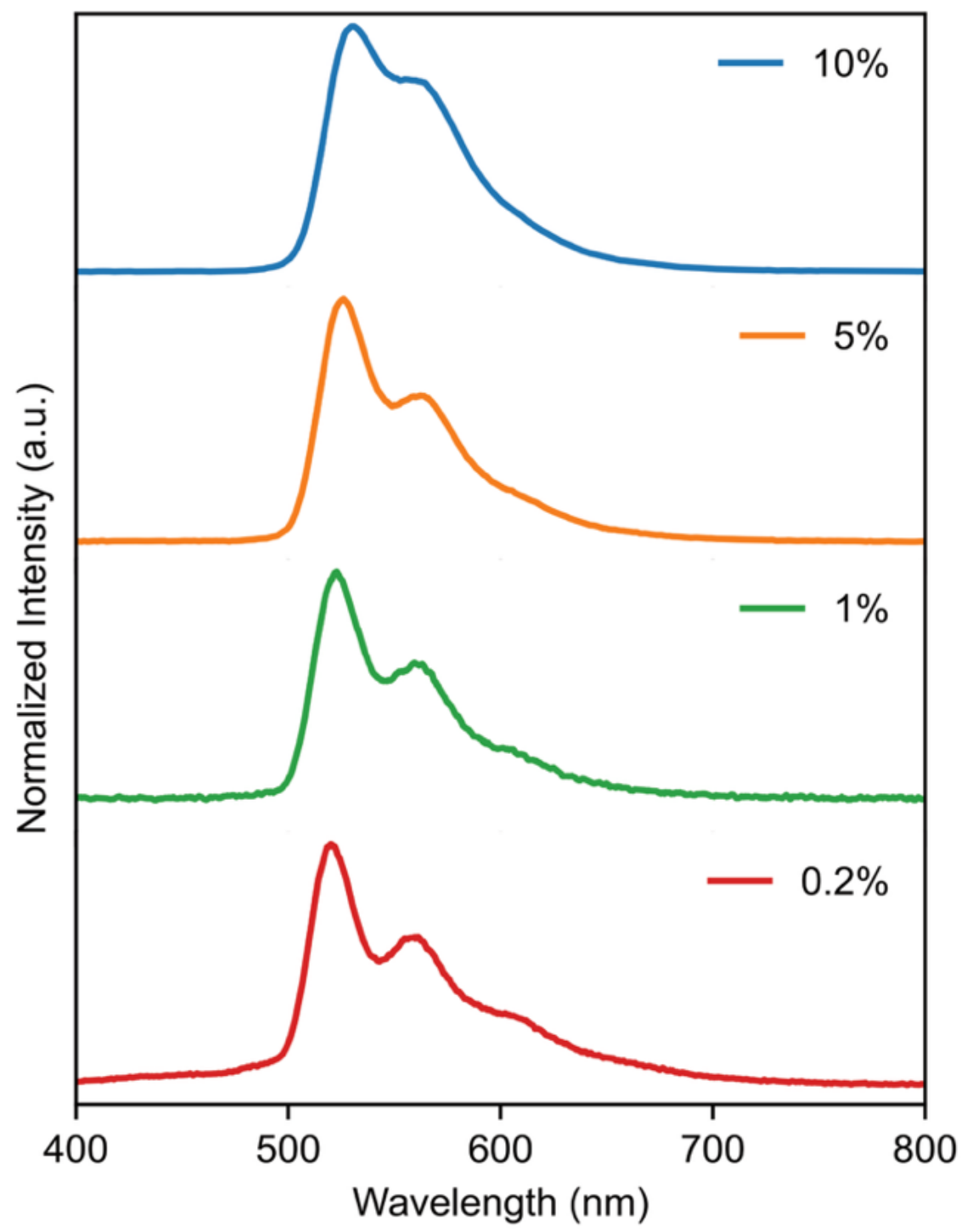


**Figure S42.** Normalized electroluminescence spectra of different ratios of compound **4a** in TCTA host material.

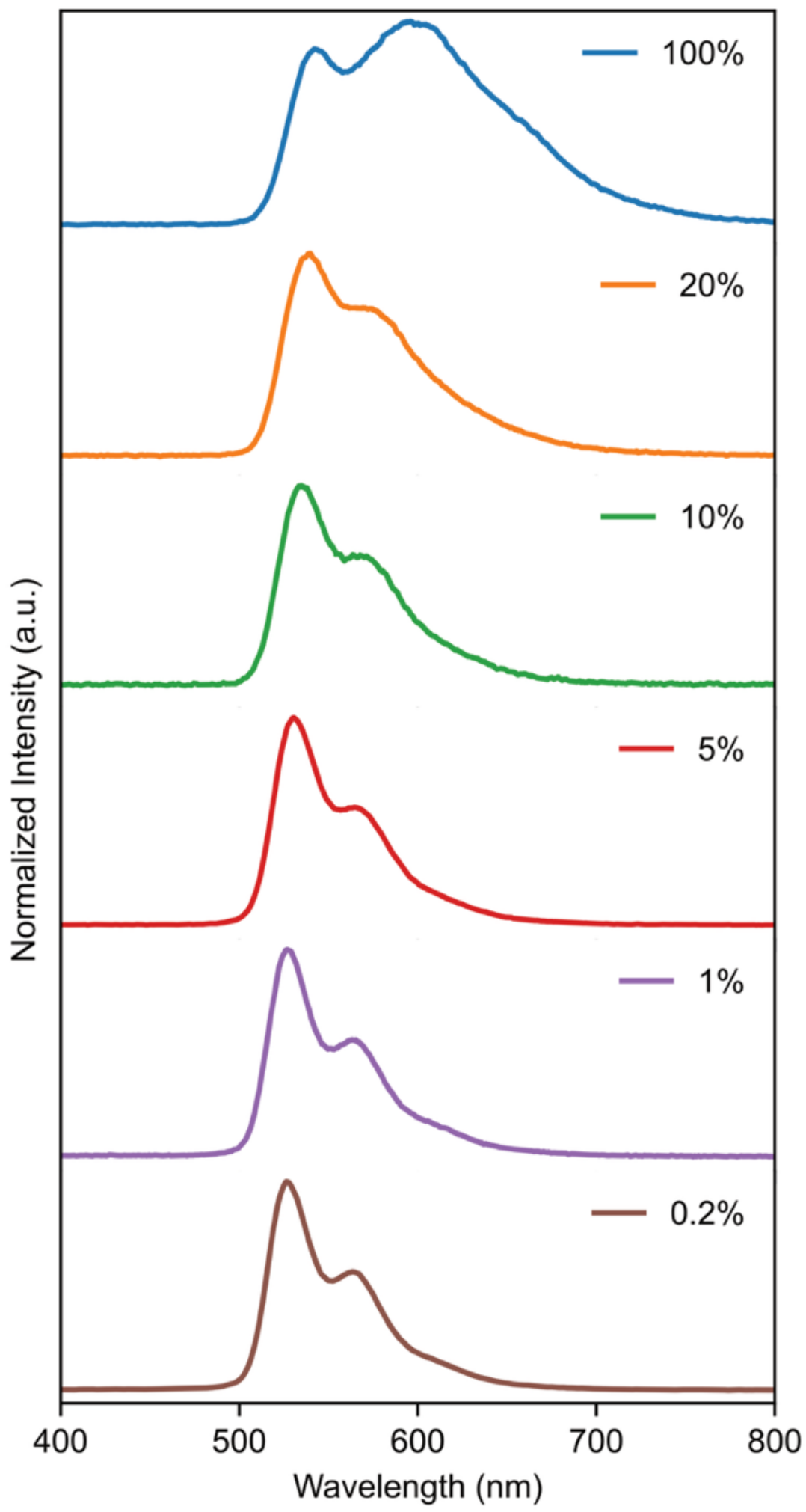


**Figure S43.** Normalized electroluminescence spectra of different ratios of compound **4b** in TCTA host material.

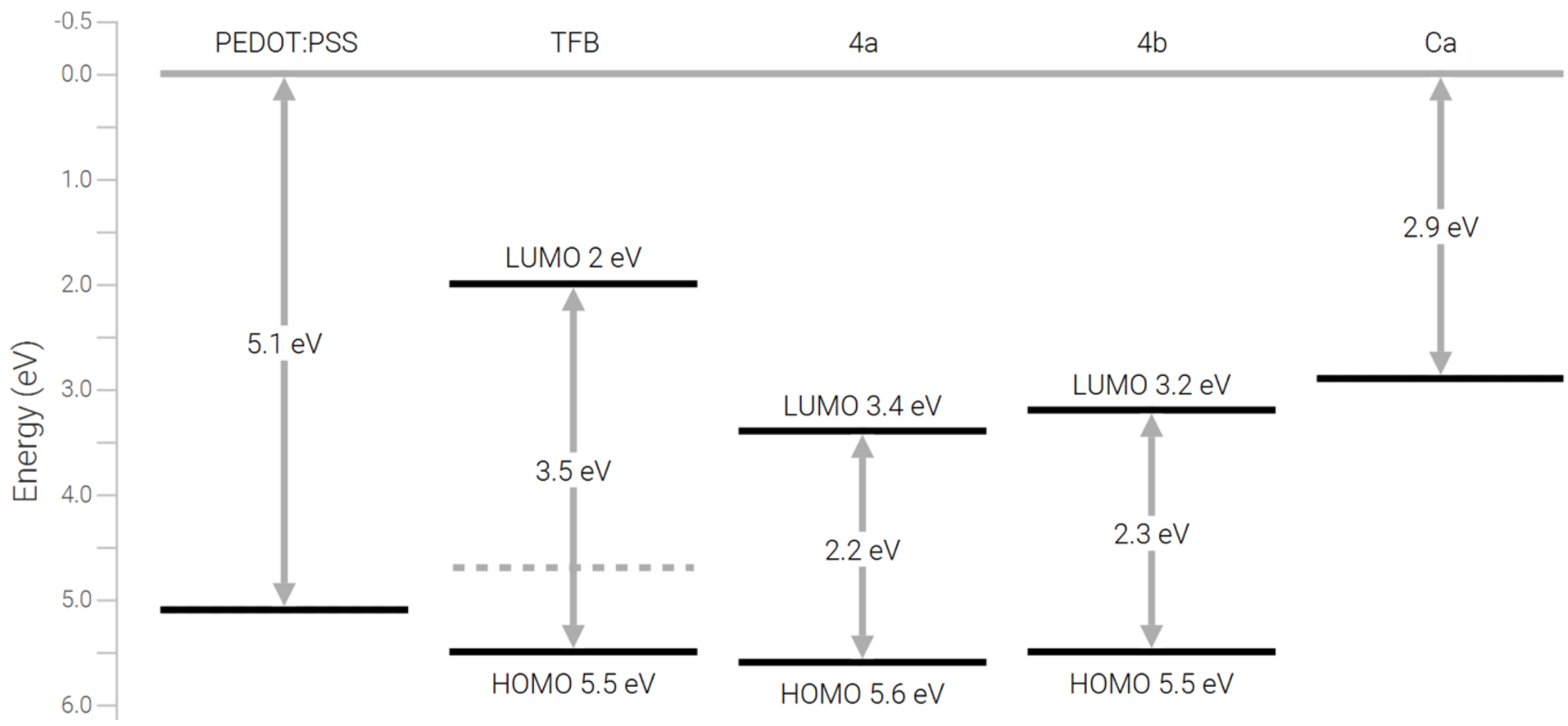


**Figure S44.** Energy levels of the used materials without interfacial effects.

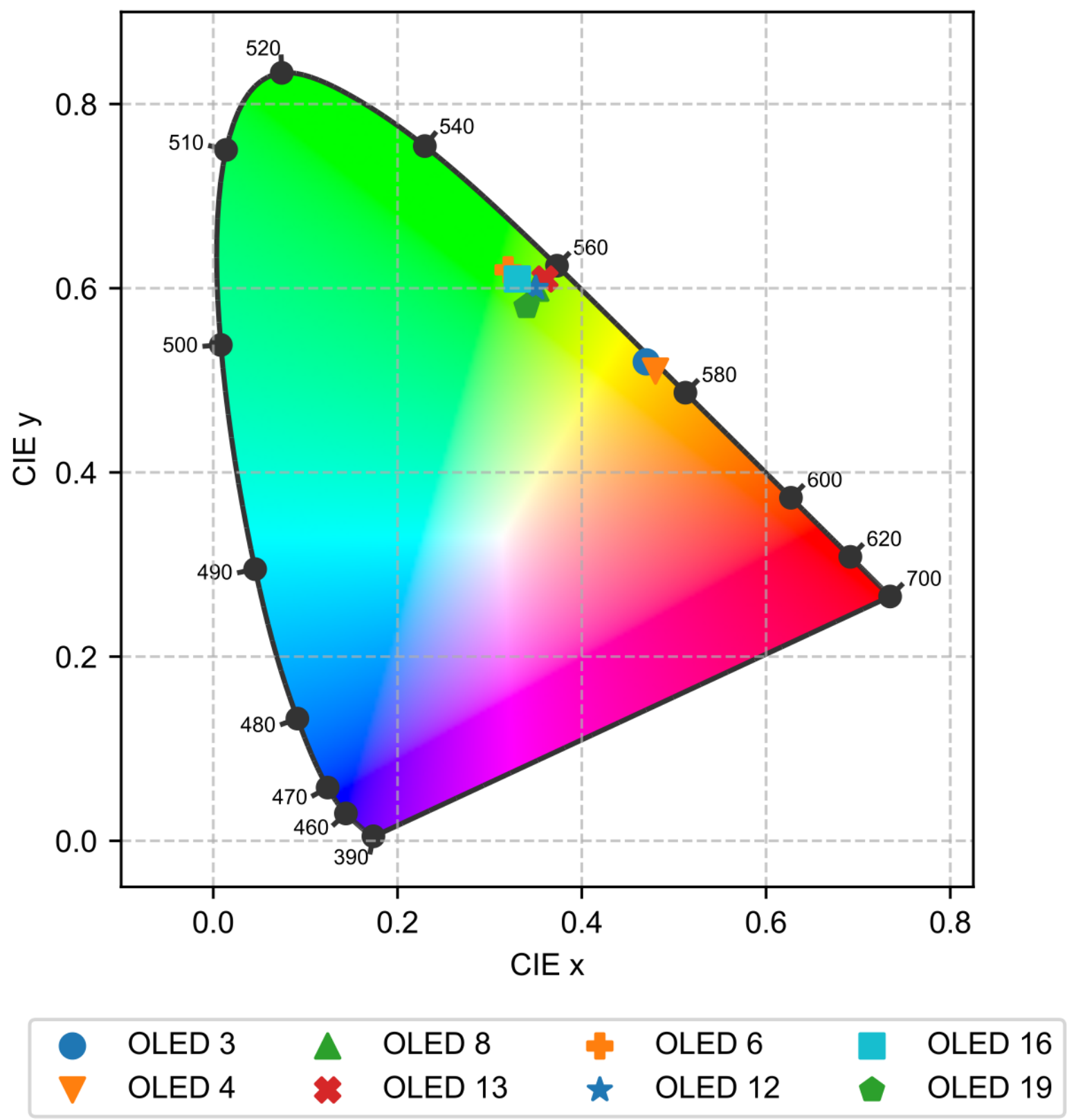


**Figure S45.** CIE 1931 chromaticity diagram coordinates of OLEDs listed in **Table S13**.

# 9 References


(1) *SAINT, V8.40B*; Bruker AXS Inc.: Madison, Wisconsin, USA, 2012.
(2) Krause, L.; Herbst-Irmer, R.; Sheldrick, G. M.; Stalke, D. Comparison of silver and molybdenum microfocus X-ray sources for single-crystal structure determination. *J. Appl. Crystallogr.* **2015**, *48* (1), 3–10. DOI: 10.1107/S1600576714022985.
(3) Sheldrick, G. SHELXT - Integrated space-group and crystal-structure determination. *Acta Crystallogr. Sect. A: Found. Adv.* **2015**, *71* (1), 3–8. DOI: 10.1107/S2053273314026370.
(4) Sheldrick, G. M. Crystal structure refinement with SHELXL. *Acta Crystallogr., Sect. C: Struct. Chem.* **2015**, *71* (1), 3–8. DOI: 10.1107/S2053229614024218.
(5) Spek, A. L. Single-crystal structure validation with the program PLATON. *J. Appl. Crystallogr.* **2003**, *36* (1), 7–13. DOI: 10.1107/S0021889802022112.
(6) Neese, F. Software Update: The ORCA Program System—Version 6.0. *Wiley Interdiscip. Rev.: Comput. Mol. Sci.* **2025**, *15* (2), e70019. DOI: 10.1002/wcms.70019.
(7) Chai, J. D.; Head-Gordon, M. Long-range corrected hybrid density functionals with damped atom-atom dispersion corrections. *Phys. Chem. Chem. Phys.* **2008**, *10* (44), 6615–6620. DOI: 10.1039/b810189b.
(8) Grimme, S.; Antony, J.; Ehrlich, S.; Krieg, H. A consistent and accurate ab initio parametrization of density functional dispersion correction (DFT-D) for the 94 elements H-Pu. *J. Chem. Phys.* **2010**, *132* (15), 154104. DOI: 10.1063/1.3382344.
(9) Weigend, F.; Ahlrichs, R. Balanced basis sets of split valence, triple zeta valence and quadruple zeta valence quality for H to Rn: Design and assessment of accuracy. *Phys. Chem. Chem. Phys.* **2005**, *7* (18), 3297–3305. DOI: 10.1039/b508541a.
(10) Tomasi, J.; Mennucci, B.; Cammi, R. Quantum Mechanical Continuum Solvation Models. *Chem. Rev.* **2005**, *105*, 2999–3093. DOI: 10.1021/cr9904009.
(11) Becke, A. D. Density-functional thermochemistry. III. The role of exact exchange. *J. Chem. Phys.* **1993**, *98* (7), 5648–5652. DOI: 10.1063/1.464913.
(12) Lee, C.; Yang, W.; Parr, R. G. Development of the Colle-Salvetti correlation-energy formula into a functional of the electron density. *Phys. Rev. B* **1988**, *37* (2), 785–789. DOI: 10.1103/physrevb.37.785.
(13) *Gaussian 16 Rev. C.01*; Wallingford, CT, 2016.
(14) *NBO 7.0;* Madison, WI, 2018.
(15) Szczepanik, D. W.; Andrzejak, M.; Dyduch, K.; Żak, E.; Makowski, M.; Mazur, G.; Mrozek, J. A uniform approach to the description of multicenter bonding. *Phys. Chem. Chem. Phys.* **2014**, *16* (38), 20514–20523. DOI: 10.1039/c4cp02932a.
(16) Szczepanik, D. W. A new perspective on quantifying electron localization and delocalization in molecular systems. *Comput. Theor. Chem.* **2016**, *1080*, 33–37. DOI: 10.1016/j.comptc.2016.02.003.
(17) Szczepanik, D. W.; Andrzejak, M.; Dominikowska, J.; Pawełek, B.; Krygowski, T. M.; Szatylowicz, H.; Solà, M. The electron density of delocalized bonds (EDDB) applied for quantifying aromaticity. *Phys. Chem. Chem. Phys.* **2017**, *19* (42), 28970–28981. DOI: 10.1039/c7cp06114e.
(18) Parr, R. G.; Yang, W. Density functional approach to the frontier-electron theory of chemical reactivity. *J. Am. Chem. Soc.* **1984**, *106* (14), 4049–4050. DOI: 10.1021/ja00326a036.
(19) Lu, T.; Chen, F. Multiwfn: A multifunctional wavefunction analyzer. *J. Comput. Chem.* **2012**, *33* (5), 580–592. DOI: 10.1002/jcc.22885.
(20) Lu, T. A comprehensive electron wavefunction analysis toolbox for chemists, Multiwfn. *J. Chem. Phys.* **2024**, *161* (8), 082503. DOI: 10.1063/5.0216272.
(21) Meng, E. C.; Goddard, T. D.; Pettersen, E. F.; Couch, G. S.; Pearson, Z. J.; Morris, J. H.; Ferrin, T. E. UCSF ChimeraX: Tools for structure building and analysis. *Protein Sci.* **2023**, *32* (11), e4792. DOI: 10.1002/pro.4792.
(22) Rietsch, P.; Soyka, J.; Brulls, S.; Er, J.; Hoffmann, K.; Beerhues, J.; Sarkar, B.; Resch-Genger, U.; Eigler, S. Fluorescence of a chiral pentaphene derivative derived from the hexabenzocoronene Motif. *Chem. Commun.* **2019**, *55* (71), 10515–10518. DOI: 10.1039/c9cc05451k.

(23) Eyring, H. The theory of absolute reaction rates. *Trans. Faraday Soc.* **1938**, *34*, 41–48. DOI: 10.1039/tf9383400041.
(24) Greenham, N. C.; Friend, R. H.; Bradley, D. D. C. Angular Dependence of the Emission from a Conjugated Polymer Light-Emitting Diode: Implications for efficiency calculations. *Adv. Mater.* **1994**, *6* (6), 491–494. DOI: 10.1002/adma.19940060612.
(25) Forrest, S. R.; Bradley, D. D. C.; Thompson, M. E. Measuring the Efficiency of Organic Light-Emitting Devices. *Adv. Mater.* **2003**, *15* (13), 1043–1048. DOI: 10.1002/adma.200302151.

# 10 Spectra Appendix

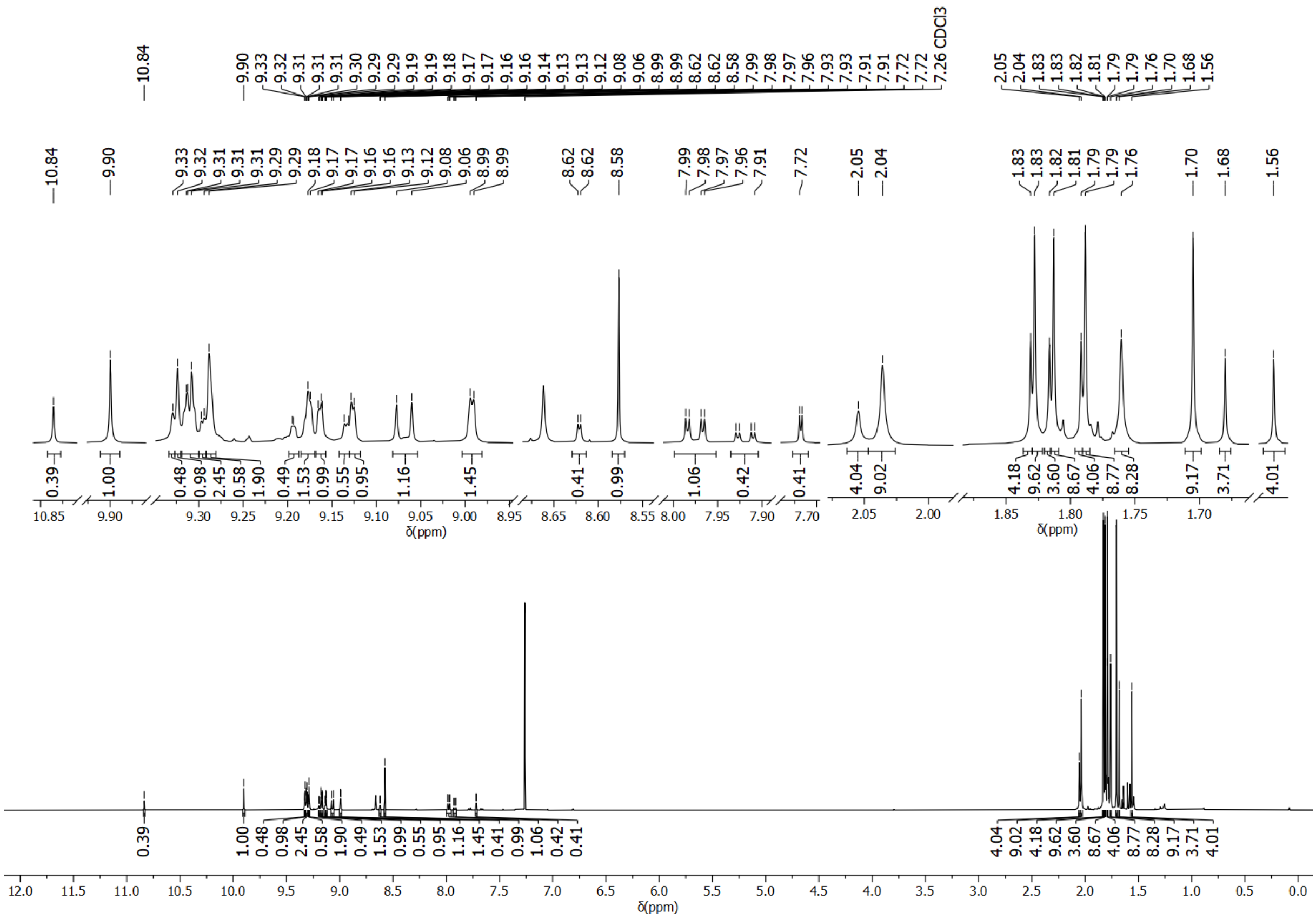

**Figure S46.** $^{1}H$ NMR spectrum (500 MHz, $CDCl_3$, rt) of compound mixture **3a**/**3b**.

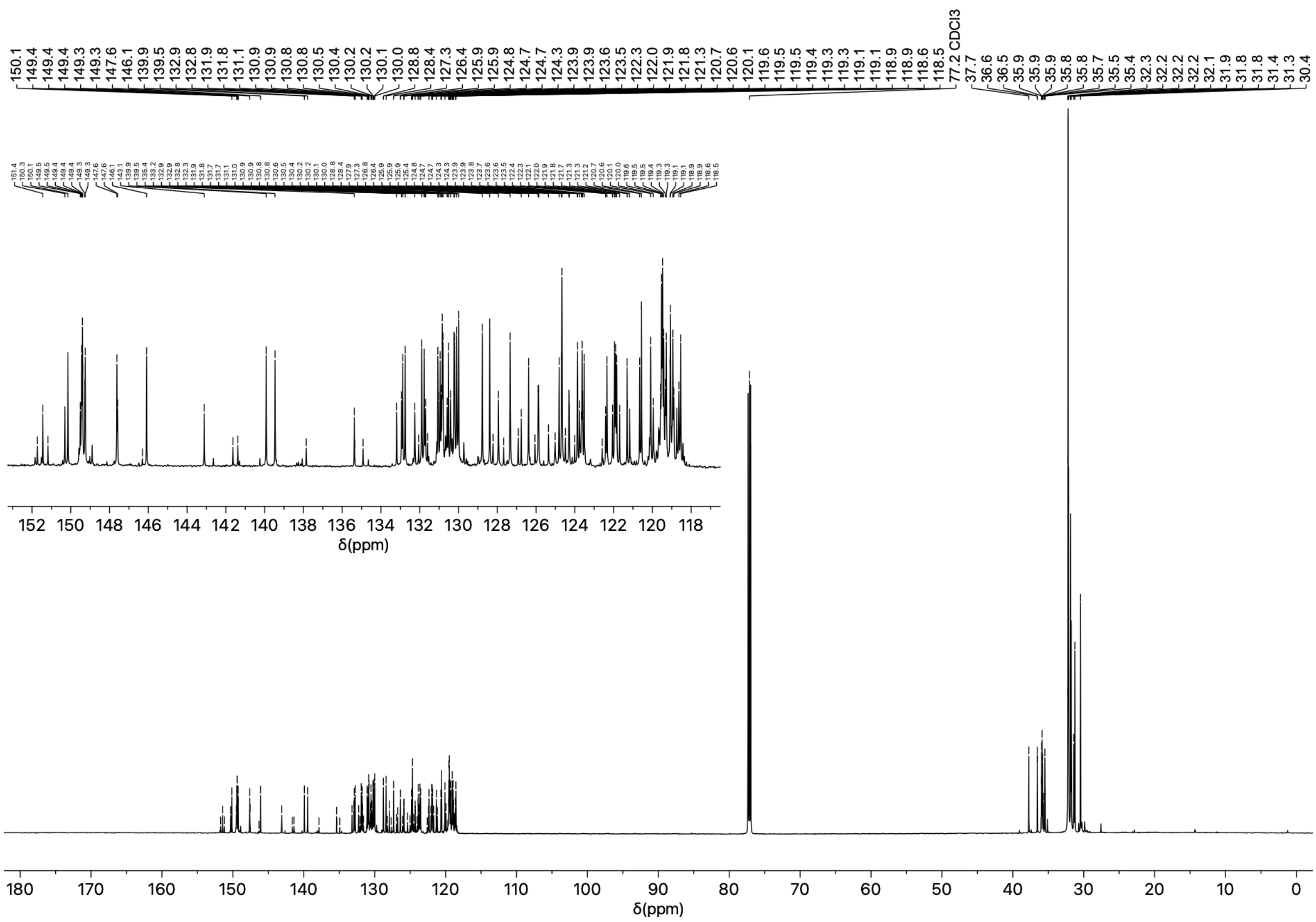

**Figure S47**. $^{13}C$ NMR spectrum (176 MHz, $CDCl_3$, rt) of compound mixture **3a**/**3b**.

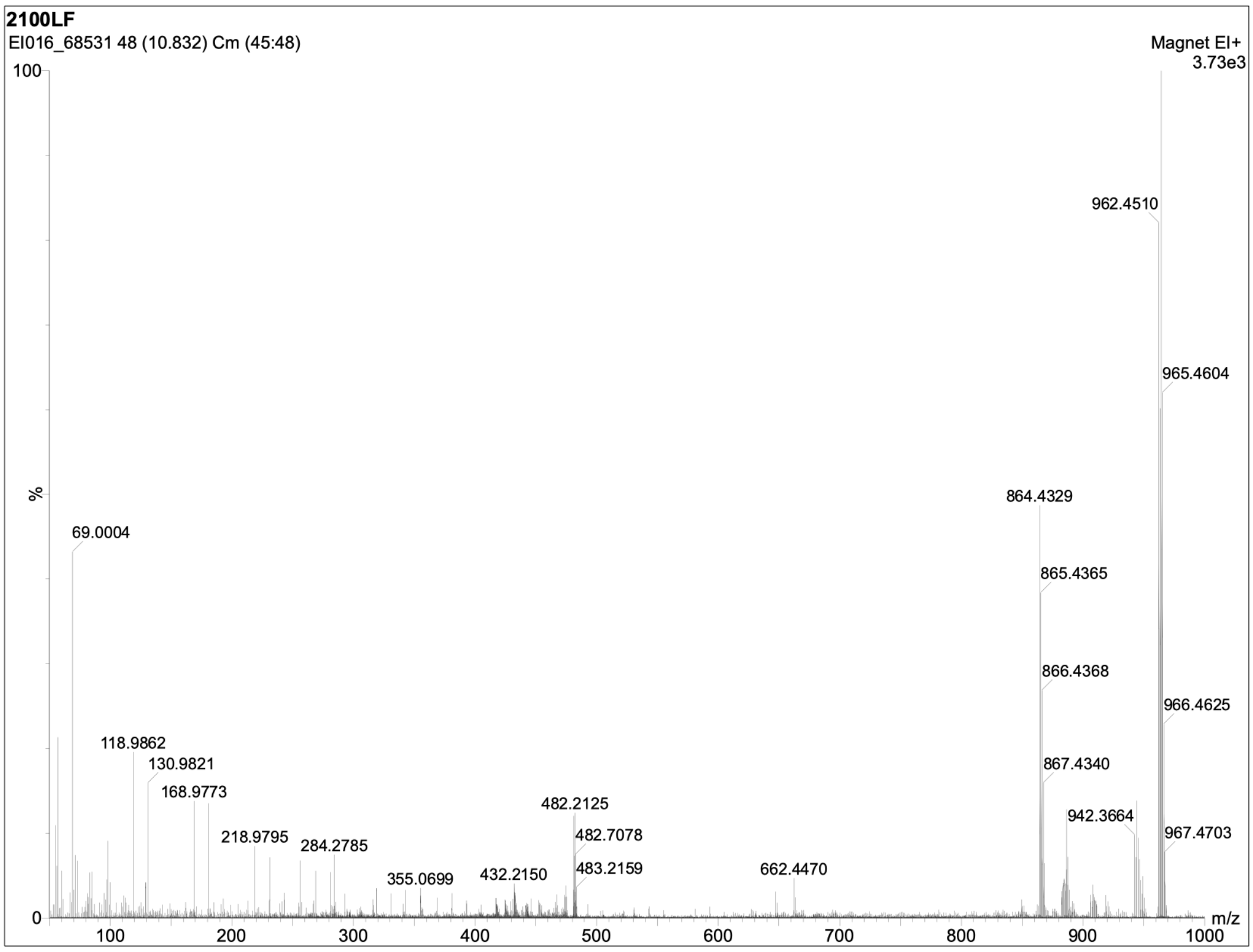


**Figure S48.** EI-HRMS spectrum of compound mixture **3a**/**3b**.

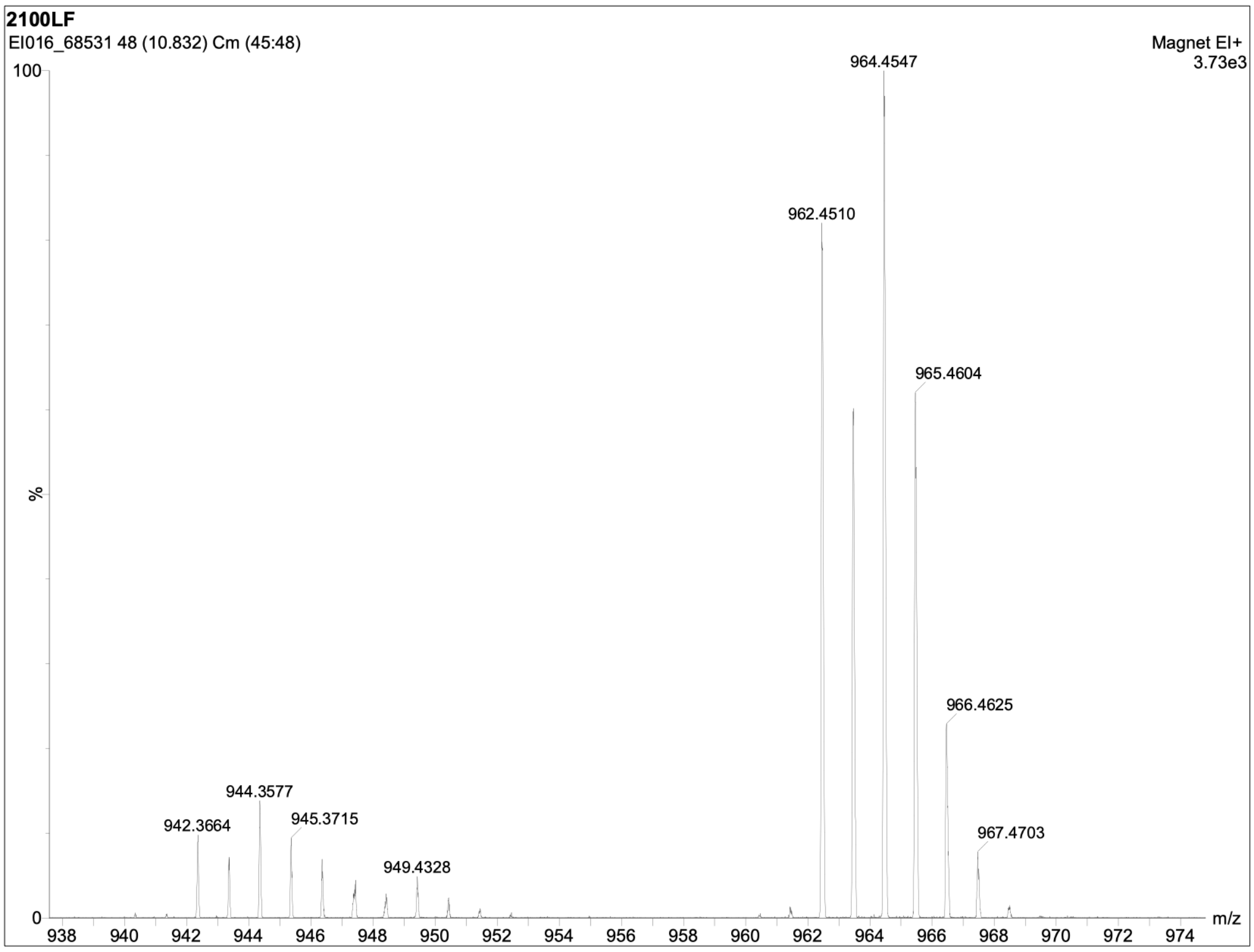


**Figure S49.** EI-HRMS spectrum of compound mixture **3a**/**3b**, isotope pattern magnified.

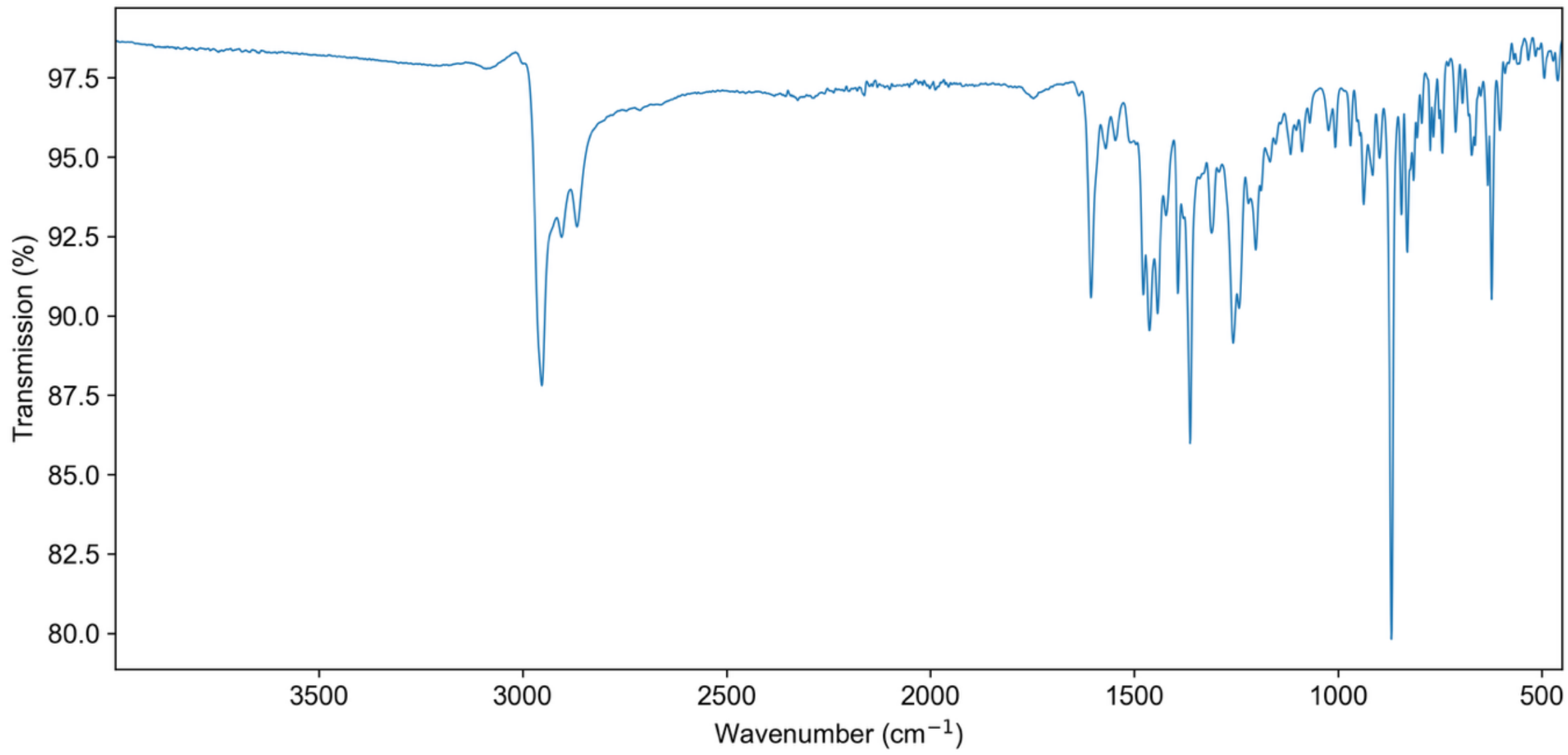

**Figure S50.** FT-IR spectrum of compound mixture **3a**/**3b**.

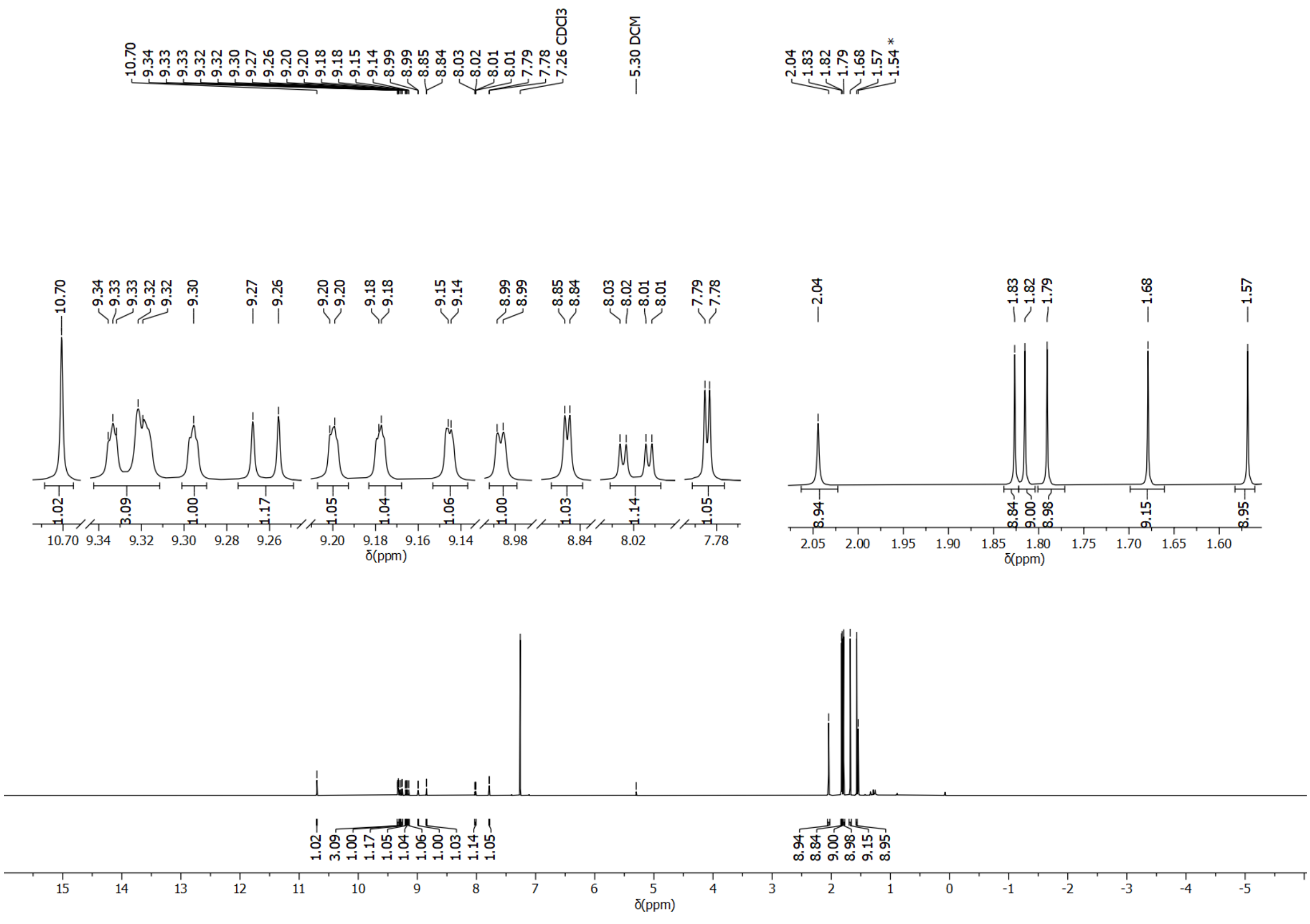

**Figure S51.** $^{1}$H NMR spectrum (700 MHz, $CDCl_3$, rt) of compound **4a**.

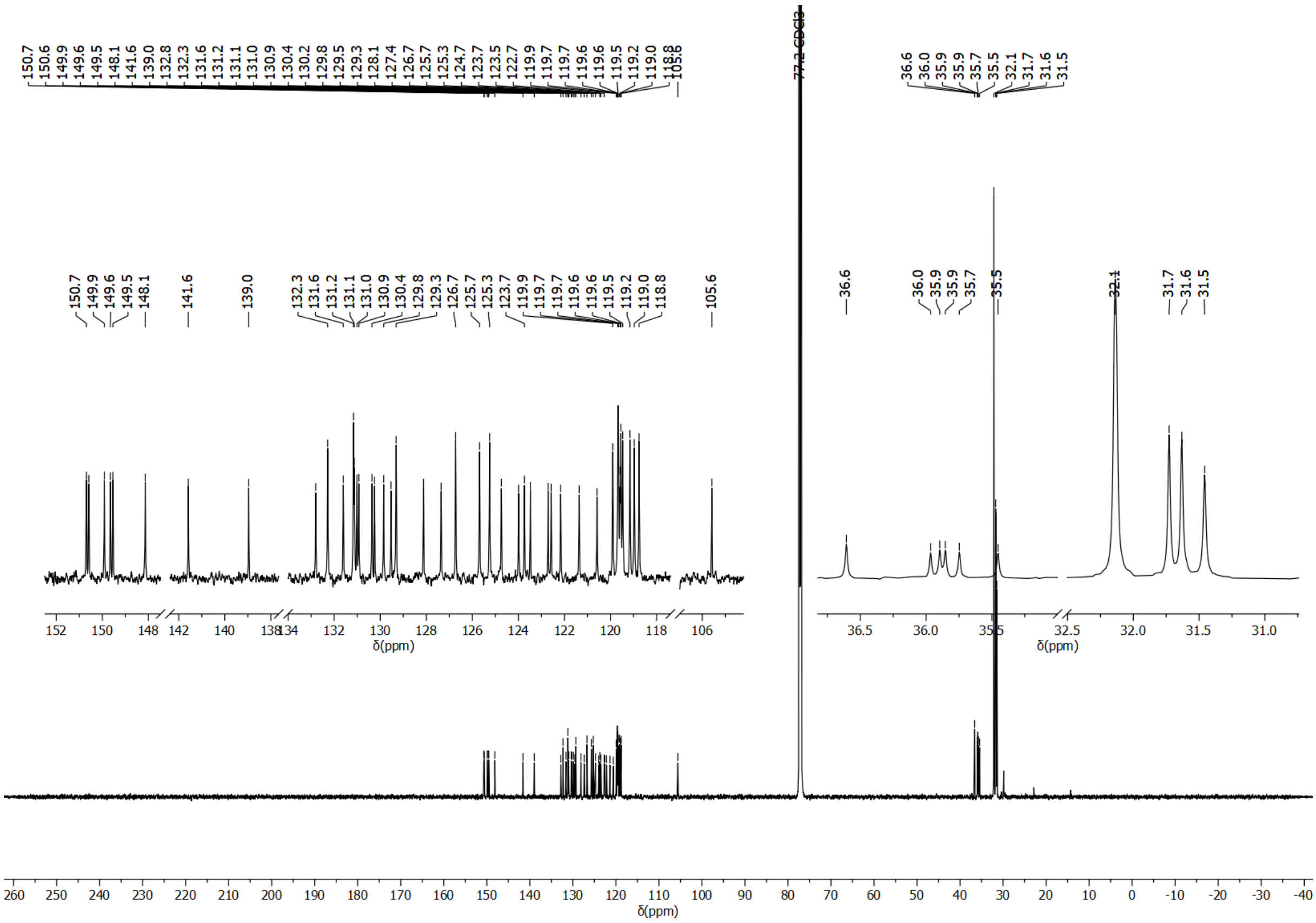

**Figure S52.** $^{13}C$ NMR spectrum (176 MHz, $CDCl_3$, rt) of compound **4a**.

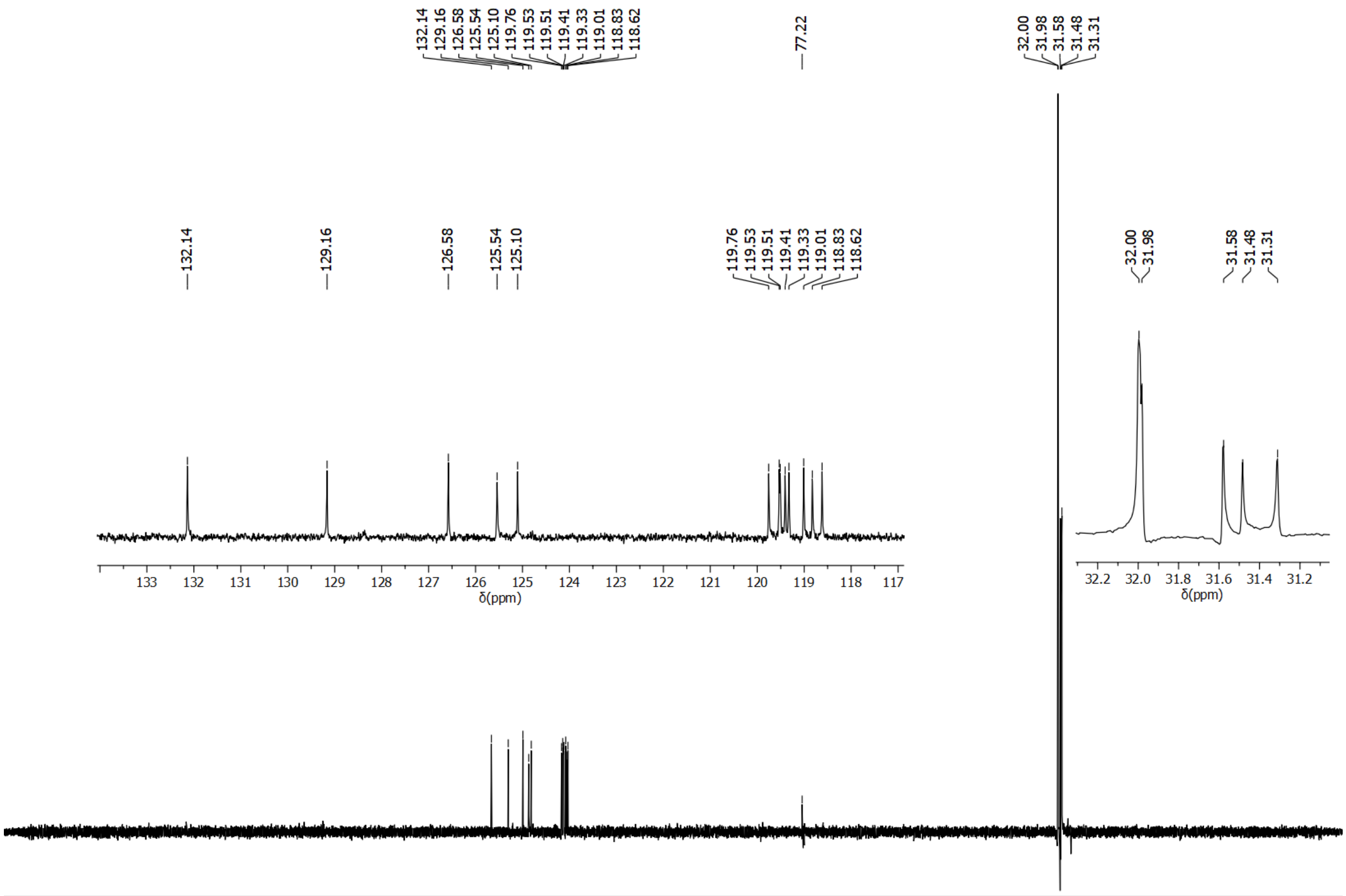

**Figure S53.** $^{13}C$ DEPT135 NMR spectrum (176 MHz, $CDCl_3$, rt) of compound **4a**.

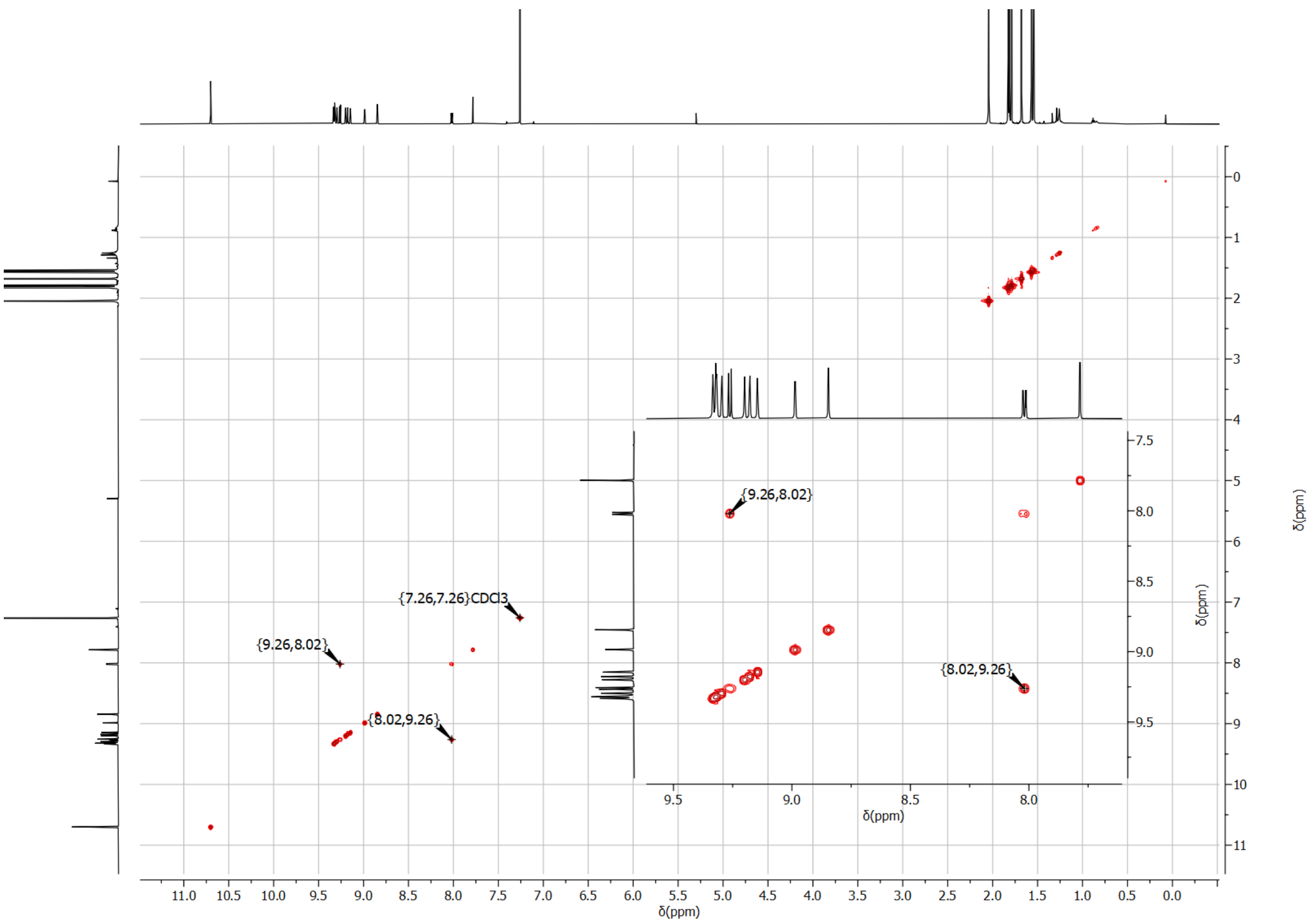

**Figure S54.** $^{1}H$-$^{1}H$ COSY NMR spectrum (700, 700 MHz, $CDCl_3$, rt) of compound **4a**.

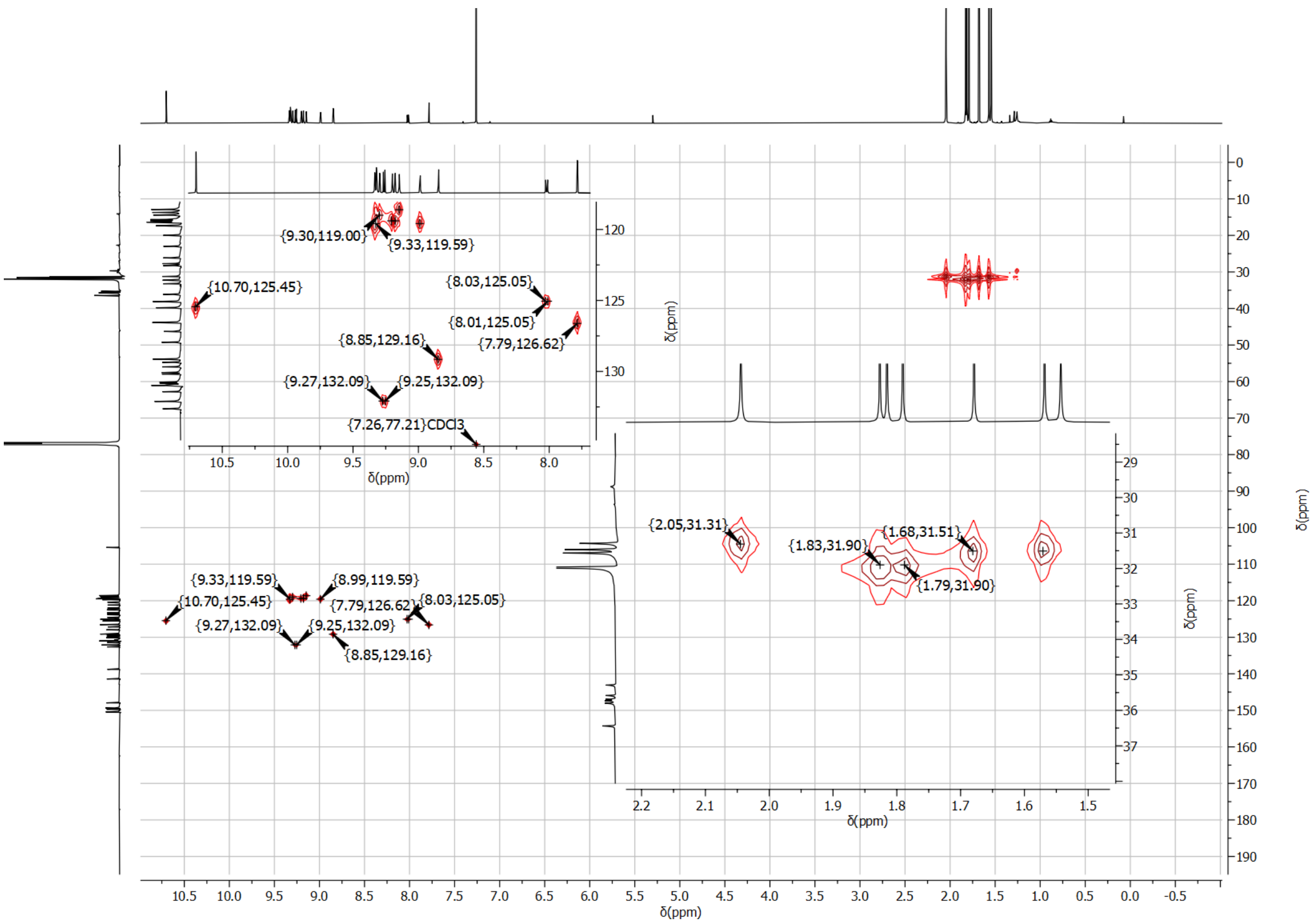

**Figure S55.** $^{1}H$-$^{13}C$ HMQC NMR spectrum (700, 176 MHz, $CDCl_3$, rt) of compound **4a**.

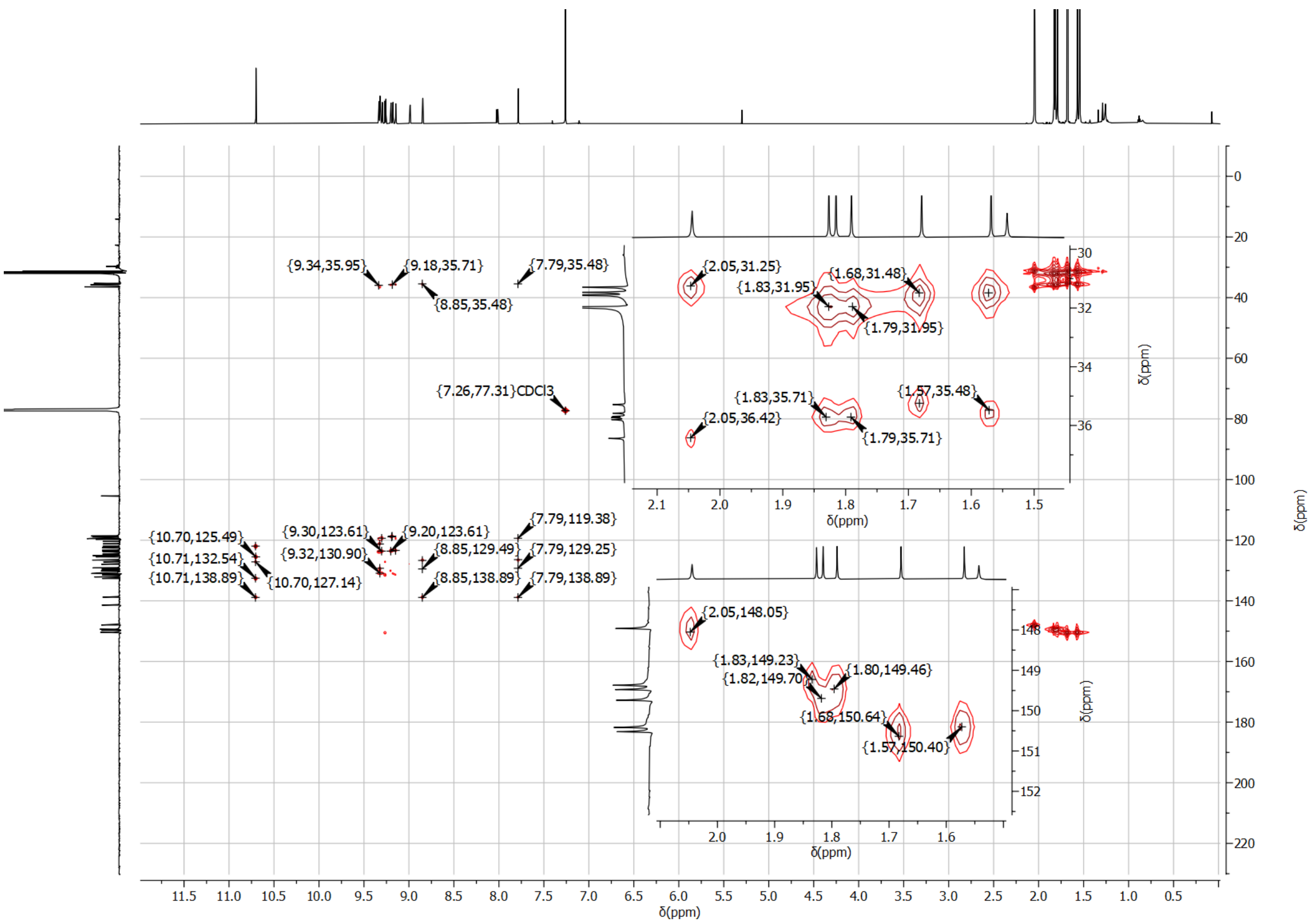


**Figure S56.** $^1H$-$^{13}C$ HMBC NMR spectrum (700, 176 MHz, $CDCl_3$, rt) of compound **4a**.

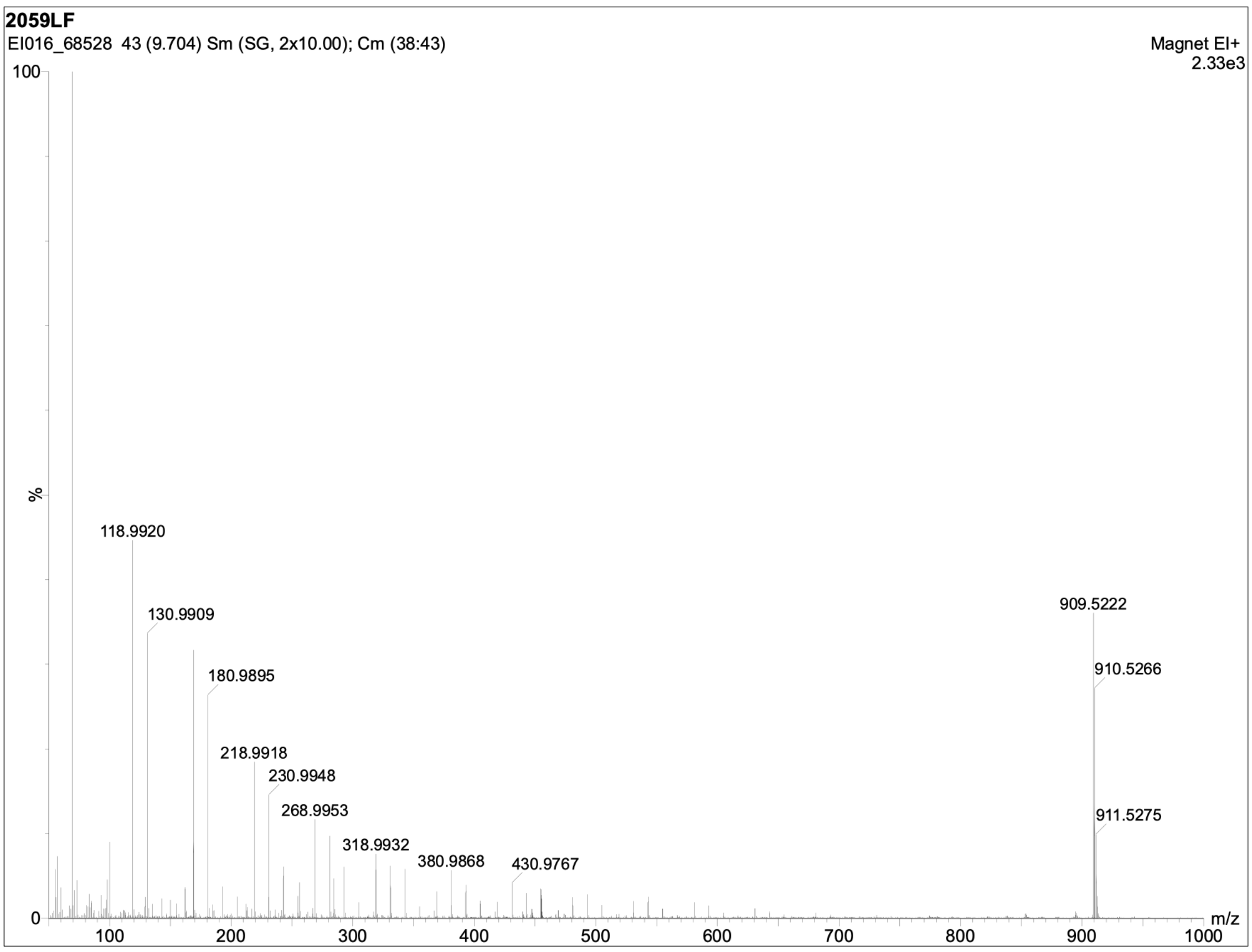


**Figure S57.** EI-HRMS spectrum of compound **4a**.

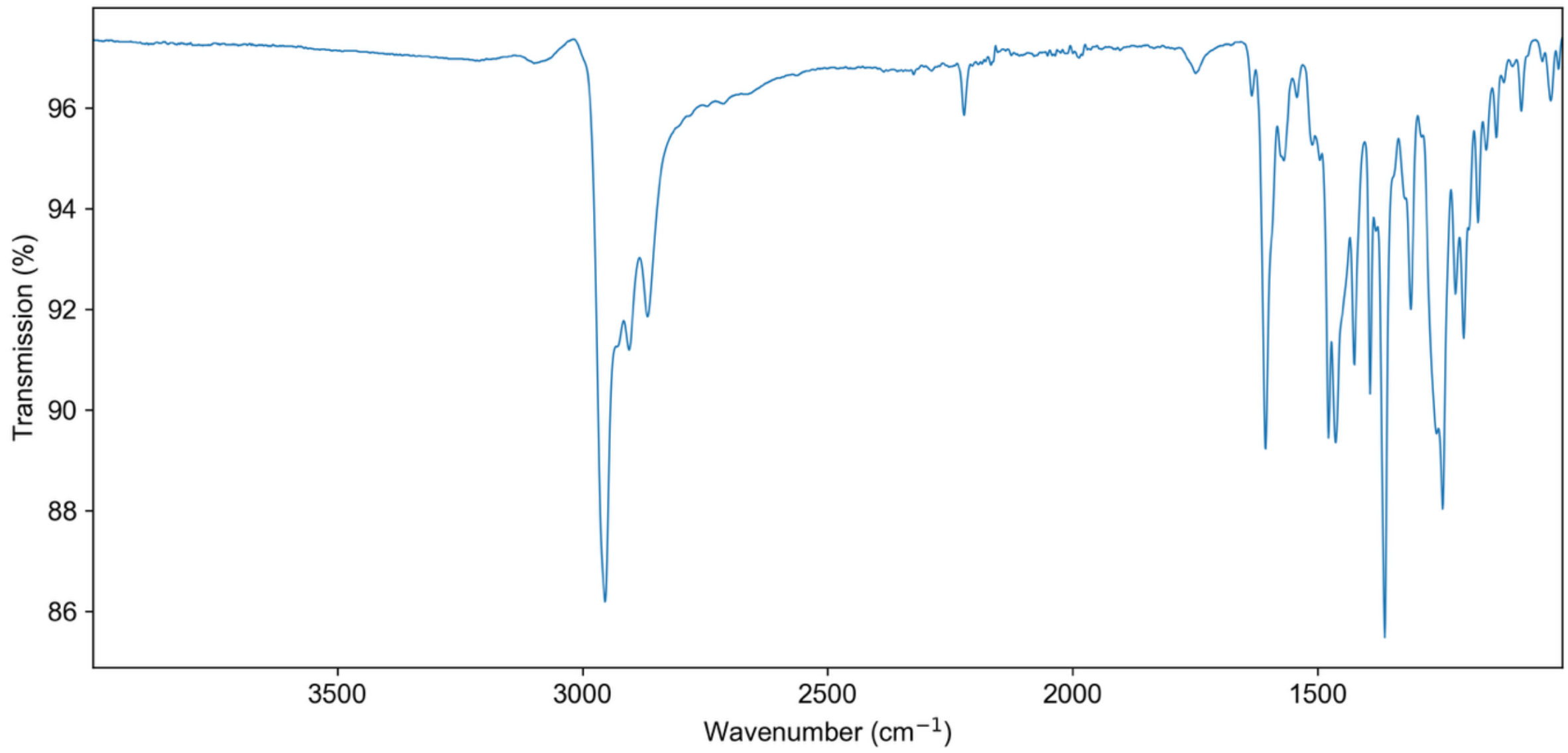


**Figure S58.** FT-IR spectrum of compound **4a**.

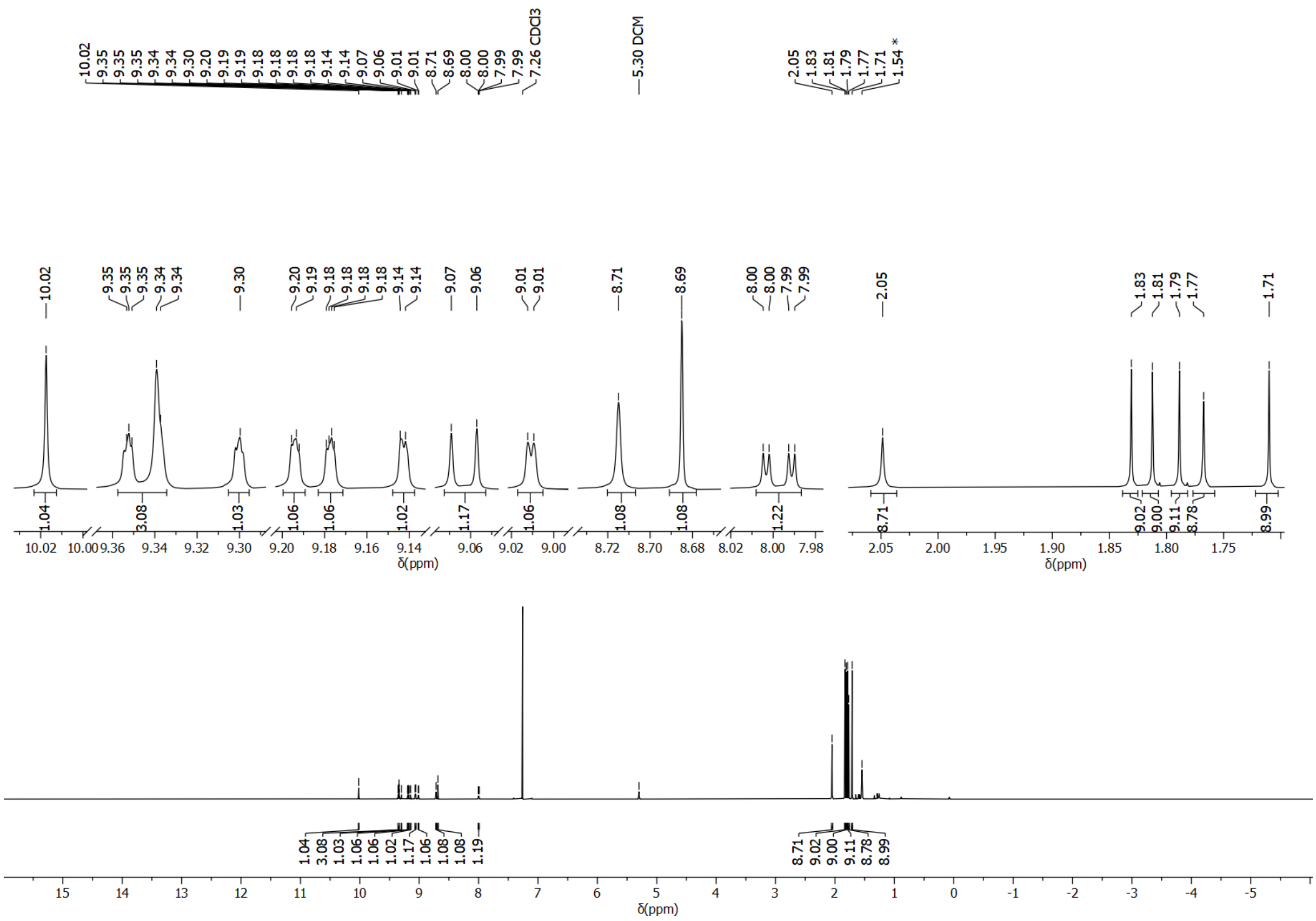


**Figure S59.** $^{1}H$ NMR spectrum (700 MHz, $CDCl_3$, rt) of compound **4b**.

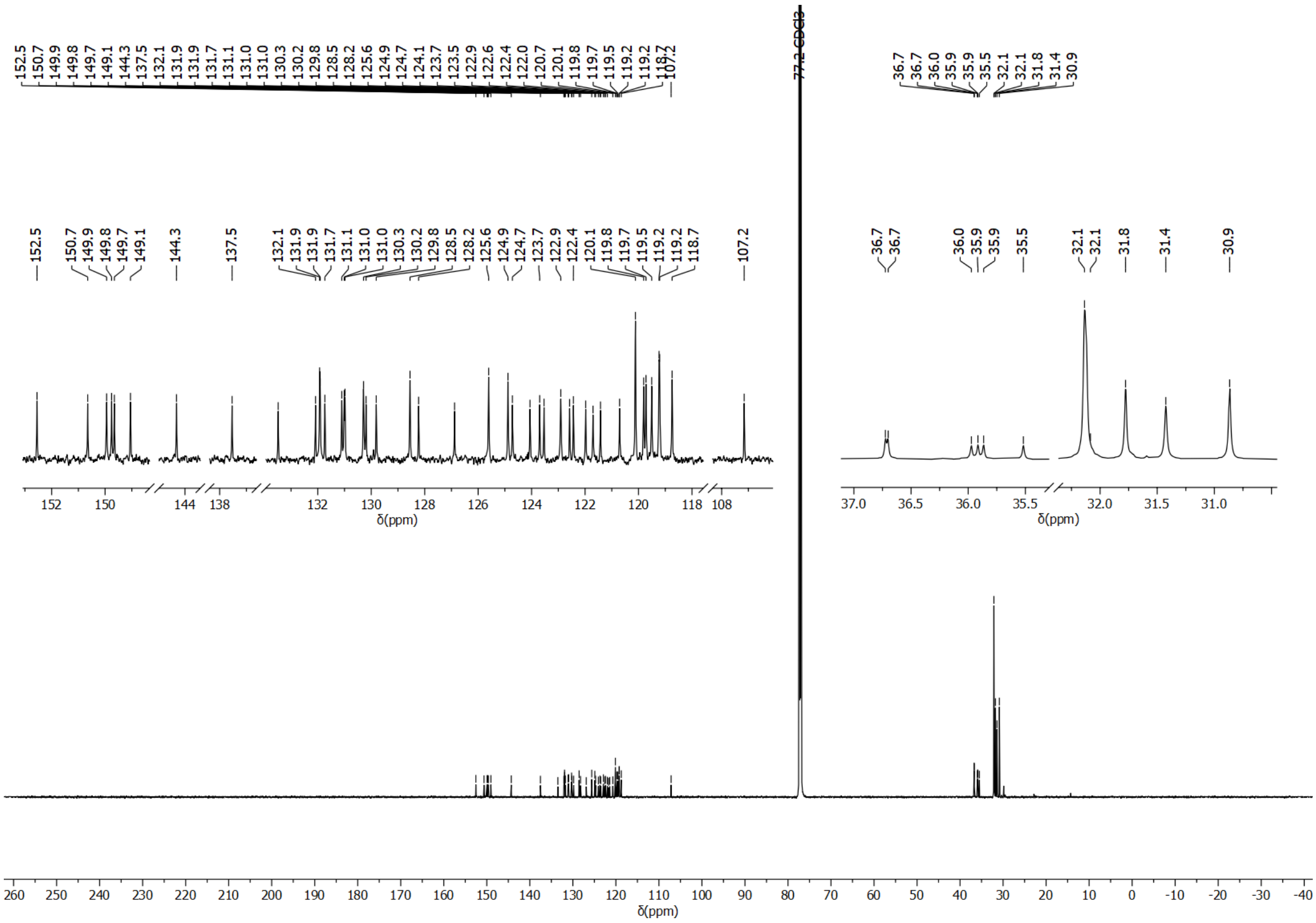

**Figure S60.** $^{13}$C NMR spectrum (176 MHz, $CDCl_3$, rt) of compound **4b**.

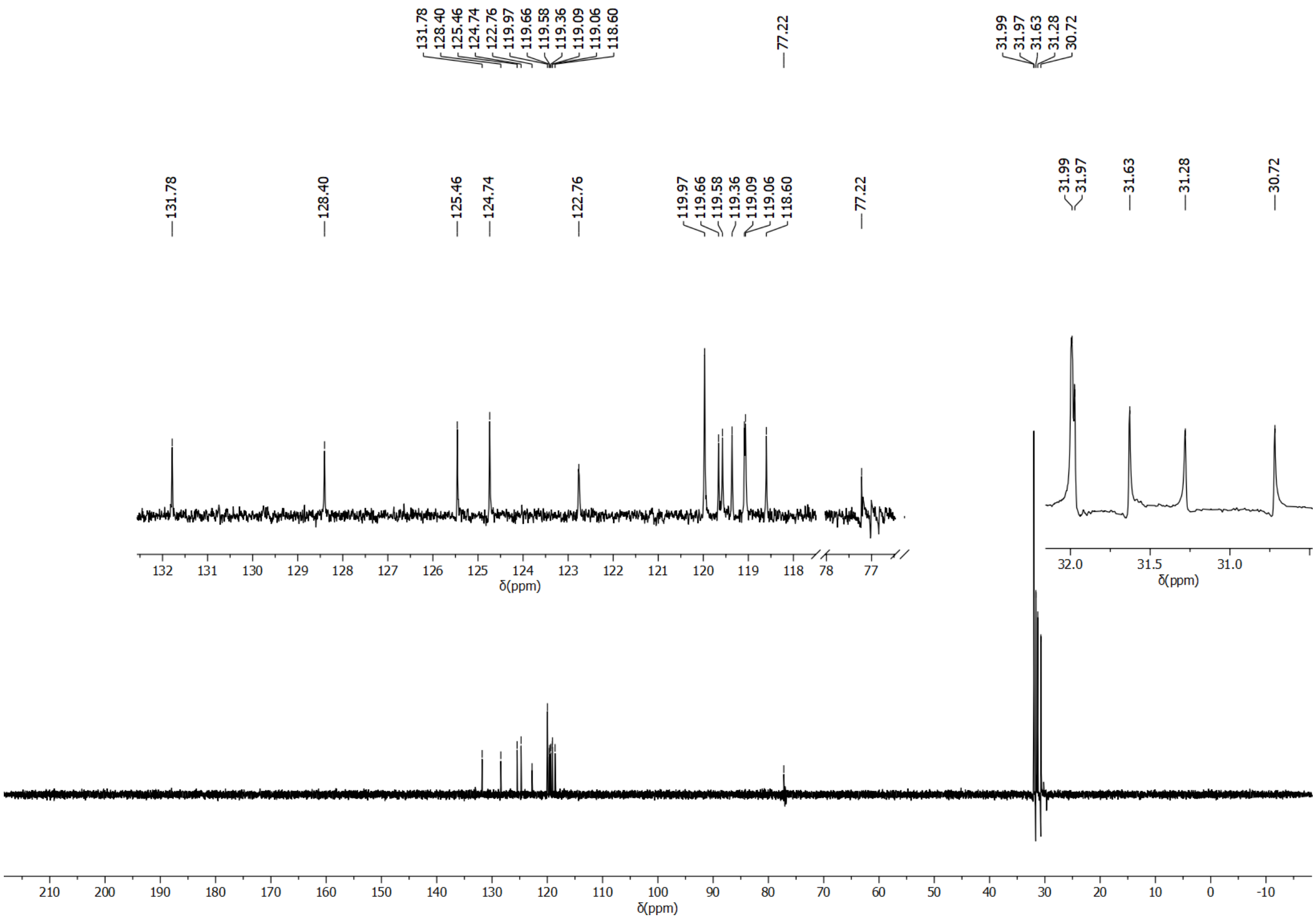

**Figure S61.** $^{13}$C DEPT135 NMR spectrum (176 MHz, $CDCl_3$, rt) of compound **4b**.

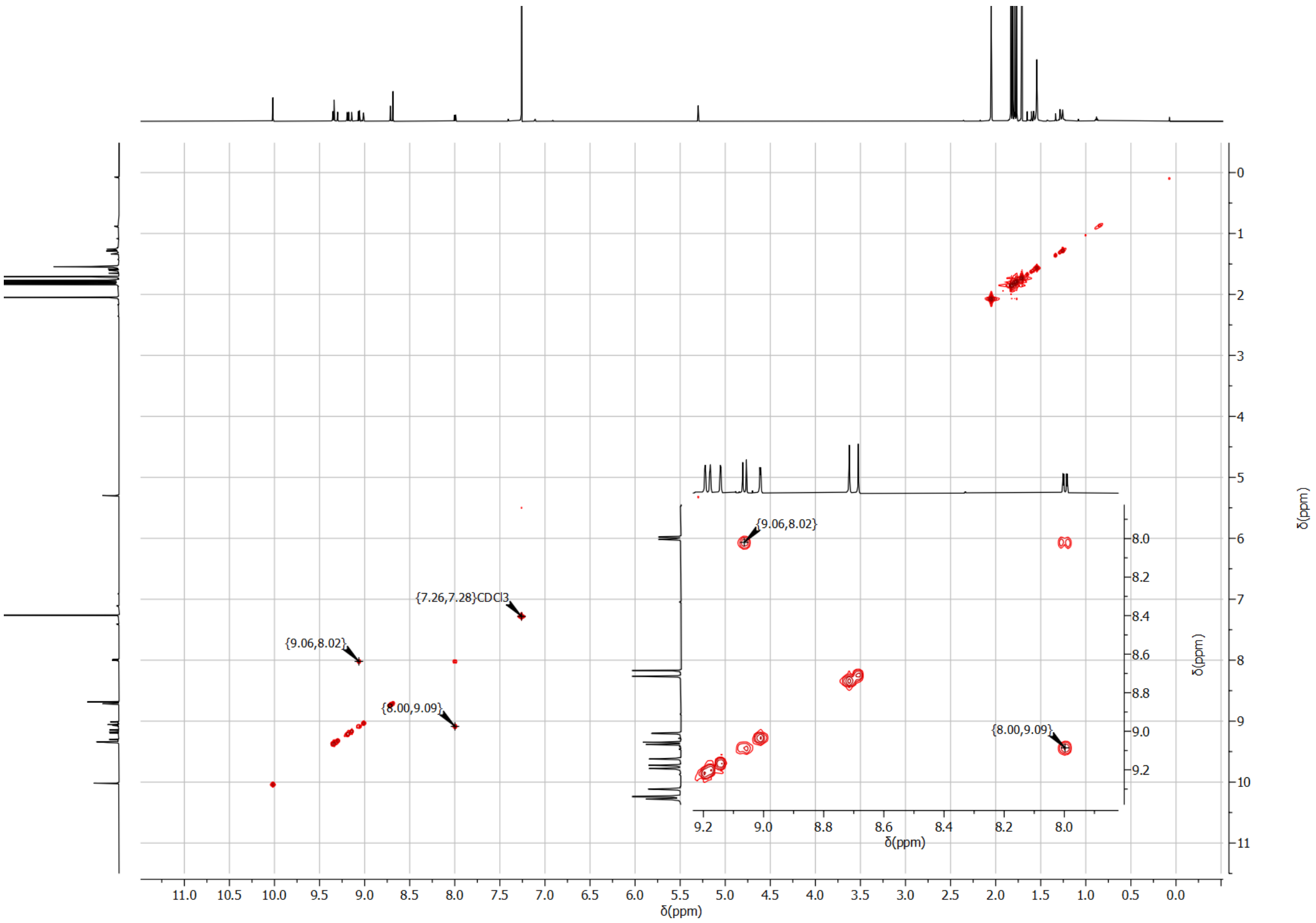

**Figure S62.** $^1H$-$^1H$ COSY NMR spectrum (700, 700 MHz, $CDCl_3$, rt) of compound **4b**.

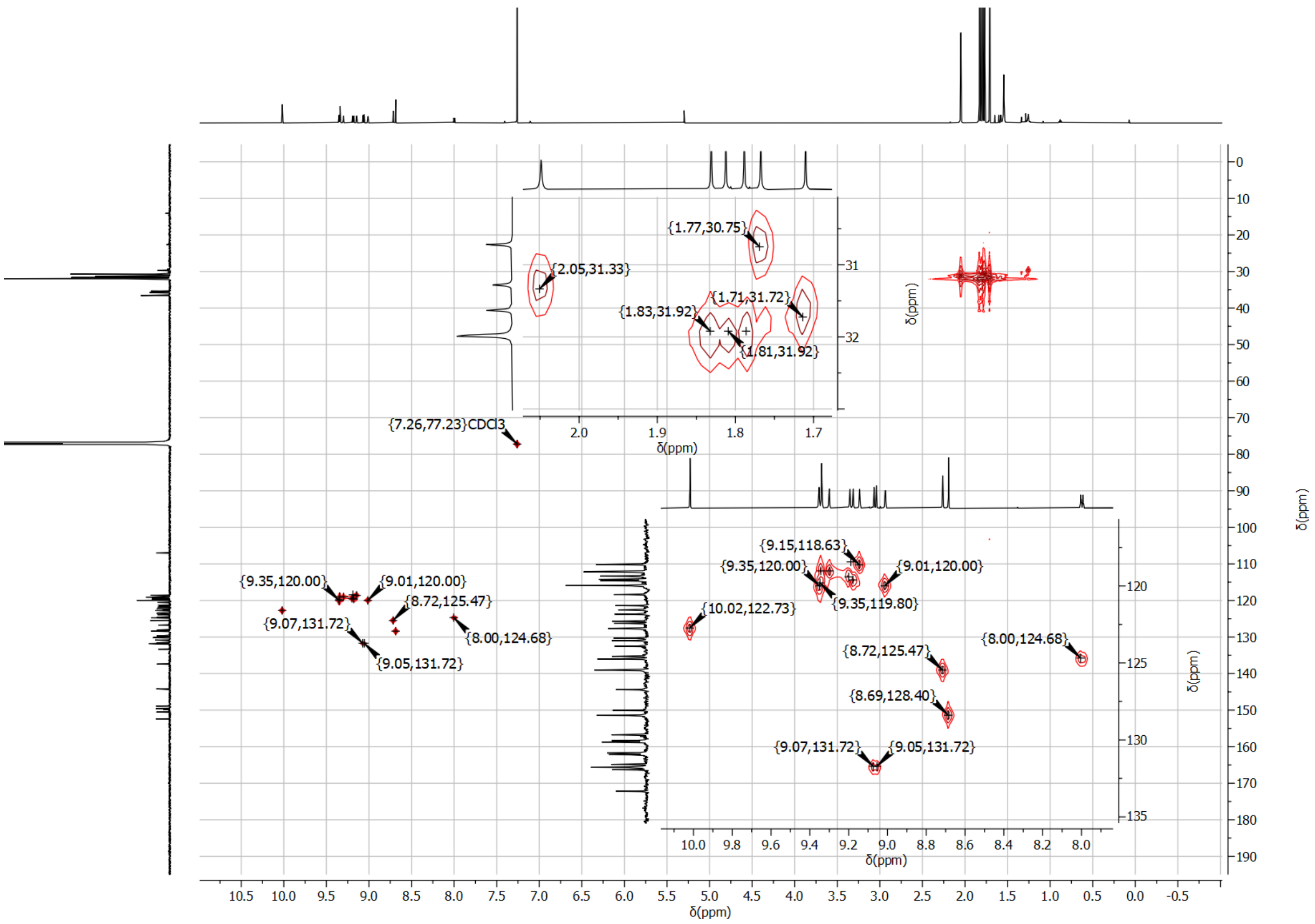

**Figure S63.** $^1H$-$^{13}C$ HMQC NMR spectrum (700, 176 MHz, $CDCl_3$, rt) of compound **4b**.

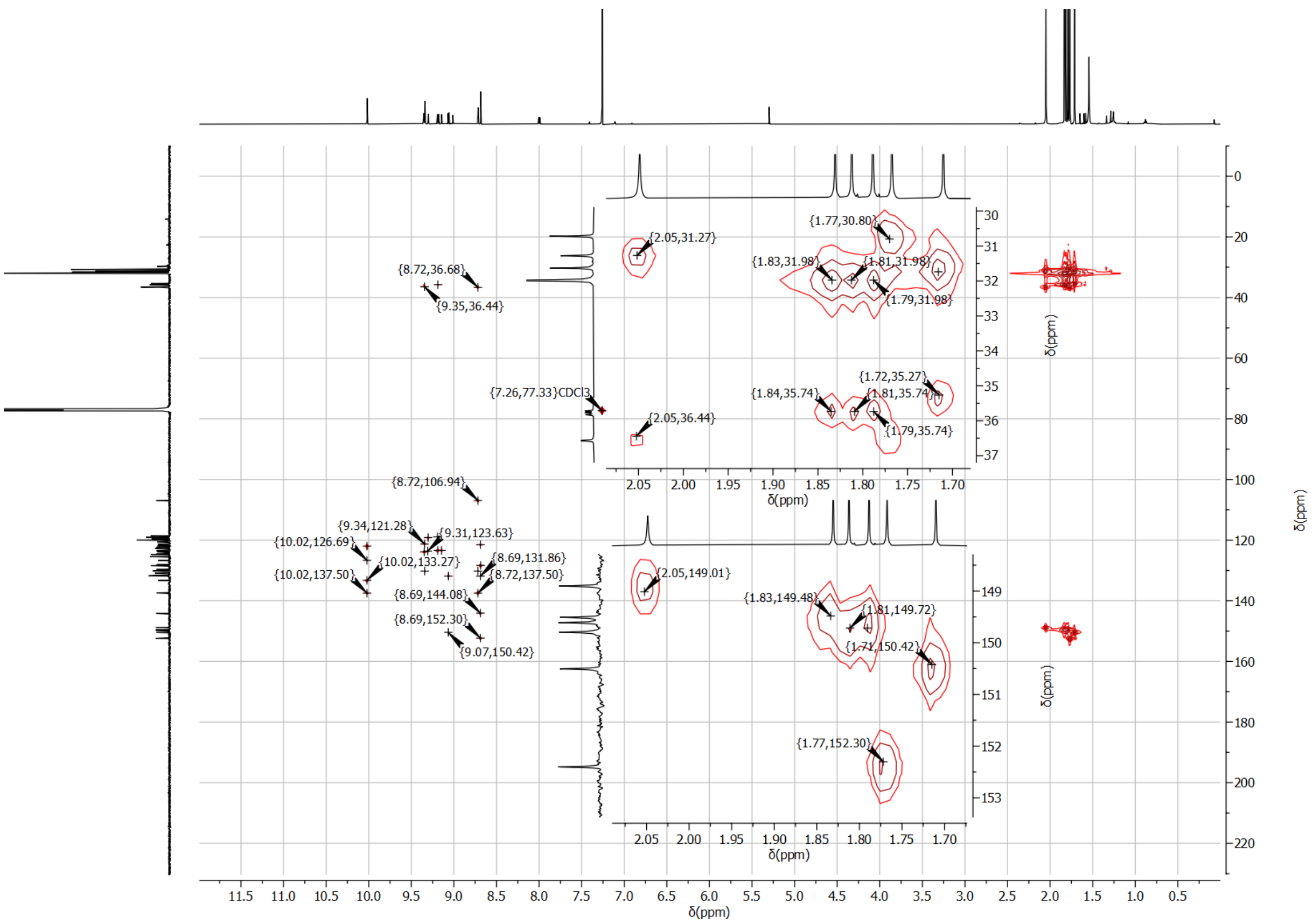

**Figure S64.** $^{1}H$-$^{13}C$ HMBC NMR spectrum (700, 176 MHz, $CDCl_3$, rt) of compound **4b**.

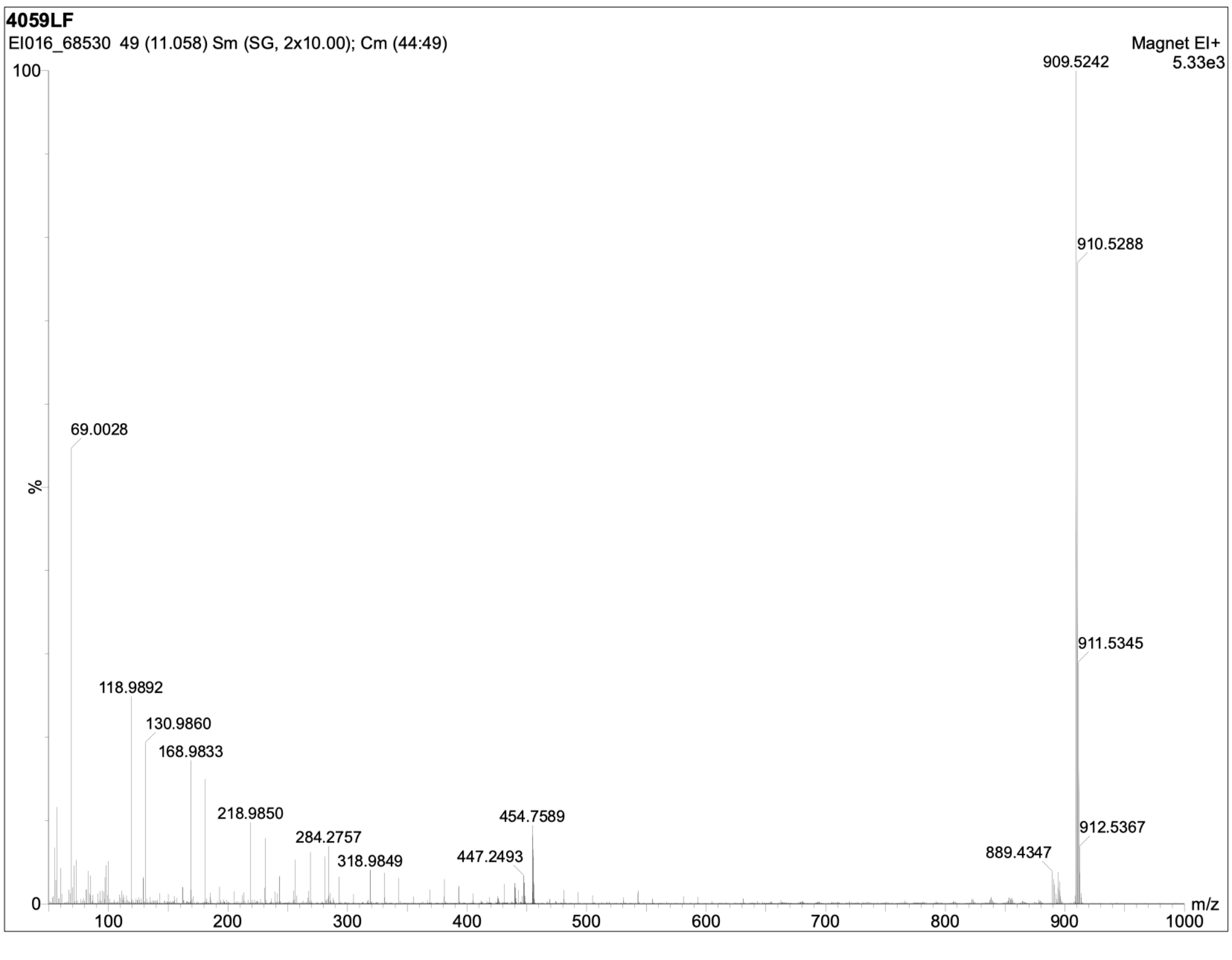


**Figure S65.** EI-HRMS spectrum of compound **4b**.

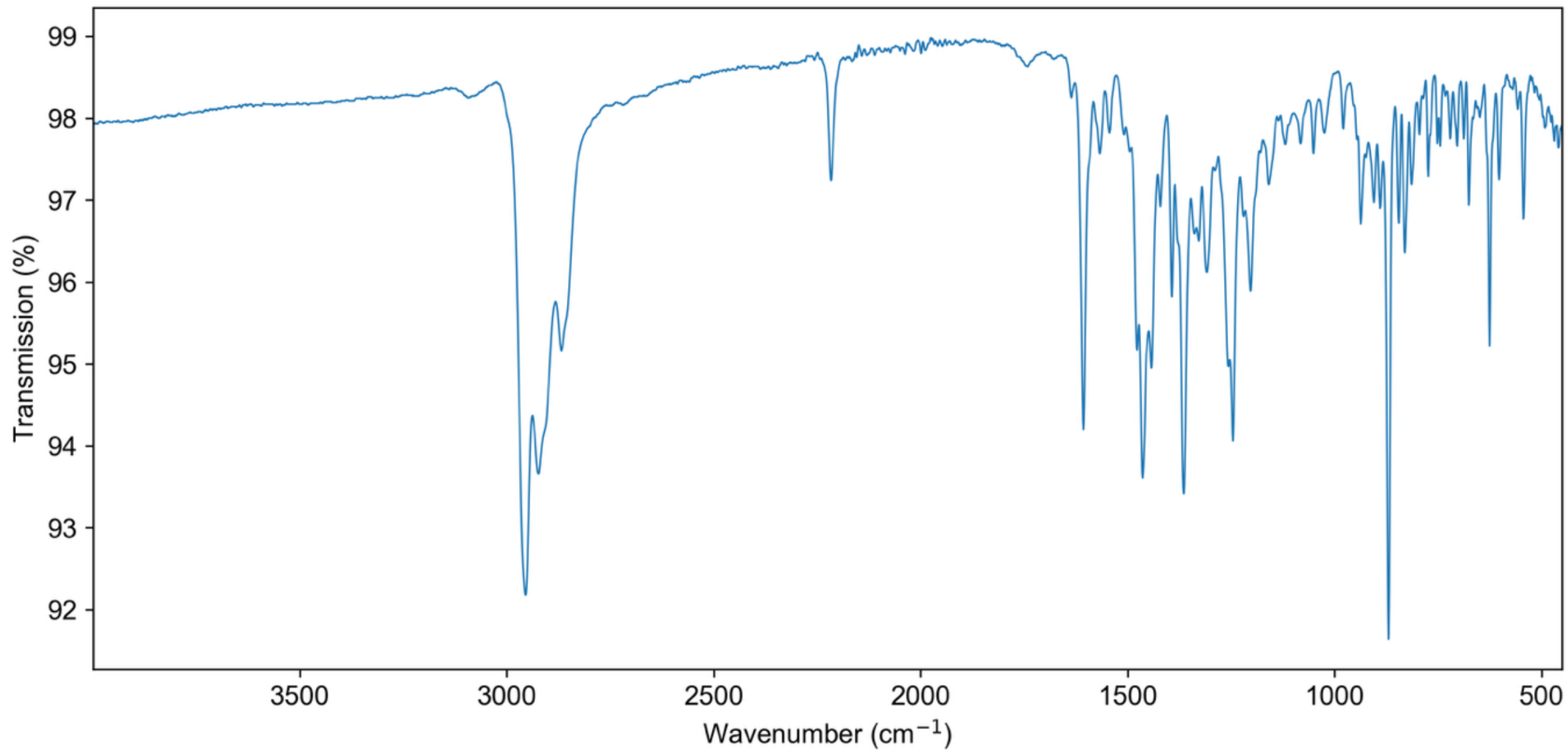


**Figure S66.** FT-IR spectrum of compound **4b**.

# 11 Optimized Geometries

**Br2.out**

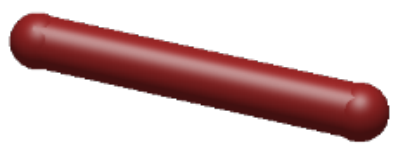

`Basis set: def2-TZVP`

Charge = 0, Multiplicity = 1

Electronic Energy = -5148.129827123738 Hartree

| | Cartesian Coordinates | | | | Cartesian Coordinates | | |
|---|---|---|---|---|---|---|---|
| **Atoms** | ***X*** | ***Y*** | ***Z*** | **Atoms** | ***X*** | ***Y*** | ***Z*** |
| Br | -4.261157 | 3.430937 | -0.000001 | Br | -2.030503 | 2.980185 | -0.000005 |

**HBr.out**

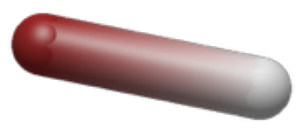

`Basis set: def2-TZVP`

Charge = 0, Multiplicity = 1

Electronic Energy = -2574.677153934227 Hartree

| | Cartesian Coordinates | | | | Cartesian Coordinates | | |
|---|---|---|---|---|---|---|---|
| **Atoms** | ***X*** | ***Y*** | ***Z*** | **Atoms** | ***X*** | ***Y*** | ***Z*** |
| Br | -3.842084 | 3.346255 | -0.000002 | H | -2.449575 | 3.064868 | -0.000004 |

**E.out**

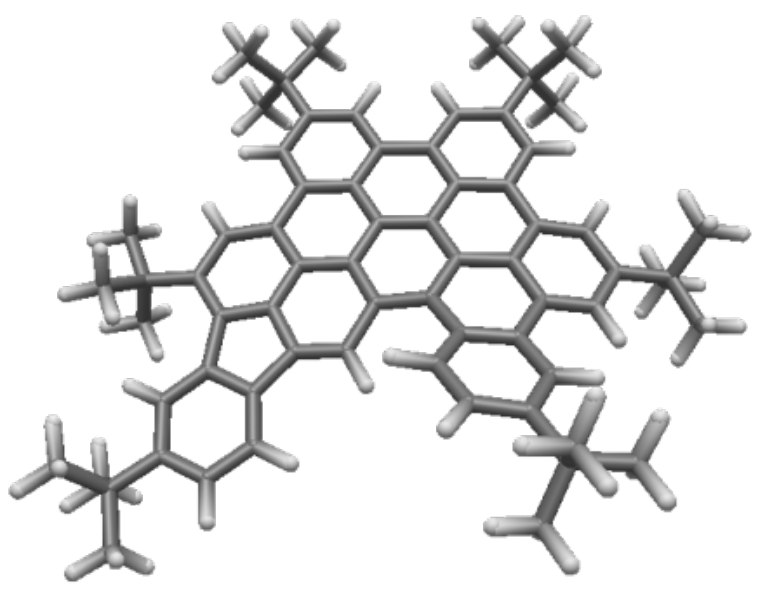

`Basis set: def2-TZVP`

Charge = 0, Multiplicity = 1

Electronic Energy = -2634.763238121755 Hartree

| | Cartesian Coordinates | | | | Cartesian Coordinates | | |
|---|---|---|---|---|---|---|---|
| **Atoms** | ***X*** | ***Y*** | ***Z*** | **Atoms** | ***X*** | ***Y*** | ***Z*** |
| C | -0.063124 | 0.182033 | 0.108610 | C | 1.465116 | 0.163116 | 0.105369 |
| C | 2.177975 | 1.374629 | 0.112881 | C | 3.551927 | 1.390812 | 0.083909 |
| C | 4.307225 | 0.201233 | 0.032909 | C | 5.773506 | 0.205769 | 0.002405 |
| C | 6.446205 | -0.921974 | 0.496548 | C | 7.847270 | -0.885307 | 0.741040 |
| C | 8.571175 | 0.273301 | 0.453824 | C | 7.920439 | 1.345555 | -0.196796 |

| | | | | | | | |
|---|---|---|---|---|---|---|---|
| C | 6.537414 | 1.307207 | -0.486256 | C | 5.973762 | 2.341005 | -1.334397 |
| C | 6.730593 | 3.392697 | -1.727093 | C | 8.098774 | 3.481631 | -1.323439 |
| C | 8.704869 | 2.476519 | -0.592322 | C | 10.071121 | 2.619343 | -0.296451 |
| C | 10.711827 | 3.772619 | -0.750179 | C | 10.099191 | 4.793242 | -1.505728 |
| C | 8.737728 | 4.632970 | -1.815612 | C | 7.678929 | 5.357080 | -2.604539 |
| C | 6.484911 | 4.586617 | -2.551045 | C | 5.338952 | 5.005371 | -3.196808 |
| C | 5.355165 | 6.201194 | -3.914997 | C | 6.506962 | 6.980519 | -3.987257 |
| C | 7.662883 | 6.543105 | -3.323355 | H | 8.545210 | 7.159342 | -3.389445 |
| C | 6.566758 | 8.299151 | -4.763586 | C | 5.235248 | 8.635783 | -5.440525 |
| H | 5.335583 | 9.580527 | -5.980931 | H | 4.944090 | 7.865749 | -6.159775 |
| H | 4.429652 | 8.749800 | -4.710399 | C | 6.920248 | 9.442407 | -3.798076 |
| H | 6.963721 | 10.390239 | -4.342471 | H | 7.890110 | 9.281966 | -3.321624 |
| H | 6.164722 | 9.531050 | -3.012369 | C | 7.649042 | 8.198966 | -5.851415 |
| H | 7.418749 | 7.389501 | -6.549718 | H | 7.700214 | 9.135486 | -6.414322 |
| H | 8.635198 | 8.008778 | -5.421803 | H | 4.450018 | 6.515329 | -4.418310 |
| H | 4.432143 | 4.410664 | -3.147715 | C | 10.931929 | 5.998709 | -1.954619 |
| C | 12.384869 | 5.947132 | -1.461173 | H | 12.901556 | 6.844165 | -1.812574 |
| H | 12.445375 | 5.932472 | -0.370082 | H | 12.919958 | 5.079405 | -1.854650 |
| C | 10.996071 | 6.023816 | -3.492857 | H | 11.609199 | 5.189417 | -3.844512 |
| H | 11.459040 | 6.954235 | -3.834096 | H | 10.020689 | 5.931255 | -3.965002 |
| C | 10.319290 | 7.282482 | -1.364961 | H | 9.247998 | 7.363327 | -1.534059 |
| H | 10.480716 | 7.298823 | -0.283484 | H | 10.805545 | 8.162844 | -1.794991 |
| H | 11.759317 | 3.887881 | -0.520402 | C | 10.729725 | 1.535073 | 0.446845 |
| C | 9.981778 | 0.371238 | 0.772128 | C | 10.626758 | -0.700421 | 1.418492 |
| C | 9.877819 | -1.936382 | 1.689412 | C | 8.520034 | -2.025009 | 1.320706 |
| C | 7.817793 | -3.232925 | 1.532466 | C | 8.467448 | -4.295079 | 2.146347 |
| C | 9.803942 | -4.219888 | 2.540779 | C | 10.488949 | -3.041401 | 2.294791 |
| H | 11.525780 | -2.975656 | 2.583622 | C | 10.454647 | -5.427122 | 3.219621 |
| C | 9.685563 | -5.756509 | 4.509364 | H | 10.141461 | -6.618698 | 5.004461 |
| H | 9.710771 | -4.909285 | 5.200383 | H | 8.640636 | -5.998378 | 4.303009 |
| C | 10.397600 | -6.634421 | 2.269407 | H | 10.861074 | -7.504016 | 2.744125 |
| H | 10.935949 | -6.420413 | 1.341905 | H | 9.368340 | -6.896516 | 2.014486 |
| C | 11.919425 | -5.169681 | 3.584210 | H | 12.020322 | -4.331866 | 4.279284 |
| H | 12.330184 | -6.059394 | 4.068009 | H | 12.524152 | -4.959455 | 2.697959 |
| H | 7.922645 | -5.208385 | 2.341208 | C | 6.410729 | -3.331572 | 1.105824 |
| C | 5.726806 | -2.166974 | 0.689478 | C | 4.357958 | -2.253661 | 0.370807 |
| C | 3.731126 | -3.504179 | 0.362839 | C | 4.408082 | -4.664789 | 0.702127 |
| C | 5.742708 | -4.549985 | 1.091962 | H | 6.277210 | -5.448388 | 1.368169 |
| C | 3.749556 | -6.045498 | 0.674254 | C | 2.287198 | -5.981900 | 0.224659 |
| H | 1.869257 | -6.991723 | 0.219061 | H | 1.683377 | -5.372200 | 0.902142 |
| H | 2.193575 | -5.573693 | -0.785225 | C | 4.517093 | -6.951597 | -0.302182 |
| H | 4.055606 | -7.942839 | -0.331168 | H | 5.560380 | -7.072551 | -0.002263 |
| H | 4.497923 | -6.533077 | -1.312316 | C | 3.795866 | -6.658905 | 2.083261 |
| H | 3.258135 | -6.027572 | 2.796153 | H | 3.325628 | -7.646414 | 2.074750 |

| | | | | | | | |
|---|---|---|---|---|---|---|---|
| H | 4.822366 | -6.776313 | 2.437499 | H | 2.693015 | -3.567139 | 0.074656 |
| C | 3.610890 | -1.012405 | 0.141028 | C | 2.202428 | -1.002860 | 0.145396 |
| H | 1.680300 | -1.944331 | 0.222513 | C | 11.977668 | -0.572535 | 1.768422 |
| C | 12.709239 | 0.569926 | 1.488220 | C | 12.064834 | 1.609999 | 0.816708 |
| H | 12.627119 | 2.502721 | 0.576933 | C | 14.180940 | 0.725630 | 1.877117 |
| C | 14.728134 | -0.513169 | 2.591588 | H | 15.777810 | -0.346081 | 2.845805 |
| H | 14.185999 | -0.716497 | 3.519019 | H | 14.673400 | -1.401424 | 1.956474 |
| C | 15.019150 | 0.963358 | 0.610520 | H | 16.073449 | 1.076500 | 0.878683 |
| H | 14.705144 | 1.867700 | 0.084793 | H | 14.927475 | 0.118096 | -0.077294 |
| C | 14.326542 | 1.932551 | 2.818327 | H | 15.374850 | 2.057970 | 3.104092 |
| H | 13.992865 | 2.856093 | 2.339887 | H | 13.736590 | 1.785497 | 3.727263 |
| H | 12.468989 | -1.389502 | 2.272610 | H | 4.957859 | 2.239126 | -1.689019 |
| H | 4.056946 | 2.344413 | 0.149631 | H | 1.646859 | 2.318656 | 0.162062 |
| C | -0.555631 | 0.938391 | -1.136093 | H | -1.648977 | 0.966548 | -1.146625 |
| H | -0.189769 | 1.967412 | -1.149233 | H | -0.215007 | 0.441988 | -2.049101 |
| C | -0.662926 | -1.226400 | 0.087328 | H | -1.753099 | -1.152015 | 0.064537 |
| H | -0.384750 | -1.797431 | 0.977160 | H | -0.344063 | -1.785503 | -0.796479 |
| C | -0.559983 | 0.903336 | 1.372075 | H | -0.217893 | 0.384207 | 2.271719 |
| H | -0.199914 | 1.933633 | 1.413535 | H | -1.653516 | 0.925911 | 1.382006 |

**TS11.out**

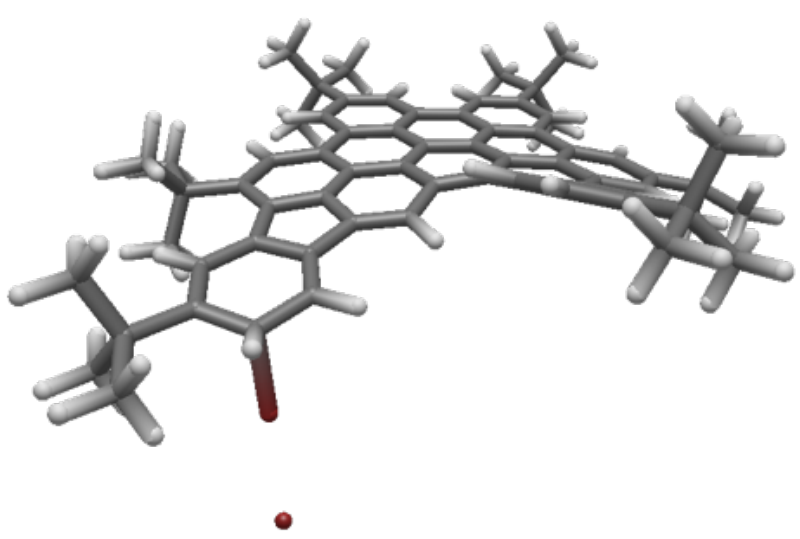

`Basis set: def2-TZVP`

Charge = 0, Multiplicity = 1

Electronic Energy = -7782.871323128860 Hartree

| | Cartesian Coordinates | | | | Cartesian Coordinates | | |
|---|---|---|---|---|---|---|---|
| **Atoms** | ***X*** | ***Y*** | ***Z*** | **Atoms** | ***X*** | ***Y*** | ***Z*** |
| C | -7.528047 | -0.667113 | 0.086132 | C | -5.993469 | -0.616058 | 0.081326 |
| C | -5.292595 | 0.604298 | 0.079575 | C | -3.910613 | 0.634386 | 0.097665 |
| C | -3.138098 | -0.547256 | 0.108189 | C | -1.670388 | -0.530696 | 0.108060 |
| C | -0.991852 | -1.643540 | 0.643897 | C | 0.407565 | -1.583290 | 0.917777 |
| C | 1.129122 | -0.418441 | 0.615584 | C | 0.478721 | 0.632443 | -0.073892 |
| C | -0.902824 | 0.569707 | -0.393712 | C | -1.451960 | 1.572467 | -1.280965 |
| C | -0.693044 | 2.633720 | -1.676488 | C | 0.656006 | 2.753354 | -1.244111 |
| C | 1.258419 | 1.763981 | -0.476185 | C | 2.620083 | 1.929316 | -0.160238 |
| C | 3.267630 | 3.092793 | -0.621434 | C | 2.675126 | 4.096310 | -1.400617 |
| C | 1.300415 | 3.913397 | -1.741017 | C | 0.286601 | 4.600223 | -2.544913 |
| C | -0.924133 | 3.800843 | -2.532736 | C | -2.013961 | 4.175977 | -3.251710 |
| C | -1.962790 | 5.357219 | -4.101313 | C | -0.806051 | 6.245051 | -3.974425 |
| C | 0.291005 | 5.820352 | -3.251794 | H | 1.165463 | 6.462957 | -3.219549 |

| | | | |
|---|---|---|---|
| C | -0.780827 | 7.620276 | -4.645307 |
| H | -2.018049 | 8.967535 | -5.793537 |
| H | -2.941664 | 8.015693 | -4.617050 |
| H | -0.523860 | 9.680082 | -4.007771 |
| H | -1.287271 | 8.644641 | -2.773386 |
| H | 0.190363 | 6.899687 | -6.475026 |
| H | 1.336208 | 7.462587 | -5.229523 |
| H | -2.925747 | 3.572803 | -3.258636 |
| C | 4.969595 | 5.229929 | -1.343337 |
| H | 5.031043 | 5.219835 | -0.244738 |
| C | 3.594264 | 5.321559 | -3.387692 |
| H | 4.093134 | 6.241536 | -3.729864 |
| C | 2.911516 | 6.588229 | -1.249898 |
| H | 3.069097 | 6.604066 | -0.160666 |
| H | 4.316484 | 3.214089 | -0.365125 |
| C | 2.532652 | -0.298226 | 0.951158 |
| C | 2.442744 | -2.592102 | 1.918667 |
| C | 0.388686 | -3.916016 | 1.768786 |
| C | 2.387003 | -4.870728 | 2.811863 |
| H | 4.102555 | -3.596399 | 2.850347 |
| C | 2.274928 | -6.388949 | 4.800626 |
| H | 2.274949 | -5.526718 | 5.485827 |
| C | 3.021292 | -7.281048 | 2.567873 |
| H | 3.567249 | -7.068286 | 1.635331 |
| C | 4.511839 | -5.774395 | 3.882939 |
| H | 4.947968 | -6.654829 | 4.378606 |
| H | 0.508606 | -5.888042 | 2.609876 |
| C | -1.703961 | -2.890455 | 0.864322 |
| C | -3.695539 | -4.255360 | 0.574525 |
| C | -1.683143 | -5.261418 | 1.371550 |
| C | -3.677633 | -6.788760 | 1.017302 |
| H | -5.560684 | -7.761494 | 0.567105 |
| H | -5.203472 | -6.396897 | -0.512356 |
| H | -3.344908 | -8.751632 | 0.128200 |
| H | -2.875265 | -7.393960 | -0.927698 |
| H | -4.229979 | -6.634906 | 3.129665 |
| H | -2.641252 | -7.394407 | 2.863269 |
| C | -3.828197 | -1.773184 | 0.236618 |
| H | -5.757151 | -2.727955 | 0.279635 |
| C | 5.267259 | -0.058819 | 1.688642 |
| H | 5.181402 | 1.856002 | 0.718369 |
| C | 7.255138 | -1.077991 | 2.907326 |
| H | 6.686951 | -1.193855 | 3.843372 |
| C | 7.584805 | 0.196359 | 0.786147 |

| | | | |
|---|---|---|---|
| C | -2.106901 | 7.972512 | -5.333712 |
| H | -2.364535 | 7.261301 | -6.131390 |
| C | -0.511663 | 8.677550 | -3.553775 |
| H | 0.467387 | 8.540046 | -3.071825 |
| C | 0.354278 | 7.651214 | -5.688502 |
| H | 0.389859 | 8.643745 | -6.162794 |
| H | -2.931308 | 5.855781 | -4.246954 |
| C | 3.518161 | 5.300383 | -1.846286 |
| H | 5.508738 | 6.122068 | -1.695561 |
| H | 5.501248 | 4.348508 | -1.732193 |
| H | 4.184907 | 4.462882 | -3.741411 |
| H | 2.618826 | 5.263031 | -3.884430 |
| H | 1.832062 | 6.689150 | -1.414209 |
| H | 3.404970 | 7.474560 | -1.678064 |
| C | 3.278313 | 0.867781 | 0.607228 |
| C | 3.183308 | -1.353738 | 1.629561 |
| C | 1.084080 | -2.702284 | 1.535561 |
| C | 1.049993 | -4.963987 | 2.406363 |
| C | 3.060673 | -3.681452 | 2.551959 |
| C | 3.052214 | -6.064581 | 3.511634 |
| H | 2.740848 | -7.240615 | 5.320998 |
| H | 1.228068 | -6.656708 | 4.591933 |
| H | 3.495431 | -8.150070 | 3.050914 |
| H | 1.992549 | -7.566942 | 2.301056 |
| H | 4.597797 | -4.924594 | 4.577811 |
| H | 5.125656 | -5.555986 | 2.995358 |
| C | -1.015582 | -4.037254 | 1.335185 |
| C | -3.073601 | -2.999002 | 0.531276 |
| C | -3.018394 | -5.402796 | 0.976542 |
| H | -1.142829 | -6.149508 | 1.699992 |
| C | -5.130573 | -6.749274 | 0.528243 |
| H | -5.758130 | -6.098767 | 1.156906 |
| C | -2.884685 | -7.751023 | 0.114073 |
| H | -1.840727 | -7.859865 | 0.444870 |
| C | -3.664289 | -7.308374 | 2.466752 |
| H | -4.128244 | -8.306128 | 2.518021 |
| H | -4.740352 | -4.333469 | 0.281535 |
| C | -5.235616 | -1.776336 | 0.188118 |
| C | 4.531895 | -1.202596 | 1.989581 |
| C | 4.618181 | 0.960518 | 0.984150 |
| C | 6.744579 | 0.103842 | 2.073337 |
| H | 8.309047 | -0.909472 | 3.175407 |
| H | 7.203702 | -2.027561 | 2.352524 |
| H | 8.651773 | 0.311675 | 1.034450 |

| | | | | | | | |
|---|---|---|---|---|---|---|---|
| H | 7.290868 | 1.057472 | 0.166453 | H | 7.471864 | -0.714215 | 0.177181 |
| C | 6.916420 | 1.392423 | 2.897811 | H | 7.972269 | 1.522401 | 3.182751 |
| H | 6.613722 | 2.286585 | 2.332284 | H | 6.315560 | 1.354345 | 3.820076 |
| H | 5.027673 | -2.012138 | 2.520046 | H | -2.463564 | 1.454157 | -1.668091 |
| H | -3.419507 | 1.605963 | 0.151129 | H | -5.831959 | 1.552948 | 0.083866 |
| C | -8.127477 | 0.568542 | -0.603022 | H | -9.222867 | 0.471619 | -0.651724 |
| H | -7.908670 | 1.498298 | -0.057728 | H | -7.750153 | 0.677018 | -1.632214 |
| C | -8.040295 | -1.917860 | -0.646834 | H | -9.140317 | -1.901383 | -0.686334 |
| H | -7.749584 | -2.850260 | -0.141100 | H | -7.662781 | -1.956946 | -1.680792 |
| C | -7.999407 | -0.707646 | 1.552371 | H | -7.605219 | -1.597177 | 2.068135 |
| H | -7.656810 | 0.182912 | 2.102301 | H | -9.099642 | -0.740241 | 1.605018 |
| Br | -1.704117 | 4.478995 | -5.987902 | Br | -1.392501 | 3.359019 | -8.643604 |

**TS12.out**

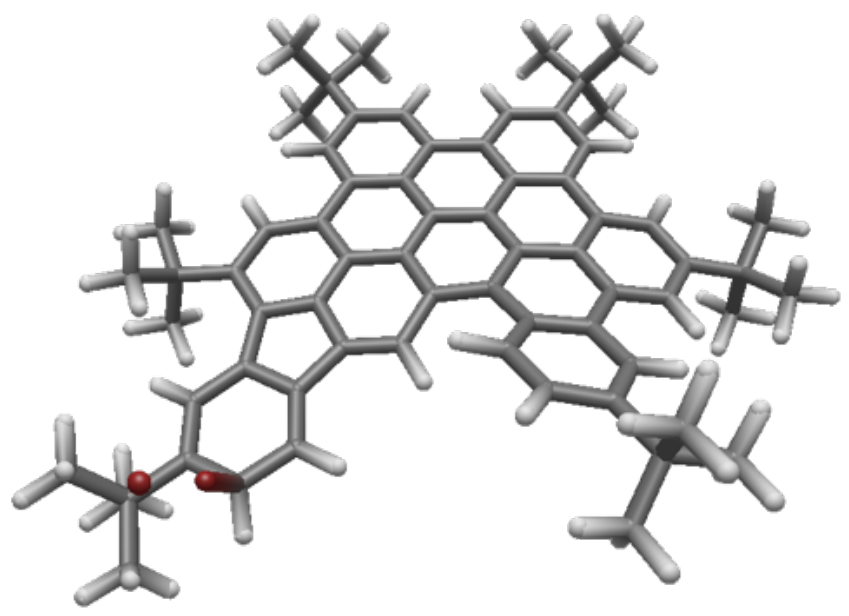

Basis set: def2-TZVP

Charge = 0, Multiplicity = 1

Electronic Energy = -7782.871805699803 Hartree

| | Cartesian Coordinates | | | | Cartesian Coordinates | | |
|---|---|---|---|---|---|---|---|
| Atoms | *X* | *Y* | *Z* | Atoms | *X* | *Y* | *Z* |
| C | -0.142567 | 0.009481 | -0.071927 | C | 1.389611 | 0.026093 | -0.008007 |
| C | 2.079983 | 1.254691 | 0.035512 | C | 3.458960 | 1.298553 | 0.052680 |
| C | 4.242109 | 0.121226 | 0.019864 | C | 5.709646 | 0.150182 | 0.001542 |
| C | 6.404699 | -0.971513 | 0.496480 | C | 7.808431 | -0.909631 | 0.746270 |
| C | 8.518901 | 0.264962 | 0.455353 | C | 7.850186 | 1.327461 | -0.197457 |
| C | 6.461468 | 1.267440 | -0.486623 | C | 5.887635 | 2.293522 | -1.330512 |
| C | 6.635429 | 3.363985 | -1.721853 | C | 7.996344 | 3.473277 | -1.326334 |
| C | 8.617686 | 2.469981 | -0.592373 | C | 9.983144 | 2.637234 | -0.294902 |
| C | 10.614883 | 3.816442 | -0.738105 | C | 10.003415 | 4.833284 | -1.484609 |
| C | 8.625833 | 4.646400 | -1.811469 | C | 7.592944 | 5.345475 | -2.579999 |
| C | 6.374492 | 4.560312 | -2.527381 | C | 5.239908 | 4.991754 | -3.137166 |
| C | 5.208394 | 6.287627 | -3.800142 | C | 6.474603 | 6.999451 | -3.980243 |
| C | 7.601976 | 6.540620 | -3.328996 | H | 8.517860 | 7.115375 | -3.422844 |
| C | 6.566414 | 8.253449 | -4.853400 | C | 5.259195 | 8.561462 | -5.596631 |
| H | 5.400631 | 9.463863 | -6.209084 | H | 4.972839 | 7.744456 | -6.276145 |
| H | 4.424258 | 8.757113 | -4.908841 | C | 6.939359 | 9.458261 | -3.965503 |
| H | 7.021035 | 10.361651 | -4.589015 | H | 7.904883 | 9.308681 | -3.459679 |
| H | 6.172340 | 9.639158 | -3.198232 | C | 7.672862 | 8.026693 | -5.904821 |
| H | 7.451549 | 7.146603 | -6.527694 | H | 7.732958 | 8.906780 | -6.562985 |
| H | 8.661690 | 7.884579 | -5.445432 | H | 4.501515 | 6.354901 | -4.638949 |

| | | | | | | | |
|---|---|---|---|---|---|---|---|
| H | 4.314869 | 4.410567 | -3.093801 | C | 10.830292 | 6.055446 | -1.910990 |
| C | 12.268340 | 6.020613 | -1.367257 | H | 12.792691 | 6.929711 | -1.697827 |
| H | 12.296367 | 6.003696 | -0.267310 | H | 12.837351 | 5.158009 | -1.745092 |
| C | 10.945996 | 6.067975 | -3.450234 | H | 11.580873 | 5.232006 | -3.780996 |
| H | 11.413014 | 7.005557 | -3.789854 | H | 9.985874 | 5.960863 | -3.968665 |
| C | 10.181963 | 7.333665 | -1.339017 | H | 9.109790 | 7.424126 | -1.548877 |
| H | 10.294912 | 7.349958 | -0.244309 | H | 10.682146 | 8.226324 | -1.745482 |
| H | 11.665866 | 3.941253 | -0.492460 | C | 10.660004 | 1.563697 | 0.438798 |
| C | 9.927935 | 0.384626 | 0.766932 | C | 10.596693 | -0.682635 | 1.408569 |
| C | 9.865356 | -1.928285 | 1.690893 | C | 8.500221 | -2.036978 | 1.330099 |
| C | 7.812283 | -3.256610 | 1.555752 | C | 8.486561 | -4.310329 | 2.170252 |
| C | 9.828394 | -4.217070 | 2.558681 | C | 10.495800 | -3.023520 | 2.301134 |
| H | 11.541541 | -2.938838 | 2.586017 | C | 10.503758 | -5.414690 | 3.242105 |
| C | 9.740924 | -5.748281 | 4.537462 | H | 10.213940 | -6.601825 | 5.048308 |
| H | 9.746180 | -4.889916 | 5.227495 | H | 8.692506 | -6.017092 | 4.338150 |
| C | 10.463849 | -6.625141 | 2.291091 | H | 10.941294 | -7.497491 | 2.764785 |
| H | 11.001951 | -6.407072 | 1.355192 | H | 9.432479 | -6.908545 | 2.031510 |
| C | 11.966763 | -5.124468 | 3.599617 | H | 12.058585 | -4.279691 | 4.299855 |
| H | 12.409637 | -6.007615 | 4.084408 | H | 12.570896 | -4.898581 | 2.707250 |
| H | 7.951410 | -5.238597 | 2.370917 | C | 6.403057 | -3.377204 | 1.139681 |
| C | 5.704341 | -2.227550 | 0.698918 | C | 4.330320 | -2.338853 | 0.376403 |
| C | 3.719506 | -3.597924 | 0.407296 | C | 4.405367 | -4.751130 | 0.787961 |
| C | 5.742845 | -4.609106 | 1.166304 | H | 6.297400 | -5.494779 | 1.469910 |
| C | 3.727492 | -6.129069 | 0.739665 | C | 2.229776 | -6.021617 | 1.071549 |
| H | 1.782923 | -7.027211 | 1.102044 | H | 2.070484 | -5.548636 | 2.053620 |
| H | 1.671954 | -5.444889 | 0.319005 | C | 3.893359 | -6.688516 | -0.686197 |
| H | 3.418094 | -7.679133 | -0.770734 | H | 4.958340 | -6.796250 | -0.945183 |
| H | 3.429297 | -6.021122 | -1.429334 | C | 4.366240 | -7.104868 | 1.740960 |
| H | 4.339239 | -6.703135 | 2.766382 | H | 3.813316 | -8.056554 | 1.734548 |
| H | 5.411962 | -7.338414 | 1.491613 | H | 2.672458 | -3.680707 | 0.122258 |
| C | 3.564475 | -1.112408 | 0.113468 | C | 2.154414 | -1.129209 | 0.060855 |
| H | 1.643839 | -2.088751 | 0.111527 | C | 11.953046 | -0.534856 | 1.740509 |
| C | 12.675494 | 0.621267 | 1.455555 | C | 12.004968 | 1.656921 | 0.796452 |
| H | 12.555212 | 2.566262 | 0.551743 | C | 14.153799 | 0.793506 | 1.832413 |
| C | 14.734344 | -0.470026 | 2.479430 | H | 15.793357 | -0.302144 | 2.727018 |
| H | 14.213001 | -0.730756 | 3.413429 | H | 14.683649 | -1.337776 | 1.803352 |
| C | 14.968194 | 1.111059 | 0.564754 | H | 16.034354 | 1.223898 | 0.816620 |
| H | 14.639208 | 2.046890 | 0.088287 | H | 14.874870 | 0.301323 | -0.175907 |
| C | 14.276318 | 1.958277 | 2.832225 | H | 15.330365 | 2.112090 | 3.112603 |
| H | 13.901548 | 2.901852 | 2.406785 | H | 13.704055 | 1.749416 | 3.749669 |
| H | 12.463453 | -1.355595 | 2.239000 | H | 4.864048 | 2.187881 | -1.688079 |
| H | 3.942029 | 2.271643 | 0.140562 | H | 1.526827 | 2.196665 | 0.071961 |
| C | -0.598288 | 0.807445 | -1.307361 | H | -1.697451 | 0.803674 | -1.379015 |
| H | -0.270709 | 1.856840 | -1.261347 | H | -0.194049 | 0.365962 | -2.231999 |

| | | | | | | | |
|---|---|---|---|---|---|---|---|
| C | -0.699917 | -1.415222 | -0.181568 | H | -1.797292 | -1.377194 | -0.255734 |
| H | -0.449364 | -2.025878 | 0.699645 | H | -0.321544 | -1.932277 | -1.077168 |
| C | -0.706766 | 0.662248 | 1.203337 | H | -0.390275 | 0.107706 | 2.100675 |
| H | -0.371299 | 1.704760 | 1.312317 | H | -1.807978 | 0.666253 | 1.174243 |
| Br | 4.190863 | 7.429797 | -2.365747 | Br | 2.742447 | 9.093861 | -0.485006 |

**TS21.out**

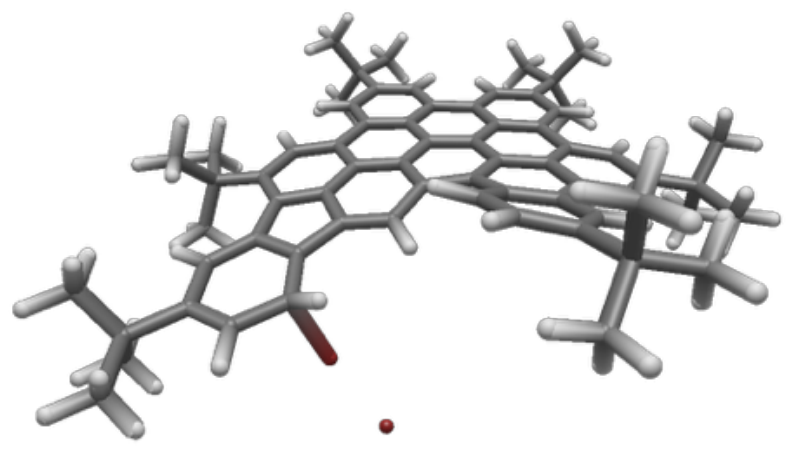

Basis set: def2-TZVP

Charge = 0, Multiplicity = 1

Electronic Energy = -7782.862766388584 Hartree

| | Cartesian Coordinates | | | | Cartesian Coordinates | | |
|---|---|---|---|---|---|---|---|
| Atoms | X | Y | Z | Atoms | X | Y | Z |
| C | -7.578755 | 0.725571 | 1.347655 | C | -6.068299 | 0.494601 | 1.217629 |
| C | -5.211972 | 1.579416 | 0.935308 | C | -3.853726 | 1.396016 | 0.783373 |
| C | -3.258008 | 0.116255 | 0.886691 | C | -1.821071 | -0.101324 | 0.703415 |
| C | -1.231429 | -1.232600 | 1.297883 | C | 0.190396 | -1.354388 | 1.384648 |
| C | 1.017236 | -0.355699 | 0.844502 | C | 0.428096 | 0.693941 | 0.101662 |
| C | -0.986213 | 0.800185 | -0.039422 | C | -1.513984 | 1.739864 | -0.988534 |
| C | -0.676098 | 2.625010 | -1.624061 | C | 0.730407 | 2.595518 | -1.371980 |
| C | 1.292219 | 1.636215 | -0.542882 | C | 2.695035 | 1.636177 | -0.410331 |
| C | 3.410706 | 2.617866 | -1.108788 | C | 2.843180 | 3.589963 | -1.963832 |
| C | 1.438028 | 3.562708 | -2.105301 | C | 0.377416 | 4.273335 | -2.885765 |
| C | -0.878198 | 3.669856 | -2.588347 | C | -2.089194 | 4.092539 | -3.248289 |
| C | -1.996381 | 5.282864 | -4.081261 | C | -0.797328 | 5.883113 | -4.363664 |
| C | 0.382427 | 5.347493 | -3.760072 | H | 1.326976 | 5.829863 | -4.001337 |
| C | -0.651889 | 7.084213 | -5.304669 | C | -2.014113 | 7.578920 | -5.803124 |
| H | -1.869162 | 8.446144 | -6.464287 | H | -2.544674 | 6.806556 | -6.381031 |
| H | -2.662693 | 7.896870 | -4.971831 | C | 0.048216 | 8.234654 | -4.558816 |
| H | 0.145556 | 9.107822 | -5.222101 | H | 1.061634 | 7.961321 | -4.226349 |
| H | -0.531128 | 8.540144 | -3.673501 | C | 0.193308 | 6.653154 | -6.517765 |
| H | -0.288542 | 5.820892 | -7.053953 | H | 0.307941 | 7.494568 | -7.218566 |
| H | 1.203013 | 6.328011 | -6.222093 | H | -2.929243 | 5.650175 | -4.511371 |
| H | -3.019419 | 3.971776 | -2.675898 | C | 3.762639 | 4.596170 | -2.681019 |
| C | 5.248253 | 4.349111 | -2.368024 | H | 5.849307 | 5.108588 | -2.889919 |
| H | 5.469743 | 4.435321 | -1.293700 | H | 5.586522 | 3.361547 | -2.717496 |
| C | 3.586234 | 4.481349 | -4.222453 | H | 4.565909 | 4.477287 | -4.721236 |
| H | 3.032904 | 5.327142 | -4.652126 | H | 3.067610 | 3.556642 | -4.515397 |
| C | 3.431187 | 6.014709 | -2.174617 | H | 2.369340 | 6.273359 | -2.287662 |
| H | 3.675489 | 6.106712 | -1.105205 | H | 4.021799 | 6.762900 | -2.726146 |

| | | | | | | | |
|---|---|---|---|---|---|---|---|
| H | 4.492146 | 2.617386 | -1.001273 | C | 3.300983 | 0.588783 | 0.424339 |
| C | 2.458591 | -0.409313 | 0.995375 | C | 3.045286 | -1.468520 | 1.723137 |
| C | 2.190368 | -2.547648 | 2.242122 | C | 0.787571 | -2.488292 | 2.053760 |
| C | -0.027087 | -3.549541 | 2.527357 | C | 0.573371 | -4.621164 | 3.184913 |
| C | 1.956852 | -4.698117 | 3.386918 | C | 2.740598 | -3.653808 | 2.907372 |
| H | 3.817316 | -3.701150 | 3.048762 | C | 2.550118 | -5.917791 | 4.106080 |
| C | 1.930125 | -6.029722 | 5.510939 | H | 2.349302 | -6.899304 | 6.041188 |
| H | 2.143798 | -5.129484 | 6.108286 | H | 0.838142 | -6.158940 | 5.469362 |
| C | 2.221101 | -7.183134 | 3.292070 | H | 2.642492 | -8.073667 | 3.784715 |
| H | 2.648292 | -7.119503 | 2.278939 | H | 1.135769 | -7.336665 | 3.193258 |
| C | 4.073348 | -5.815826 | 4.253563 | H | 4.370449 | -4.935245 | 4.844125 |
| H | 4.453503 | -6.708311 | 4.773161 | H | 4.578236 | -5.761754 | 3.276655 |
| H | -0.050764 | -5.430080 | 3.564407 | C | -1.486047 | -3.491404 | 2.317826 |
| C | -2.069192 | -2.313512 | 1.784419 | C | -3.475498 | -2.236602 | 1.661428 |
| C | -4.257300 | -3.356282 | 1.983634 | C | -3.698154 | -4.537482 | 2.459293 |
| C | -2.309076 | -4.572371 | 2.632248 | H | -1.859693 | -5.488644 | 3.015300 |
| C | -4.538346 | -5.773803 | 2.810004 | C | -6.034219 | -5.538859 | 2.566227 |
| H | -6.598341 | -6.446976 | 2.827612 | H | -6.425767 | -4.715412 | 3.183522 |
| H | -6.246563 | -5.308363 | 1.510735 | C | -4.080426 | -6.957938 | 1.938964 |
| H | -4.673356 | -7.855694 | 2.175216 | H | -3.020759 | -7.203713 | 2.106661 |
| H | -4.212173 | -6.733163 | 0.868953 | C | -4.333710 | -6.115253 | 4.297431 |
| H | -4.639716 | -5.274470 | 4.939719 | H | -4.938052 | -6.993977 | 4.573141 |
| H | -3.282503 | -6.349496 | 4.524044 | H | -5.335919 | -3.296525 | 1.857020 |
| C | -4.084876 | -0.955711 | 1.284511 | C | -5.474846 | -0.744009 | 1.408684 |
| H | -6.102783 | -1.583499 | 1.698816 | C | 4.436595 | -1.477263 | 1.914854 |
| C | 5.269271 | -0.487946 | 1.400332 | C | 4.675303 | 0.531222 | 0.645812 |
| H | 5.310995 | 1.307246 | 0.216322 | C | 6.787938 | -0.481255 | 1.624639 |
| C | 7.251723 | -1.684401 | 2.455323 | H | 8.342183 | -1.635959 | 2.595341 |
| H | 6.789103 | -1.696197 | 3.454306 | H | 7.022853 | -2.639764 | 1.958081 |
| C | 7.502156 | -0.523627 | 0.261588 | H | 8.594299 | -0.524229 | 0.404463 |
| H | 7.248790 | 0.346586 | -0.362690 | H | 7.230231 | -1.432538 | -0.297795 |
| C | 7.174062 | 0.807823 | 2.373132 | H | 8.260765 | 0.834947 | 2.550952 |
| H | 6.906504 | 1.708671 | 1.800183 | H | 6.666003 | 0.862042 | 3.348683 |
| H | 4.883485 | -2.286665 | 2.487029 | H | -2.573978 | 1.718684 | -1.241450 |
| H | -3.230543 | 2.276866 | 0.629622 | H | -5.614767 | 2.591984 | 0.852980 |
| C | -8.112209 | 1.267537 | 0.008555 | H | -9.196586 | 1.449242 | 0.075790 |
| H | -7.629389 | 2.216499 | -0.269893 | H | -7.936025 | 0.545580 | -0.804334 |
| C | -8.333424 | -0.565313 | 1.688130 | H | -9.411150 | -0.354725 | 1.761111 |
| H | -8.010650 | -0.985097 | 2.653562 | H | -8.196340 | -1.336877 | 0.914578 |
| C | -7.839533 | 1.752813 | 2.464750 | H | -7.455097 | 1.387536 | 3.430111 |
| H | -7.362171 | 2.721506 | 2.253736 | H | -8.921239 | 1.930246 | 2.573118 |
| Br | -2.389303 | 2.571062 | -4.659334 | Br | -3.030108 | 0.348900 | -6.407613 |

**TS22.out**

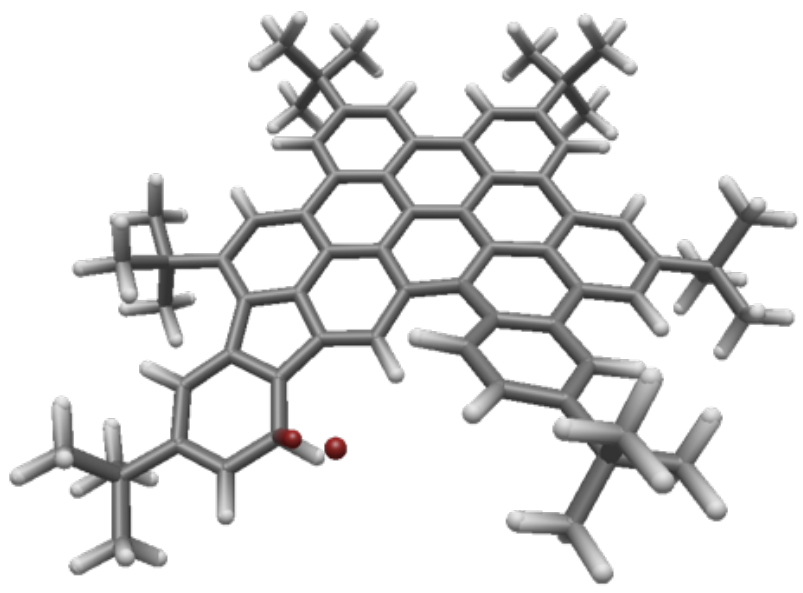

`Basis set: def2-TZVP`

Charge = 0, Multiplicity = 1

Electronic Energy = -7782.869571562170 Hartree

| | Cartesian Coordinates | | | | Cartesian Coordinates | | |
|---|---|---|---|---|---|---|---|
| Atoms | *X* | *Y* | *Z* | Atoms | *X* | *Y* | *Z* |
| C | -7.470572 | -0.635857 | -0.215019 | C | -5.942185 | -0.659866 | -0.104741 |
| C | -5.238569 | 0.560507 | -0.051536 | C | -3.863214 | 0.585263 | 0.007810 |
| C | -3.101083 | -0.605920 | 0.019525 | C | -1.641566 | -0.590461 | 0.053690 |
| C | -0.966218 | -1.697078 | 0.593869 | C | 0.423242 | -1.614151 | 0.914687 |
| C | 1.134013 | -0.427132 | 0.667850 | C | 0.493901 | 0.621205 | -0.029899 |
| C | -0.873884 | 0.524783 | -0.415517 | C | -1.406496 | 1.492823 | -1.326225 |
| C | -0.659433 | 2.596744 | -1.668856 | C | 0.653048 | 2.772440 | -1.130570 |
| C | 1.253375 | 1.787722 | -0.359430 | C | 2.583357 | 1.999416 | 0.052634 |
| C | 3.184574 | 3.212400 | -0.314430 | C | 2.570744 | 4.223793 | -1.087501 |
| C | 1.248073 | 3.980286 | -1.526257 | C | 0.205860 | 4.631679 | -2.384393 |
| C | -0.913690 | 3.752076 | -2.474326 | C | -2.089165 | 4.092525 | -3.248270 |
| C | -2.035251 | 5.345880 | -3.992468 | C | -0.980274 | 6.211423 | -3.893117 |
| C | 0.132178 | 5.830276 | -3.075636 | H | 0.962620 | 6.532749 | -3.014578 |
| C | -0.908551 | 7.555184 | -4.627180 | C | -2.152895 | 7.794395 | -5.489470 |
| H | -2.067089 | 8.766747 | -5.996921 | H | -2.267222 | 7.023309 | -6.267298 |
| H | -3.072066 | 7.815598 | -4.883885 | C | -0.801600 | 8.685049 | -3.586862 |
| H | -0.731085 | 9.658787 | -4.095854 | H | 0.089596 | 8.580591 | -2.947757 |
| H | -1.687349 | 8.702973 | -2.933463 | C | 0.333920 | 7.559895 | -5.536370 |
| H | 0.303164 | 6.724660 | -6.253331 | H | 0.378406 | 8.500876 | -6.106291 |
| H | 1.269285 | 7.481346 | -4.960025 | H | -2.895423 | 5.573195 | -4.623899 |
| H | -2.522910 | 3.251412 | -3.806796 | C | 3.352183 | 5.505269 | -1.420459 |
| C | 4.756537 | 5.526474 | -0.796807 | H | 5.250446 | 6.473423 | -1.061933 |
| H | 4.722354 | 5.467322 | 0.301519 | H | 5.390999 | 4.708719 | -1.170347 |
| C | 3.542933 | 5.598119 | -2.947864 | H | 4.210695 | 4.793615 | -3.292044 |
| H | 4.002879 | 6.561566 | -3.217857 | H | 2.608731 | 5.500358 | -3.514616 |
| C | 2.595468 | 6.719419 | -0.844790 | H | 1.537809 | 6.757190 | -1.133750 |
| H | 2.622439 | 6.690993 | 0.255281 | H | 3.070328 | 7.656939 | -1.173657 |
| H | 4.207276 | 3.386449 | 0.011954 | C | 3.238411 | 0.923899 | 0.813024 |
| C | 2.517116 | -0.278799 | 1.073533 | C | 3.164536 | -1.342409 | 1.742056 |
| C | 2.439759 | -2.605677 | 1.963430 | C | 1.095116 | -2.732139 | 1.534010 |
| C | 0.407769 | -3.960179 | 1.723146 | C | 1.069172 | -5.011980 | 2.353042 |
| C | 2.393365 | -4.907097 | 2.797058 | C | 3.055629 | -3.701076 | 2.587978 |
| H | 4.085473 | -3.606606 | 2.923158 | C | 3.054724 | -6.107085 | 3.489613 |

| | | | | | | | |
|---|---|---|---|---|---|---|---|
| C | 2.254131 | -6.457522 | 4.757643 | H | 2.716510 | -7.313670 | 5.273780 |
| H | 2.234097 | -5.606237 | 5.456030 | H | 1.213641 | -6.729747 | 4.524177 |
| C | 3.051840 | -7.308365 | 2.526193 | H | 3.524228 | -8.180721 | 3.004821 |
| H | 3.613805 | -7.076403 | 1.607826 | H | 2.030949 | -7.599026 | 2.235337 |
| C | 4.504243 | -5.812216 | 3.894894 | H | 4.569207 | -4.977492 | 4.609837 |
| H | 4.938494 | -6.699417 | 4.380011 | H | 5.132743 | -5.569264 | 3.024104 |
| H | 0.538688 | -5.949406 | 2.520260 | C | -0.988364 | -4.090892 | 1.261360 |
| C | -1.676268 | -2.946570 | 0.785062 | C | -3.040640 | -3.055315 | 0.423988 |
| C | -3.658358 | -4.314703 | 0.456814 | C | -2.981777 | -5.460424 | 0.864975 |
| C | -1.651772 | -5.317321 | 1.280165 | H | -1.115451 | -6.205590 | 1.614642 |
| C | -3.636258 | -6.849183 | 0.893722 | C | -5.080495 | -6.815518 | 0.378731 |
| H | -5.506153 | -7.830210 | 0.407908 | H | -5.722153 | -6.169354 | 0.997794 |
| H | -5.136367 | -6.460955 | -0.662222 | C | -2.823250 | -7.808105 | 0.004763 |
| H | -3.280174 | -8.810306 | 0.009971 | H | -1.785175 | -7.913751 | 0.354829 |
| H | -2.796032 | -7.450459 | -1.036506 | C | -3.646587 | -7.369230 | 2.343072 |
| H | -4.225950 | -6.698040 | 2.996327 | H | -4.108010 | -8.368566 | 2.385609 |
| H | -2.630320 | -7.452124 | 2.757452 | H | -4.699956 | -4.394592 | 0.152297 |
| C | -3.793357 | -1.831771 | 0.113605 | C | -5.201955 | -1.829247 | 0.007564 |
| H | -5.730190 | -2.780285 | 0.050953 | C | 4.493365 | -1.172443 | 2.164556 |
| C | 5.203511 | 0.004663 | 1.944723 | C | 4.552908 | 1.038501 | 1.260000 |
| H | 5.095828 | 1.964711 | 1.064927 | C | 6.652351 | 0.201781 | 2.412827 |
| C | 7.206360 | -1.042119 | 3.118031 | H | 8.244008 | -0.854306 | 3.432731 |
| H | 6.627217 | -1.296475 | 4.018999 | H | 7.214820 | -1.921174 | 2.455219 |
| C | 7.539315 | 0.505458 | 1.191805 | H | 8.585275 | 0.647126 | 1.506353 |
| H | 7.221227 | 1.420885 | 0.671121 | H | 7.508794 | -0.324406 | 0.468945 |
| C | 6.701370 | 1.384489 | 3.396944 | H | 7.734378 | 1.553012 | 3.739790 |
| H | 6.345173 | 2.317598 | 2.934794 | H | 6.076014 | 1.184158 | 4.280678 |
| H | 4.990145 | -1.989518 | 2.682471 | H | -2.391461 | 1.338882 | -1.767709 |
| H | -3.372381 | 1.555359 | 0.099112 | H | -5.770102 | 1.515661 | -0.033500 |
| C | -7.868504 | 0.189372 | -1.452133 | H | -8.964964 | 0.264349 | -1.523978 |
| H | -7.461475 | 1.211253 | -1.410800 | H | -7.495661 | -0.281257 | -2.375401 |
| C | -8.061964 | -2.043163 | -0.353977 | H | -9.154480 | -1.975909 | -0.469361 |
| H | -7.860672 | -2.664749 | 0.531610 | H | -7.662439 | -2.563894 | -1.238009 |
| C | -8.049086 | 0.023888 | 1.050277 | H | -7.761082 | -0.538906 | 1.951640 |
| H | -7.696341 | 1.060307 | 1.168101 | H | -9.149300 | 0.047923 | 0.998253 |
| Br | -3.624446 | 4.363032 | -1.846202 | Br | -5.848579 | 4.438063 | 0.011949 |

**TS31.out**

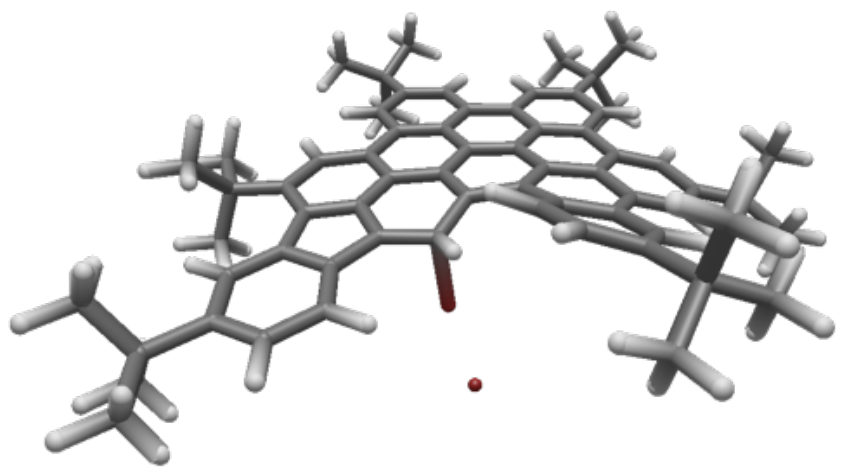

Basis set: def2-TZVP

Charge = 0, Multiplicity = 1

Electronic Energy = -7782.869009790223 Hartree

| | Cartesian Coordinates | | | | Cartesian Coordinates | | |
|---|---|---|---|---|---|---|---|
| Atoms | X | Y | Z | Atoms | X | Y | Z |
| C | -0.111928 | 0.003995 | -0.212775 | C | 1.418024 | 0.022491 | -0.109560 |
| C | 2.104681 | 1.251115 | -0.030759 | C | 3.483342 | 1.295687 | 0.015552 |
| C | 4.267225 | 0.120953 | -0.026726 | C | 5.737540 | 0.151910 | -0.028396 |
| C | 6.428069 | -0.971025 | 0.488285 | C | 7.827255 | -0.916845 | 0.731588 |
| C | 8.548825 | 0.242812 | 0.400493 | C | 7.883760 | 1.282680 | -0.288785 |
| C | 6.496184 | 1.228102 | -0.550589 | C | 5.924020 | 2.201339 | -1.527484 |
| C | 6.701267 | 3.366711 | -1.831493 | C | 8.060207 | 3.432003 | -1.449389 |
| C | 8.663192 | 2.415253 | -0.716106 | C | 10.030011 | 2.576362 | -0.410273 |
| C | 10.651050 | 3.746497 | -0.855834 | C | 10.035621 | 4.794769 | -1.592450 |
| C | 8.680108 | 4.619837 | -1.899200 | C | 7.584833 | 5.372861 | -2.620882 |
| C | 6.398030 | 4.583432 | -2.557160 | C | 5.207837 | 5.010805 | -3.124776 |
| C | 5.174833 | 6.249434 | -3.775818 | C | 6.316532 | 7.047054 | -3.859929 |
| C | 7.524020 | 6.588095 | -3.271258 | H | 8.403728 | 7.222609 | -3.347587 |
| C | 6.331027 | 8.403110 | -4.574386 | C | 4.948334 | 8.788645 | -5.113038 |
| H | 5.010308 | 9.767501 | -5.611793 | H | 4.578092 | 8.062591 | -5.852899 |
| H | 4.203300 | 8.872550 | -4.306738 | C | 6.790681 | 9.490709 | -3.584683 |
| H | 6.790638 | 10.473054 | -4.081989 | H | 7.809466 | 9.309483 | -3.210340 |
| H | 6.113921 | 9.546276 | -2.717698 | C | 7.315746 | 8.324684 | -5.756924 |
| H | 7.007149 | 7.548248 | -6.474057 | H | 7.343569 | 9.289128 | -6.287681 |
| H | 8.340414 | 8.095050 | -5.426984 | H | 4.235399 | 6.579949 | -4.218459 |
| H | 4.309464 | 4.390701 | -3.071936 | C | 10.867919 | 6.021658 | -2.000661 |
| C | 12.328317 | 5.930591 | -1.529742 | H | 12.862279 | 6.836408 | -1.853483 |
| H | 12.411316 | 5.873539 | -0.433729 | H | 12.851790 | 5.066375 | -1.966151 |
| C | 10.903205 | 6.115667 | -3.538977 | H | 11.464948 | 5.265157 | -3.954437 |
| H | 11.408319 | 7.042780 | -3.851468 | H | 9.909267 | 6.100263 | -4.002215 |
| C | 10.265118 | 7.278873 | -1.342886 | H | 9.191761 | 7.400326 | -1.533058 |
| H | 10.396585 | 7.230967 | -0.251029 | H | 10.778169 | 8.181838 | -1.708789 |
| H | 11.704776 | 3.870479 | -0.617075 | C | 10.706403 | 1.510278 | 0.343511 |
| C | 9.962806 | 0.354719 | 0.707251 | C | 10.619474 | -0.700935 | 1.383149 |
| C | 9.875012 | -1.931446 | 1.701131 | C | 8.511066 | -2.037181 | 1.340364 |
| C | 7.814710 | -3.245754 | 1.588510 | C | 8.476957 | -4.291079 | 2.231645 |
| C | 9.816514 | -4.198321 | 2.625085 | C | 10.494045 | -3.015628 | 2.340362 |
| H | 11.539906 | -2.932422 | 2.625243 | C | 10.482917 | -5.385429 | 3.335524 |

| | | | | | | | |
|---|---|---|---|---|---|---|---|
| C | 9.707089 | -5.699193 | 4.627981 | H | 10.174424 | -6.545761 | 5.155347 |
| H | 9.706720 | -4.831008 | 5.305683 | H | 8.660348 | -5.970035 | 4.422787 |
| C | 10.452066 | -6.610029 | 2.402399 | H | 10.926339 | -7.475018 | 2.892568 |
| H | 10.997583 | -6.405099 | 1.467874 | H | 9.423093 | -6.897665 | 2.138279 |
| C | 11.942443 | -5.090868 | 3.703800 | H | 12.027535 | -4.233181 | 4.389098 |
| H | 12.378689 | -5.965571 | 4.209433 | H | 12.556670 | -4.882530 | 2.814127 |
| H | 7.932155 | -5.210316 | 2.448383 | C | 6.412359 | -3.368966 | 1.154598 |
| C | 5.719799 | -2.221701 | 0.691621 | C | 4.354913 | -2.336712 | 0.336262 |
| C | 3.743295 | -3.596904 | 0.359365 | C | 4.424139 | -4.744037 | 0.760519 |
| C | 5.752755 | -4.598553 | 1.172401 | H | 6.297319 | -5.486145 | 1.495196 |
| C | 3.773239 | -6.134310 | 0.779138 | C | 2.325896 | -6.099210 | 0.273198 |
| H | 1.901724 | -7.114405 | 0.298724 | H | 1.687138 | -5.457450 | 0.899656 |
| H | 2.263369 | -5.738678 | -0.765239 | C | 4.583609 | -7.081749 | -0.124413 |
| H | 4.128520 | -8.084778 | -0.127659 | H | 5.623576 | -7.188808 | 0.219144 |
| H | 4.604804 | -6.712662 | -1.161771 | C | 3.773268 | -6.668153 | 2.223498 |
| H | 3.195611 | -6.004932 | 2.886427 | H | 3.315083 | -7.669221 | 2.259116 |
| H | 4.792250 | -6.751688 | 2.630844 | H | 2.702817 | -3.680975 | 0.053544 |
| C | 3.591782 | -1.112917 | 0.048458 | C | 2.183299 | -1.132179 | -0.035117 |
| H | 1.673959 | -2.093243 | -0.005961 | C | 11.974650 | -0.557446 | 1.710628 |
| C | 12.711625 | 0.581296 | 1.387397 | C | 12.053845 | 1.600845 | 0.693108 |
| H | 12.613221 | 2.491412 | 0.410273 | C | 14.210855 | 0.675416 | 1.709209 |
| C | 14.554622 | -0.096600 | 2.993493 | H | 15.617324 | 0.049841 | 3.239625 |
| H | 13.955626 | 0.257218 | 3.847477 | H | 14.395166 | -1.179822 | 2.888052 |
| C | 14.985514 | 0.062438 | 0.526794 | H | 16.070824 | 0.107291 | 0.711912 |
| H | 14.774948 | 0.606474 | -0.407207 | H | 14.707245 | -0.992150 | 0.375215 |
| C | 14.651295 | 2.135868 | 1.897389 | H | 15.711411 | 2.167101 | 2.191597 |
| H | 14.557235 | 2.725032 | 0.973308 | H | 14.065372 | 2.635304 | 2.685175 |
| H | 12.479197 | -1.368987 | 2.230321 | H | 4.834849 | 2.347887 | -1.533078 |
| H | 3.962417 | 2.266955 | 0.143788 | H | 1.549047 | 2.191362 | 0.010902 |
| C | -0.536675 | 0.813044 | -1.452014 | H | -1.633742 | 0.810263 | -1.550999 |
| H | -0.209882 | 1.861878 | -1.388857 | H | -0.109722 | 0.379354 | -2.370011 |
| C | -0.662785 | -1.420676 | -0.350836 | H | -1.758011 | -1.384387 | -0.452538 |
| H | -0.432890 | -2.039363 | 0.530360 | H | -0.260218 | -1.927515 | -1.241637 |
| C | -0.710446 | 0.642960 | 1.053778 | H | -0.416839 | 0.079520 | 1.953367 |
| H | -0.380043 | 1.684883 | 1.182412 | H | -1.810528 | 0.645525 | 0.995802 |
| Br | 6.141161 | 1.303419 | -3.410035 | Br | 6.170510 | -0.198979 | -5.889176 |

**TS32.out**

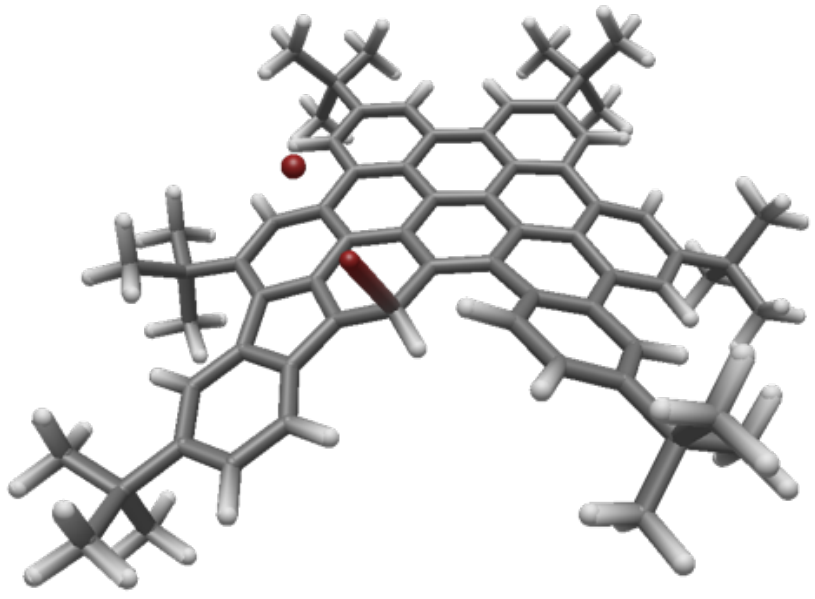

Basis set: def2-TZVP

Charge = 0, Multiplicity = 1

Electronic Energy = -7782.867684979782 Hartree

| Atoms | Cartesian Coordinates X | Y | Z | Atoms | Cartesian Coordinates X | Y | Z |
|---|---|---|---|---|---|---|---|
| C | -0.168432 | -0.121156 | -0.274017 | C | 1.349506 | -0.032767 | -0.077620 |
| C | 1.964054 | 1.211867 | 0.149278 | C | 3.333234 | 1.319565 | 0.291530 |
| C | 4.194605 | 0.202424 | 0.208576 | C | 5.669524 | 0.297549 | 0.273370 |
| C | 6.385675 | -0.866769 | 0.665831 | C | 7.785317 | -0.819982 | 0.914839 |
| C | 8.509259 | 0.350526 | 0.630975 | C | 7.827789 | 1.447969 | 0.060335 |
| C | 6.419587 | 1.451648 | -0.092387 | C | 5.790761 | 2.671526 | -0.678925 |
| C | 6.639512 | 3.606649 | -1.357673 | C | 8.038730 | 3.569435 | -1.149419 |
| C | 8.631205 | 2.549269 | -0.418348 | C | 10.027776 | 2.629748 | -0.249693 |
| C | 10.687234 | 3.712830 | -0.839161 | C | 10.084279 | 4.739044 | -1.612401 |
| C | 8.694608 | 4.648557 | -1.777472 | C | 7.591584 | 5.407157 | -2.483100 |
| C | 6.362181 | 4.741195 | -2.219004 | C | 5.159002 | 5.198450 | -2.738529 |
| C | 5.163063 | 6.345122 | -3.534716 | C | 6.350001 | 7.033942 | -3.807440 |
| C | 7.565008 | 6.541420 | -3.274638 | H | 8.480058 | 7.079083 | -3.499665 |
| C | 6.351649 | 8.272245 | -4.714561 | C | 5.075022 | 9.104259 | -4.508547 |
| H | 5.126087 | 10.018239 | -5.119619 | H | 4.167172 | 8.563536 | -4.812115 |
| H | 4.960706 | 9.405054 | -3.455334 | C | 7.562037 | 9.177644 | -4.434793 |
| H | 7.483486 | 10.092881 | -5.040364 | H | 8.517086 | 8.699694 | -4.699056 |
| H | 7.606685 | 9.475924 | -3.375345 | C | 6.415054 | 7.780622 | -6.174070 |
| H | 5.545264 | 7.152250 | -6.420518 | H | 6.424743 | 8.639513 | -6.863832 |
| H | 7.325688 | 7.187479 | -6.352073 | H | 4.215647 | 6.701677 | -3.940701 |
| H | 4.223481 | 4.676610 | -2.522584 | C | 10.960397 | 5.851500 | -2.211283 |
| C | 12.444769 | 5.702170 | -1.841863 | H | 13.006426 | 6.532011 | -2.296227 |
| H | 12.609306 | 5.747901 | -0.754560 | H | 12.876870 | 4.764541 | -2.223379 |
| C | 10.874080 | 5.783147 | -3.749118 | H | 11.347758 | 4.858164 | -4.112114 |
| H | 11.404590 | 6.636859 | -4.198748 | H | 9.845216 | 5.787593 | -4.129087 |
| C | 10.497755 | 7.211740 | -1.652165 | H | 9.422722 | 7.391207 | -1.773404 |
| H | 10.715438 | 7.269483 | -0.574702 | H | 11.035964 | 8.031372 | -2.153172 |
| H | 11.766042 | 3.763827 | -0.711224 | C | 10.699475 | 1.562427 | 0.502654 |
| C | 9.937856 | 0.424455 | 0.887587 | C | 10.590758 | -0.644394 | 1.540493 |
| C | 9.838478 | -1.871673 | 1.842571 | C | 8.478234 | -1.966771 | 1.465565 |
| C | 7.793794 | -3.191358 | 1.650269 | C | 8.445904 | -4.251930 | 2.278494 |
| C | 9.773871 | -4.161158 | 2.709096 | C | 10.450966 | -2.969381 | 2.464704 |
| H | 11.491920 | -2.889604 | 2.767759 | C | 10.430128 | -5.359482 | 3.409498 |

| | | | | | | | |
|---|---|---|---|---|---|---|---|
| C | 9.633325 | -5.693480 | 4.684081 | H | 10.092036 | -6.548333 | 5.205655 |
| H | 9.622039 | -4.836035 | 5.375130 | H | 8.589983 | -5.960401 | 4.457282 |
| C | 10.415517 | -6.569566 | 2.457316 | H | 10.885135 | -7.440609 | 2.941157 |
| H | 10.974032 | -6.349171 | 1.534004 | H | 9.391374 | -6.856438 | 2.174413 |
| C | 11.883365 | -5.069713 | 3.805439 | H | 11.956896 | -4.224231 | 4.506929 |
| H | 12.312972 | -5.952710 | 4.302262 | H | 12.510862 | -4.844700 | 2.929111 |
| H | 7.901337 | -5.180558 | 2.452203 | C | 6.415739 | -3.314912 | 1.153019 |
| C | 5.711855 | -2.152608 | 0.745989 | C | 4.367462 | -2.281789 | 0.331819 |
| C | 3.796852 | -3.558131 | 0.229519 | C | 4.492813 | -4.713585 | 0.572907 |
| C | 5.796663 | -4.561199 | 1.053950 | H | 6.357622 | -5.452809 | 1.335711 |
| C | 3.880568 | -6.116772 | 0.462732 | C | 2.457463 | -6.079849 | -0.108257 |
| H | 2.058473 | -7.103539 | -0.171288 | H | 1.774669 | -5.496446 | 0.528629 |
| H | 2.434009 | -5.649597 | -1.121492 | C | 4.756495 | -6.980584 | -0.462814 |
| H | 4.324872 | -7.989144 | -0.561555 | H | 5.779757 | -7.092750 | -0.073654 |
| H | 4.822077 | -6.536668 | -1.468601 | C | 3.827847 | -6.748346 | 1.866262 |
| H | 3.209698 | -6.140411 | 2.545412 | H | 3.388452 | -7.757163 | 1.814442 |
| H | 4.830043 | -6.841394 | 2.311640 | H | 2.774069 | -3.650528 | -0.126846 |
| C | 3.575330 | -1.063728 | 0.115920 | C | 2.177876 | -1.144606 | -0.060999 |
| H | 1.723786 | -2.128091 | -0.154745 | C | 11.955139 | -0.525354 | 1.849819 |
| C | 12.704201 | 0.598053 | 1.513462 | C | 12.052984 | 1.627537 | 0.823229 |
| H | 12.624791 | 2.509916 | 0.531637 | C | 14.197869 | 0.728383 | 1.842471 |
| C | 14.710260 | -0.457304 | 2.669849 | H | 15.777428 | -0.312843 | 2.896307 |
| H | 14.175061 | -0.548566 | 3.627799 | H | 14.614038 | -1.411203 | 2.128720 |
| C | 14.992006 | 0.787658 | 0.524412 | H | 16.070193 | 0.876712 | 0.731755 |
| H | 14.697577 | 1.650796 | -0.092145 | H | 14.832219 | -0.124825 | -0.071090 |
| C | 14.431232 | 2.019935 | 2.646943 | H | 15.498937 | 2.123670 | 2.896220 |
| H | 14.134211 | 2.916570 | 2.082432 | H | 13.859852 | 2.006495 | 3.588378 |
| H | 12.450109 | -1.344704 | 2.365264 | H | 4.789766 | 2.542828 | -1.113949 |
| H | 3.722785 | 2.301243 | 0.540272 | H | 1.362998 | 2.120759 | 0.232902 |
| C | -0.558481 | 0.715180 | -1.506647 | H | -1.647406 | 0.671425 | -1.667511 |
| H | -0.280378 | 1.773368 | -1.387860 | H | -0.064045 | 0.332709 | -2.413527 |
| C | -0.638668 | -1.564093 | -0.494114 | H | -1.729203 | -1.578465 | -0.641775 |
| H | -0.413098 | -2.206205 | 0.371451 | H | -0.175893 | -2.014699 | -1.386013 |
| C | -0.872531 | 0.438456 | 0.975707 | H | -0.598959 | -0.140105 | 1.872022 |
| H | -0.611190 | 1.491570 | 1.158424 | H | -1.965840 | 0.382128 | 0.853535 |
| Br | 5.473162 | 4.061338 | 0.858919 | Br | 4.698637 | 5.642448 | 3.162047 |

**Int11.out**

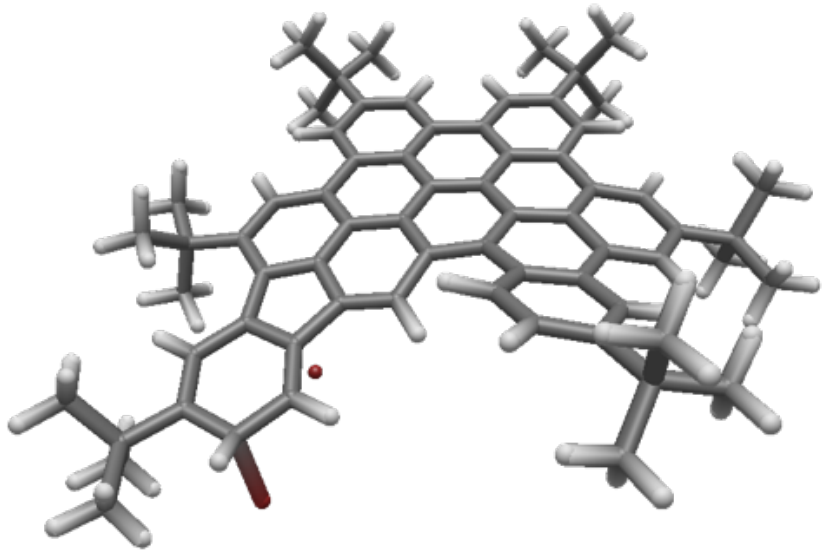

Basis set: def2-TZVP

Charge = 0, Multiplicity = 1

Electronic Energy = -7782.878997720129 Hartree

| | Cartesian Coordinates | | | | Cartesian Coordinates | | |
|---|---|---|---|---|---|---|---|
| Atoms | X | Y | Z | Atoms | X | Y | Z |
| C | -0.124284 | 0.018316 | 0.026874 | C | 1.402761 | 0.027501 | 0.046250 |
| C | 2.092119 | 1.251700 | 0.064305 | C | 3.464587 | 1.292586 | 0.053260 |
| C | 4.238965 | 0.116113 | 0.011631 | C | 5.700042 | 0.144728 | -0.011889 |
| C | 6.392466 | -0.968751 | 0.479614 | C | 7.790292 | -0.905081 | 0.724872 |
| C | 8.492372 | 0.266550 | 0.438713 | C | 7.823976 | 1.323658 | -0.206975 |
| C | 6.444755 | 1.259632 | -0.496986 | C | 5.873951 | 2.269573 | -1.350757 |
| C | 6.618327 | 3.334499 | -1.740692 | C | 7.965116 | 3.452492 | -1.339687 |
| C | 8.585746 | 2.460371 | -0.604711 | C | 9.946769 | 2.623522 | -0.324683 |
| C | 10.588240 | 3.797115 | -0.784394 | C | 9.986293 | 4.795990 | -1.529763 |
| C | 8.600342 | 4.614914 | -1.848424 | C | 7.614362 | 5.276335 | -2.637143 |
| C | 6.376620 | 4.500528 | -2.589617 | C | 5.291297 | 4.882042 | -3.265586 |
| C | 5.316356 | 6.091929 | -4.118595 | C | 6.550481 | 6.929300 | -4.067492 |
| C | 7.630807 | 6.490338 | -3.380133 | H | 8.517058 | 7.098231 | -3.381163 |
| C | 6.594834 | 8.283666 | -4.771455 | C | 5.204853 | 8.787492 | -5.179682 |
| H | 5.316333 | 9.761977 | -5.659347 | H | 4.710476 | 8.123937 | -5.889400 |
| H | 4.558525 | 8.918532 | -4.307467 | C | 7.205840 | 9.330896 | -3.821434 |
| H | 7.166676 | 10.309519 | -4.305604 | H | 8.249970 | 9.119870 | -3.586058 |
| H | 6.642219 | 9.385000 | -2.886700 | C | 7.489718 | 8.162493 | -6.019025 |
| H | 7.076219 | 7.453726 | -6.737888 | H | 7.565259 | 9.141060 | -6.500074 |
| H | 8.494754 | 7.832927 | -5.745850 | H | 4.421534 | 6.694532 | -3.967805 |
| H | 4.374625 | 4.302979 | -3.254360 | C | 10.803143 | 6.006122 | -1.973197 |
| C | 12.249752 | 5.972368 | -1.460171 | H | 12.756811 | 6.875641 | -1.808257 |
| H | 12.296207 | 5.961886 | -0.368524 | H | 12.800530 | 5.112232 | -1.847388 |
| C | 10.893435 | 6.019458 | -3.512834 | H | 11.570923 | 5.227118 | -3.839959 |
| H | 11.297777 | 6.977432 | -3.850339 | H | 9.945619 | 5.843764 | -4.015050 |
| C | 10.170914 | 7.282581 | -1.381725 | H | 9.093416 | 7.344883 | -1.514012 |
| H | 10.362647 | 7.314658 | -0.306063 | H | 10.626820 | 8.166102 | -1.835913 |
| H | 11.632977 | 3.912835 | -0.550021 | C | 10.622396 | 1.562142 | 0.408238 |
| C | 9.895209 | 0.389225 | 0.744439 | C | 10.563280 | -0.668516 | 1.385398 |
| C | 9.839069 | -1.913413 | 1.667239 | C | 8.483479 | -2.027955 | 1.303015 |
| C | 7.802967 | -3.245069 | 1.520213 | C | 8.471616 | -4.290928 | 2.137785 |
| C | 9.805858 | -4.189628 | 2.530336 | C | 10.470272 | -3.001852 | 2.277116 |
| H | 11.505852 | -2.916858 | 2.564608 | C | 10.477312 | -5.380525 | 3.214686 |

| | | | | | | | |
|---|---|---|---|---|---|---|---|
| C | 9.712575 | -5.716087 | 4.505124 | H | 10.183464 | -6.567042 | 5.005362 |
| H | 9.721925 | -4.864768 | 5.191427 | H | 8.672478 | -5.978133 | 4.299264 |
| C | 10.441410 | -6.592146 | 2.269402 | H | 10.921178 | -7.450828 | 2.747544 |
| H | 10.975048 | -6.372335 | 1.340622 | H | 9.417049 | -6.874426 | 2.016640 |
| C | 11.936731 | -5.094934 | 3.578475 | H | 12.022350 | -4.252028 | 4.269407 |
| H | 12.362867 | -5.974621 | 4.067052 | H | 12.538270 | -4.879006 | 2.691477 |
| H | 7.943773 | -5.212651 | 2.337873 | C | 6.400392 | -3.370719 | 1.095032 |
| C | 5.696811 | -2.222871 | 0.673730 | C | 4.332688 | -2.334005 | 0.352212 |
| C | 3.728344 | -3.593997 | 0.350003 | C | 4.424458 | -4.739800 | 0.696164 |
| C | 5.755500 | -4.599679 | 1.086199 | H | 6.305815 | -5.487009 | 1.365963 |
| C | 3.791338 | -6.131095 | 0.675210 | C | 2.326939 | -6.095158 | 0.230327 |
| H | 1.926826 | -7.112107 | 0.230671 | H | 1.714677 | -5.493059 | 0.907052 |
| H | 2.222716 | -5.693594 | -0.781145 | C | 4.572882 | -7.025134 | -0.300763 |
| H | 4.130178 | -8.024946 | -0.325416 | H | 5.619147 | -7.125575 | -0.003715 |
| H | 4.542778 | -6.610076 | -1.311982 | C | 3.853818 | -6.737796 | 2.086198 |
| H | 3.307215 | -6.113573 | 2.798570 | H | 3.401375 | -7.733516 | 2.083004 |
| H | 4.883338 | -6.835746 | 2.437520 | H | 2.692090 | -3.677104 | 0.061384 |
| C | 3.565786 | -1.109332 | 0.113810 | C | 2.159136 | -1.124804 | 0.098324 |
| H | 1.652042 | -2.074684 | 0.167261 | C | 11.912554 | -0.514767 | 1.717913 |
| C | 12.628207 | 0.637395 | 1.428027 | C | 11.963636 | 1.661539 | 0.761696 |
| H | 12.510515 | 2.560150 | 0.512673 | C | 14.100191 | 0.813390 | 1.801788 |
| C | 14.668955 | -0.416757 | 2.513507 | H | 15.718339 | -0.235266 | 2.757978 |
| H | 14.138043 | -0.625020 | 3.446360 | H | 14.620876 | -1.306873 | 1.880499 |
| C | 14.920767 | 1.059336 | 0.525640 | H | 15.975988 | 1.186976 | 0.782603 |
| H | 14.589738 | 1.958683 | 0.001740 | H | 14.832839 | 0.211775 | -0.159577 |
| C | 14.236340 | 2.024384 | 2.738629 | H | 15.285254 | 2.165487 | 3.013880 |
| H | 13.885532 | 2.942079 | 2.261069 | H | 13.657155 | 1.871217 | 3.653373 |
| H | 12.422655 | -1.322603 | 2.218192 | H | 4.866087 | 2.147752 | -1.721393 |
| H | 3.951574 | 2.254964 | 0.129251 | H | 1.542745 | 2.185159 | 0.107841 |
| C | -0.611518 | 0.776146 | -1.218621 | H | -1.704820 | 0.783021 | -1.245824 |
| H | -0.266308 | 1.812284 | -1.217572 | H | -0.247376 | 0.294644 | -2.130459 |
| C | -0.697055 | -1.400598 | -0.015466 | H | -1.787758 | -1.346355 | -0.057367 |
| H | -0.424511 | -1.973428 | 0.874894 | H | -0.351838 | -1.946395 | -0.897600 |
| C | -0.651161 | 0.719570 | 1.289174 | H | -0.312395 | 0.198743 | 2.189031 |
| H | -0.310358 | 1.755795 | 1.344355 | H | -1.744871 | 0.722503 | 1.283824 |
| Br | 5.110658 | 5.385133 | -5.947206 | Br | 8.619972 | 2.865036 | -5.102824 |

**Int12.out**

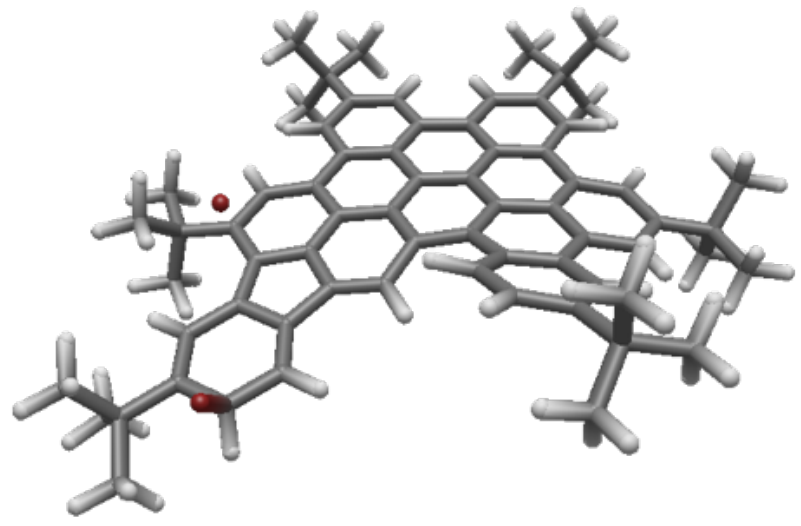

Basis set: def2-TZVP

Charge = 0, Multiplicity = 1

Electronic Energy = -7782.879202543359 Hartree

| Atoms | Cartesian Coordinates X | Y | Z | Atoms | Cartesian Coordinates X | Y | Z |
|---|---|---|---|---|---|---|---|
| C | -0.111390 | 0.049198 | 0.109942 | C | 1.415749 | 0.044847 | 0.095201 |
| C | 2.115562 | 1.263256 | 0.091803 | C | 3.487924 | 1.292957 | 0.054868 |
| C | 4.250623 | 0.109166 | 0.005226 | C | 5.711237 | 0.125772 | -0.037688 |
| C | 6.401028 | -0.989220 | 0.454276 | C | 7.799866 | -0.930787 | 0.693110 |
| C | 8.504561 | 0.238029 | 0.402485 | C | 7.839020 | 1.292152 | -0.250380 |
| C | 6.458994 | 1.232205 | -0.536467 | C | 5.887001 | 2.244406 | -1.386755 |
| C | 6.635241 | 3.303903 | -1.784040 | C | 7.983439 | 3.418051 | -1.386589 |
| C | 8.602024 | 2.427776 | -0.647734 | C | 9.955968 | 2.602884 | -0.342144 |
| C | 10.590903 | 3.788152 | -0.779251 | C | 9.987564 | 4.791596 | -1.518707 |
| C | 8.609101 | 4.599310 | -1.861388 | C | 7.603605 | 5.296239 | -2.595126 |
| C | 6.378738 | 4.499289 | -2.586625 | C | 5.278219 | 4.910247 | -3.219244 |
| C | 5.247024 | 6.216133 | -3.916467 | C | 6.529194 | 6.977080 | -3.985169 |
| C | 7.616314 | 6.521117 | -3.320050 | H | 8.521225 | 7.098738 | -3.366109 |
| C | 6.625329 | 8.254203 | -4.816677 | C | 5.465875 | 8.401879 | -5.810047 |
| H | 5.609135 | 9.324758 | -6.375603 | H | 5.447920 | 7.573373 | -6.523429 |
| H | 4.496160 | 8.463991 | -5.315718 | C | 6.650896 | 9.464034 | -3.863497 |
| H | 6.778378 | 10.376891 | -4.451103 | H | 7.483499 | 9.385755 | -3.160556 |
| H | 5.723752 | 9.544748 | -3.294973 | C | 7.933387 | 8.240300 | -5.629030 |
| H | 7.998507 | 7.343776 | -6.250568 | H | 7.949679 | 9.115821 | -6.282219 |
| H | 8.817726 | 8.287404 | -4.991960 | H | 4.789705 | 6.124252 | -4.901016 |
| H | 4.360659 | 4.332663 | -3.208883 | C | 10.798841 | 6.014822 | -1.936107 |
| C | 12.240850 | 5.985349 | -1.410008 | H | 12.743265 | 6.897972 | -1.739905 |
| H | 12.275507 | 5.960805 | -0.318390 | H | 12.803500 | 5.135050 | -1.802553 |
| C | 10.905534 | 6.042162 | -3.474559 | H | 11.572707 | 5.241281 | -3.803361 |
| H | 11.330612 | 6.995647 | -3.798657 | H | 9.957139 | 5.896843 | -3.986054 |
| C | 10.151606 | 7.281578 | -1.340641 | H | 9.072506 | 7.328536 | -1.462209 |
| H | 10.345807 | 7.313803 | -0.265759 | H | 10.595113 | 8.171274 | -1.795336 |
| H | 11.629675 | 3.914592 | -0.524635 | C | 10.630112 | 1.540756 | 0.391997 |
| C | 9.905407 | 0.362695 | 0.715652 | C | 10.572124 | -0.696240 | 1.356115 |
| C | 9.848795 | -1.943953 | 1.628935 | C | 8.491636 | -2.055341 | 1.269265 |
| C | 7.808732 | -3.271280 | 1.486090 | C | 8.479511 | -4.322041 | 2.093122 |
| C | 9.816749 | -4.225825 | 2.476858 | C | 10.481597 | -3.037219 | 2.228413 |
| H | 11.518790 | -2.955724 | 2.511127 | C | 10.490310 | -5.422370 | 3.149288 |

| | | | | | | | |
|---|---|---|---|---|---|---|---|
| C | 9.734139 | -5.763541 | 4.443289 | H | 10.206501 | -6.618558 | 4.935155 |
| H | 9.750626 | -4.916211 | 5.134328 | H | 8.691921 | -6.021732 | 4.243405 |
| C | 10.444584 | -6.628402 | 2.197262 | H | 10.925938 | -7.490900 | 2.666851 |
| H | 10.971904 | -6.404330 | 1.265856 | H | 9.417706 | -6.906828 | 1.950480 |
| C | 11.953050 | -5.142428 | 3.504089 | H | 12.045600 | -4.304400 | 4.200000 |
| H | 12.380790 | -6.026315 | 3.983588 | H | 12.548431 | -4.921764 | 2.614077 |
| H | 7.951662 | -5.244081 | 2.291622 | C | 6.401158 | -3.389699 | 1.074475 |
| C | 5.699727 | -2.238287 | 0.659118 | C | 4.330903 | -2.340396 | 0.354826 |
| C | 3.718000 | -3.596179 | 0.362991 | C | 4.410940 | -4.745943 | 0.702959 |
| C | 5.747735 | -4.614268 | 1.076108 | H | 6.294426 | -5.504886 | 1.352466 |
| C | 3.767897 | -6.132887 | 0.694245 | C | 2.297684 | -6.087912 | 0.269746 |
| H | 1.890484 | -7.102012 | 0.278819 | H | 1.699379 | -5.479444 | 0.953152 |
| H | 2.182118 | -5.688588 | -0.741398 | C | 4.529542 | -7.034704 | -0.290314 |
| H | 4.079667 | -8.031489 | -0.306294 | H | 5.579141 | -7.141549 | -0.007535 |
| H | 4.488118 | -6.621839 | -1.302066 | C | 3.845885 | -6.736863 | 2.105653 |
| H | 3.313434 | -6.107349 | 2.824012 | H | 3.386726 | -7.729490 | 2.110849 |
| H | 4.879523 | -6.840892 | 2.442825 | H | 2.677463 | -3.672641 | 0.088036 |
| C | 3.569594 | -1.110364 | 0.124661 | C | 2.162910 | -1.114114 | 0.137262 |
| H | 1.649382 | -2.059250 | 0.222645 | C | 11.917797 | -0.538601 | 1.701308 |
| C | 12.630446 | 0.618829 | 1.424982 | C | 11.967302 | 1.644287 | 0.758964 |
| H | 12.511829 | 2.547706 | 0.522286 | C | 14.097703 | 0.800103 | 1.814682 |
| C | 14.665463 | -0.430946 | 2.525732 | H | 15.711353 | -0.245299 | 2.781790 |
| H | 14.126186 | -0.646566 | 3.452041 | H | 14.628328 | -1.318070 | 1.887733 |
| C | 14.930032 | 1.056688 | 0.548216 | H | 15.981936 | 1.188174 | 0.816563 |
| H | 14.599835 | 1.957260 | 0.025845 | H | 14.853321 | 0.212105 | -0.142128 |
| C | 14.218429 | 2.006861 | 2.759091 | H | 15.263850 | 2.151284 | 3.045663 |
| H | 13.867951 | 2.925416 | 2.283022 | H | 13.630724 | 1.846068 | 3.666990 |
| H | 12.426853 | -1.346518 | 2.202550 | H | 4.870613 | 2.135860 | -1.737568 |
| H | 3.983810 | 2.251824 | 0.119325 | H | 1.575720 | 2.201918 | 0.141692 |
| C | -0.619397 | 0.795900 | -1.134005 | H | -1.712909 | 0.814118 | -1.136133 |
| H | -0.263225 | 1.828134 | -1.154148 | H | -0.281312 | 0.298627 | -2.047475 |
| C | -0.697789 | -1.364719 | 0.099099 | H | -1.788656 | -1.300492 | 0.083779 |
| H | -0.408209 | -1.929114 | 0.989463 | H | -0.380070 | -1.924689 | -0.784565 |
| C | -0.603646 | 0.771311 | 1.374520 | H | -0.248215 | 0.259743 | 2.273242 |
| H | -0.253774 | 1.805291 | 1.407991 | H | -1.697137 | 0.782742 | 1.394057 |
| Br | 3.937274 | 7.264671 | -2.881718 | Br | 6.497518 | 6.414263 | 0.581800 |

**Int21.out**

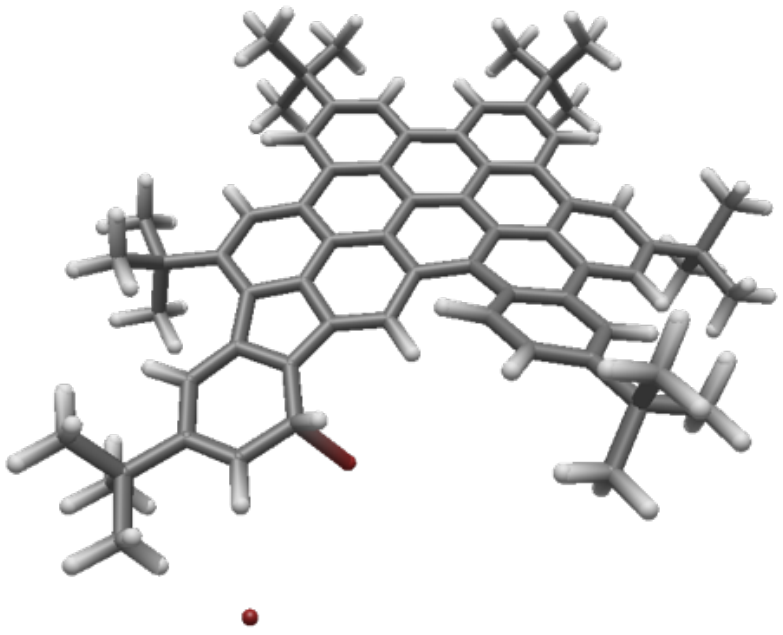

Basis set: def2-TZVP

Charge = 0, Multiplicity = 1

Electronic Energy = -7782.868046125962 Hartree

| | Cartesian Coordinates | | | | Cartesian Coordinates | | |
|---|---|---|---|---|---|---|---|
| Atoms | *X* | *Y* | *Z* | Atoms | *X* | *Y* | *Z* |
| C | -0.049963 | 0.083483 | -0.069658 | C | 1.475981 | 0.075264 | -0.019232 |
| C | 2.177500 | 1.293514 | 0.009388 | C | 3.549103 | 1.319935 | 0.022182 |
| C | 4.309707 | 0.132520 | -0.004336 | C | 5.765095 | 0.139985 | -0.023032 |
| C | 6.448025 | -0.974147 | 0.465494 | C | 7.849489 | -0.913844 | 0.718525 |
| C | 8.569182 | 0.253057 | 0.435191 | C | 7.919598 | 1.310506 | -0.220966 |
| C | 6.525714 | 1.251523 | -0.515395 | C | 5.966903 | 2.218757 | -1.381268 |
| C | 6.726360 | 3.298373 | -1.798236 | C | 8.087549 | 3.415033 | -1.384911 |
| C | 8.695379 | 2.433666 | -0.628168 | C | 10.054678 | 2.594973 | -0.328580 |
| C | 10.686639 | 3.749698 | -0.799752 | C | 10.068397 | 4.745600 | -1.572814 |
| C | 8.709230 | 4.556545 | -1.886996 | C | 7.635690 | 5.231116 | -2.671062 |
| C | 6.462676 | 4.419742 | -2.602089 | C | 5.222042 | 4.802223 | -3.297396 |
| C | 5.261748 | 6.102330 | -4.008257 | C | 6.363254 | 6.873726 | -4.038668 |
| C | 7.560101 | 6.397721 | -3.383098 | H | 8.433882 | 7.024639 | -3.473295 |
| C | 6.441534 | 8.227980 | -4.735516 | C | 5.083379 | 8.652818 | -5.295041 |
| H | 5.181639 | 9.639978 | -5.752812 | H | 4.730152 | 7.959873 | -6.062261 |
| H | 4.331559 | 8.720977 | -4.503624 | C | 6.910227 | 9.286302 | -3.723033 |
| H | 6.951061 | 10.261988 | -4.213290 | H | 7.907169 | 9.073400 | -3.328976 |
| H | 6.214177 | 9.352519 | -2.882376 | C | 7.449658 | 8.129047 | -5.891581 |
| H | 7.110398 | 7.404767 | -6.636366 | H | 7.546003 | 9.103536 | -6.377039 |
| H | 8.442164 | 7.829717 | -5.543451 | H | 4.346231 | 6.392404 | -4.506145 |
| H | 4.369733 | 4.766629 | -2.614404 | C | 10.876970 | 5.955608 | -2.041861 |
| C | 12.332221 | 5.930102 | -1.555842 | H | 12.835279 | 6.827538 | -1.924315 |
| H | 12.397816 | 5.933697 | -0.465136 | H | 12.874589 | 5.062601 | -1.939234 |
| C | 10.930125 | 5.963257 | -3.580704 | H | 11.544486 | 5.128869 | -3.928870 |
| H | 11.384956 | 6.892898 | -3.932619 | H | 9.955222 | 5.858417 | -4.052176 |
| C | 10.246344 | 7.237030 | -1.465861 | H | 9.173844 | 7.308692 | -1.634911 |
| H | 10.402559 | 7.266936 | -0.384310 | H | 10.723824 | 8.117019 | -1.904732 |
| H | 11.731301 | 3.880371 | -0.567624 | C | 10.712324 | 1.530258 | 0.431269 |
| C | 9.972535 | 0.365987 | 0.760294 | C | 10.619540 | -0.693058 | 1.418602 |
| C | 9.879462 | -1.930708 | 1.690122 | C | 8.526269 | -2.037816 | 1.308481 |
| C | 7.833443 | -3.250599 | 1.524435 | C | 8.487559 | -4.298338 | 2.151802 |
| C | 9.818745 | -4.204610 | 2.557468 | C | 10.494554 | -3.022177 | 2.309655 |
| H | 11.526866 | -2.944522 | 2.609445 | C | 10.474622 | -5.398271 | 3.250859 |

| | | | | | | | |
|---|---|---|---|---|---|---|---|
| C | 9.692302 | -5.727039 | 4.532691 | H | 10.153848 | -6.579216 | 5.039228 |
| H | 9.698115 | -4.874596 | 5.217552 | H | 8.653566 | -5.984930 | 4.315317 |
| C | 10.440999 | -6.610156 | 2.305553 | H | 10.909688 | -7.471016 | 2.790448 |
| H | 10.986975 | -6.394728 | 1.382966 | H | 9.417761 | -6.885970 | 2.041490 |
| C | 11.931630 | -5.121745 | 3.631062 | H | 12.014682 | -4.278586 | 4.321961 |
| H | 12.345597 | -6.003743 | 4.125665 | H | 12.544771 | -4.911244 | 2.750768 |
| H | 7.951772 | -5.215536 | 2.350018 | C | 6.432535 | -3.369747 | 1.093292 |
| C | 5.740490 | -2.218547 | 0.664916 | C | 4.375653 | -2.316240 | 0.343075 |
| C | 3.757991 | -3.570798 | 0.350472 | C | 4.442808 | -4.720716 | 0.702260 |
| C | 5.776655 | -4.591882 | 1.090342 | H | 6.317013 | -5.483517 | 1.375455 |
| C | 3.795671 | -6.105414 | 0.692105 | C | 2.332195 | -6.057798 | 0.245233 |
| H | 1.922088 | -7.070643 | 0.252744 | H | 1.725357 | -5.444726 | 0.916923 |
| H | 2.233365 | -5.662817 | -0.769378 | C | 4.569273 | -7.015269 | -0.275665 |
| H | 4.115599 | -8.010208 | -0.292570 | H | 5.613960 | -7.124941 | 0.023708 |
| H | 4.544652 | -6.608213 | -1.290261 | C | 3.850904 | -6.700425 | 2.108469 |
| H | 3.309689 | -6.064800 | 2.814786 | H | 3.388534 | -7.691469 | 2.112697 |
| H | 4.879128 | -6.805716 | 2.461534 | H | 2.720297 | -3.645593 | 0.065209 |
| C | 3.622812 | -1.087096 | 0.093874 | C | 2.216772 | -1.086069 | 0.052019 |
| H | 1.696214 | -2.029095 | 0.112116 | C | 11.963595 | -0.550571 | 1.779321 |
| C | 12.689289 | 0.595392 | 1.497409 | C | 12.043434 | 1.621546 | 0.811069 |
| H | 12.599230 | 2.516805 | 0.566826 | C | 14.154284 | 0.765954 | 1.899655 |
| C | 14.702119 | -0.461425 | 2.632511 | H | 15.747259 | -0.283221 | 2.896915 |
| H | 14.151167 | -0.659573 | 3.555847 | H | 14.662234 | -1.356213 | 2.005546 |
| C | 15.002591 | 0.996763 | 0.638826 | H | 16.053061 | 1.119996 | 0.916651 |
| H | 14.688086 | 1.893687 | 0.100797 | H | 14.923793 | 0.143915 | -0.041035 |
| C | 14.278934 | 1.983643 | 2.829566 | H | 15.322968 | 2.120457 | 3.124855 |
| H | 13.943151 | 2.899453 | 2.337940 | H | 13.680656 | 1.840796 | 3.733631 |
| H | 12.457906 | -1.358451 | 2.294439 | H | 4.960116 | 2.091784 | -1.752784 |
| H | 4.045687 | 2.277054 | 0.109024 | H | 1.636922 | 2.232248 | 0.042824 |
| C | -0.502099 | 0.849826 | -1.323363 | H | -1.594338 | 0.867425 | -1.373138 |
| H | -0.147013 | 1.882452 | -1.312376 | H | -0.123902 | 0.366772 | -2.228572 |
| C | -0.637982 | -1.328660 | -0.127486 | H | -1.726546 | -1.260619 | -0.194468 |
| H | -0.393321 | -1.906074 | 0.767995 | H | -0.279300 | -1.876768 | -1.002781 |
| C | -0.593371 | 0.788183 | 1.183809 | H | -0.278919 | 0.261298 | 2.088928 |
| H | -0.242182 | 1.820380 | 1.248159 | H | -1.686511 | 0.803323 | 1.155784 |
| Br | 4.825723 | 3.408859 | -4.621225 | Br | 4.080252 | 5.924757 | -8.080508 |

## Int22_def2-TZVP.out

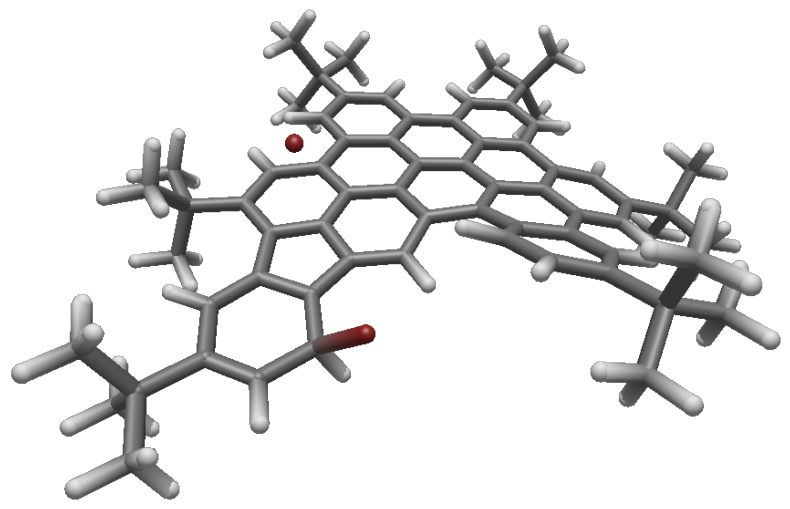

Basis set: def2-TZVP

Charge = 0, Multiplicity = 1

Electronic Energy = -7782.871736952497 Hartree

| Atoms | Cartesian Coordinates | | | Atoms | Cartesian Coordinates | | |
|---|---|---|---|---|---|---|---|
| | X | Y | Z | | X | Y | Z |
| C | -7.442577 | -0.657903 | -0.177622 | C | -5.918200 | -0.660942 | -0.075117 |
| C | -5.221895 | 0.561681 | -0.039701 | C | -3.851482 | 0.594116 | 0.024023 |
| C | -3.087587 | -0.592428 | 0.041126 | C | -1.632117 | -0.577011 | 0.077533 |
| C | -0.960306 | -1.679090 | 0.610518 | C | 0.429520 | -1.599347 | 0.927088 |
| C | 1.146031 | -0.421253 | 0.672278 | C | 0.514664 | 0.623249 | -0.025266 |
| C | -0.861781 | 0.538147 | -0.395122 | C | -1.391826 | 1.487547 | -1.298135 |
| C | -0.635628 | 2.587947 | -1.675623 | C | 0.696865 | 2.738459 | -1.171221 |
| C | 1.286580 | 1.766493 | -0.386418 | C | 2.618562 | 1.966255 | 0.008808 |
| C | 3.236605 | 3.156898 | -0.388798 | C | 2.630025 | 4.153763 | -1.173924 |
| C | 1.304644 | 3.921506 | -1.592109 | C | 0.255167 | 4.589207 | -2.421625 |
| C | -0.895603 | 3.725937 | -2.456725 | C | -2.093055 | 4.086728 | -3.243241 |
| C | -2.051025 | 5.419029 | -3.897533 | C | -0.982872 | 6.230273 | -3.820563 |
| C | 0.174713 | 5.790567 | -3.065728 | H | 1.008317 | 6.474727 | -3.021882 |
| C | -0.907314 | 7.604668 | -4.478734 | C | -2.187693 | 7.933226 | -5.248609 |
| H | -2.088177 | 8.922226 | -5.701509 | H | -2.369696 | 7.211490 | -6.049304 |
| H | -3.058581 | 7.950418 | -4.588205 | C | -0.703871 | 8.665789 | -3.384352 |
| H | -0.647783 | 9.655935 | -3.843553 | H | 0.219683 | 8.506076 | -2.822671 |
| H | -1.539211 | 8.656565 | -2.679545 | C | 0.277708 | 7.630456 | -5.457807 |
| H | 0.146563 | 6.880053 | -6.241960 | H | 0.340201 | 8.614834 | -5.928305 |
| H | 1.229875 | 7.440694 | -4.956419 | H | -2.937049 | 5.690424 | -4.455421 |
| H | -2.254817 | 3.316884 | -4.005967 | C | 3.412340 | 5.418923 | -1.532388 |
| C | 4.837338 | 5.427123 | -0.962387 | H | 5.321913 | 6.361138 | -1.257747 |
| H | 4.838983 | 5.380043 | 0.129064 | H | 5.436919 | 4.601055 | -1.352565 |
| C | 3.555423 | 5.513617 | -3.062971 | H | 4.224501 | 4.724626 | -3.416486 |
| H | 3.990402 | 6.478615 | -3.336409 | H | 2.614622 | 5.397714 | -3.596764 |
| C | 2.691698 | 6.639403 | -0.927707 | H | 1.623296 | 6.660474 | -1.133092 |
| H | 2.803398 | 6.626816 | 0.159421 | H | 3.138536 | 7.562213 | -1.307525 |
| H | 4.254955 | 3.323656 | -0.075891 | C | 3.261813 | 0.902169 | 0.787527 |
| C | 2.530426 | -0.282565 | 1.069575 | C | 3.165789 | -1.336698 | 1.750752 |
| C | 2.432526 | -2.588755 | 1.986102 | C | 1.094629 | -2.713951 | 1.551495 |
| C | 0.405946 | -3.935759 | 1.745307 | C | 1.051347 | -4.976676 | 2.395580 |
| C | 2.367859 | -4.867258 | 2.847421 | C | 3.037785 | -3.674691 | 2.627717 |
| H | 4.056959 | -3.584305 | 2.967002 | C | 3.014370 | -6.054289 | 3.563372 |

| | | | | | | | |
|---|---|---|---|---|---|---|---|
| C | 2.200147 | -6.384978 | 4.825182 | H | 2.654743 | -7.231744 | 5.346867 |
| H | 2.180902 | -5.529932 | 5.506444 | H | 1.169763 | -6.651505 | 4.580350 |
| C | 3.017444 | -7.270834 | 2.623048 | H | 3.479019 | -8.125249 | 3.125864 |
| H | 3.588113 | -7.055199 | 1.715541 | H | 2.004682 | -7.556627 | 2.331025 |
| C | 4.458443 | -5.764577 | 3.982458 | H | 4.515313 | -4.921696 | 4.676261 |
| H | 4.866867 | -6.643553 | 4.486983 | H | 5.092189 | -5.547013 | 3.118651 |
| H | 0.520243 | -5.900647 | 2.575145 | C | -0.981539 | -4.070303 | 1.267249 |
| C | -1.666936 | -2.927799 | 0.799435 | C | -3.021446 | -3.037952 | 0.430625 |
| C | -3.632655 | -4.297159 | 0.436535 | C | -2.953330 | -5.438380 | 0.829580 |
| C | -1.632442 | -5.296780 | 1.260432 | H | -1.098914 | -6.181413 | 1.578995 |
| C | -3.595187 | -6.826979 | 0.821449 | C | -5.040869 | -6.794130 | 0.317530 |
| H | -5.446300 | -7.808943 | 0.326434 | H | -5.675790 | -6.172789 | 0.954771 |
| H | -5.101307 | -6.416039 | -0.706539 | C | -2.782748 | -7.753651 | -0.097917 |
| H | -3.234197 | -8.749649 | -0.113078 | H | -1.750460 | -7.855373 | 0.243992 |
| H | -2.768314 | -7.366128 | -1.120349 | C | -3.593080 | -7.394569 | 2.250355 |
| H | -4.162744 | -6.747276 | 2.922826 | H | -4.052212 | -8.387140 | 2.255626 |
| H | -2.578683 | -7.488742 | 2.643976 | H | -4.660708 | -4.381416 | 0.119497 |
| C | -3.772568 | -1.815471 | 0.133946 | C | -5.177428 | -1.820184 | 0.041418 |
| H | -5.697170 | -2.764284 | 0.097959 | C | 4.490587 | -1.173066 | 2.173891 |
| C | 5.206848 | -0.010444 | 1.934357 | C | 4.572821 | 1.013071 | 1.230407 |
| H | 5.121925 | 1.921870 | 1.023069 | C | 6.651530 | 0.181370 | 2.399881 |
| C | 7.187308 | -1.040652 | 3.151087 | H | 8.216371 | -0.845682 | 3.462683 |
| H | 6.598901 | -1.252593 | 4.047847 | H | 7.190924 | -1.932791 | 2.519057 |
| C | 7.548676 | 0.429477 | 1.176132 | H | 8.584826 | 0.566515 | 1.498002 |
| H | 7.243513 | 1.323801 | 0.628383 | H | 7.510219 | -0.421948 | 0.490979 |
| C | 6.719745 | 1.397056 | 3.338550 | H | 7.749046 | 1.548055 | 3.675789 |
| H | 6.389667 | 2.309813 | 2.837656 | H | 6.087936 | 1.241053 | 4.217229 |
| H | 4.976502 | -1.976755 | 2.703757 | H | -2.375012 | 1.335239 | -1.719448 |
| H | -3.361537 | 1.554363 | 0.119247 | H | -5.765391 | 1.499564 | -0.038702 |
| C | -7.854975 | 0.091677 | -1.455363 | H | -8.944980 | 0.103506 | -1.542687 |
| H | -7.504495 | 1.125819 | -1.443516 | H | -7.443349 | -0.400310 | -2.341042 |
| C | -8.024948 | -2.072708 | -0.236907 | H | -9.110544 | -2.008662 | -0.344446 |
| H | -7.811376 | -2.636184 | 0.675197 | H | -7.632769 | -2.632471 | -1.090293 |
| C | -8.029904 | 0.061050 | 1.047928 | H | -7.746519 | -0.454680 | 1.969505 |
| H | -7.681453 | 1.094158 | 1.112230 | H | -9.121461 | 0.074503 | 0.981252 |
| Br | -3.714698 | 3.988575 | -2.145297 | Br | -1.133657 | 4.910756 | 1.249558 |

**Int31.out**

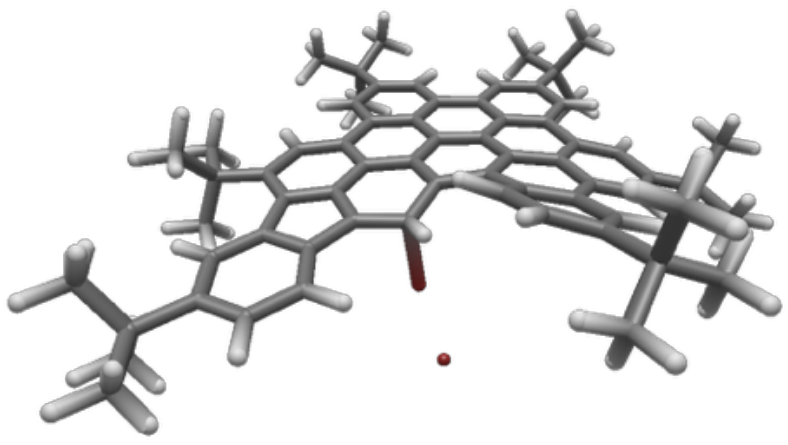

Basis set: def2-TZVP

Charge = 0, Multiplicity = 1

Electronic Energy = -7782.872931080577 Hartree

| | Cartesian Coordinates | | | | Cartesian Coordinates | | |
|---|---|---|---|---|---|---|---|
| Atoms | X | Y | Z | Atoms | X | Y | Z |
| C | -0.073667 | 0.080991 | -0.039253 | C | 1.452858 | 0.076964 | 0.002653 |
| C | 2.151954 | 1.293458 | 0.054749 | C | 3.525811 | 1.320277 | 0.058312 |
| C | 4.286314 | 0.139219 | -0.008895 | C | 5.752803 | 0.154852 | -0.029596 |
| C | 6.434716 | -0.964595 | 0.495357 | C | 7.825343 | -0.921058 | 0.722887 |
| C | 8.550461 | 0.227943 | 0.379054 | C | 7.892306 | 1.252822 | -0.319789 |
| C | 6.512401 | 1.200887 | -0.569672 | C | 5.933395 | 2.148421 | -1.588862 |
| C | 6.744154 | 3.342700 | -1.883452 | C | 8.073262 | 3.402918 | -1.475002 |
| C | 8.670447 | 2.382805 | -0.746569 | C | 10.024050 | 2.546823 | -0.427858 |
| C | 10.637173 | 3.722678 | -0.851234 | C | 10.027863 | 4.778850 | -1.579702 |
| C | 8.693259 | 4.598188 | -1.902590 | C | 7.606001 | 5.346758 | -2.638674 |
| C | 6.435942 | 4.535864 | -2.604020 | C | 5.260372 | 4.923742 | -3.215670 |
| C | 5.227955 | 6.146137 | -3.886904 | C | 6.351460 | 6.956978 | -3.936000 |
| C | 7.547397 | 6.544421 | -3.288927 | H | 8.405197 | 7.193059 | -3.350373 |
| C | 6.372330 | 8.283837 | -4.684321 | C | 5.029190 | 8.602621 | -5.344531 |
| H | 5.107117 | 9.560566 | -5.863623 | H | 4.752998 | 7.842827 | -6.079849 |
| H | 4.226520 | 8.683379 | -4.606908 | C | 6.713016 | 9.412705 | -3.696655 |
| H | 6.733036 | 10.366776 | -4.229785 | H | 7.689805 | 9.267359 | -3.230696 |
| H | 5.960366 | 9.475394 | -2.906078 | C | 7.451196 | 8.208832 | -5.779670 |
| H | 7.239718 | 7.391917 | -6.474182 | H | 7.465271 | 9.147503 | -6.339661 |
| H | 8.446667 | 8.049846 | -5.360093 | H | 4.312701 | 6.446611 | -4.376603 |
| H | 4.383079 | 4.286717 | -3.186686 | C | 10.848648 | 6.013728 | -1.954659 |
| C | 12.297001 | 5.941084 | -1.452935 | H | 12.811824 | 6.855254 | -1.757556 |
| H | 12.350999 | 5.875790 | -0.363091 | H | 12.837498 | 5.095782 | -1.886202 |
| C | 10.916142 | 6.126470 | -3.487944 | H | 11.540329 | 5.322853 | -3.886934 |
| H | 11.366628 | 7.082020 | -3.769063 | H | 9.946518 | 6.044155 | -3.973443 |
| C | 10.215433 | 7.252361 | -1.295728 | H | 9.149050 | 7.346414 | -1.488909 |
| H | 10.347349 | 7.198856 | -0.211727 | H | 10.711820 | 8.156916 | -1.656470 |
| H | 11.678416 | 3.851639 | -0.600467 | C | 10.701190 | 1.485861 | 0.325510 |
| C | 9.959208 | 0.335432 | 0.688393 | C | 10.605444 | -0.711900 | 1.367694 |
| C | 9.860383 | -1.937575 | 1.685835 | C | 8.502839 | -2.038998 | 1.330036 |
| C | 7.803525 | -3.237290 | 1.578315 | C | 8.455489 | -4.279583 | 2.223350 |
| C | 9.790430 | -4.189936 | 2.609857 | C | 10.474260 | -3.019173 | 2.321698 |
| H | 11.512711 | -2.945128 | 2.601596 | C | 10.446391 | -5.373553 | 3.321823 |

| | | | | | | | |
|---|---|---|---|---|---|---|---|
| C | 9.676459 | -5.672817 | 4.617994 | H | 10.137740 | -6.517906 | 5.136692 |
| H | 9.694177 | -4.807295 | 5.286127 | H | 8.633892 | -5.928236 | 4.416470 |
| C | 10.397399 | -6.604593 | 2.402455 | H | 10.864985 | -7.458947 | 2.900003 |
| H | 10.936066 | -6.411230 | 1.470714 | H | 9.369986 | -6.878789 | 2.152996 |
| C | 11.908714 | -5.097668 | 3.681724 | H | 12.003689 | -4.239386 | 4.352250 |
| H | 12.322600 | -5.970692 | 4.192234 | H | 12.514754 | -4.910967 | 2.791172 |
| H | 7.911997 | -5.186948 | 2.446543 | C | 6.405451 | -3.353252 | 1.144404 |
| C | 5.719329 | -2.205141 | 0.698533 | C | 4.361154 | -2.309482 | 0.342737 |
| C | 3.749002 | -3.561696 | 0.340330 | C | 4.428080 | -4.710608 | 0.720428 |
| C | 5.748709 | -4.578261 | 1.138793 | H | 6.286319 | -5.466424 | 1.439535 |
| C | 3.781057 | -6.095465 | 0.701784 | C | 2.329302 | -6.051097 | 0.217683 |
| H | 1.919189 | -7.063962 | 0.221406 | H | 1.705484 | -5.433771 | 0.869651 |
| H | 2.256126 | -5.662824 | -0.801655 | C | 4.578837 | -7.011026 | -0.240653 |
| H | 4.124795 | -8.005692 | -0.264147 | H | 5.615207 | -7.119777 | 0.086420 |
| H | 4.581152 | -6.609442 | -1.257717 | C | 3.800396 | -6.681993 | 2.122576 |
| H | 3.242519 | -6.041768 | 2.811578 | H | 3.337001 | -7.672595 | 2.121319 |
| H | 4.819442 | -6.785918 | 2.501515 | H | 2.719637 | -3.641207 | 0.027334 |
| C | 3.604666 | -1.079237 | 0.080256 | C | 2.199143 | -1.083169 | 0.050314 |
| H | 1.683023 | -2.029135 | 0.105314 | C | 11.956057 | -0.571649 | 1.702069 |
| C | 12.686890 | 0.558045 | 1.374252 | C | 12.038857 | 1.575309 | 0.675364 |
| H | 12.603227 | 2.455681 | 0.399654 | C | 14.162046 | 0.722295 | 1.740021 |
| C | 14.711779 | -0.490428 | 2.495436 | H | 15.764476 | -0.317531 | 2.731855 |
| H | 14.179076 | -0.655783 | 3.435744 | H | 14.647682 | -1.401958 | 1.895242 |
| C | 14.984548 | 0.906661 | 0.454685 | H | 16.042531 | 1.022630 | 0.705779 |
| H | 14.670387 | 1.792757 | -0.101145 | H | 14.878980 | 0.036830 | -0.199450 |
| C | 14.321532 | 1.964099 | 2.631936 | H | 15.373381 | 2.095900 | 2.900520 |
| H | 13.986425 | 2.870175 | 2.122210 | H | 13.741760 | 1.854563 | 3.552579 |
| H | 12.451133 | -1.370124 | 2.230902 | H | 4.885628 | 2.382457 | -1.481781 |
| H | 4.019205 | 2.275742 | 0.182340 | H | 1.611679 | 2.231035 | 0.116390 |
| C | -0.535466 | 0.871319 | -1.274207 | H | -1.628067 | 0.887084 | -1.318041 |
| H | -0.182765 | 1.904451 | -1.243726 | H | -0.160716 | 0.408198 | -2.191235 |
| C | -0.656322 | -1.332050 | -0.123693 | H | -1.745483 | -1.267682 | -0.185219 |
| H | -0.405774 | -1.926574 | 0.758855 | H | -0.298264 | -1.860767 | -1.011093 |
| C | -0.613917 | 0.756941 | 1.231119 | H | -0.292993 | 0.212393 | 2.123422 |
| H | -0.266096 | 1.788864 | 1.315857 | H | -1.707305 | 0.768788 | 1.209210 |
| Br | 6.000629 | 1.216486 | -3.336710 | Br | 8.440748 | 3.735424 | -5.980074 |

**Int32.out**

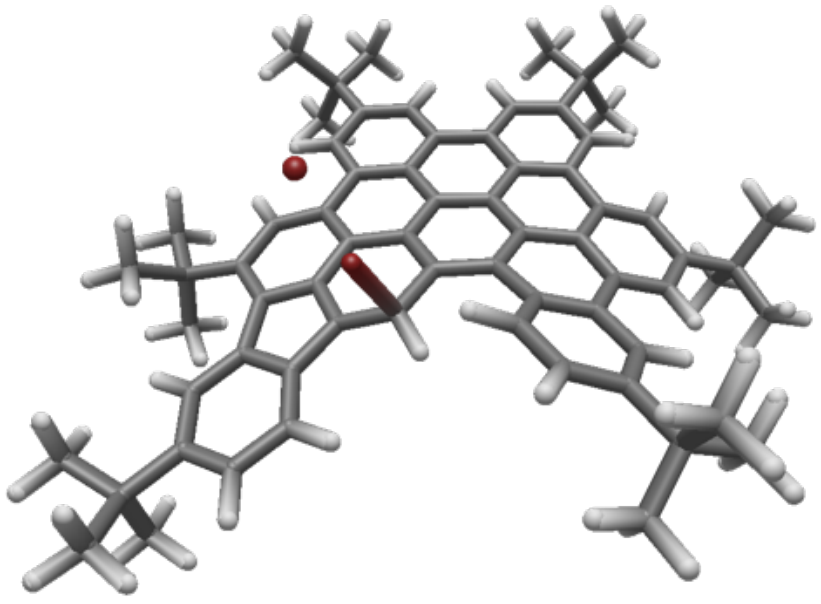

Basis set: def2-TZVP

Charge = 0, Multiplicity = 1

Electronic Energy = -7782.869620216245 Hartree

| | Cartesian Coordinates | | | | Cartesian Coordinates | | |
|---|---|---|---|---|---|---|---|
| Atoms | X | Y | Z | Atoms | X | Y | Z |
| C | -0.144104 | -0.111232 | -0.240952 | C | 1.365389 | -0.015881 | -0.034450 |
| C | 1.963113 | 1.224657 | 0.217145 | C | 3.324497 | 1.339657 | 0.373839 |
| C | 4.191503 | 0.235814 | 0.279619 | C | 5.662667 | 0.340025 | 0.359253 |
| C | 6.380697 | -0.828981 | 0.727767 | C | 7.770617 | -0.786873 | 0.968736 |
| C | 8.493065 | 0.378596 | 0.685206 | C | 7.809048 | 1.478645 | 0.147106 |
| C | 6.406122 | 1.491311 | 0.029782 | C | 5.767667 | 2.753819 | -0.481620 |
| C | 6.643231 | 3.666669 | -1.237975 | C | 8.021707 | 3.566065 | -1.115712 |
| C | 8.611858 | 2.555813 | -0.371222 | C | 10.004900 | 2.603926 | -0.262077 |
| C | 10.668420 | 3.610463 | -0.959647 | C | 10.071973 | 4.614283 | -1.765423 |
| C | 8.687477 | 4.593155 | -1.819479 | C | 7.575554 | 5.394558 | -2.456615 |
| C | 6.343292 | 4.793674 | -2.073092 | C | 5.126992 | 5.295829 | -2.484738 |
| C | 5.112503 | 6.426493 | -3.304498 | C | 6.295358 | 7.033631 | -3.695421 |
| C | 7.536973 | 6.498153 | -3.260154 | H | 8.439820 | 6.991969 | -3.578390 |
| C | 6.329736 | 8.273752 | -4.580969 | C | 4.928831 | 8.756768 | -4.962317 |
| H | 5.018194 | 9.644875 | -5.592000 | H | 4.380648 | 7.997904 | -5.526480 |
| H | 4.343127 | 9.027142 | -4.080075 | C | 7.048515 | 9.404323 | -3.824337 |
| H | 7.071182 | 10.301416 | -4.448514 | H | 8.078605 | 9.139528 | -3.576911 |
| H | 6.523294 | 9.642363 | -2.895707 | C | 7.101922 | 7.941469 | -5.869519 |
| H | 6.608721 | 7.134419 | -6.417916 | H | 7.135167 | 8.825186 | -6.512072 |
| H | 8.129780 | 7.637301 | -5.660947 | H | 4.159687 | 6.822396 | -3.626275 |
| H | 4.199074 | 4.828289 | -2.173546 | C | 10.957994 | 5.611353 | -2.515298 |
| C | 12.455064 | 5.355224 | -2.296280 | H | 13.017229 | 6.096799 | -2.868662 |
| H | 12.738831 | 5.461957 | -1.246308 | H | 12.757254 | 4.364894 | -2.646862 |
| C | 10.702103 | 5.461592 | -4.025592 | H | 11.063690 | 4.487317 | -4.365387 |
| H | 11.245909 | 6.237561 | -4.570523 | H | 9.649689 | 5.529658 | -4.293389 |
| C | 10.674852 | 7.036346 | -2.010140 | H | 9.616721 | 7.286114 | -1.988002 |
| H | 11.045795 | 7.146045 | -0.988680 | H | 11.191343 | 7.760819 | -2.645493 |
| H | 11.746094 | 3.609733 | -0.903594 | C | 10.679529 | 1.563117 | 0.513921 |
| C | 9.921008 | 0.440629 | 0.919009 | C | 10.569342 | -0.617094 | 1.576891 |
| C | 9.816684 | -1.838776 | 1.884487 | C | 8.464861 | -1.933583 | 1.505351 |
| C | 7.784608 | -3.152738 | 1.677109 | C | 8.425221 | -4.209385 | 2.310356 |
| C | 9.742952 | -4.114686 | 2.749474 | C | 10.423469 | -2.931080 | 2.507907 |
| H | 11.454362 | -2.854779 | 2.814358 | C | 10.386891 | -5.306704 | 3.457951 |

| | | | | | | | |
|---|---|---|---|---|---|---|---|
| C | 9.577255 | -5.637541 | 4.721992 | H | 10.028256 | -6.489062 | 5.239232 |
| H | 9.565878 | -4.785110 | 5.406810 | H | 8.543958 | -5.896686 | 4.480960 |
| C | 10.379536 | -6.520156 | 2.514402 | H | 10.839283 | -7.379471 | 3.010724 |
| H | 10.946018 | -6.304427 | 1.604260 | H | 9.363544 | -6.799406 | 2.226853 |
| C | 11.833908 | -5.024718 | 3.870937 | H | 11.898529 | -4.178295 | 4.559828 |
| H | 12.239185 | -5.903363 | 4.378732 | H | 12.466998 | -4.815974 | 3.004428 |
| H | 7.884317 | -5.129739 | 2.481632 | C | 6.419234 | -3.274457 | 1.160394 |
| C | 5.714495 | -2.114112 | 0.775910 | C | 4.384853 | -2.241433 | 0.340324 |
| C | 3.831448 | -3.513134 | 0.185809 | C | 4.535228 | -4.665267 | 0.499865 |
| C | 5.821346 | -4.518998 | 1.009669 | H | 6.384082 | -5.406619 | 1.263014 |
| C | 3.950678 | -6.065918 | 0.318740 | C | 2.528417 | -6.032292 | -0.246860 |
| H | 2.163451 | -7.055958 | -0.360896 | H | 1.841928 | -5.503123 | 0.419490 |
| H | 2.497199 | -5.552688 | -1.228790 | C | 4.840135 | -6.860541 | -0.650838 |
| H | 4.431390 | -7.865147 | -0.791377 | H | 5.859313 | -6.959845 | -0.271016 |
| H | 4.884462 | -6.367416 | -1.625856 | C | 3.915659 | -6.777796 | 1.680549 |
| H | 3.291919 | -6.225116 | 2.388547 | H | 3.498092 | -7.781937 | 1.564504 |
| H | 4.915660 | -6.874742 | 2.108854 | H | 2.826847 | -3.607517 | -0.193196 |
| C | 3.587631 | -1.025706 | 0.152449 | C | 2.197240 | -1.116378 | -0.031852 |
| H | 1.753693 | -2.092816 | -0.141619 | C | 11.929179 | -0.496778 | 1.880532 |
| C | 12.669387 | 0.622676 | 1.536829 | C | 12.024294 | 1.641016 | 0.836622 |
| H | 12.587796 | 2.518912 | 0.548594 | C | 14.150017 | 0.773040 | 1.885103 |
| C | 14.697327 | -0.444826 | 2.633849 | H | 15.754408 | -0.281981 | 2.857570 |
| H | 14.174513 | -0.604924 | 3.580540 | H | 14.617168 | -1.355863 | 2.034709 |
| C | 14.959765 | 0.950850 | 0.590789 | H | 16.021542 | 1.056808 | 0.830010 |
| H | 14.647741 | 1.840483 | 0.039456 | H | 14.838857 | 0.082480 | -0.062778 |
| C | 14.329377 | 2.013329 | 2.775286 | H | 15.385257 | 2.136934 | 3.031784 |
| H | 13.994959 | 2.921923 | 2.269748 | H | 13.759292 | 1.908213 | 3.702393 |
| H | 12.423144 | -1.302494 | 2.399540 | H | 4.805421 | 2.606119 | -0.959098 |
| H | 3.697905 | 2.311044 | 0.643009 | H | 1.356163 | 2.117909 | 0.309904 |
| C | -0.534062 | 0.746389 | -1.455529 | H | -1.614731 | 0.695285 | -1.615636 |
| H | -0.262177 | 1.794172 | -1.309216 | H | -0.034727 | 0.385485 | -2.359064 |
| C | -0.606677 | -1.548502 | -0.492341 | H | -1.687962 | -1.555130 | -0.650507 |
| H | -0.388610 | -2.196956 | 0.360418 | H | -0.133305 | -1.974075 | -1.381304 |
| C | -0.861997 | 0.419535 | 1.010268 | H | -0.591032 | -0.170855 | 1.889887 |
| H | -0.608623 | 1.463419 | 1.206940 | H | -1.944973 | 0.354595 | 0.872287 |
| Br | 5.442922 | 3.994480 | 1.040824 | Br | 8.337535 | 6.841647 | 1.299657 |

## P.out

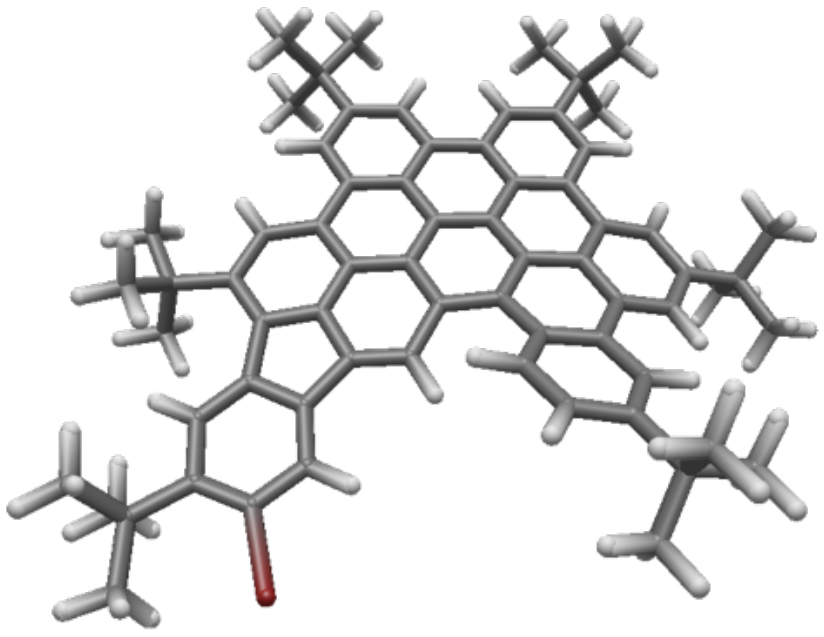

Basis set: def2-TZVP

Charge = 0, Multiplicity = 1

Electronic Energy = -5208.218340041991 Hartree

| | Cartesian Coordinates | | | | Cartesian Coordinates | | |
|---|---|---|---|---|---|---|---|
| Atoms | X | Y | Z | Atoms | X | Y | Z |
| C | -0.047263 | 0.023290 | 0.032000 | C | 1.481022 | 0.022231 | 0.044173 |
| C | 2.179614 | 1.242032 | 0.048235 | C | 3.553476 | 1.273939 | 0.032308 |
| C | 4.322712 | 0.092689 | -0.001729 | C | 5.788843 | 0.113221 | -0.021494 |
| C | 6.470881 | -1.002301 | 0.486948 | C | 7.869984 | -0.947641 | 0.740045 |
| C | 8.583551 | 0.215544 | 0.445533 | C | 7.925458 | 1.274117 | -0.219929 |
| C | 6.544134 | 1.218133 | -0.515711 | C | 5.973927 | 2.236879 | -1.376591 |
| C | 6.723242 | 3.291221 | -1.775751 | C | 8.089074 | 3.398847 | -1.369259 |
| C | 8.701290 | 2.407851 | -0.623801 | C | 10.064593 | 2.567764 | -0.322984 |
| C | 10.697429 | 3.721379 | -0.787998 | C | 10.079319 | 4.726784 | -1.558520 |
| C | 8.720827 | 4.550052 | -1.871262 | C | 7.658238 | 5.254312 | -2.669467 |
| C | 6.473411 | 4.475654 | -2.611188 | C | 5.335133 | 4.884832 | -3.263161 |
| C | 5.340446 | 6.078965 | -3.993869 | C | 6.475894 | 6.888817 | -4.084892 |
| C | 7.620319 | 6.426675 | -3.399185 | H | 8.501094 | 7.041209 | -3.464540 |
| C | 6.689751 | 8.227842 | -4.828417 | C | 5.487247 | 8.810121 | -5.577434 |
| H | 5.807259 | 9.752891 | -6.029369 | H | 5.141905 | 8.156826 | -6.378058 |
| H | 4.650917 | 9.024885 | -4.912499 | C | 7.124468 | 9.292129 | -3.801721 |
| H | 7.283596 | 10.245052 | -4.313575 | H | 8.051865 | 9.029017 | -3.290922 |
| H | 6.346593 | 9.434860 | -3.046577 | C | 7.807732 | 8.029987 | -5.870253 |
| H | 7.516282 | 7.272533 | -6.603021 | H | 7.981014 | 8.970677 | -6.399950 |
| H | 8.751207 | 7.721452 | -5.417208 | Br | 3.649693 | 6.424118 | -4.812454 |
| H | 4.430393 | 4.290489 | -3.216721 | C | 10.900440 | 5.934540 | -2.020781 |
| C | 12.353398 | 5.902042 | -1.526348 | H | 12.862307 | 6.799057 | -1.888858 |
| H | 12.413520 | 5.901616 | -0.435159 | H | 12.895945 | 5.034069 | -1.908849 |
| C | 10.963836 | 5.942242 | -3.559220 | H | 11.586639 | 5.111282 | -3.901698 |
| H | 11.414827 | 6.874235 | -3.911759 | H | 9.989756 | 5.831469 | -4.030335 |
| C | 10.274581 | 7.219062 | -1.446862 | H | 9.202044 | 7.286452 | -1.614747 |
| H | 10.436641 | 7.251414 | -0.365914 | H | 10.750270 | 8.098985 | -1.889217 |
| H | 11.742795 | 3.848967 | -0.554990 | C | 10.729062 | 1.499504 | 0.437806 |
| C | 9.990820 | 0.331925 | 0.771666 | C | 10.642339 | -0.725179 | 1.435240 |
| C | 9.904833 | -1.966134 | 1.714390 | C | 8.551081 | -2.073822 | 1.336118 |
| C | 7.860550 | -3.287170 | 1.555168 | C | 8.516954 | -4.335457 | 2.185301 |
| C | 9.849273 | -4.241047 | 2.589731 | C | 10.523258 | -3.057698 | 2.336540 |
| H | 11.556909 | -2.977154 | 2.632955 | C | 10.507608 | -5.433892 | 3.286352 |

| | | | | | | | |
|---|---|---|---|---|---|---|---|
| C | 9.729282 | -5.761415 | 4.571062 | H | 10.191351 | -6.612911 | 5.078738 |
| H | 9.736262 | -4.907645 | 5.254394 | H | 8.689821 | -6.019073 | 4.356342 |
| C | 10.475008 | -6.650130 | 2.346433 | H | 10.943758 | -7.509833 | 2.833815 |
| H | 11.020491 | -6.438002 | 1.422678 | H | 9.451616 | -6.926688 | 2.083303 |
| C | 11.965239 | -5.154979 | 3.663584 | H | 12.048571 | -4.309211 | 4.351337 |
| H | 12.381664 | -6.034844 | 4.160349 | H | 12.576655 | -4.946052 | 2.781639 |
| H | 7.980683 | -5.252746 | 2.385139 | C | 6.458142 | -3.406035 | 1.118802 |
| C | 5.764132 | -2.253359 | 0.686601 | C | 4.398838 | -2.358423 | 0.358514 |
| C | 3.786291 | -3.616007 | 0.358114 | C | 4.473944 | -4.765554 | 0.713154 |
| C | 5.804366 | -4.632087 | 1.111343 | H | 6.347150 | -5.521699 | 1.399558 |
| C | 3.831437 | -6.153893 | 0.693538 | C | 2.371183 | -6.110809 | 0.234819 |
| H | 1.964680 | -7.125238 | 0.235201 | H | 1.756442 | -5.502173 | 0.903389 |
| H | 2.279062 | -5.712357 | -0.779094 | C | 4.615257 | -7.059306 | -0.270543 |
| H | 4.165517 | -8.056079 | -0.293667 | H | 5.658078 | -7.165486 | 0.036553 |
| H | 4.597257 | -6.649710 | -1.284344 | C | 3.876617 | -6.754536 | 2.108059 |
| H | 3.327202 | -6.123523 | 2.812278 | H | 3.418110 | -7.747561 | 2.105291 |
| H | 4.902281 | -6.856752 | 2.469453 | H | 2.750996 | -3.693469 | 0.063530 |
| C | 3.639599 | -1.128038 | 0.111050 | C | 2.231211 | -1.134773 | 0.101938 |
| H | 1.719148 | -2.081469 | 0.182220 | C | 11.988863 | -0.578351 | 1.794249 |
| C | 12.710394 | 0.568625 | 1.506276 | C | 12.060440 | 1.593323 | 0.816749 |
| H | 12.615033 | 2.489074 | 0.570340 | C | 14.176974 | 0.745107 | 1.905413 |
| C | 14.730460 | -0.477426 | 2.642701 | H | 15.775690 | -0.295161 | 2.904644 |
| H | 14.181456 | -0.673475 | 3.567655 | H | 14.691778 | -1.375204 | 2.019905 |
| C | 15.023577 | 0.972795 | 0.642571 | H | 16.074425 | 1.099932 | 0.917884 |
| H | 14.705560 | 1.866437 | 0.101144 | H | 14.945840 | 0.116948 | -0.033767 |
| C | 14.302140 | 1.966996 | 2.830097 | H | 15.346775 | 2.107912 | 3.121937 |
| H | 13.962265 | 2.879964 | 2.335989 | H | 13.706803 | 1.826542 | 3.736560 |
| H | 12.484850 | -1.383856 | 2.312189 | H | 4.960909 | 2.122146 | -1.735356 |
| H | 4.046448 | 2.234070 | 0.095303 | H | 1.637036 | 2.180092 | 0.083972 |
| C | -0.536123 | 0.765875 | -1.222349 | H | -1.629552 | 0.781143 | -1.243659 |
| H | -0.182310 | 1.799051 | -1.238900 | H | -0.180853 | 0.267635 | -2.128752 |
| C | -0.630259 | -1.392273 | 0.013763 | H | -1.720897 | -1.330992 | -0.021590 |
| H | -0.355315 | -1.954128 | 0.910414 | H | -0.294926 | -1.953274 | -0.862705 |
| C | -0.564827 | 0.746879 | 1.285839 | H | -0.225596 | 0.237635 | 2.192188 |
| H | -0.217036 | 1.781540 | 1.323834 | H | -1.658580 | 0.756866 | 1.284912 |

**P2.out**

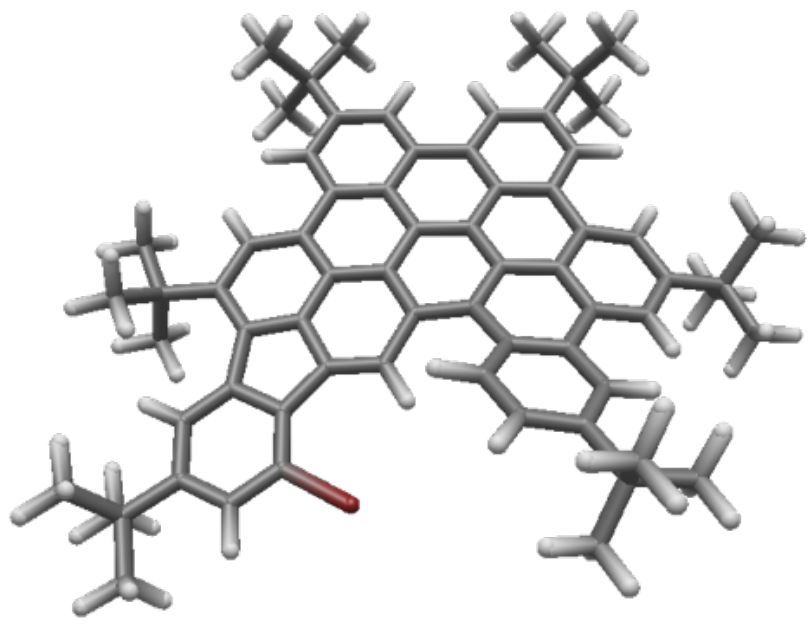

`Basis set: def2-TZVP`

Charge = 0, Multiplicity = 1

Electronic Energy = -5208.232376002050 Hartree

| | Cartesian Coordinates | | | | Cartesian Coordinates | | |
|---|---|---|---|---|---|---|---|
| Atoms | *X* | *Y* | *Z* | Atoms | *X* | *Y* | *Z* |
| C | -0.046452 | 0.028869 | -0.020610 | C | 1.481050 | 0.027247 | 0.026509 |
| C | 2.178951 | 1.247167 | 0.051350 | C | 3.552518 | 1.278925 | 0.059726 |
| C | 4.322385 | 0.097849 | 0.029323 | C | 5.788442 | 0.117661 | 0.016748 |
| C | 6.468931 | -1.001611 | 0.518526 | C | 7.868932 | -0.950439 | 0.768312 |
| C | 8.584734 | 0.211945 | 0.475691 | C | 7.926569 | 1.275350 | -0.180885 |
| C | 6.546194 | 1.224131 | -0.470345 | C | 5.971369 | 2.246721 | -1.321271 |
| C | 6.709372 | 3.313194 | -1.718482 | C | 8.081524 | 3.409646 | -1.319469 |
| C | 8.698154 | 2.410966 | -0.585370 | C | 10.063783 | 2.563257 | -0.293257 |
| C | 10.699117 | 3.716014 | -0.756017 | C | 10.080493 | 4.727639 | -1.515459 |
| C | 8.717004 | 4.558412 | -1.817413 | C | 7.658603 | 5.280038 | -2.602706 |
| C | 6.457278 | 4.509528 | -2.540192 | C | 5.331486 | 4.980842 | -3.185326 |
| C | 5.351519 | 6.175651 | -3.901949 | C | 6.515835 | 6.928926 | -3.976677 |
| C | 7.662661 | 6.466655 | -3.315945 | H | 8.555679 | 7.065645 | -3.379274 |
| C | 6.593349 | 8.245651 | -4.752961 | C | 5.263137 | 8.604900 | -5.420111 |
| H | 5.376824 | 9.546779 | -5.962395 | H | 4.953097 | 7.839467 | -6.136409 |
| H | 4.465445 | 8.734959 | -4.683940 | C | 6.975304 | 9.380238 | -3.788279 |
| H | 7.030431 | 10.327035 | -4.332969 | H | 7.946667 | 9.202660 | -3.321147 |
| H | 6.228249 | 9.481382 | -2.996163 | C | 7.667229 | 8.121415 | -5.846488 |
| H | 7.416435 | 7.316208 | -6.542479 | H | 7.733314 | 9.056356 | -6.410049 |
| H | 8.651709 | 7.912235 | -5.421911 | H | 4.444526 | 6.498976 | -4.393162 |
| Br | 3.690426 | 4.030186 | -3.121579 | C | 10.909619 | 5.931644 | -1.974953 |
| C | 12.365705 | 5.883477 | -1.489274 | H | 12.879098 | 6.778912 | -1.849441 |
| H | 12.432695 | 5.876062 | -0.398572 | H | 12.899276 | 5.014004 | -1.880772 |
| C | 10.969973 | 5.946533 | -3.514158 | H | 11.596720 | 5.119725 | -3.859381 |
| H | 11.417287 | 6.881579 | -3.863349 | H | 9.997149 | 5.832603 | -3.986599 |
| C | 10.302761 | 7.218500 | -1.384301 | H | 9.227257 | 7.293861 | -1.526251 |
| H | 10.489922 | 7.245669 | -0.307302 | H | 10.774218 | 8.097115 | -1.833790 |
| H | 11.746575 | 3.836604 | -0.530078 | C | 10.731439 | 1.489668 | 0.457690 |
| C | 9.993539 | 0.322797 | 0.793274 | C | 10.646360 | -0.738510 | 1.448439 |
| C | 9.905907 | -1.977178 | 1.729791 | C | 8.549654 | -2.079806 | 1.358522 |
| C | 7.856267 | -3.291254 | 1.578923 | C | 8.512478 | -4.342410 | 2.204494 |
| C | 9.847068 | -4.252895 | 2.602437 | C | 10.523819 | -3.071535 | 2.347359 |
| H | 11.559231 | -2.994724 | 2.638604 | C | 10.504967 | -5.448967 | 3.293881 |

| | | | | | | | |
|---|---|---|---|---|---|---|---|
| C | 9.731302 | -5.776652 | 4.581343 | H | 10.192896 | -6.630610 | 5.085311 |
| H | 9.743902 | -4.924286 | 5.266346 | H | 8.690119 | -6.030641 | 4.370597 |
| C | 10.464668 | -6.663277 | 2.351761 | H | 10.933255 | -7.525227 | 2.835314 |
| H | 11.006513 | -6.450815 | 1.425945 | H | 9.439327 | -6.936484 | 2.092732 |
| C | 11.965063 | -5.175048 | 3.665195 | H | 12.053885 | -4.330859 | 4.354199 |
| H | 12.381079 | -6.057088 | 4.158437 | H | 12.573245 | -4.966183 | 2.780993 |
| H | 7.974595 | -5.258426 | 2.405640 | C | 6.452018 | -3.405883 | 1.146902 |
| C | 5.760051 | -2.251820 | 0.714744 | C | 4.394921 | -2.354407 | 0.384069 |
| C | 3.779530 | -3.610790 | 0.387436 | C | 4.463937 | -4.760993 | 0.746204 |
| C | 5.795263 | -4.630223 | 1.142065 | H | 6.336046 | -5.520806 | 1.430849 |
| C | 3.817763 | -6.147723 | 0.732193 | C | 2.355436 | -6.102058 | 0.280366 |
| H | 1.945922 | -7.115319 | 0.286948 | H | 1.745984 | -5.488901 | 0.949649 |
| H | 2.259339 | -5.707312 | -0.734627 | C | 4.595014 | -7.056773 | -0.233778 |
| H | 4.142995 | -8.052611 | -0.252888 | H | 5.639038 | -7.164568 | 0.068677 |
| H | 4.573177 | -6.649050 | -1.248257 | C | 3.867772 | -6.745951 | 2.147615 |
| H | 3.323290 | -6.112155 | 2.853166 | H | 3.406397 | -7.737646 | 2.148606 |
| H | 4.894689 | -6.850577 | 2.504711 | H | 2.743989 | -3.687230 | 0.093810 |
| C | 3.638321 | -1.123552 | 0.129204 | C | 2.230084 | -1.129944 | 0.095003 |
| H | 1.715869 | -2.076619 | 0.159842 | C | 11.996242 | -0.597350 | 1.796554 |
| C | 12.718735 | 0.548528 | 1.506274 | C | 12.066174 | 1.578042 | 0.826406 |
| H | 12.621827 | 2.472923 | 0.579490 | C | 14.189429 | 0.718871 | 1.892882 |
| C | 14.746261 | -0.509153 | 2.618421 | H | 15.794640 | -0.331605 | 2.870836 |
| H | 14.205854 | -0.708132 | 3.547804 | H | 14.698328 | -1.403646 | 1.991549 |
| C | 15.024990 | 0.951264 | 0.623534 | H | 16.078621 | 1.074446 | 0.889846 |
| H | 14.704047 | 1.848597 | 0.089998 | H | 14.938923 | 0.099271 | -0.056647 |
| C | 14.326465 | 1.935401 | 2.822933 | H | 15.374046 | 2.071568 | 3.106375 |
| H | 13.985151 | 2.852015 | 2.336615 | H | 13.738619 | 1.791972 | 3.733804 |
| H | 12.494168 | -1.406341 | 2.307182 | H | 4.961539 | 2.119261 | -1.672126 |
| H | 4.043220 | 2.239216 | 0.134700 | H | 1.636400 | 2.185444 | 0.081456 |
| C | -0.505683 | 0.785207 | -1.278060 | H | -1.598308 | 0.800481 | -1.325291 |
| H | -0.151971 | 1.818510 | -1.274909 | H | -0.128619 | 0.297033 | -2.181133 |
| C | -0.628952 | -1.386202 | -0.068021 | H | -1.718340 | -1.324253 | -0.130538 |
| H | -0.377035 | -1.957259 | 0.829580 | H | -0.271188 | -1.938172 | -0.941338 |
| C | -0.592798 | 0.739108 | 1.228582 | H | -0.275382 | 0.219496 | 2.136944 |
| H | -0.245058 | 1.772917 | 1.286261 | H | -1.686233 | 0.750306 | 1.202019 |

**P3.out**

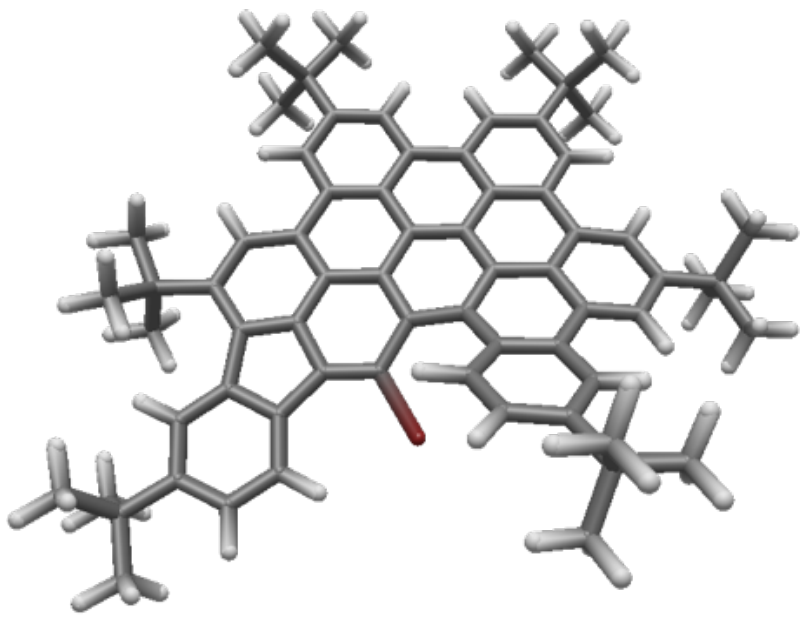

Basis set: def2-TZVP

Charge = 0, Multiplicity = 1

Electronic Energy = -5208.218553922124 Hartree

| | Cartesian Coordinates | | | | Cartesian Coordinates | | |
|---|---|---|---|---|---|---|---|
| Atoms | *X* | *Y* | *Z* | Atoms | *X* | *Y* | *Z* |
| C | -0.034806 | 0.138204 | 0.314324 | C | 1.485583 | 0.072181 | 0.172366 |
| C | 2.225075 | 1.262604 | 0.044878 | C | 3.588971 | 1.236772 | -0.111458 |
| C | 4.301426 | 0.023619 | -0.154319 | C | 5.760531 | -0.000934 | -0.268734 |
| C | 6.433340 | -1.090016 | 0.305125 | C | 7.821232 | -1.017724 | 0.591757 |
| C | 8.513235 | 0.166447 | 0.332132 | C | 7.867780 | 1.198040 | -0.388021 |
| C | 6.525521 | 1.071389 | -0.812315 | C | 6.045227 | 2.022330 | -1.813310 |
| C | 6.719516 | 3.170838 | -2.074944 | C | 8.003370 | 3.366676 | -1.456654 |
| C | 8.609255 | 2.382375 | -0.695698 | C | 9.927405 | 2.602495 | -0.262249 |
| C | 10.525642 | 3.813696 | -0.607856 | C | 9.924710 | 4.810125 | -1.399261 |
| C | 8.619469 | 4.563387 | -1.861258 | C | 7.634889 | 5.219008 | -2.785596 |
| C | 6.511956 | 4.355312 | -2.931506 | C | 5.444264 | 4.731068 | -3.727069 |
| C | 5.478661 | 5.953690 | -4.398385 | C | 6.565724 | 6.812417 | -4.280975 |
| C | 7.637945 | 6.427153 | -3.463622 | H | 8.471553 | 7.103749 | -3.373831 |
| C | 6.642156 | 8.160667 | -5.000910 | C | 5.397349 | 8.437465 | -5.847958 |
| H | 5.505206 | 9.408080 | -6.338679 | H | 5.262803 | 7.680614 | -6.625223 |
| H | 4.492681 | 8.468901 | -5.235026 | C | 6.779525 | 9.284482 | -3.960278 |
| H | 6.832064 | 10.253839 | -4.464244 | H | 7.683454 | 9.167323 | -3.358322 |
| H | 5.918556 | 9.292782 | -3.286087 | C | 7.870467 | 8.170676 | -5.925847 |
| H | 7.796373 | 7.375994 | -6.673447 | H | 7.936131 | 9.129718 | -6.447713 |
| H | 8.797105 | 8.026616 | -5.365510 | H | 4.632494 | 6.222571 | -5.017232 |
| H | 4.579412 | 4.091369 | -3.834397 | C | 10.714160 | 6.082291 | -1.727197 |
| C | 12.098485 | 6.121454 | -1.063533 | H | 12.582156 | 7.064746 | -1.330640 |
| H | 12.029569 | 6.078123 | 0.026244 | H | 12.742288 | 5.308716 | -1.408402 |
| C | 10.962176 | 6.148164 | -3.246189 | H | 11.687056 | 5.379434 | -3.527540 |
| H | 11.377684 | 7.123092 | -3.516871 | H | 10.065622 | 5.980138 | -3.838010 |
| C | 9.947003 | 7.304532 | -1.187578 | H | 8.893522 | 7.307003 | -1.457076 |
| H | 10.001732 | 7.312228 | -0.095479 | H | 10.402059 | 8.227811 | -1.557408 |
| H | 11.530567 | 3.994368 | -0.261202 | C | 10.594000 | 1.535298 | 0.498341 |
| C | 9.890981 | 0.326940 | 0.746636 | C | 10.548109 | -0.729587 | 1.406573 |
| C | 9.845693 | -2.005762 | 1.609055 | C | 8.502372 | -2.134090 | 1.202468 |
| C | 7.824348 | -3.359621 | 1.396673 | C | 8.501288 | -4.416540 | 1.989691 |
| C | 9.833295 | -4.313316 | 2.393594 | C | 10.483439 | -3.106361 | 2.194327 |
| H | 11.513401 | -3.016002 | 2.501073 | C | 10.514430 | -5.520806 | 3.041499 |

| | | | | | | | |
|---|---|---|---|---|---|---|---|
| C | 9.751921 | -5.903842 | 4.320401 | H | 10.228916 | -6.766750 | 4.793917 |
| H | 9.754382 | -5.074622 | 5.033332 | H | 8.713842 | -6.166850 | 4.105340 |
| C | 10.489856 | -6.703406 | 2.059461 | H | 10.976710 | -7.572379 | 2.511297 |
| H | 11.022033 | -6.450727 | 1.138130 | H | 9.468032 | -6.986423 | 1.797215 |
| C | 11.971443 | -5.234888 | 3.415780 | H | 12.049168 | -4.416692 | 4.136680 |
| H | 12.406134 | -6.127544 | 3.872444 | H | 12.570487 | -4.980868 | 2.537162 |
| H | 7.983054 | -5.349685 | 2.160668 | C | 6.409025 | -3.473744 | 0.998061 |
| C | 5.714918 | -2.322030 | 0.564716 | C | 4.333656 | -2.407193 | 0.305153 |
| C | 3.697321 | -3.650246 | 0.364837 | C | 4.382886 | -4.804413 | 0.712315 |
| C | 5.732550 | -4.687198 | 1.046891 | H | 6.268147 | -5.580174 | 1.337740 |
| C | 3.716370 | -6.181012 | 0.755876 | C | 2.235275 | -6.120161 | 0.372343 |
| H | 1.810944 | -7.126299 | 0.416896 | H | 1.667649 | -5.485084 | 1.057722 |
| H | 2.097686 | -5.742153 | -0.644235 | C | 4.433726 | -7.119374 | -0.228175 |
| H | 3.966987 | -8.108366 | -0.207428 | H | 5.489186 | -7.237387 | 0.027173 |
| H | 4.370125 | -6.729734 | -1.247982 | C | 3.824546 | -6.754105 | 2.178374 |
| H | 3.322685 | -6.099769 | 2.896654 | H | 3.349939 | -7.738662 | 2.219390 |
| H | 4.865583 | -6.867520 | 2.488763 | H | 2.646307 | -3.710269 | 0.124849 |
| C | 3.592744 | -1.162866 | 0.075884 | C | 2.191083 | -1.114221 | 0.203960 |
| H | 1.657791 | -2.037009 | 0.377013 | C | 11.865400 | -0.542989 | 1.845215 |
| C | 12.552517 | 0.642276 | 1.638912 | C | 11.898401 | 1.666755 | 0.953064 |
| H | 12.430086 | 2.590776 | 0.769113 | C | 13.988885 | 0.859562 | 2.119772 |
| C | 14.547056 | -0.361326 | 2.856339 | H | 15.568147 | -0.147423 | 3.181897 |
| H | 13.955085 | -0.602965 | 3.743245 | H | 14.578677 | -1.242596 | 2.210026 |
| C | 14.889549 | 1.146751 | 0.907270 | H | 15.920302 | 1.303221 | 1.237985 |
| H | 14.567010 | 2.041502 | 0.370334 | H | 14.875675 | 0.305033 | 0.209273 |
| C | 14.024335 | 2.061354 | 3.077800 | H | 15.047187 | 2.228706 | 3.427071 |
| H | 13.680136 | 2.975307 | 2.588633 | H | 13.388363 | 1.879097 | 3.948555 |
| H | 12.364416 | -1.345974 | 2.364093 | Br | 4.647858 | 1.501654 | -2.988932 |
| H | 4.127896 | 2.173588 | -0.180176 | H | 1.720823 | 2.221781 | 0.077885 |
| C | -0.623011 | 0.822286 | -0.930611 | H | -1.711660 | 0.882753 | -0.843416 |
| H | -0.235436 | 1.836353 | -1.051046 | H | -0.380733 | 0.254093 | -1.833091 |
| C | -0.667369 | -1.249774 | 0.447495 | H | -1.752236 | -1.144144 | 0.527830 |
| H | -0.313424 | -1.769440 | 1.341817 | H | -0.452727 | -1.874326 | -0.423852 |
| C | -0.395235 | 0.957385 | 1.564259 | H | 0.018286 | 0.491005 | 2.462840 |
| H | -0.011514 | 1.977869 | 1.500819 | H | -1.482327 | 1.010561 | 1.673222 |
